# Supplementary material for: DART-ID increases single-cell proteome coverage
Source: PLoS Comput Biol. 2019 Jul 1;15(7):e1007082. doi: 10.1371/journal.pcbi.1007082 (PMC6625733; doi:10.1371/journal.pcbi.1007082)
Supplement: S1 File — A optional HTML report generated by the dart_id Python script. The report gives a summary of the alignment for each experiment, as well as a broad overview of the performance of the run as a whole, by showing aggregate increases in PSMs at a chosen confidence threshold. (ZIP) [file pcbi.1007082.s001.zip › DART-ID_SCoPE-MS_Report/figures.html]

DART-ID Summary Figures


DART-ID Summary


Home
Experiment Summary

### Residual Summary

### PEP Update Summary

### Alignment Summaries

Previous


Next

...
