# Supplementary figures and images for: DART-ID increases single-cell proteome coverage (part 2 of 3)
Source: PLoS Comput Biol. 2019 Jul 1;15(7):e1007082. doi: 10.1371/journal.pcbi.1007082 (PMC6625733; doi:10.1371/journal.pcbi.1007082)

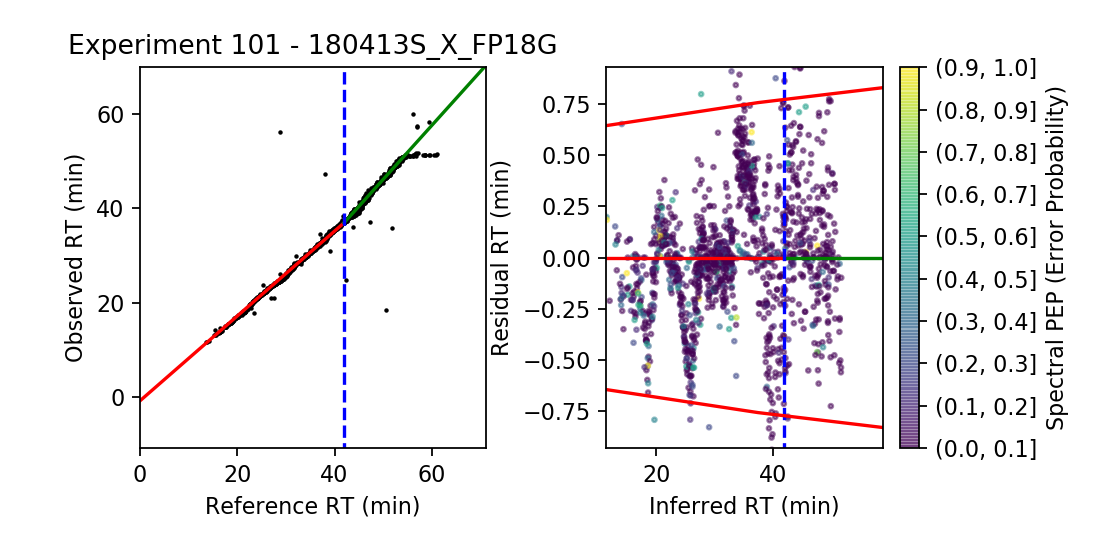

Supplement: S1 File — A optional HTML report generated by the dart_id Python script. The report gives a summary of the alignment for each experiment, as well as a broad overview of the performance of the run as a whole, by showing aggregate increases in PSMs at a chosen confidence threshold. (ZIP) [file pcbi.1007082.s001.zip › DART-ID_SCoPE-MS_Report/figures/alignment_101_180413S_X_FP18G.png]

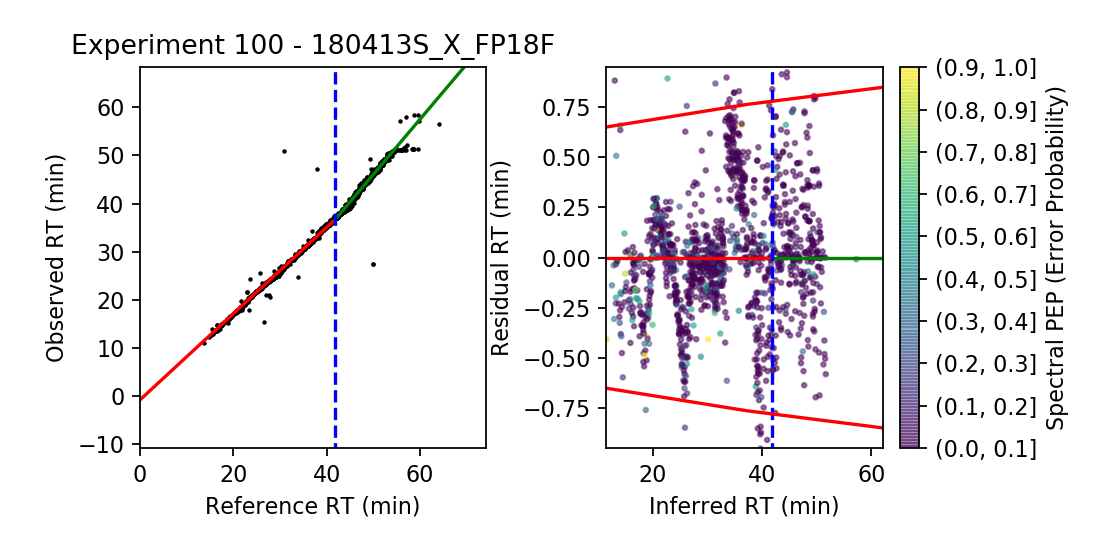

Supplement: S1 File — A optional HTML report generated by the dart_id Python script. The report gives a summary of the alignment for each experiment, as well as a broad overview of the performance of the run as a whole, by showing aggregate increases in PSMs at a chosen confidence threshold. (ZIP) [file pcbi.1007082.s001.zip › DART-ID_SCoPE-MS_Report/figures/alignment_100_180413S_X_FP18F.png]

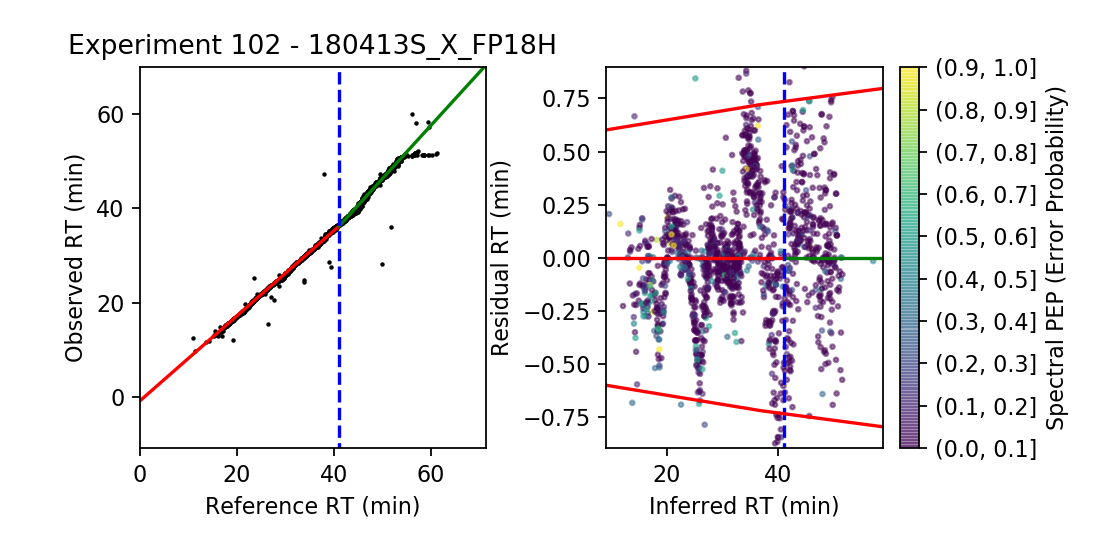

Supplement: S1 File — A optional HTML report generated by the dart_id Python script. The report gives a summary of the alignment for each experiment, as well as a broad overview of the performance of the run as a whole, by showing aggregate increases in PSMs at a chosen confidence threshold. (ZIP) [file pcbi.1007082.s001.zip › DART-ID_SCoPE-MS_Report/figures/alignment_102_180413S_X_FP18H.png]

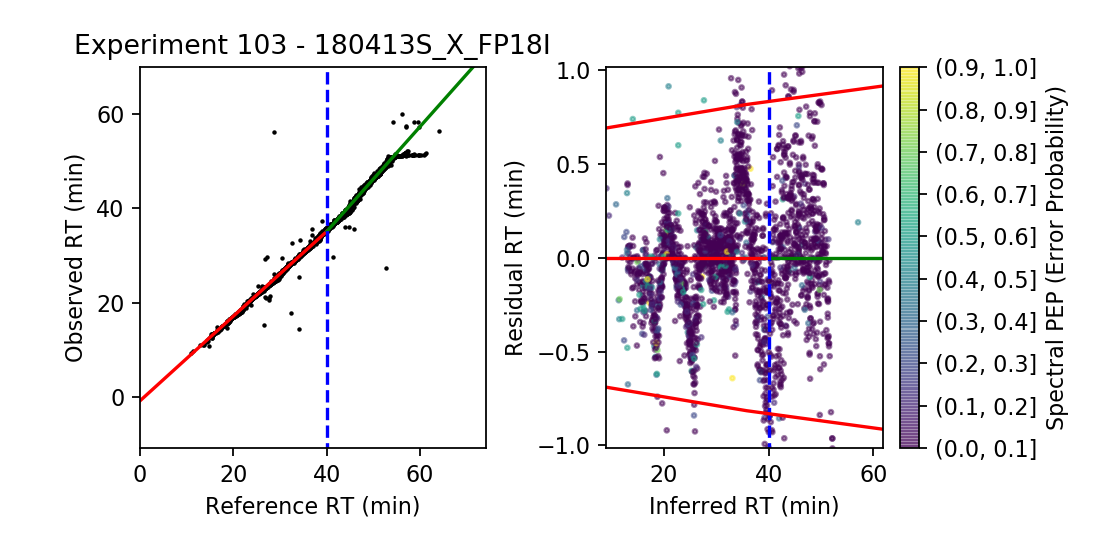

Supplement: S1 File — A optional HTML report generated by the dart_id Python script. The report gives a summary of the alignment for each experiment, as well as a broad overview of the performance of the run as a whole, by showing aggregate increases in PSMs at a chosen confidence threshold. (ZIP) [file pcbi.1007082.s001.zip › DART-ID_SCoPE-MS_Report/figures/alignment_103_180413S_X_FP18I.png]

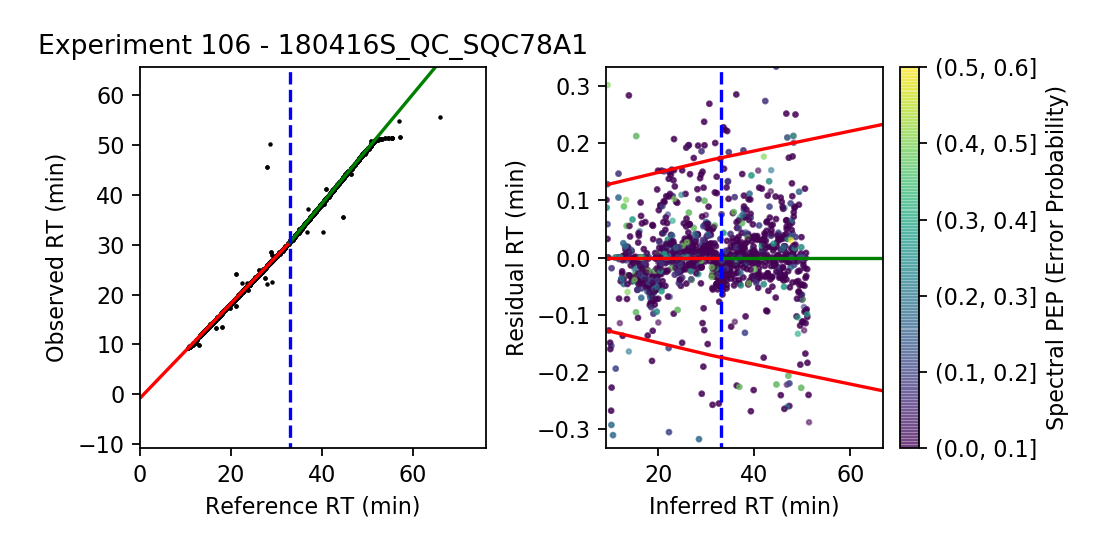

Supplement: S1 File — A optional HTML report generated by the dart_id Python script. The report gives a summary of the alignment for each experiment, as well as a broad overview of the performance of the run as a whole, by showing aggregate increases in PSMs at a chosen confidence threshold. (ZIP) [file pcbi.1007082.s001.zip › DART-ID_SCoPE-MS_Report/figures/alignment_106_180416S_QC_SQC78A1.png]

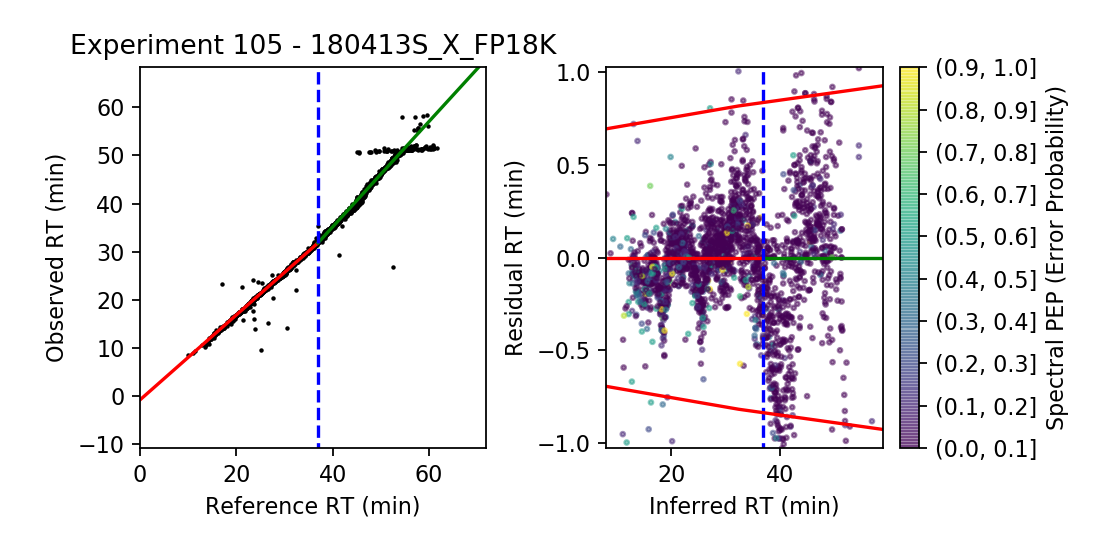

Supplement: S1 File — A optional HTML report generated by the dart_id Python script. The report gives a summary of the alignment for each experiment, as well as a broad overview of the performance of the run as a whole, by showing aggregate increases in PSMs at a chosen confidence threshold. (ZIP) [file pcbi.1007082.s001.zip › DART-ID_SCoPE-MS_Report/figures/alignment_105_180413S_X_FP18K.png]

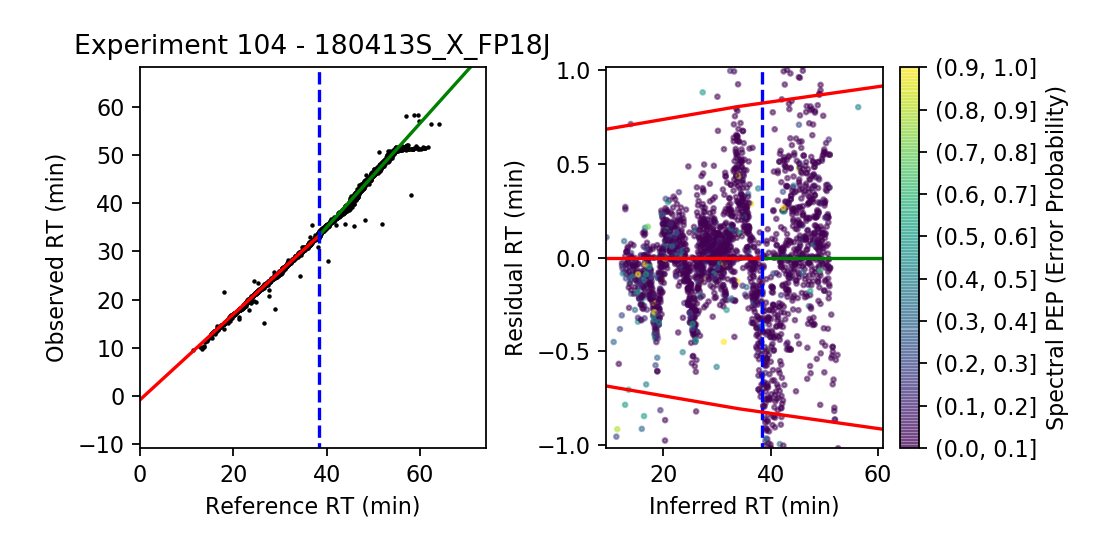

Supplement: S1 File — A optional HTML report generated by the dart_id Python script. The report gives a summary of the alignment for each experiment, as well as a broad overview of the performance of the run as a whole, by showing aggregate increases in PSMs at a chosen confidence threshold. (ZIP) [file pcbi.1007082.s001.zip › DART-ID_SCoPE-MS_Report/figures/alignment_104_180413S_X_FP18J.png]

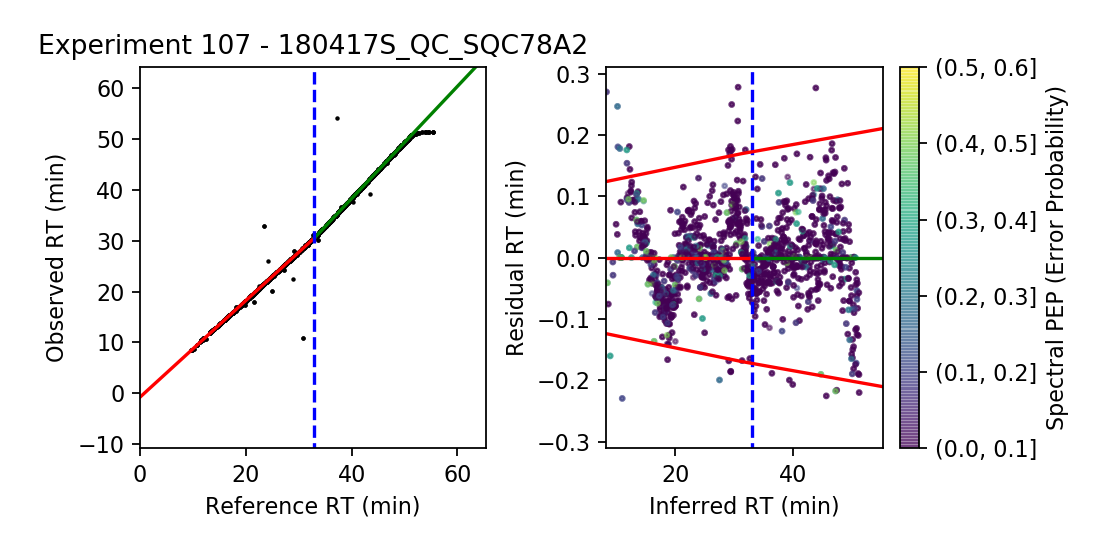

Supplement: S1 File — A optional HTML report generated by the dart_id Python script. The report gives a summary of the alignment for each experiment, as well as a broad overview of the performance of the run as a whole, by showing aggregate increases in PSMs at a chosen confidence threshold. (ZIP) [file pcbi.1007082.s001.zip › DART-ID_SCoPE-MS_Report/figures/alignment_107_180417S_QC_SQC78A2.png]

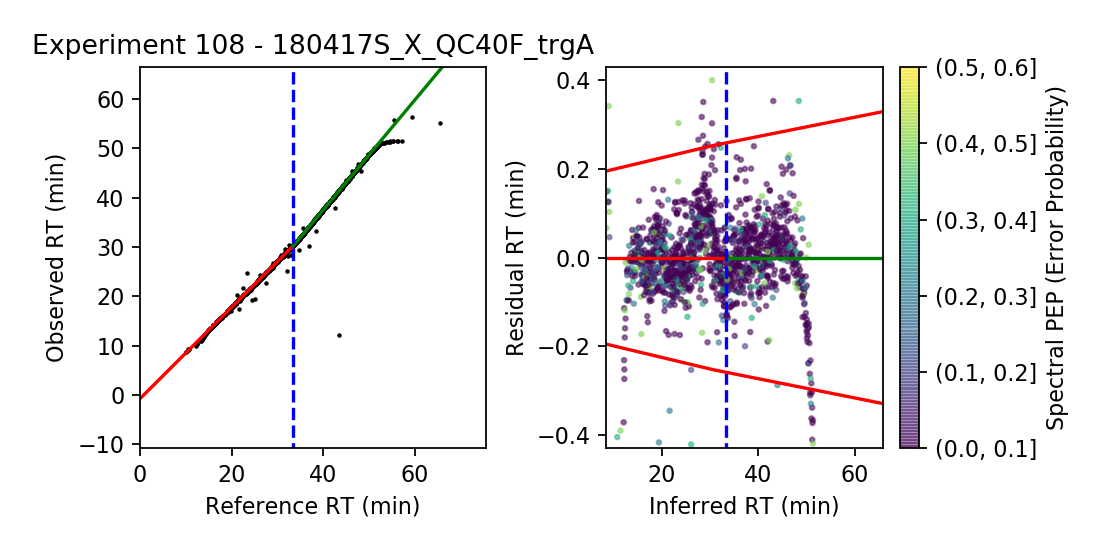

Supplement: S1 File — A optional HTML report generated by the dart_id Python script. The report gives a summary of the alignment for each experiment, as well as a broad overview of the performance of the run as a whole, by showing aggregate increases in PSMs at a chosen confidence threshold. (ZIP) [file pcbi.1007082.s001.zip › DART-ID_SCoPE-MS_Report/figures/alignment_108_180417S_X_QC40F_trgA.png]

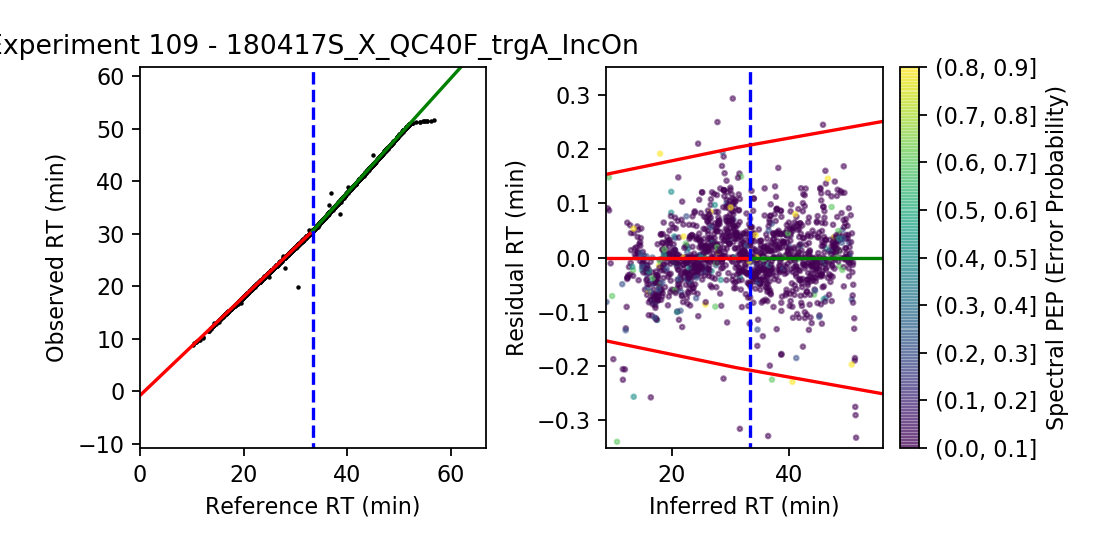

Supplement: S1 File — A optional HTML report generated by the dart_id Python script. The report gives a summary of the alignment for each experiment, as well as a broad overview of the performance of the run as a whole, by showing aggregate increases in PSMs at a chosen confidence threshold. (ZIP) [file pcbi.1007082.s001.zip › DART-ID_SCoPE-MS_Report/figures/alignment_109_180417S_X_QC40F_trgA_IncOn.png]

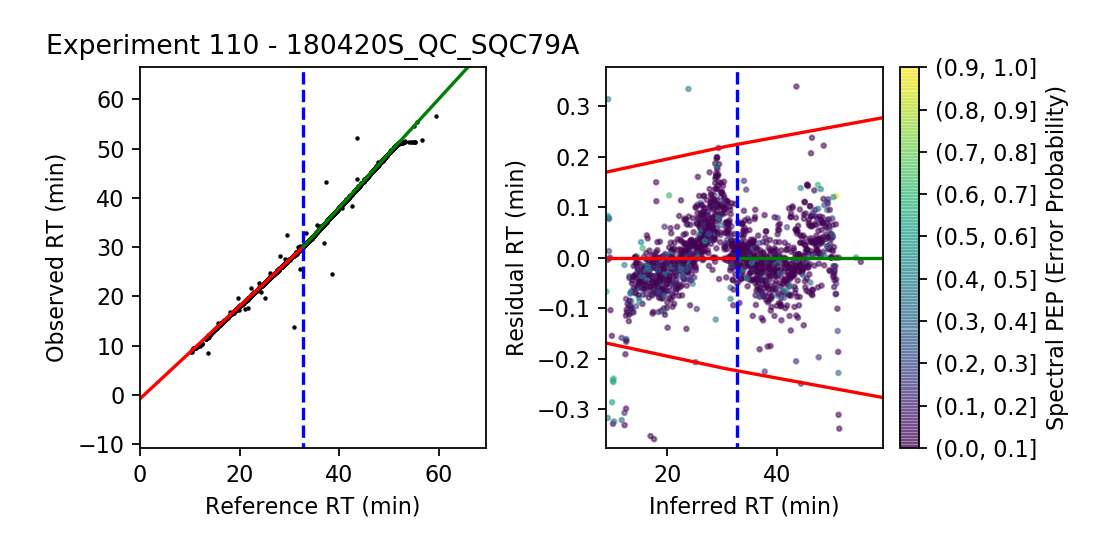

Supplement: S1 File — A optional HTML report generated by the dart_id Python script. The report gives a summary of the alignment for each experiment, as well as a broad overview of the performance of the run as a whole, by showing aggregate increases in PSMs at a chosen confidence threshold. (ZIP) [file pcbi.1007082.s001.zip › DART-ID_SCoPE-MS_Report/figures/alignment_110_180420S_QC_SQC79A.png]

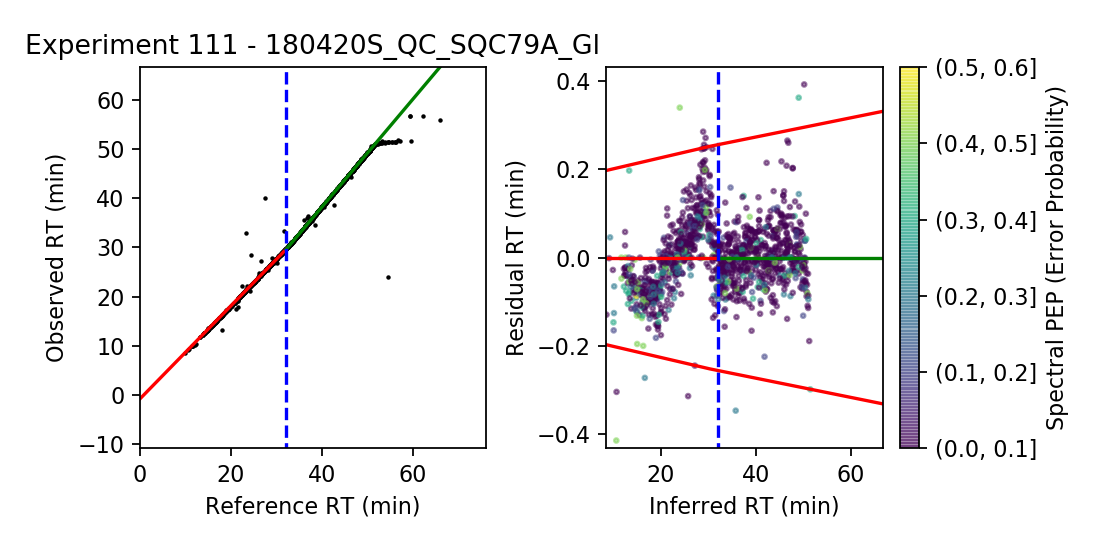

Supplement: S1 File — A optional HTML report generated by the dart_id Python script. The report gives a summary of the alignment for each experiment, as well as a broad overview of the performance of the run as a whole, by showing aggregate increases in PSMs at a chosen confidence threshold. (ZIP) [file pcbi.1007082.s001.zip › DART-ID_SCoPE-MS_Report/figures/alignment_111_180420S_QC_SQC79A_Gl.png]

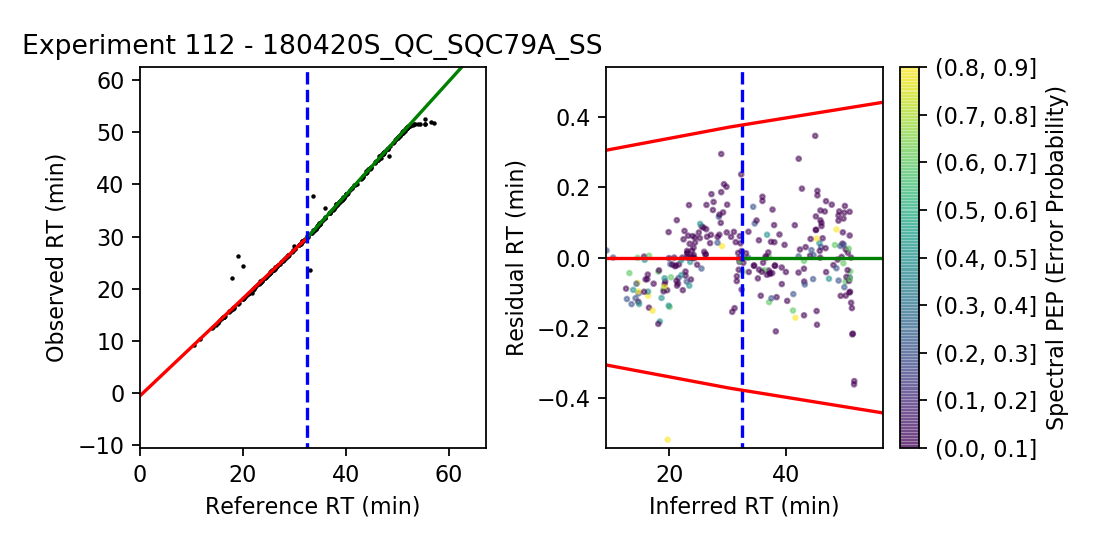

Supplement: S1 File — A optional HTML report generated by the dart_id Python script. The report gives a summary of the alignment for each experiment, as well as a broad overview of the performance of the run as a whole, by showing aggregate increases in PSMs at a chosen confidence threshold. (ZIP) [file pcbi.1007082.s001.zip › DART-ID_SCoPE-MS_Report/figures/alignment_112_180420S_QC_SQC79A_SS.png]

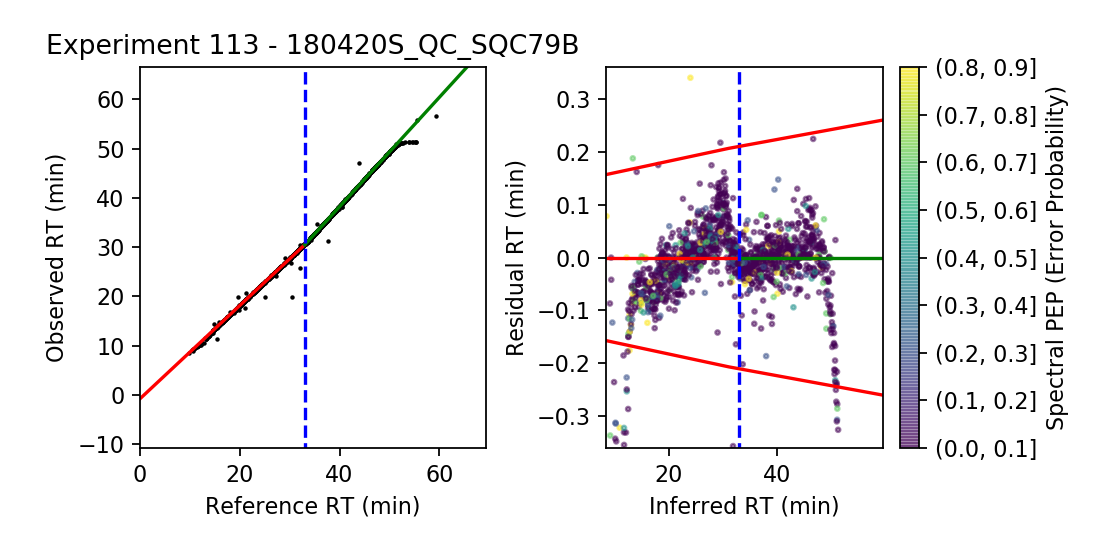

Supplement: S1 File — A optional HTML report generated by the dart_id Python script. The report gives a summary of the alignment for each experiment, as well as a broad overview of the performance of the run as a whole, by showing aggregate increases in PSMs at a chosen confidence threshold. (ZIP) [file pcbi.1007082.s001.zip › DART-ID_SCoPE-MS_Report/figures/alignment_113_180420S_QC_SQC79B.png]

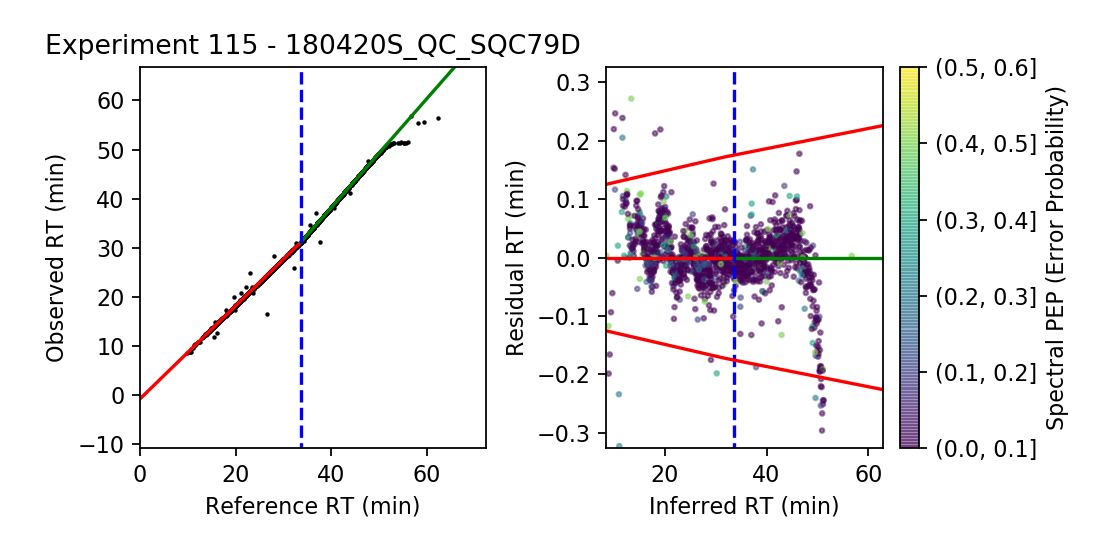

Supplement: S1 File — A optional HTML report generated by the dart_id Python script. The report gives a summary of the alignment for each experiment, as well as a broad overview of the performance of the run as a whole, by showing aggregate increases in PSMs at a chosen confidence threshold. (ZIP) [file pcbi.1007082.s001.zip › DART-ID_SCoPE-MS_Report/figures/alignment_115_180420S_QC_SQC79D.png]

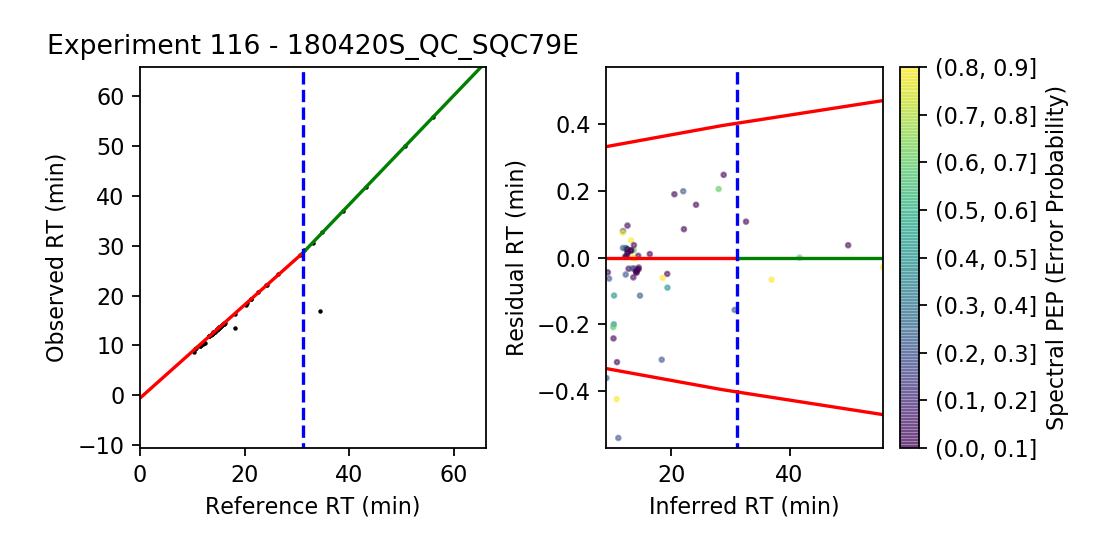

Supplement: S1 File — A optional HTML report generated by the dart_id Python script. The report gives a summary of the alignment for each experiment, as well as a broad overview of the performance of the run as a whole, by showing aggregate increases in PSMs at a chosen confidence threshold. (ZIP) [file pcbi.1007082.s001.zip › DART-ID_SCoPE-MS_Report/figures/alignment_116_180420S_QC_SQC79E.png]

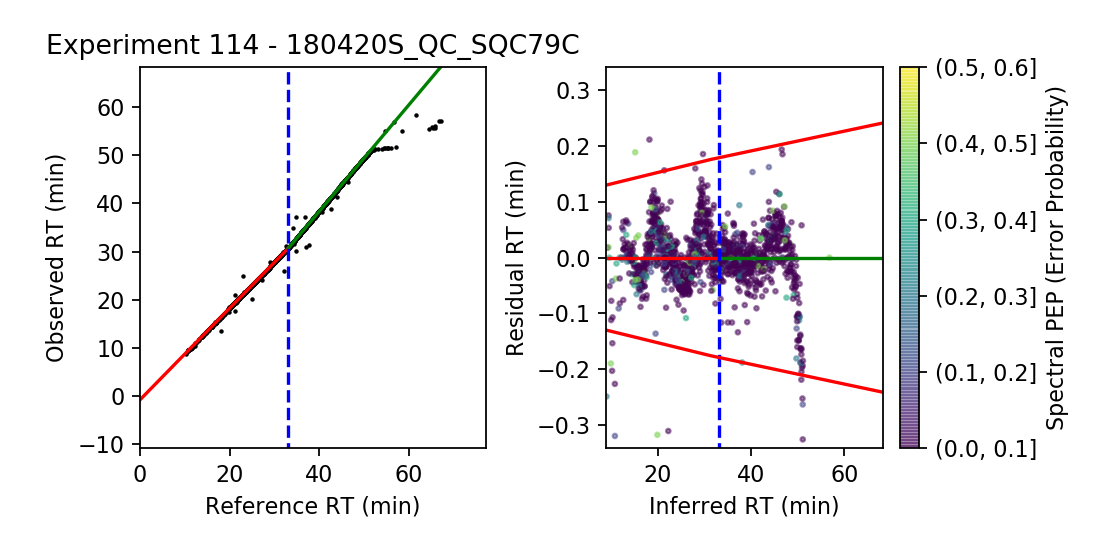

Supplement: S1 File — A optional HTML report generated by the dart_id Python script. The report gives a summary of the alignment for each experiment, as well as a broad overview of the performance of the run as a whole, by showing aggregate increases in PSMs at a chosen confidence threshold. (ZIP) [file pcbi.1007082.s001.zip › DART-ID_SCoPE-MS_Report/figures/alignment_114_180420S_QC_SQC79C.png]

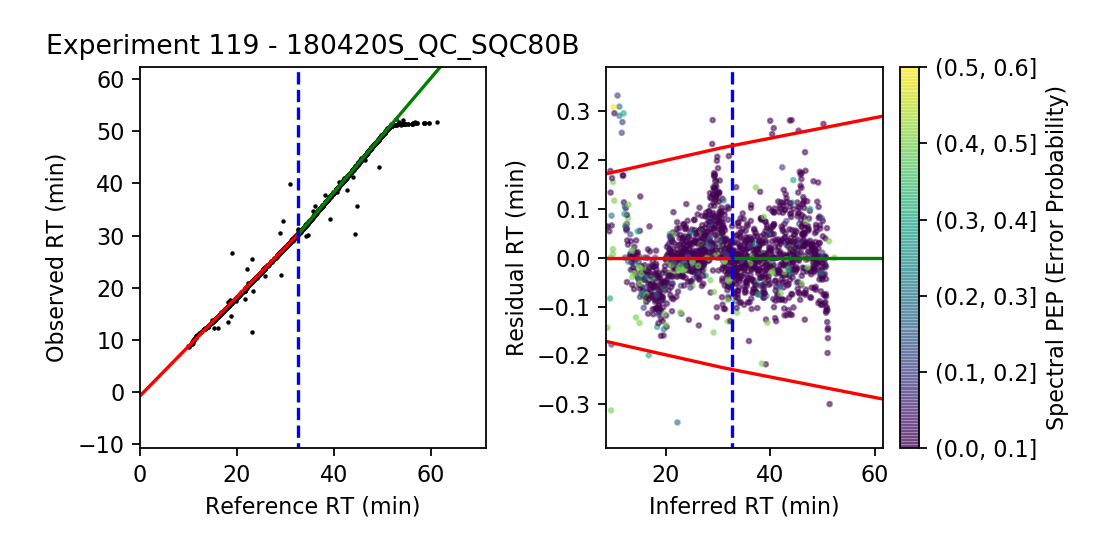

Supplement: S1 File — A optional HTML report generated by the dart_id Python script. The report gives a summary of the alignment for each experiment, as well as a broad overview of the performance of the run as a whole, by showing aggregate increases in PSMs at a chosen confidence threshold. (ZIP) [file pcbi.1007082.s001.zip › DART-ID_SCoPE-MS_Report/figures/alignment_119_180420S_QC_SQC80B.png]

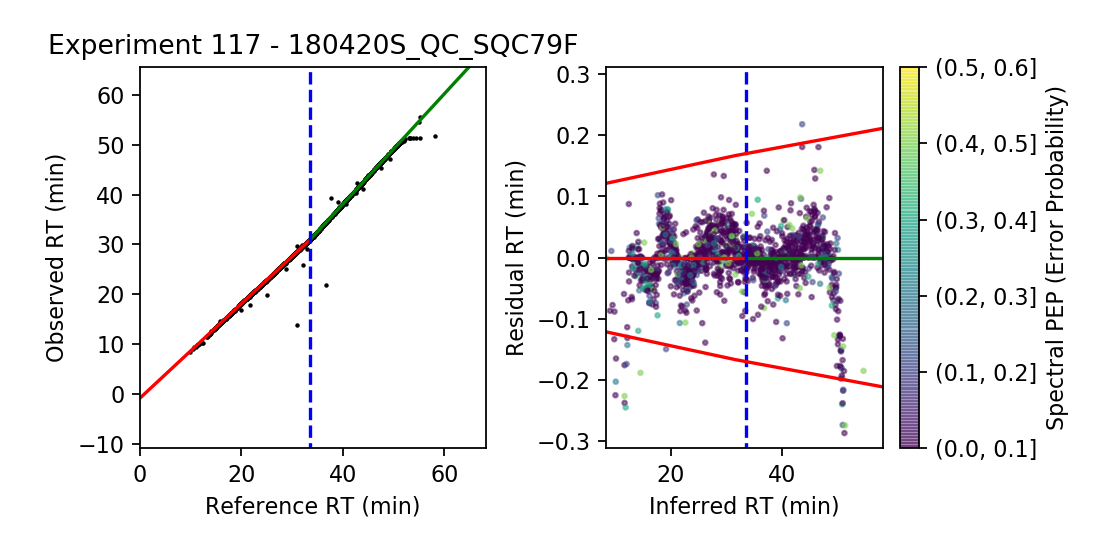

Supplement: S1 File — A optional HTML report generated by the dart_id Python script. The report gives a summary of the alignment for each experiment, as well as a broad overview of the performance of the run as a whole, by showing aggregate increases in PSMs at a chosen confidence threshold. (ZIP) [file pcbi.1007082.s001.zip › DART-ID_SCoPE-MS_Report/figures/alignment_117_180420S_QC_SQC79F.png]

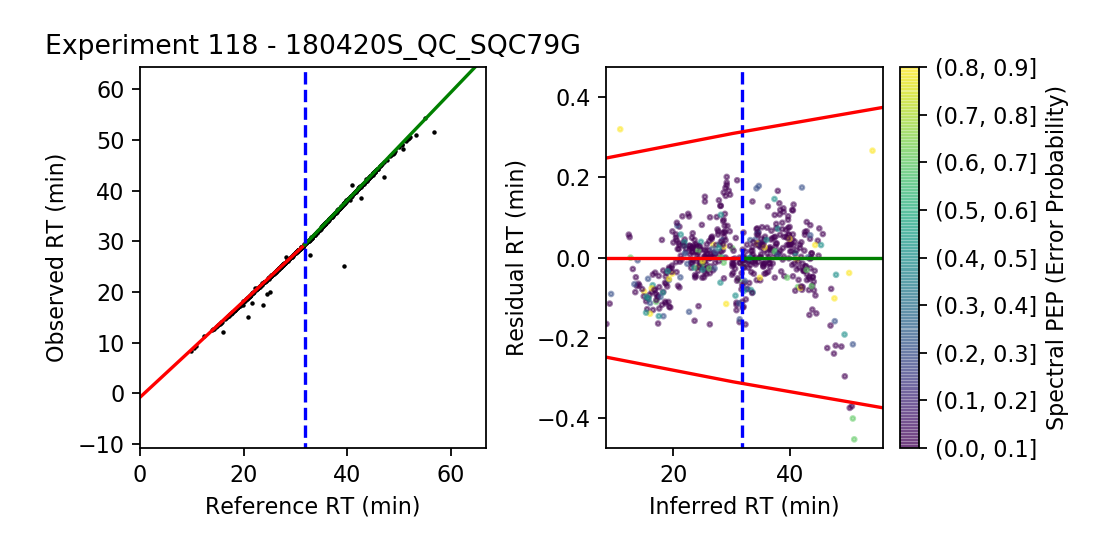

Supplement: S1 File — A optional HTML report generated by the dart_id Python script. The report gives a summary of the alignment for each experiment, as well as a broad overview of the performance of the run as a whole, by showing aggregate increases in PSMs at a chosen confidence threshold. (ZIP) [file pcbi.1007082.s001.zip › DART-ID_SCoPE-MS_Report/figures/alignment_118_180420S_QC_SQC79G.png]

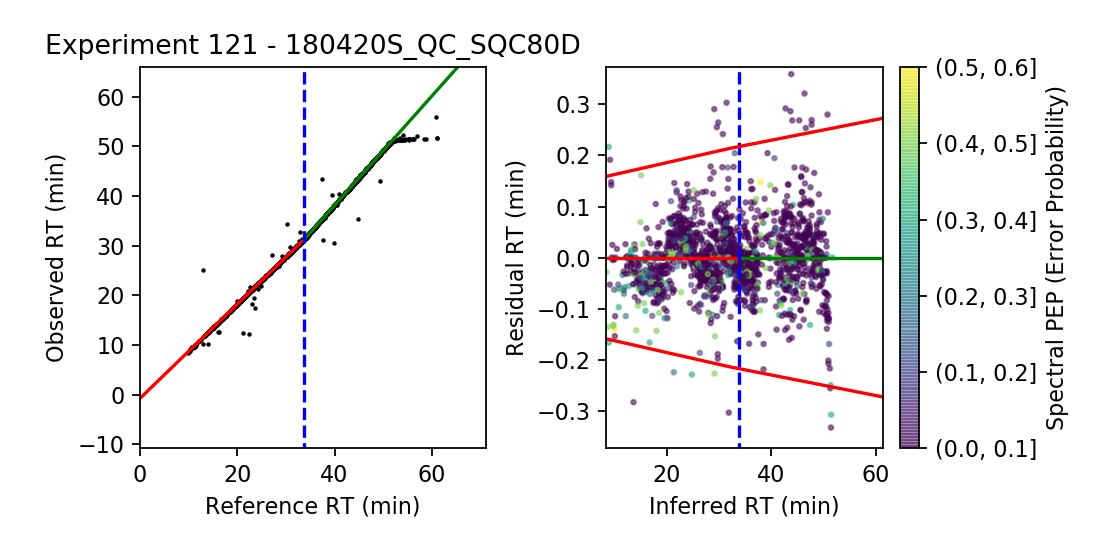

Supplement: S1 File — A optional HTML report generated by the dart_id Python script. The report gives a summary of the alignment for each experiment, as well as a broad overview of the performance of the run as a whole, by showing aggregate increases in PSMs at a chosen confidence threshold. (ZIP) [file pcbi.1007082.s001.zip › DART-ID_SCoPE-MS_Report/figures/alignment_121_180420S_QC_SQC80D.png]

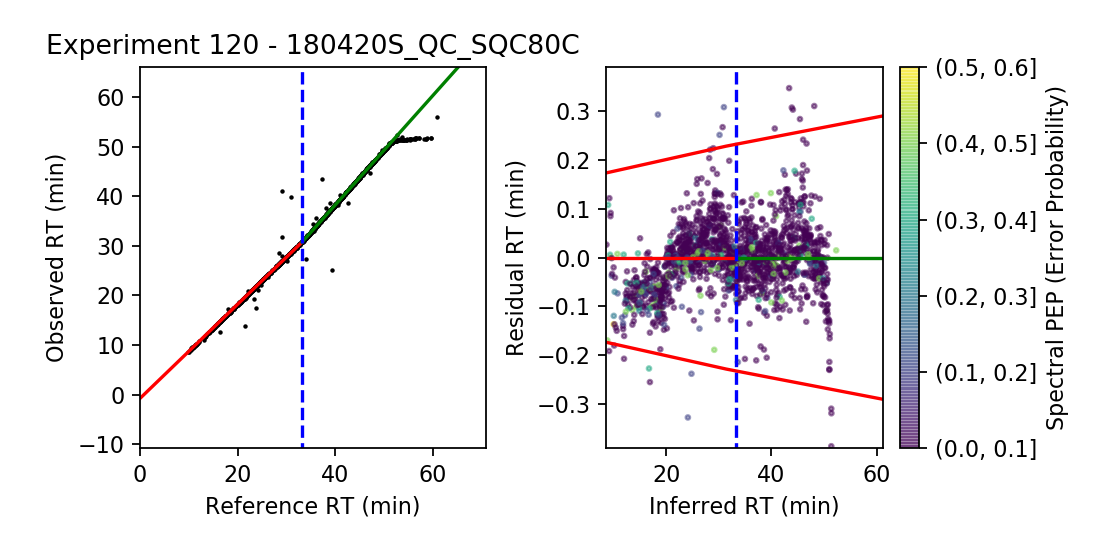

Supplement: S1 File — A optional HTML report generated by the dart_id Python script. The report gives a summary of the alignment for each experiment, as well as a broad overview of the performance of the run as a whole, by showing aggregate increases in PSMs at a chosen confidence threshold. (ZIP) [file pcbi.1007082.s001.zip › DART-ID_SCoPE-MS_Report/figures/alignment_120_180420S_QC_SQC80C.png]

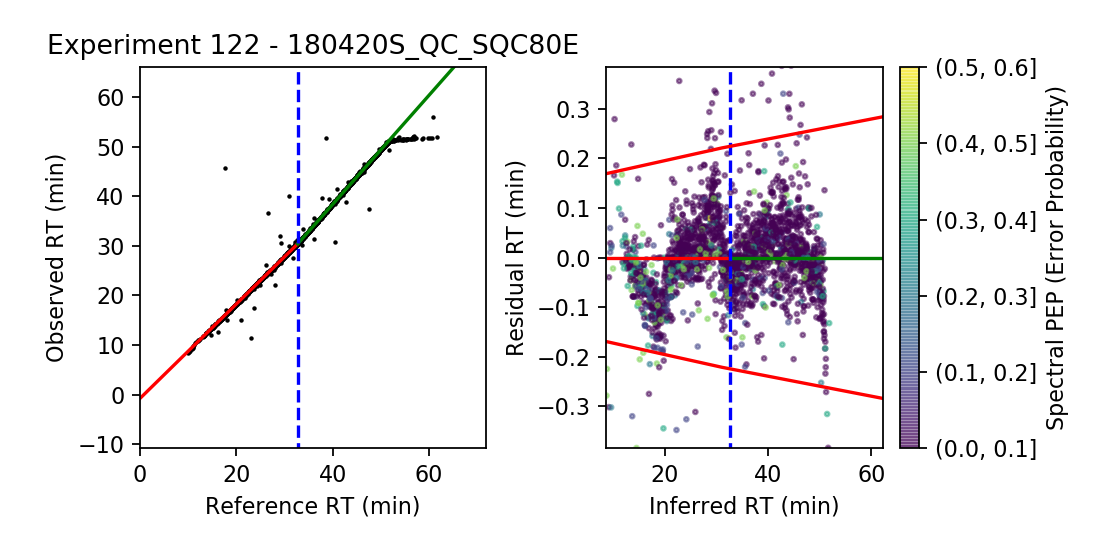

Supplement: S1 File — A optional HTML report generated by the dart_id Python script. The report gives a summary of the alignment for each experiment, as well as a broad overview of the performance of the run as a whole, by showing aggregate increases in PSMs at a chosen confidence threshold. (ZIP) [file pcbi.1007082.s001.zip › DART-ID_SCoPE-MS_Report/figures/alignment_122_180420S_QC_SQC80E.png]

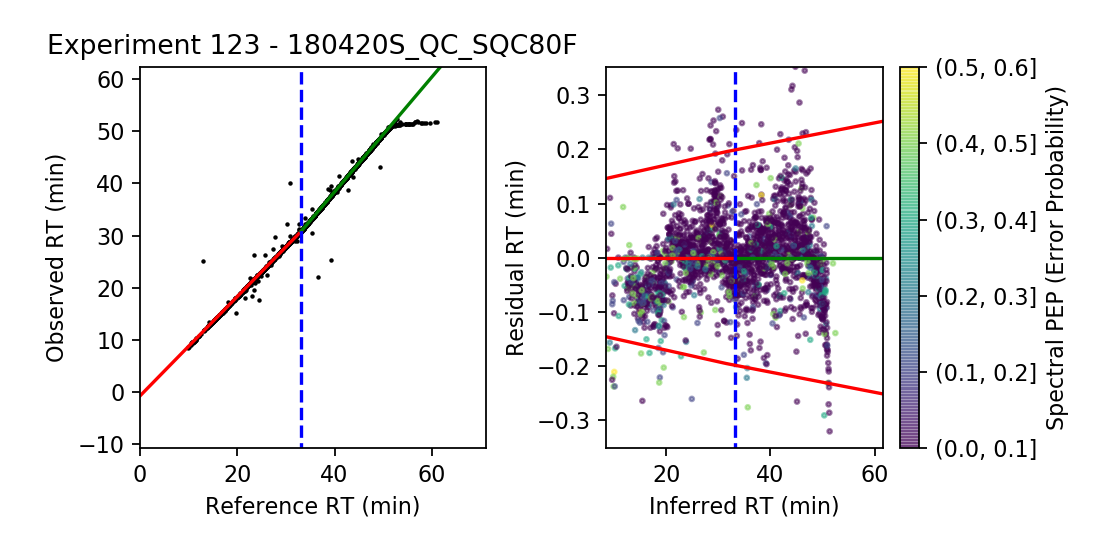

Supplement: S1 File — A optional HTML report generated by the dart_id Python script. The report gives a summary of the alignment for each experiment, as well as a broad overview of the performance of the run as a whole, by showing aggregate increases in PSMs at a chosen confidence threshold. (ZIP) [file pcbi.1007082.s001.zip › DART-ID_SCoPE-MS_Report/figures/alignment_123_180420S_QC_SQC80F.png]

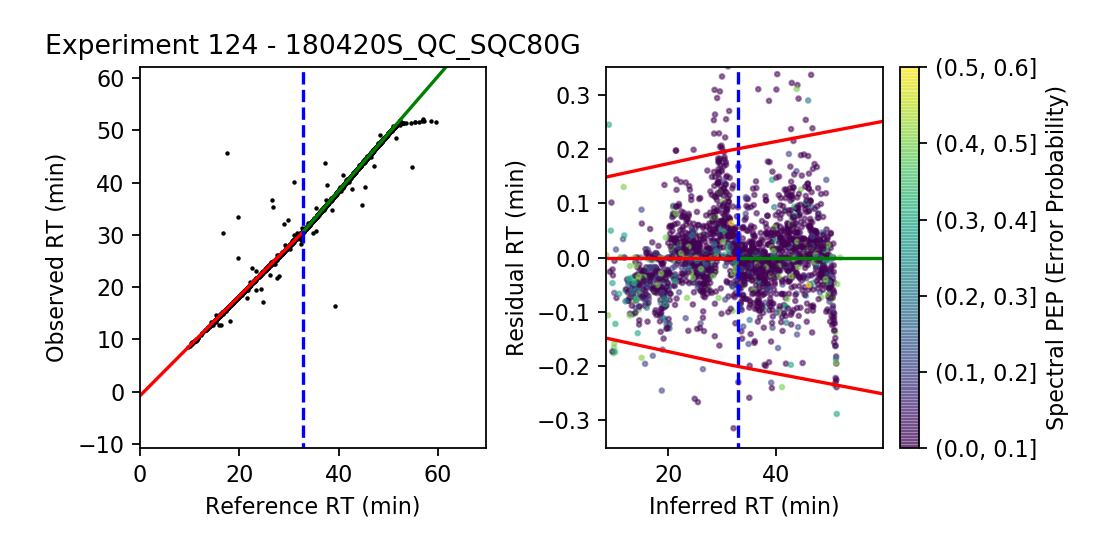

Supplement: S1 File — A optional HTML report generated by the dart_id Python script. The report gives a summary of the alignment for each experiment, as well as a broad overview of the performance of the run as a whole, by showing aggregate increases in PSMs at a chosen confidence threshold. (ZIP) [file pcbi.1007082.s001.zip › DART-ID_SCoPE-MS_Report/figures/alignment_124_180420S_QC_SQC80G.png]

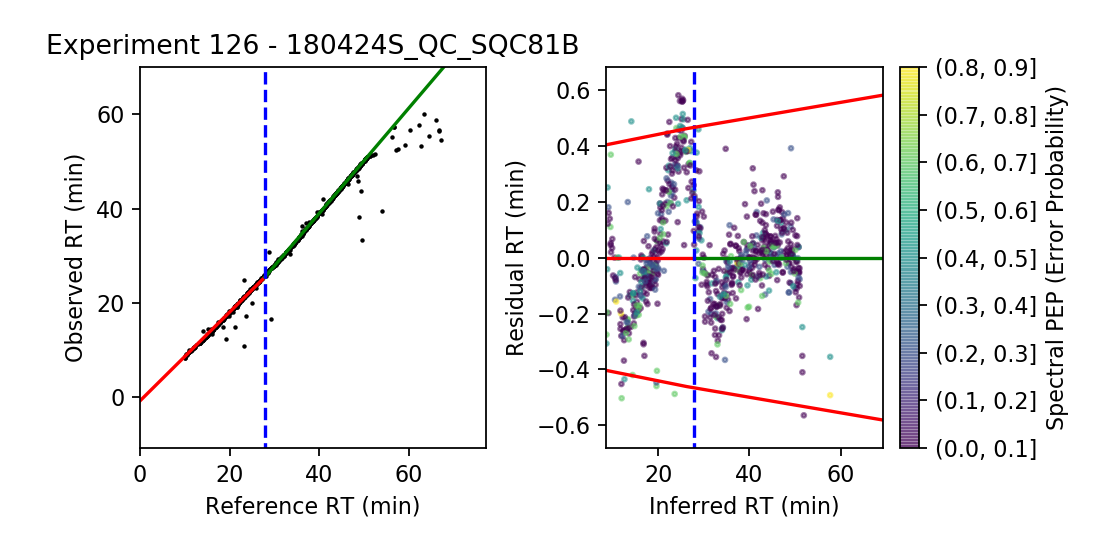

Supplement: S1 File — A optional HTML report generated by the dart_id Python script. The report gives a summary of the alignment for each experiment, as well as a broad overview of the performance of the run as a whole, by showing aggregate increases in PSMs at a chosen confidence threshold. (ZIP) [file pcbi.1007082.s001.zip › DART-ID_SCoPE-MS_Report/figures/alignment_126_180424S_QC_SQC81B.png]

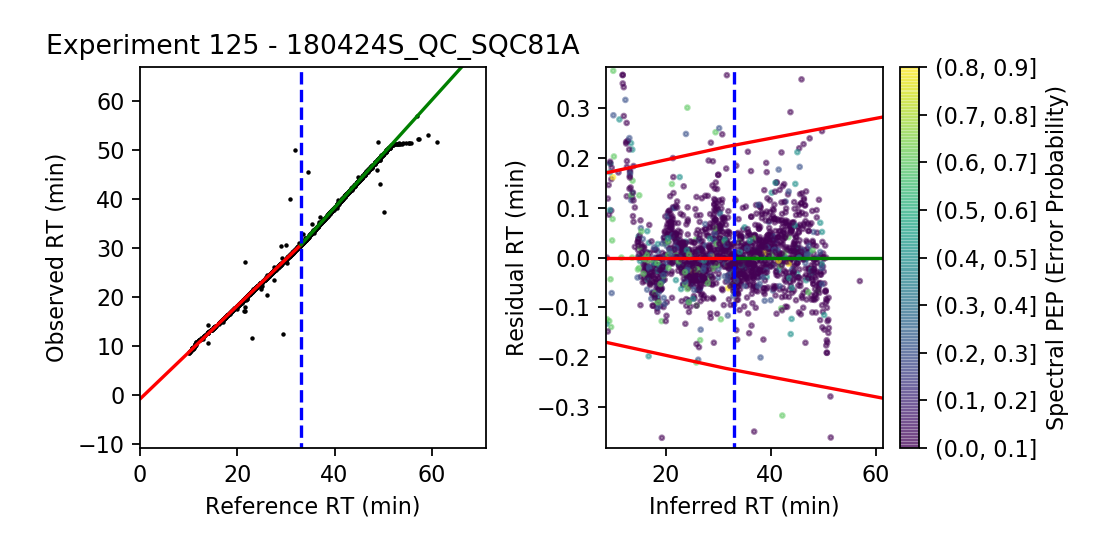

Supplement: S1 File — A optional HTML report generated by the dart_id Python script. The report gives a summary of the alignment for each experiment, as well as a broad overview of the performance of the run as a whole, by showing aggregate increases in PSMs at a chosen confidence threshold. (ZIP) [file pcbi.1007082.s001.zip › DART-ID_SCoPE-MS_Report/figures/alignment_125_180424S_QC_SQC81A.png]

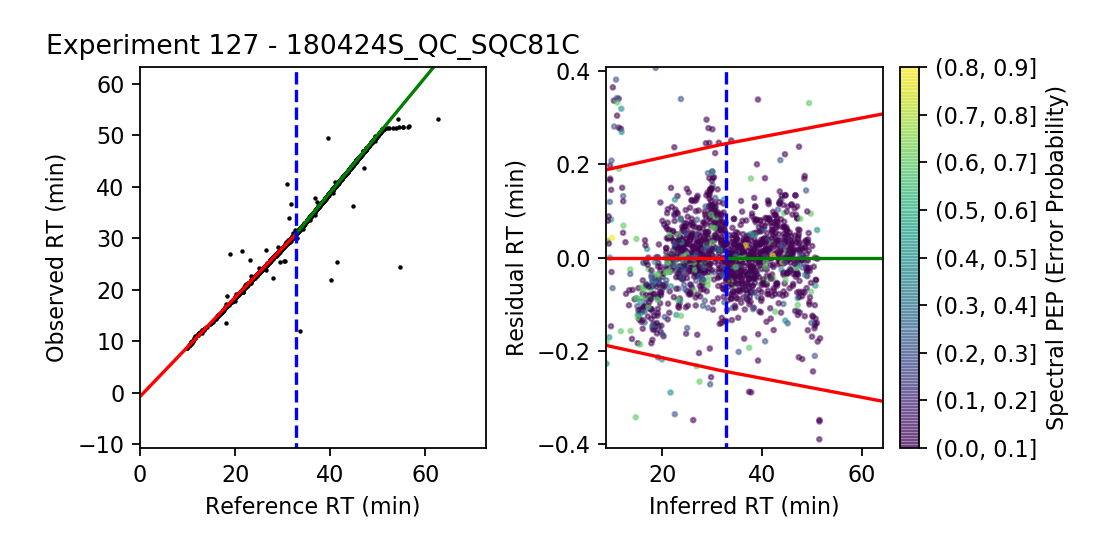

Supplement: S1 File — A optional HTML report generated by the dart_id Python script. The report gives a summary of the alignment for each experiment, as well as a broad overview of the performance of the run as a whole, by showing aggregate increases in PSMs at a chosen confidence threshold. (ZIP) [file pcbi.1007082.s001.zip › DART-ID_SCoPE-MS_Report/figures/alignment_127_180424S_QC_SQC81C.png]

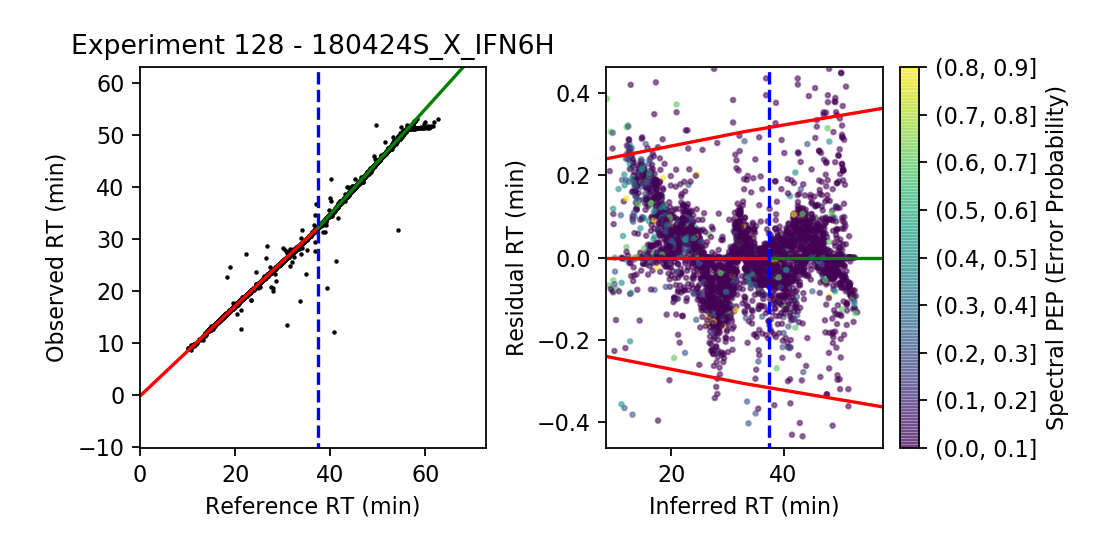

Supplement: S1 File — A optional HTML report generated by the dart_id Python script. The report gives a summary of the alignment for each experiment, as well as a broad overview of the performance of the run as a whole, by showing aggregate increases in PSMs at a chosen confidence threshold. (ZIP) [file pcbi.1007082.s001.zip › DART-ID_SCoPE-MS_Report/figures/alignment_128_180424S_X_IFN6H.png]

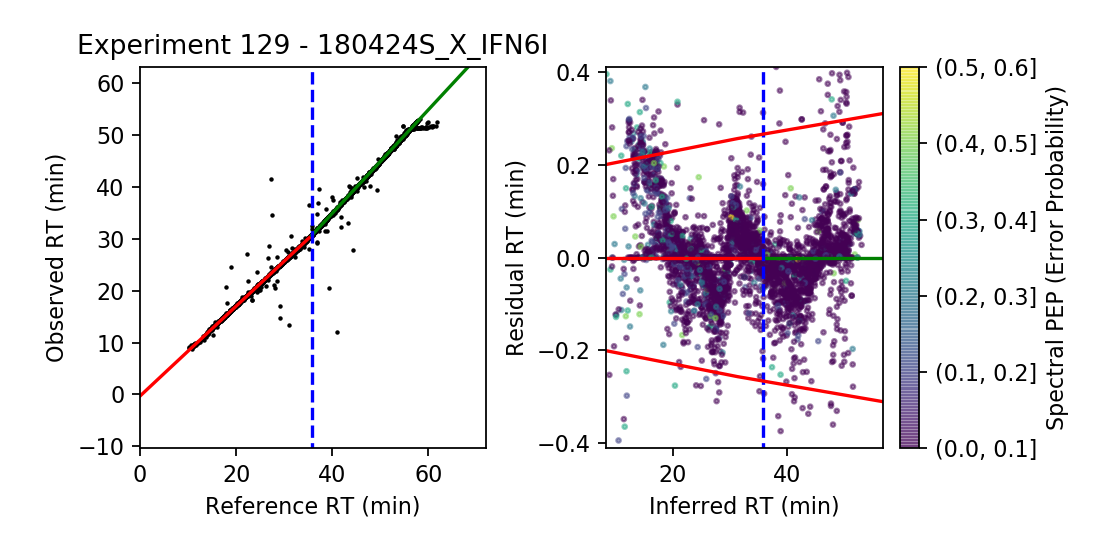

Supplement: S1 File — A optional HTML report generated by the dart_id Python script. The report gives a summary of the alignment for each experiment, as well as a broad overview of the performance of the run as a whole, by showing aggregate increases in PSMs at a chosen confidence threshold. (ZIP) [file pcbi.1007082.s001.zip › DART-ID_SCoPE-MS_Report/figures/alignment_129_180424S_X_IFN6I.png]

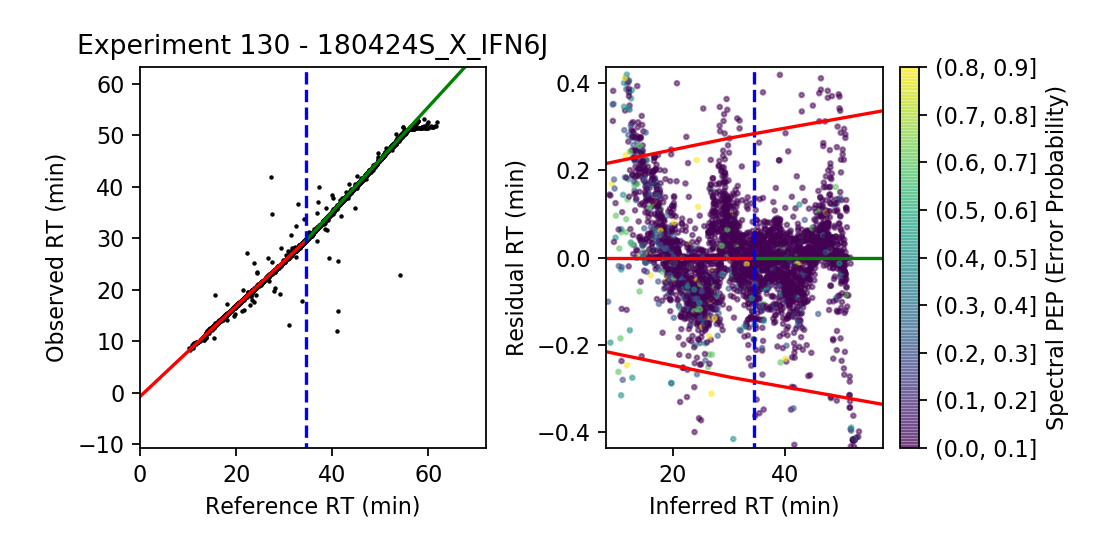

Supplement: S1 File — A optional HTML report generated by the dart_id Python script. The report gives a summary of the alignment for each experiment, as well as a broad overview of the performance of the run as a whole, by showing aggregate increases in PSMs at a chosen confidence threshold. (ZIP) [file pcbi.1007082.s001.zip › DART-ID_SCoPE-MS_Report/figures/alignment_130_180424S_X_IFN6J.png]

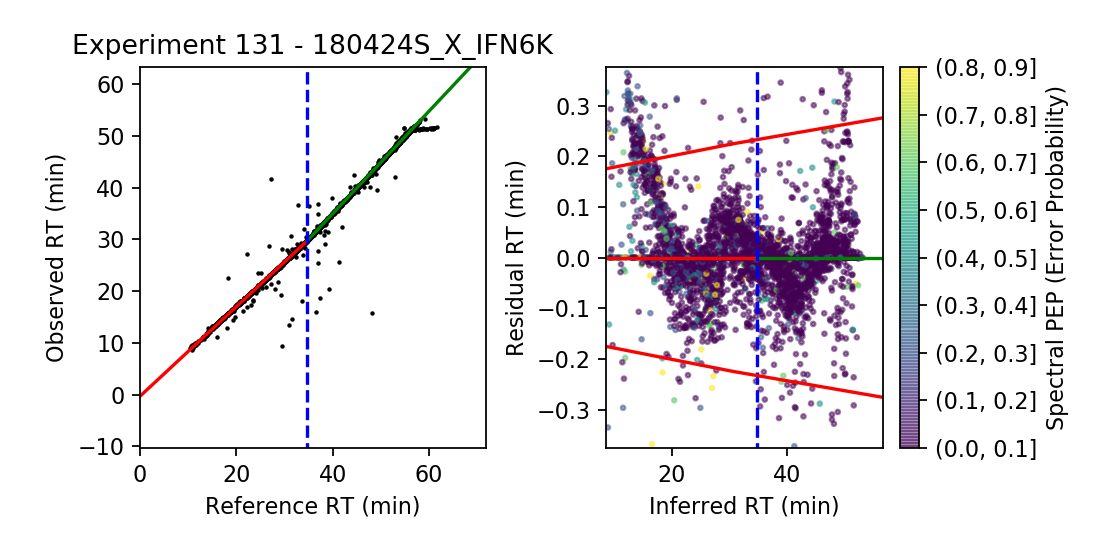

Supplement: S1 File — A optional HTML report generated by the dart_id Python script. The report gives a summary of the alignment for each experiment, as well as a broad overview of the performance of the run as a whole, by showing aggregate increases in PSMs at a chosen confidence threshold. (ZIP) [file pcbi.1007082.s001.zip › DART-ID_SCoPE-MS_Report/figures/alignment_131_180424S_X_IFN6K.png]

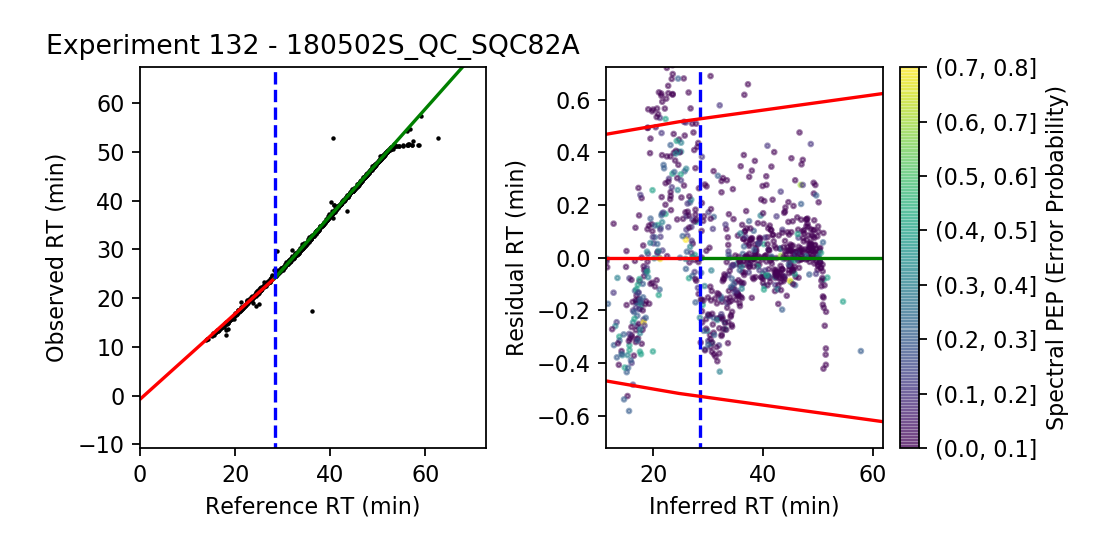

Supplement: S1 File — A optional HTML report generated by the dart_id Python script. The report gives a summary of the alignment for each experiment, as well as a broad overview of the performance of the run as a whole, by showing aggregate increases in PSMs at a chosen confidence threshold. (ZIP) [file pcbi.1007082.s001.zip › DART-ID_SCoPE-MS_Report/figures/alignment_132_180502S_QC_SQC82A.png]

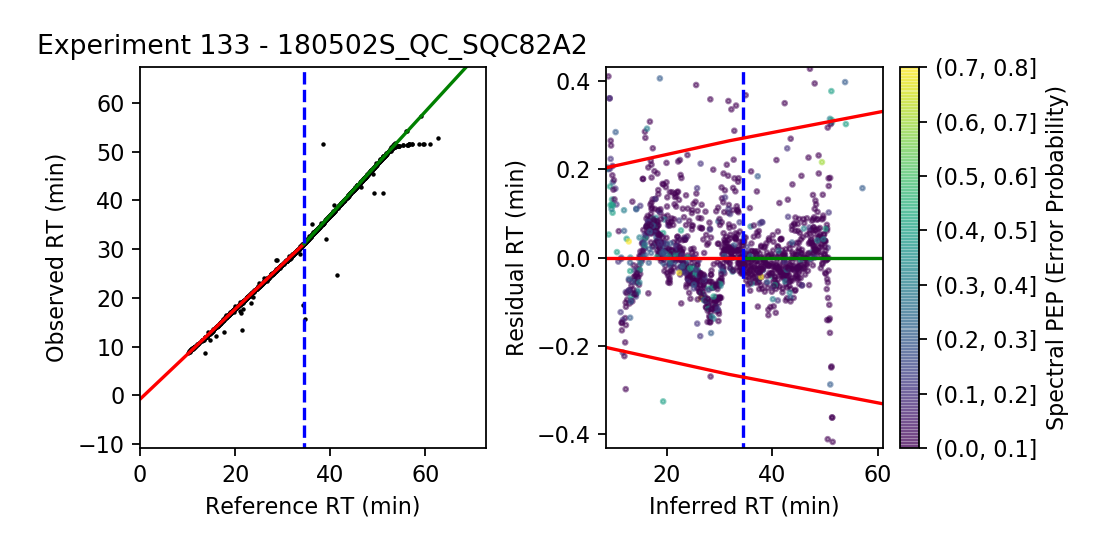

Supplement: S1 File — A optional HTML report generated by the dart_id Python script. The report gives a summary of the alignment for each experiment, as well as a broad overview of the performance of the run as a whole, by showing aggregate increases in PSMs at a chosen confidence threshold. (ZIP) [file pcbi.1007082.s001.zip › DART-ID_SCoPE-MS_Report/figures/alignment_133_180502S_QC_SQC82A2.png]

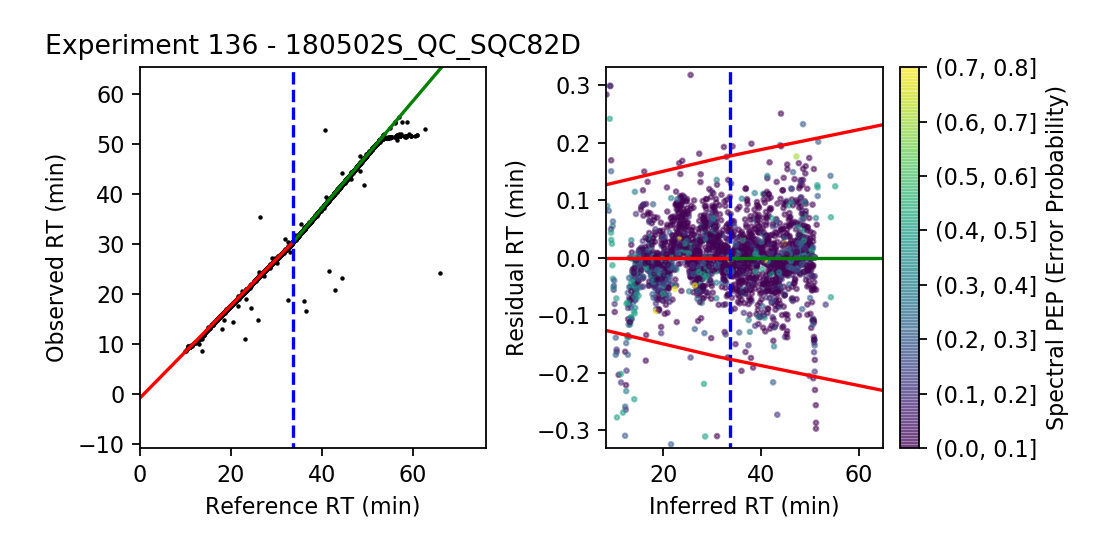

Supplement: S1 File — A optional HTML report generated by the dart_id Python script. The report gives a summary of the alignment for each experiment, as well as a broad overview of the performance of the run as a whole, by showing aggregate increases in PSMs at a chosen confidence threshold. (ZIP) [file pcbi.1007082.s001.zip › DART-ID_SCoPE-MS_Report/figures/alignment_136_180502S_QC_SQC82D.png]

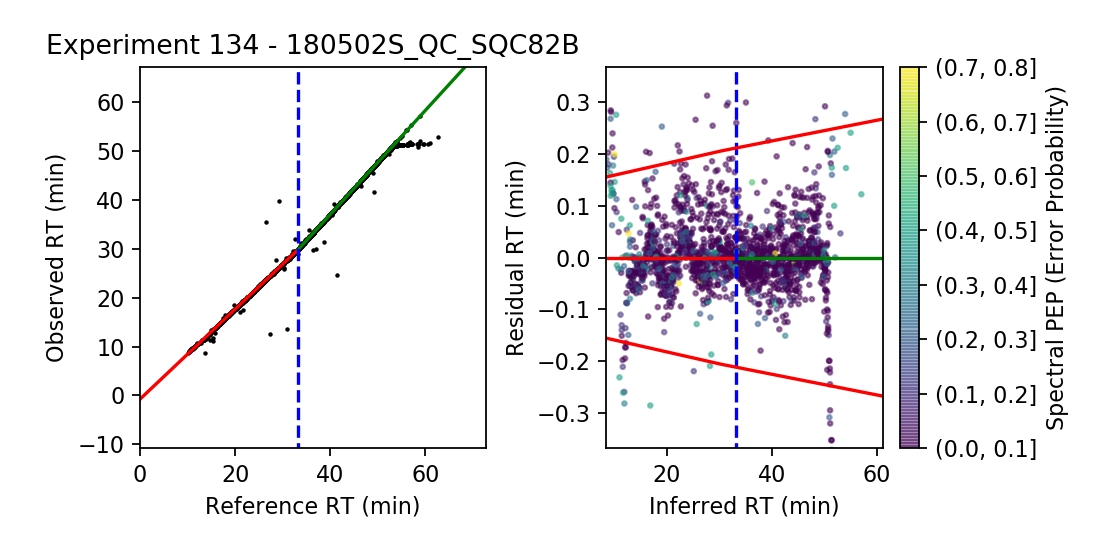

Supplement: S1 File — A optional HTML report generated by the dart_id Python script. The report gives a summary of the alignment for each experiment, as well as a broad overview of the performance of the run as a whole, by showing aggregate increases in PSMs at a chosen confidence threshold. (ZIP) [file pcbi.1007082.s001.zip › DART-ID_SCoPE-MS_Report/figures/alignment_134_180502S_QC_SQC82B.png]

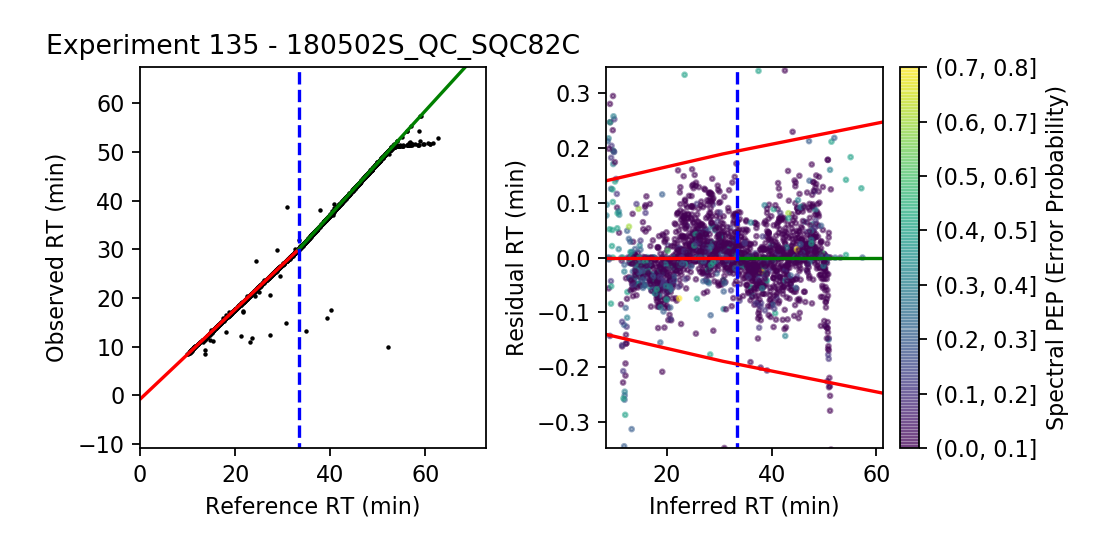

Supplement: S1 File — A optional HTML report generated by the dart_id Python script. The report gives a summary of the alignment for each experiment, as well as a broad overview of the performance of the run as a whole, by showing aggregate increases in PSMs at a chosen confidence threshold. (ZIP) [file pcbi.1007082.s001.zip › DART-ID_SCoPE-MS_Report/figures/alignment_135_180502S_QC_SQC82C.png]

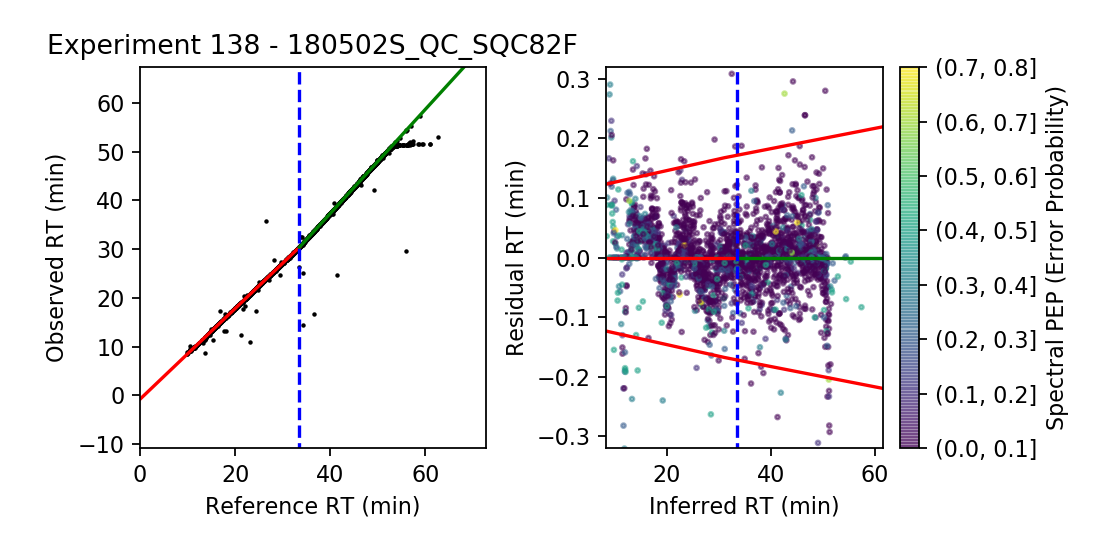

Supplement: S1 File — A optional HTML report generated by the dart_id Python script. The report gives a summary of the alignment for each experiment, as well as a broad overview of the performance of the run as a whole, by showing aggregate increases in PSMs at a chosen confidence threshold. (ZIP) [file pcbi.1007082.s001.zip › DART-ID_SCoPE-MS_Report/figures/alignment_138_180502S_QC_SQC82F.png]

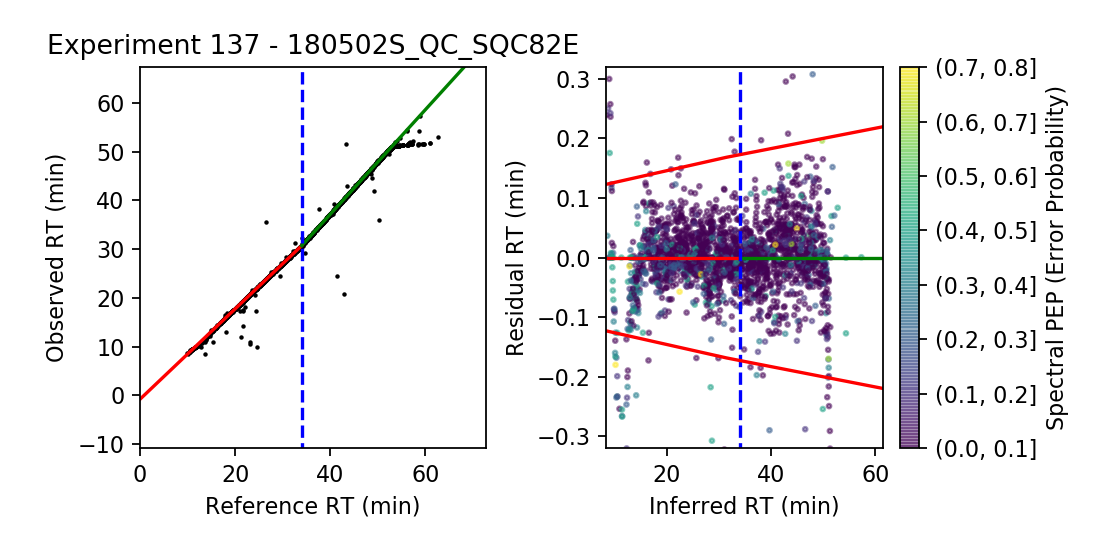

Supplement: S1 File — A optional HTML report generated by the dart_id Python script. The report gives a summary of the alignment for each experiment, as well as a broad overview of the performance of the run as a whole, by showing aggregate increases in PSMs at a chosen confidence threshold. (ZIP) [file pcbi.1007082.s001.zip › DART-ID_SCoPE-MS_Report/figures/alignment_137_180502S_QC_SQC82E.png]

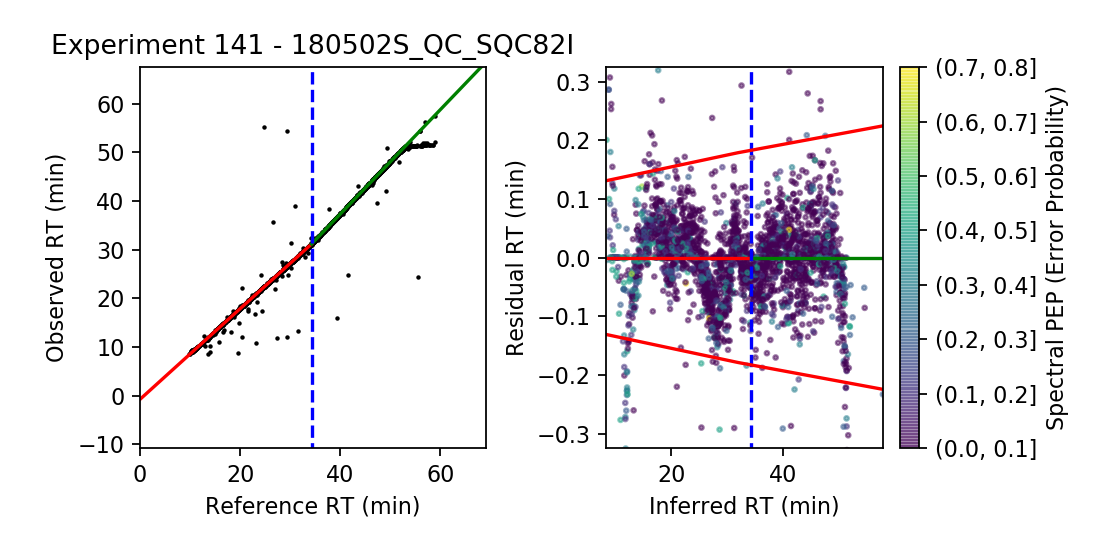

Supplement: S1 File — A optional HTML report generated by the dart_id Python script. The report gives a summary of the alignment for each experiment, as well as a broad overview of the performance of the run as a whole, by showing aggregate increases in PSMs at a chosen confidence threshold. (ZIP) [file pcbi.1007082.s001.zip › DART-ID_SCoPE-MS_Report/figures/alignment_141_180502S_QC_SQC82I.png]

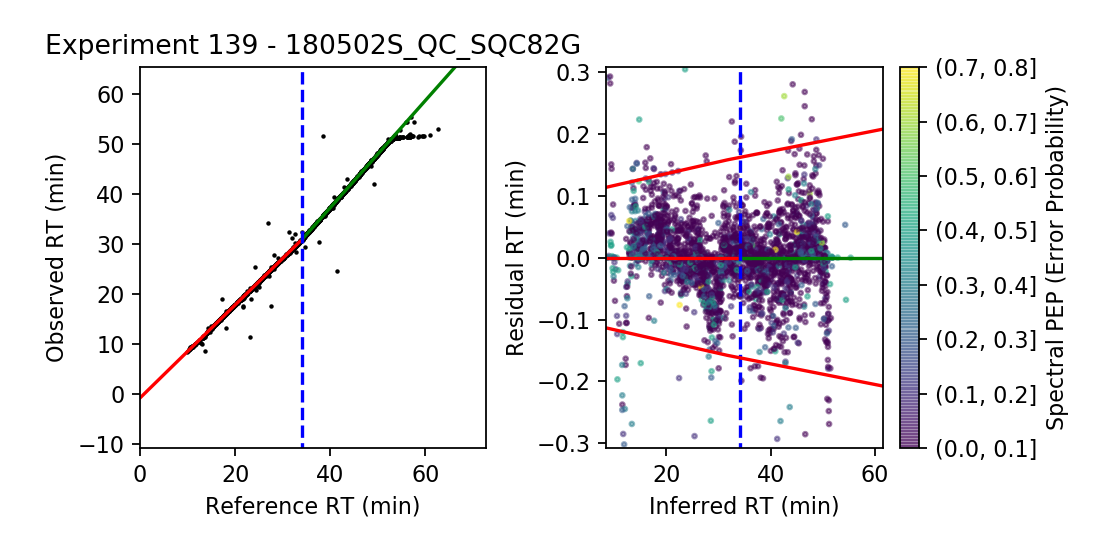

Supplement: S1 File — A optional HTML report generated by the dart_id Python script. The report gives a summary of the alignment for each experiment, as well as a broad overview of the performance of the run as a whole, by showing aggregate increases in PSMs at a chosen confidence threshold. (ZIP) [file pcbi.1007082.s001.zip › DART-ID_SCoPE-MS_Report/figures/alignment_139_180502S_QC_SQC82G.png]

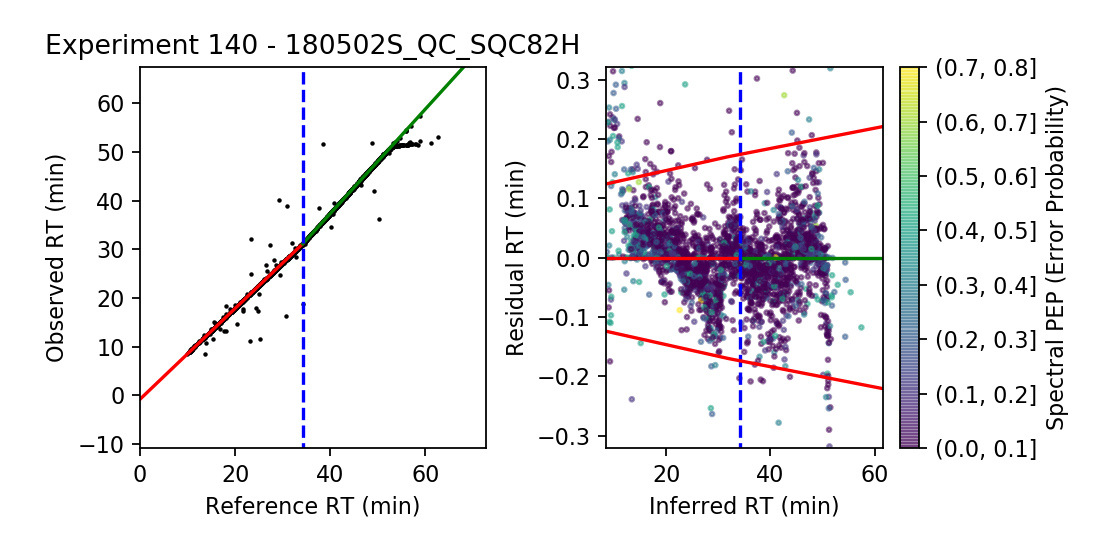

Supplement: S1 File — A optional HTML report generated by the dart_id Python script. The report gives a summary of the alignment for each experiment, as well as a broad overview of the performance of the run as a whole, by showing aggregate increases in PSMs at a chosen confidence threshold. (ZIP) [file pcbi.1007082.s001.zip › DART-ID_SCoPE-MS_Report/figures/alignment_140_180502S_QC_SQC82H.png]

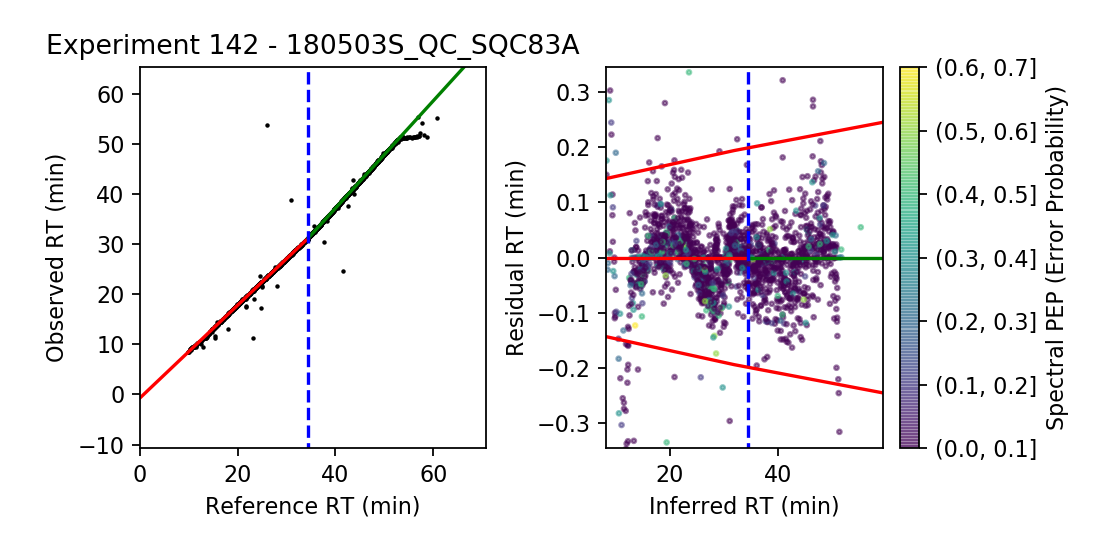

Supplement: S1 File — A optional HTML report generated by the dart_id Python script. The report gives a summary of the alignment for each experiment, as well as a broad overview of the performance of the run as a whole, by showing aggregate increases in PSMs at a chosen confidence threshold. (ZIP) [file pcbi.1007082.s001.zip › DART-ID_SCoPE-MS_Report/figures/alignment_142_180503S_QC_SQC83A.png]

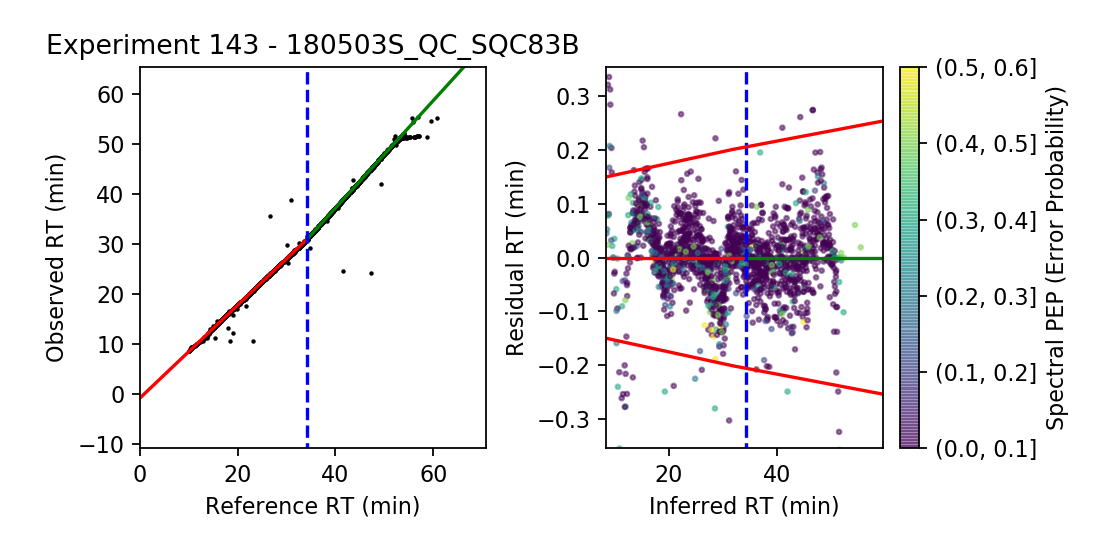

Supplement: S1 File — A optional HTML report generated by the dart_id Python script. The report gives a summary of the alignment for each experiment, as well as a broad overview of the performance of the run as a whole, by showing aggregate increases in PSMs at a chosen confidence threshold. (ZIP) [file pcbi.1007082.s001.zip › DART-ID_SCoPE-MS_Report/figures/alignment_143_180503S_QC_SQC83B.png]

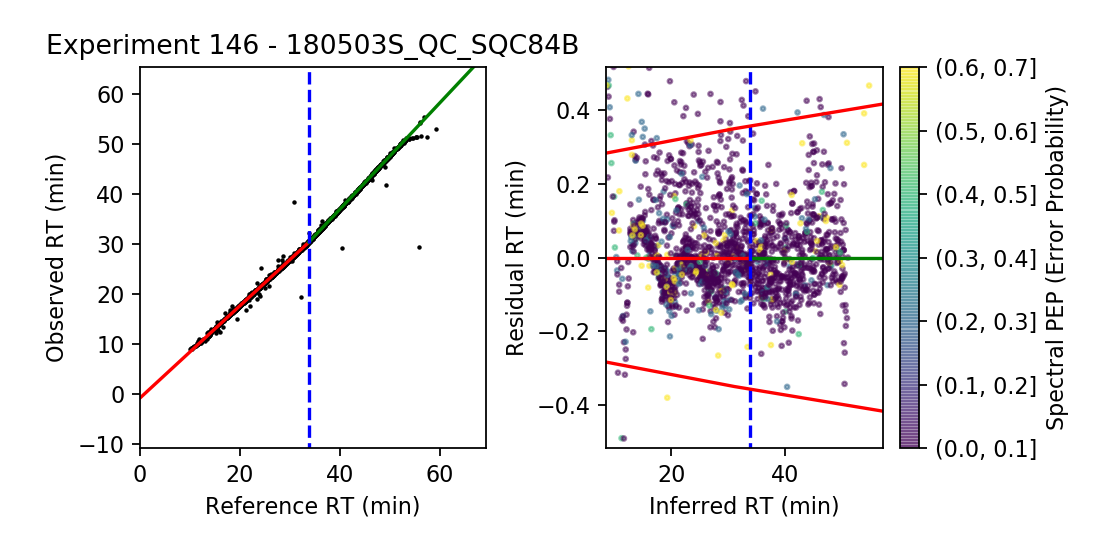

Supplement: S1 File — A optional HTML report generated by the dart_id Python script. The report gives a summary of the alignment for each experiment, as well as a broad overview of the performance of the run as a whole, by showing aggregate increases in PSMs at a chosen confidence threshold. (ZIP) [file pcbi.1007082.s001.zip › DART-ID_SCoPE-MS_Report/figures/alignment_146_180503S_QC_SQC84B.png]

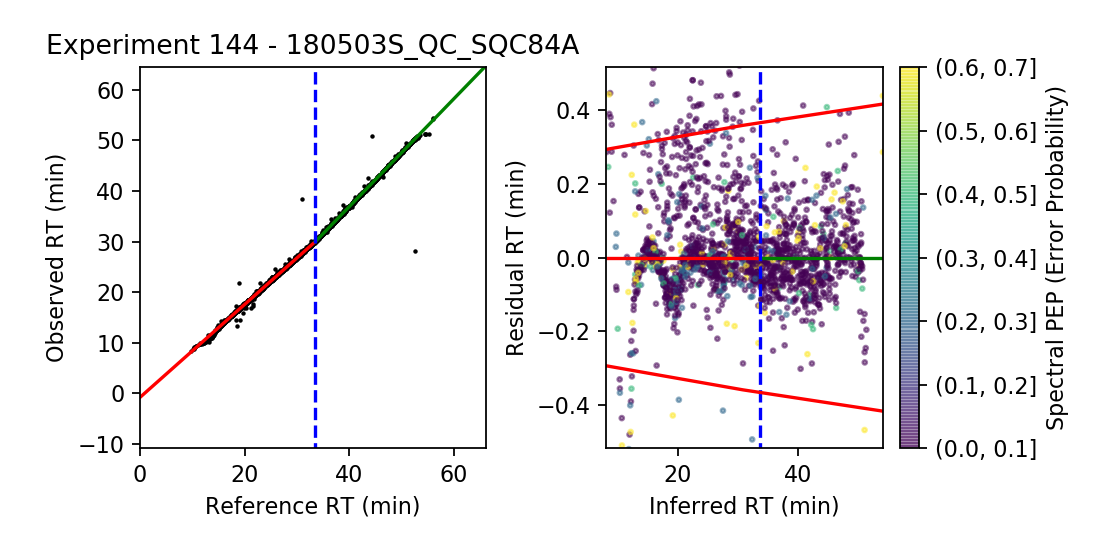

Supplement: S1 File — A optional HTML report generated by the dart_id Python script. The report gives a summary of the alignment for each experiment, as well as a broad overview of the performance of the run as a whole, by showing aggregate increases in PSMs at a chosen confidence threshold. (ZIP) [file pcbi.1007082.s001.zip › DART-ID_SCoPE-MS_Report/figures/alignment_144_180503S_QC_SQC84A.png]

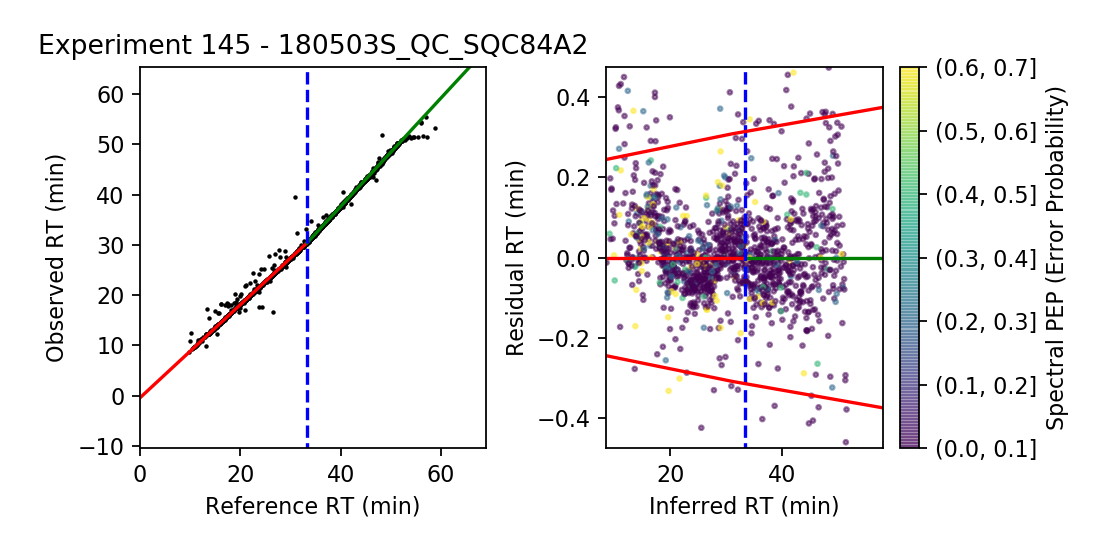

Supplement: S1 File — A optional HTML report generated by the dart_id Python script. The report gives a summary of the alignment for each experiment, as well as a broad overview of the performance of the run as a whole, by showing aggregate increases in PSMs at a chosen confidence threshold. (ZIP) [file pcbi.1007082.s001.zip › DART-ID_SCoPE-MS_Report/figures/alignment_145_180503S_QC_SQC84A2.png]

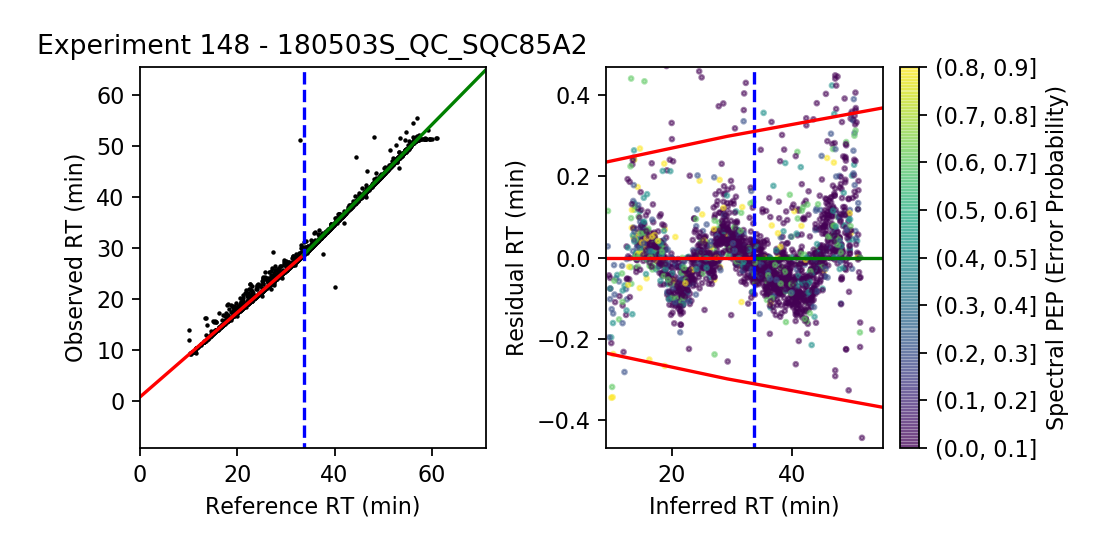

Supplement: S1 File — A optional HTML report generated by the dart_id Python script. The report gives a summary of the alignment for each experiment, as well as a broad overview of the performance of the run as a whole, by showing aggregate increases in PSMs at a chosen confidence threshold. (ZIP) [file pcbi.1007082.s001.zip › DART-ID_SCoPE-MS_Report/figures/alignment_148_180503S_QC_SQC85A2.png]

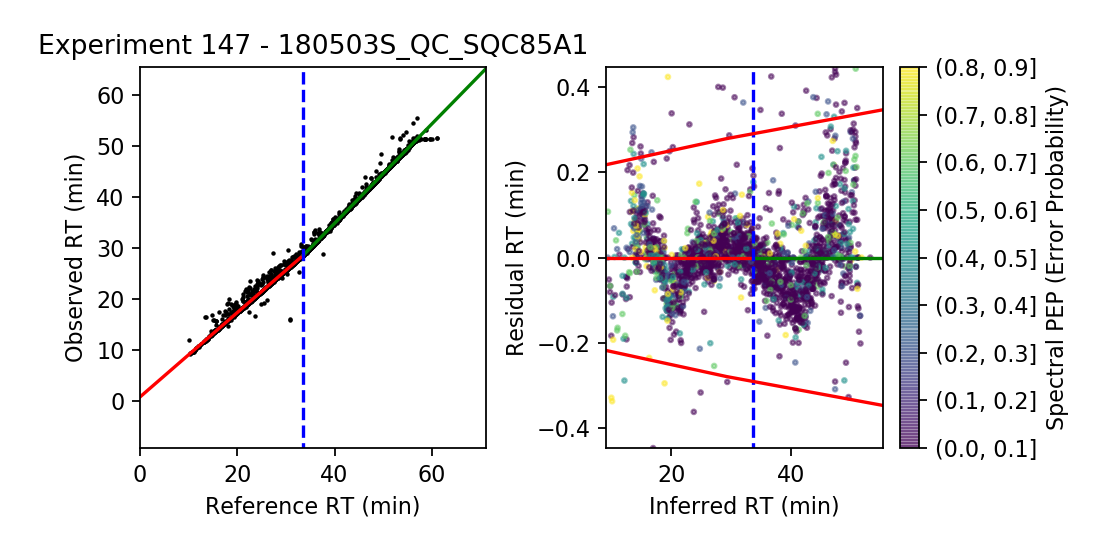

Supplement: S1 File — A optional HTML report generated by the dart_id Python script. The report gives a summary of the alignment for each experiment, as well as a broad overview of the performance of the run as a whole, by showing aggregate increases in PSMs at a chosen confidence threshold. (ZIP) [file pcbi.1007082.s001.zip › DART-ID_SCoPE-MS_Report/figures/alignment_147_180503S_QC_SQC85A1.png]

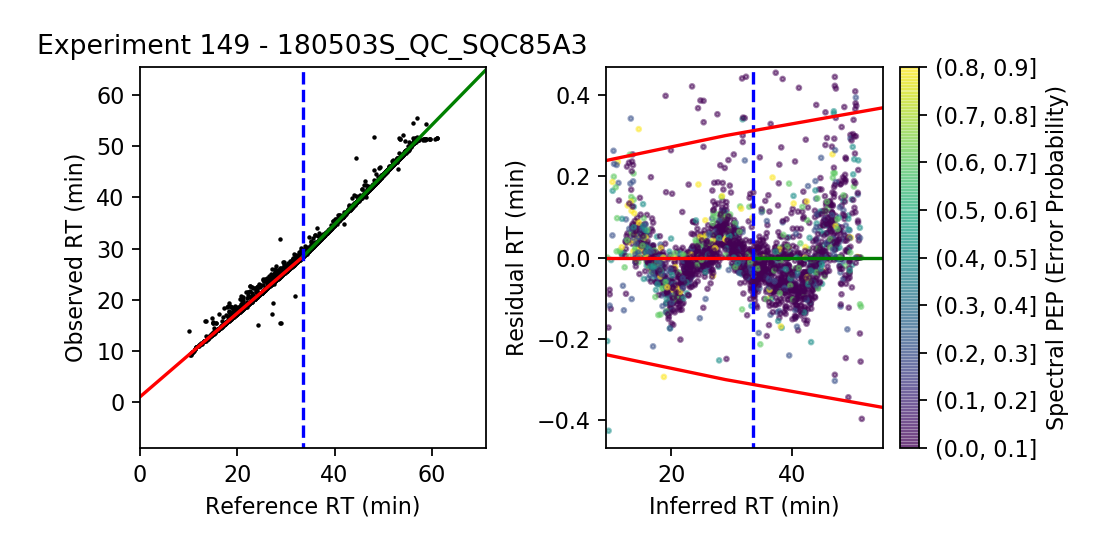

Supplement: S1 File — A optional HTML report generated by the dart_id Python script. The report gives a summary of the alignment for each experiment, as well as a broad overview of the performance of the run as a whole, by showing aggregate increases in PSMs at a chosen confidence threshold. (ZIP) [file pcbi.1007082.s001.zip › DART-ID_SCoPE-MS_Report/figures/alignment_149_180503S_QC_SQC85A3.png]

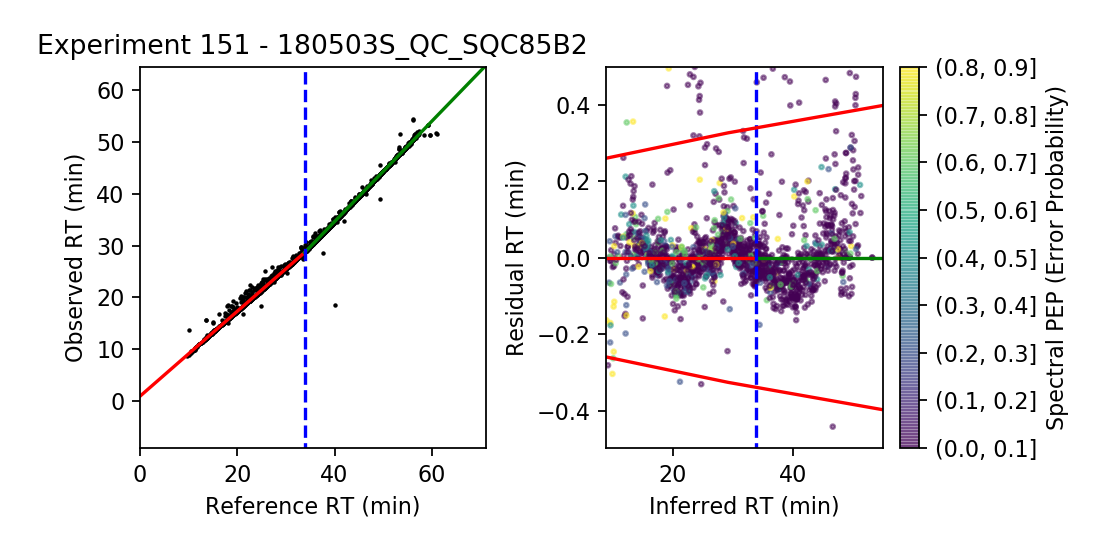

Supplement: S1 File — A optional HTML report generated by the dart_id Python script. The report gives a summary of the alignment for each experiment, as well as a broad overview of the performance of the run as a whole, by showing aggregate increases in PSMs at a chosen confidence threshold. (ZIP) [file pcbi.1007082.s001.zip › DART-ID_SCoPE-MS_Report/figures/alignment_151_180503S_QC_SQC85B2.png]

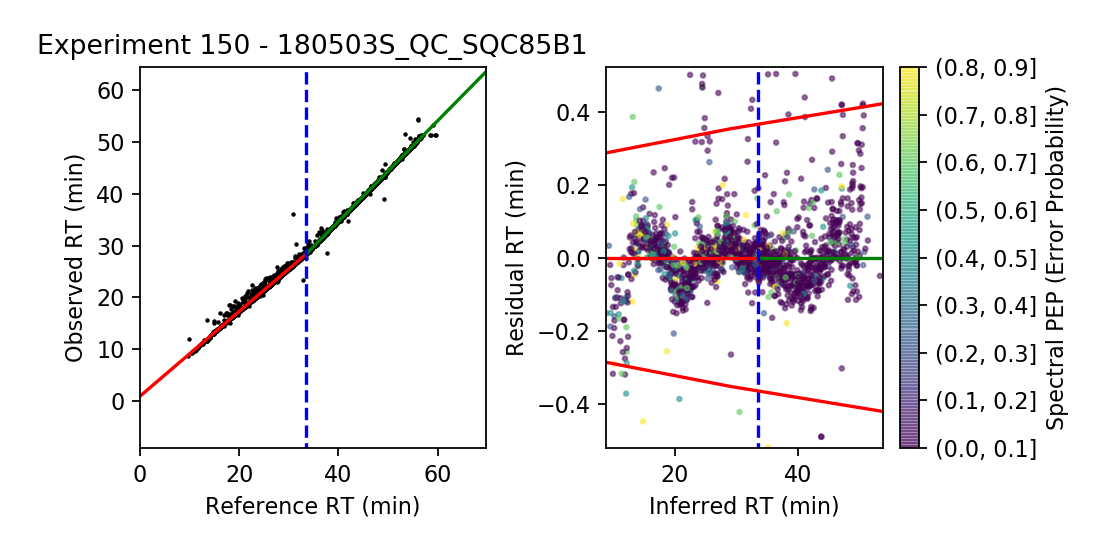

Supplement: S1 File — A optional HTML report generated by the dart_id Python script. The report gives a summary of the alignment for each experiment, as well as a broad overview of the performance of the run as a whole, by showing aggregate increases in PSMs at a chosen confidence threshold. (ZIP) [file pcbi.1007082.s001.zip › DART-ID_SCoPE-MS_Report/figures/alignment_150_180503S_QC_SQC85B1.png]

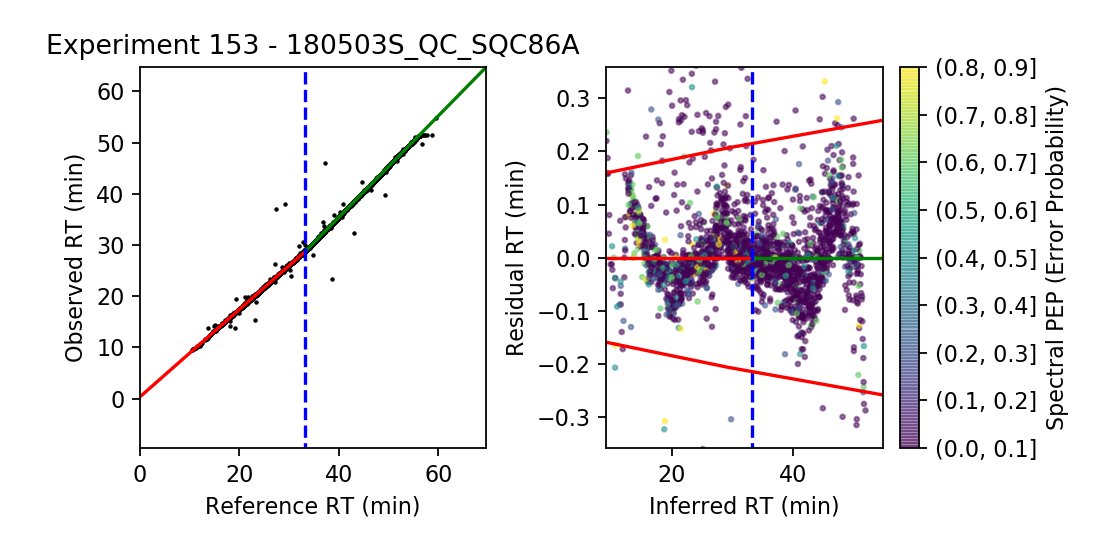

Supplement: S1 File — A optional HTML report generated by the dart_id Python script. The report gives a summary of the alignment for each experiment, as well as a broad overview of the performance of the run as a whole, by showing aggregate increases in PSMs at a chosen confidence threshold. (ZIP) [file pcbi.1007082.s001.zip › DART-ID_SCoPE-MS_Report/figures/alignment_153_180503S_QC_SQC86A.png]

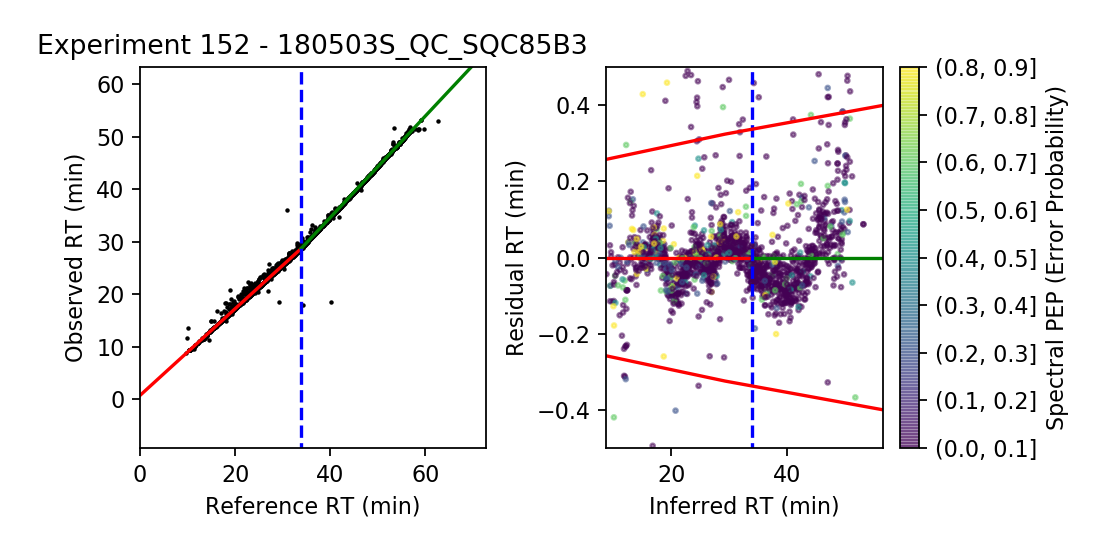

Supplement: S1 File — A optional HTML report generated by the dart_id Python script. The report gives a summary of the alignment for each experiment, as well as a broad overview of the performance of the run as a whole, by showing aggregate increases in PSMs at a chosen confidence threshold. (ZIP) [file pcbi.1007082.s001.zip › DART-ID_SCoPE-MS_Report/figures/alignment_152_180503S_QC_SQC85B3.png]

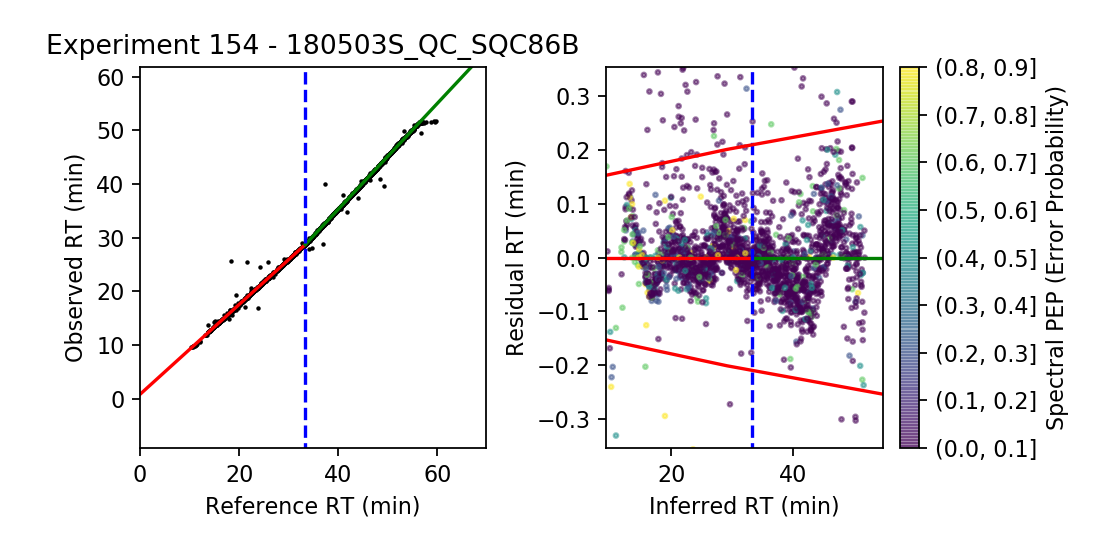

Supplement: S1 File — A optional HTML report generated by the dart_id Python script. The report gives a summary of the alignment for each experiment, as well as a broad overview of the performance of the run as a whole, by showing aggregate increases in PSMs at a chosen confidence threshold. (ZIP) [file pcbi.1007082.s001.zip › DART-ID_SCoPE-MS_Report/figures/alignment_154_180503S_QC_SQC86B.png]

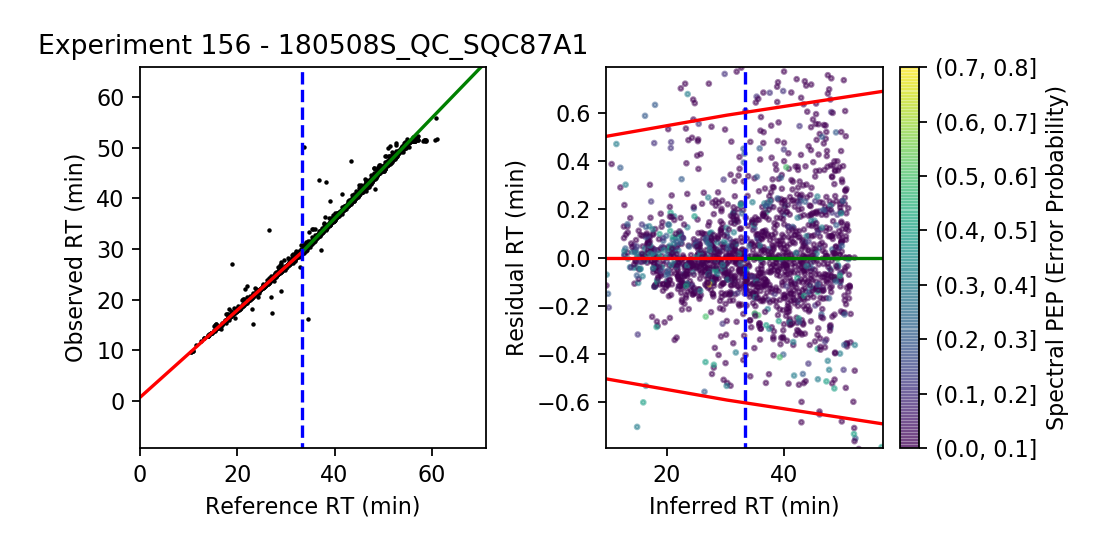

Supplement: S1 File — A optional HTML report generated by the dart_id Python script. The report gives a summary of the alignment for each experiment, as well as a broad overview of the performance of the run as a whole, by showing aggregate increases in PSMs at a chosen confidence threshold. (ZIP) [file pcbi.1007082.s001.zip › DART-ID_SCoPE-MS_Report/figures/alignment_156_180508S_QC_SQC87A1.png]

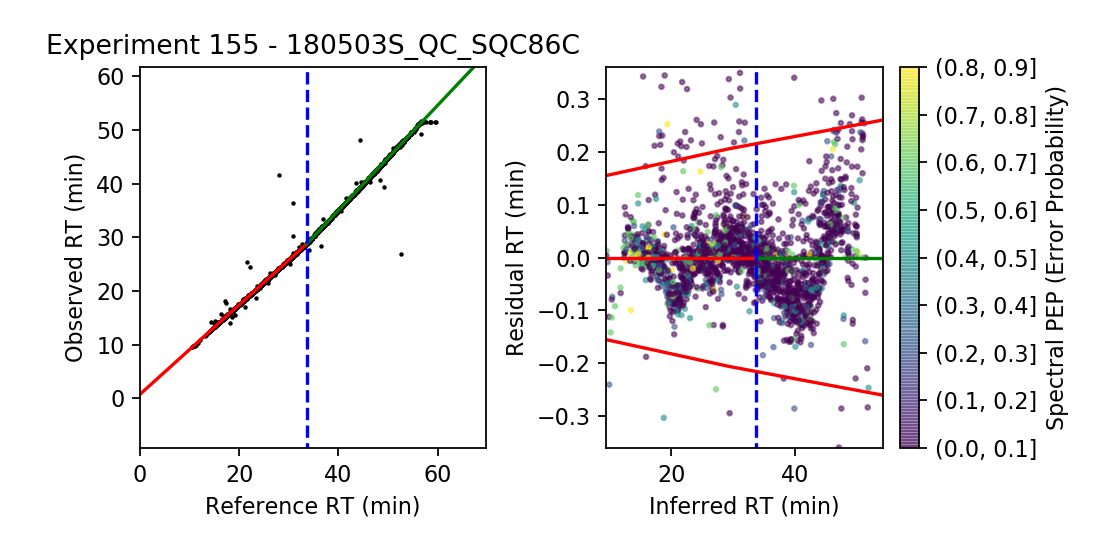

Supplement: S1 File — A optional HTML report generated by the dart_id Python script. The report gives a summary of the alignment for each experiment, as well as a broad overview of the performance of the run as a whole, by showing aggregate increases in PSMs at a chosen confidence threshold. (ZIP) [file pcbi.1007082.s001.zip › DART-ID_SCoPE-MS_Report/figures/alignment_155_180503S_QC_SQC86C.png]

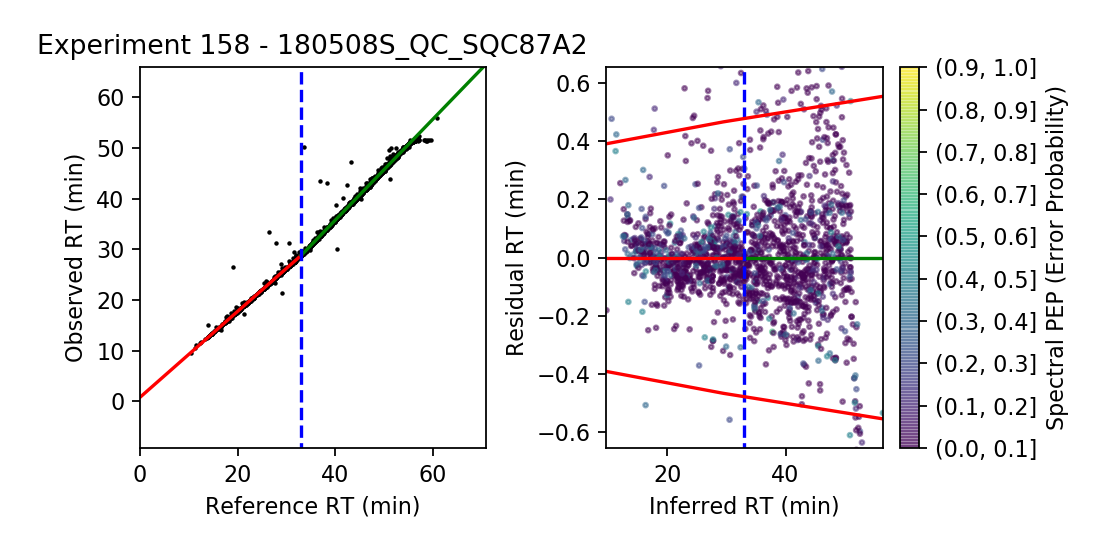

Supplement: S1 File — A optional HTML report generated by the dart_id Python script. The report gives a summary of the alignment for each experiment, as well as a broad overview of the performance of the run as a whole, by showing aggregate increases in PSMs at a chosen confidence threshold. (ZIP) [file pcbi.1007082.s001.zip › DART-ID_SCoPE-MS_Report/figures/alignment_158_180508S_QC_SQC87A2.png]

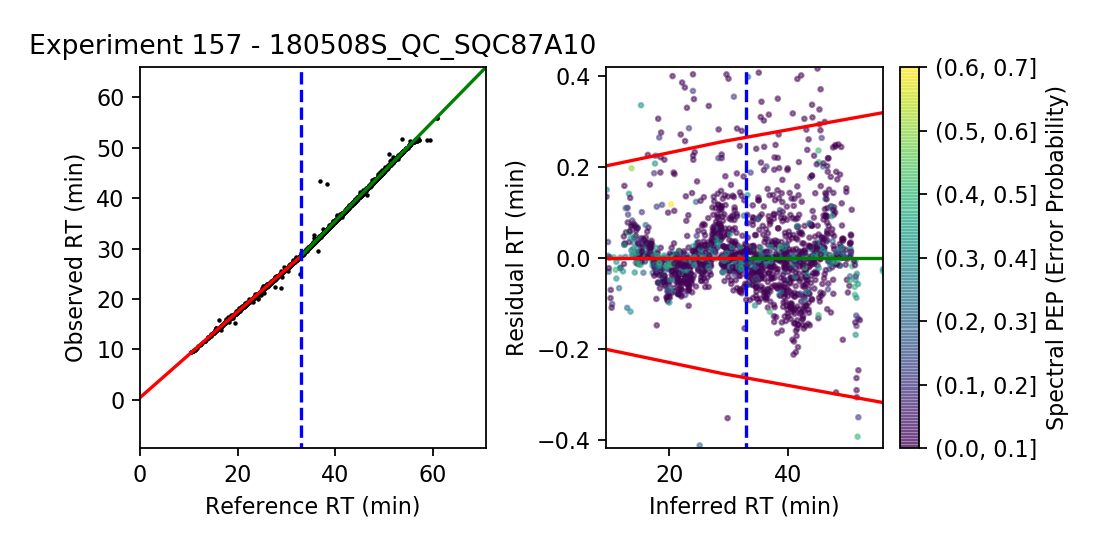

Supplement: S1 File — A optional HTML report generated by the dart_id Python script. The report gives a summary of the alignment for each experiment, as well as a broad overview of the performance of the run as a whole, by showing aggregate increases in PSMs at a chosen confidence threshold. (ZIP) [file pcbi.1007082.s001.zip › DART-ID_SCoPE-MS_Report/figures/alignment_157_180508S_QC_SQC87A10.png]

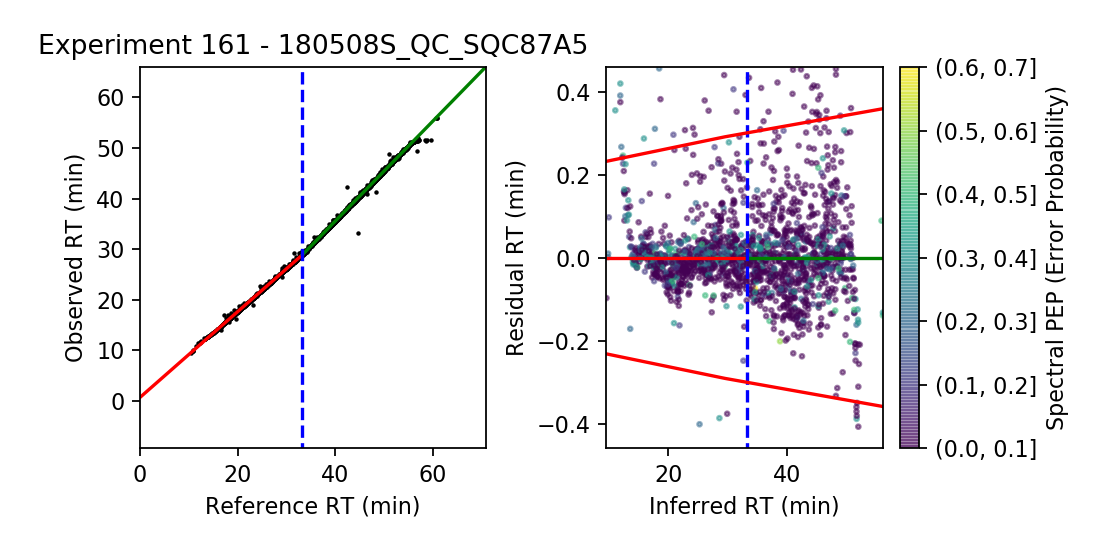

Supplement: S1 File — A optional HTML report generated by the dart_id Python script. The report gives a summary of the alignment for each experiment, as well as a broad overview of the performance of the run as a whole, by showing aggregate increases in PSMs at a chosen confidence threshold. (ZIP) [file pcbi.1007082.s001.zip › DART-ID_SCoPE-MS_Report/figures/alignment_161_180508S_QC_SQC87A5.png]

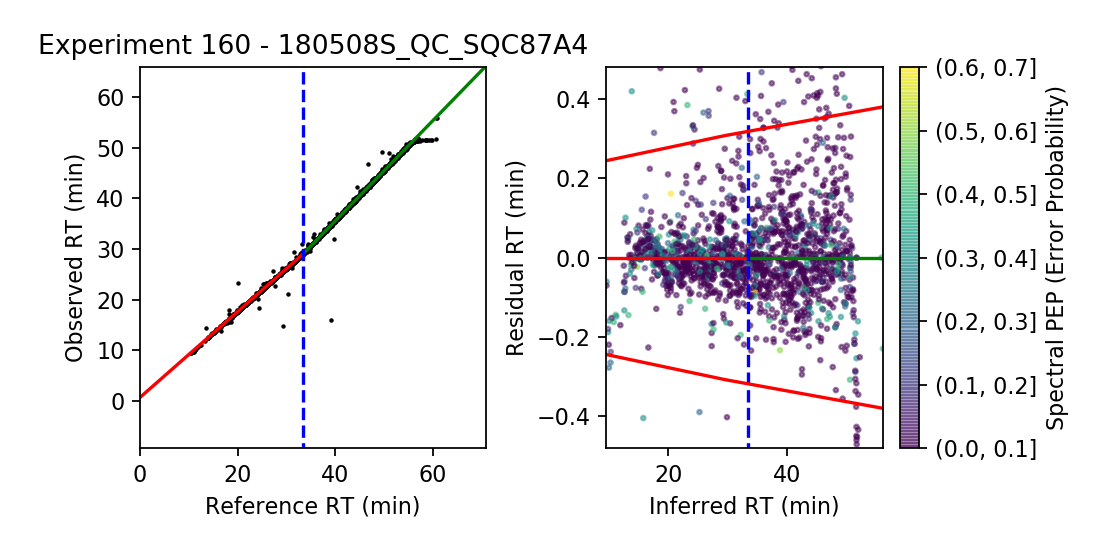

Supplement: S1 File — A optional HTML report generated by the dart_id Python script. The report gives a summary of the alignment for each experiment, as well as a broad overview of the performance of the run as a whole, by showing aggregate increases in PSMs at a chosen confidence threshold. (ZIP) [file pcbi.1007082.s001.zip › DART-ID_SCoPE-MS_Report/figures/alignment_160_180508S_QC_SQC87A4.png]

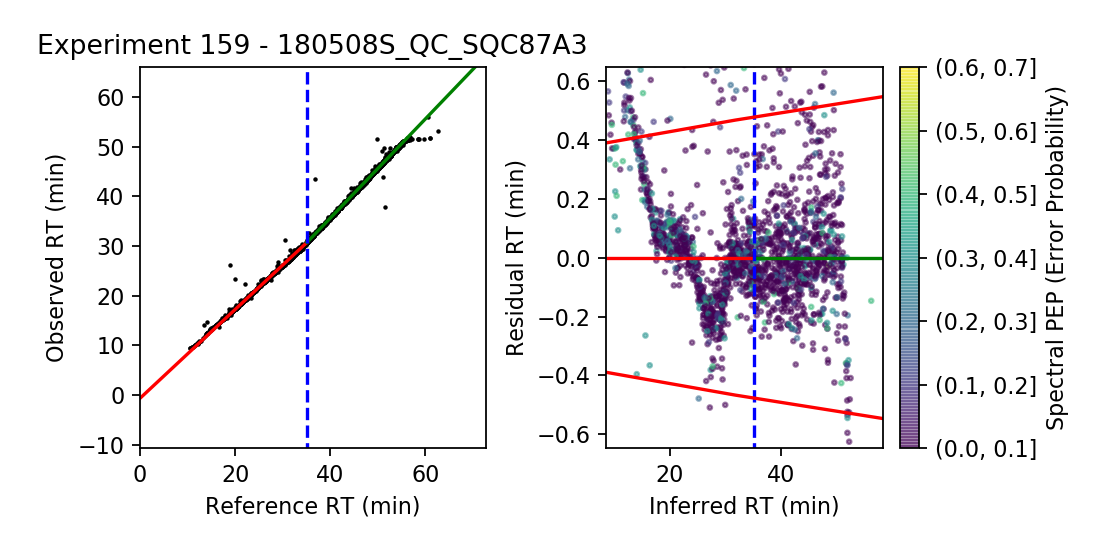

Supplement: S1 File — A optional HTML report generated by the dart_id Python script. The report gives a summary of the alignment for each experiment, as well as a broad overview of the performance of the run as a whole, by showing aggregate increases in PSMs at a chosen confidence threshold. (ZIP) [file pcbi.1007082.s001.zip › DART-ID_SCoPE-MS_Report/figures/alignment_159_180508S_QC_SQC87A3.png]

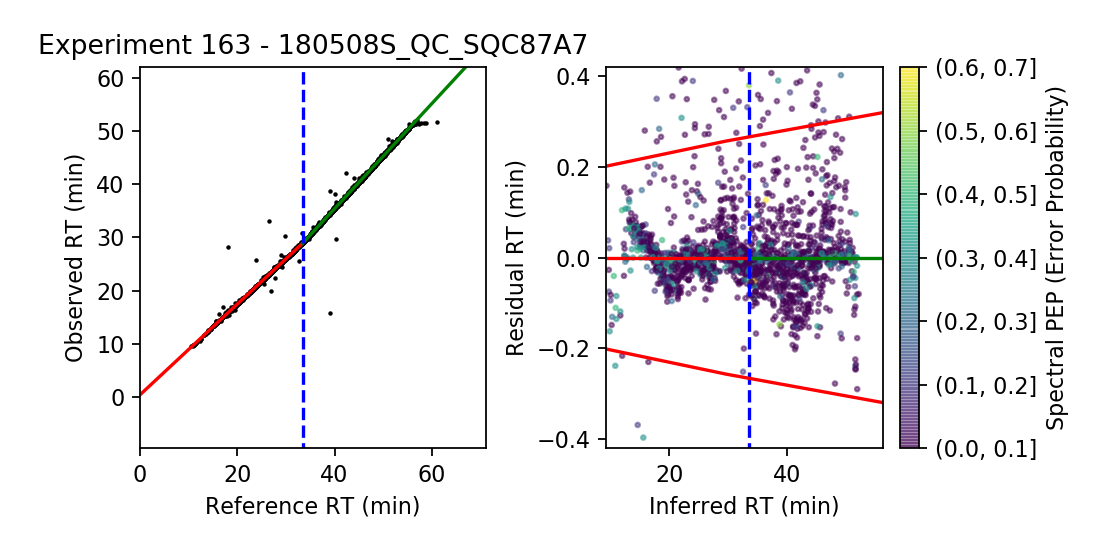

Supplement: S1 File — A optional HTML report generated by the dart_id Python script. The report gives a summary of the alignment for each experiment, as well as a broad overview of the performance of the run as a whole, by showing aggregate increases in PSMs at a chosen confidence threshold. (ZIP) [file pcbi.1007082.s001.zip › DART-ID_SCoPE-MS_Report/figures/alignment_163_180508S_QC_SQC87A7.png]

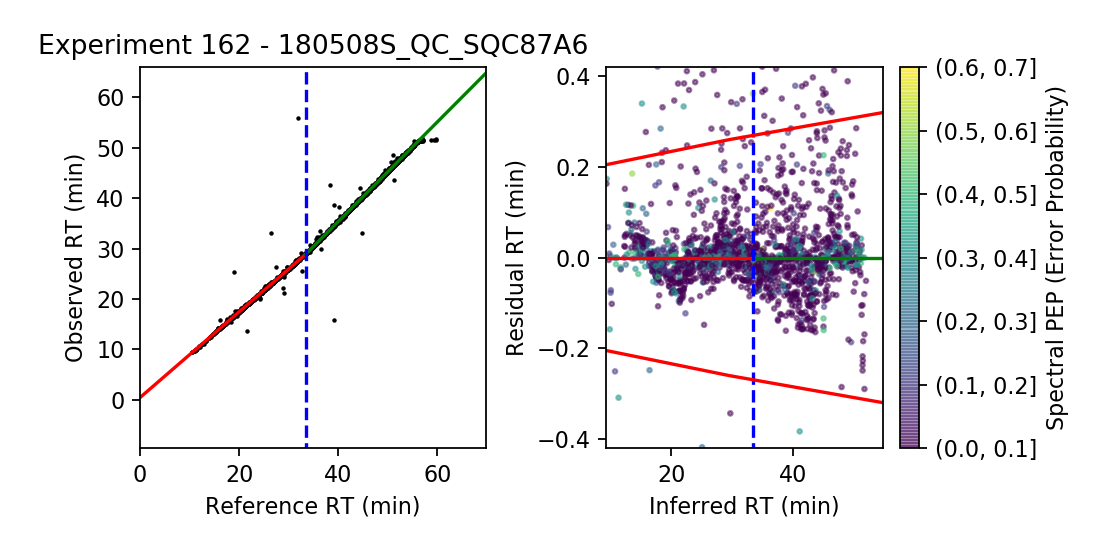

Supplement: S1 File — A optional HTML report generated by the dart_id Python script. The report gives a summary of the alignment for each experiment, as well as a broad overview of the performance of the run as a whole, by showing aggregate increases in PSMs at a chosen confidence threshold. (ZIP) [file pcbi.1007082.s001.zip › DART-ID_SCoPE-MS_Report/figures/alignment_162_180508S_QC_SQC87A6.png]

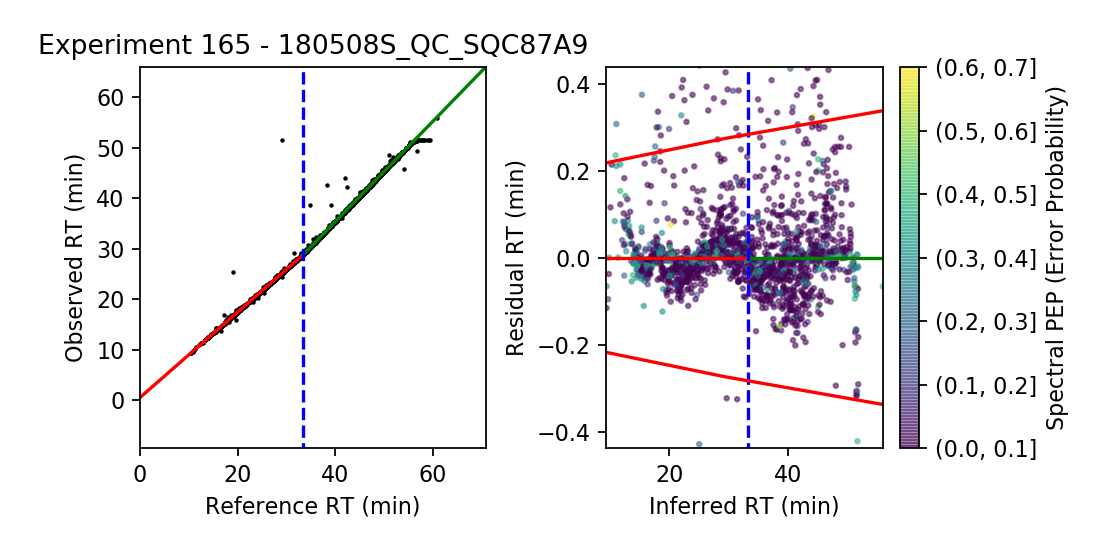

Supplement: S1 File — A optional HTML report generated by the dart_id Python script. The report gives a summary of the alignment for each experiment, as well as a broad overview of the performance of the run as a whole, by showing aggregate increases in PSMs at a chosen confidence threshold. (ZIP) [file pcbi.1007082.s001.zip › DART-ID_SCoPE-MS_Report/figures/alignment_165_180508S_QC_SQC87A9.png]

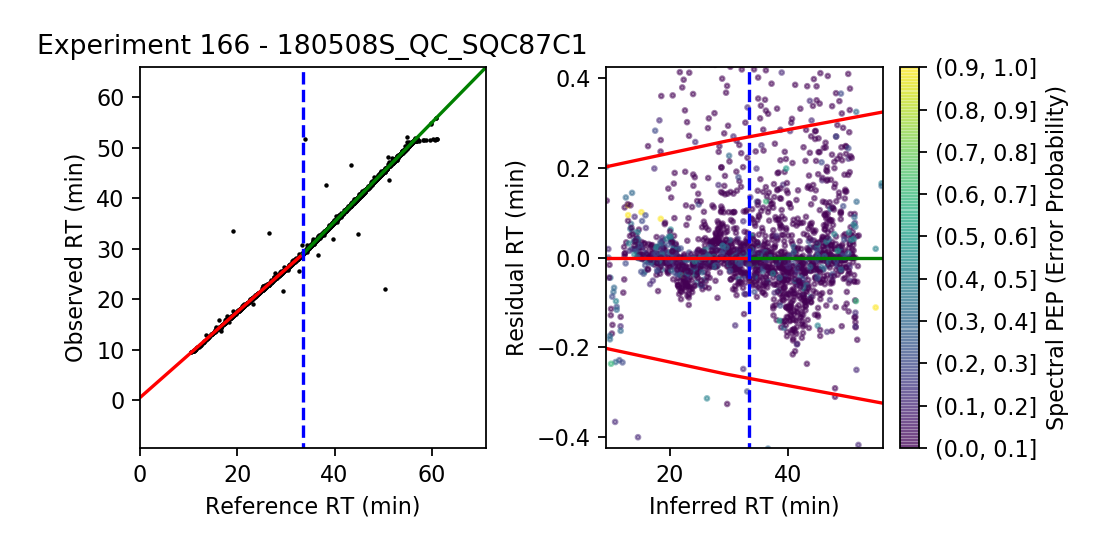

Supplement: S1 File — A optional HTML report generated by the dart_id Python script. The report gives a summary of the alignment for each experiment, as well as a broad overview of the performance of the run as a whole, by showing aggregate increases in PSMs at a chosen confidence threshold. (ZIP) [file pcbi.1007082.s001.zip › DART-ID_SCoPE-MS_Report/figures/alignment_166_180508S_QC_SQC87C1.png]

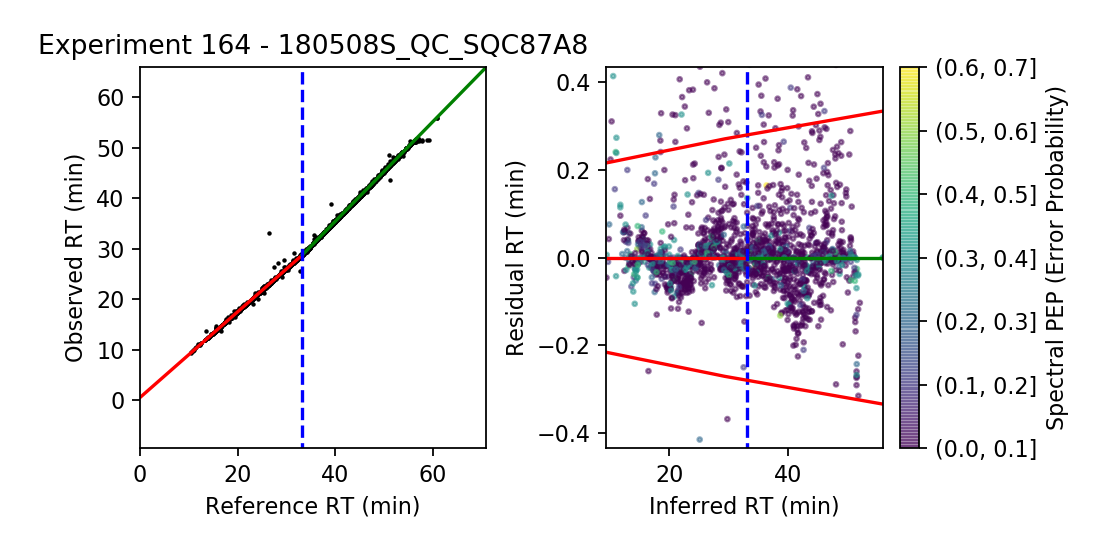

Supplement: S1 File — A optional HTML report generated by the dart_id Python script. The report gives a summary of the alignment for each experiment, as well as a broad overview of the performance of the run as a whole, by showing aggregate increases in PSMs at a chosen confidence threshold. (ZIP) [file pcbi.1007082.s001.zip › DART-ID_SCoPE-MS_Report/figures/alignment_164_180508S_QC_SQC87A8.png]

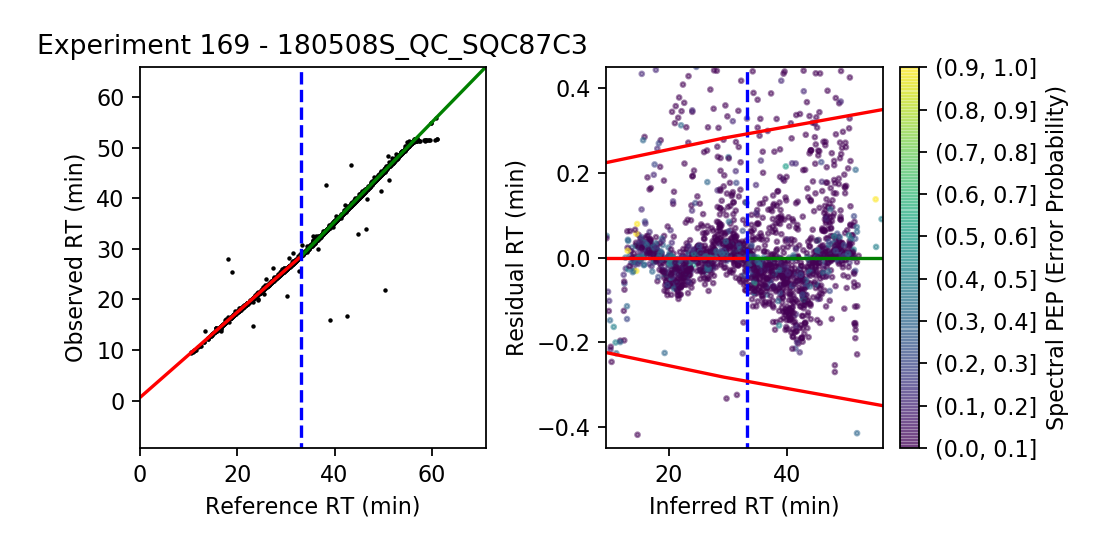

Supplement: S1 File — A optional HTML report generated by the dart_id Python script. The report gives a summary of the alignment for each experiment, as well as a broad overview of the performance of the run as a whole, by showing aggregate increases in PSMs at a chosen confidence threshold. (ZIP) [file pcbi.1007082.s001.zip › DART-ID_SCoPE-MS_Report/figures/alignment_169_180508S_QC_SQC87C3.png]

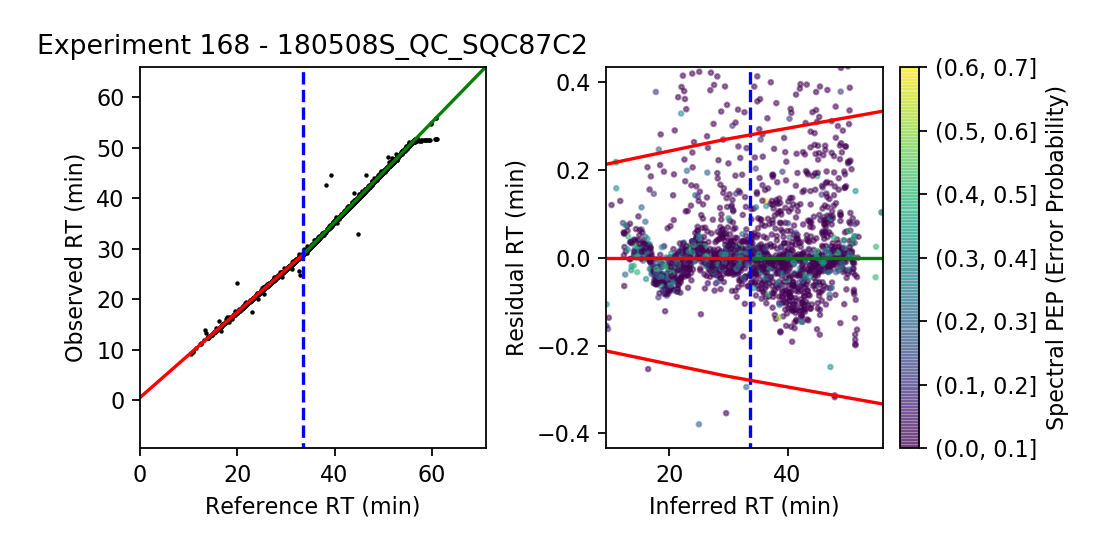

Supplement: S1 File — A optional HTML report generated by the dart_id Python script. The report gives a summary of the alignment for each experiment, as well as a broad overview of the performance of the run as a whole, by showing aggregate increases in PSMs at a chosen confidence threshold. (ZIP) [file pcbi.1007082.s001.zip › DART-ID_SCoPE-MS_Report/figures/alignment_168_180508S_QC_SQC87C2.png]

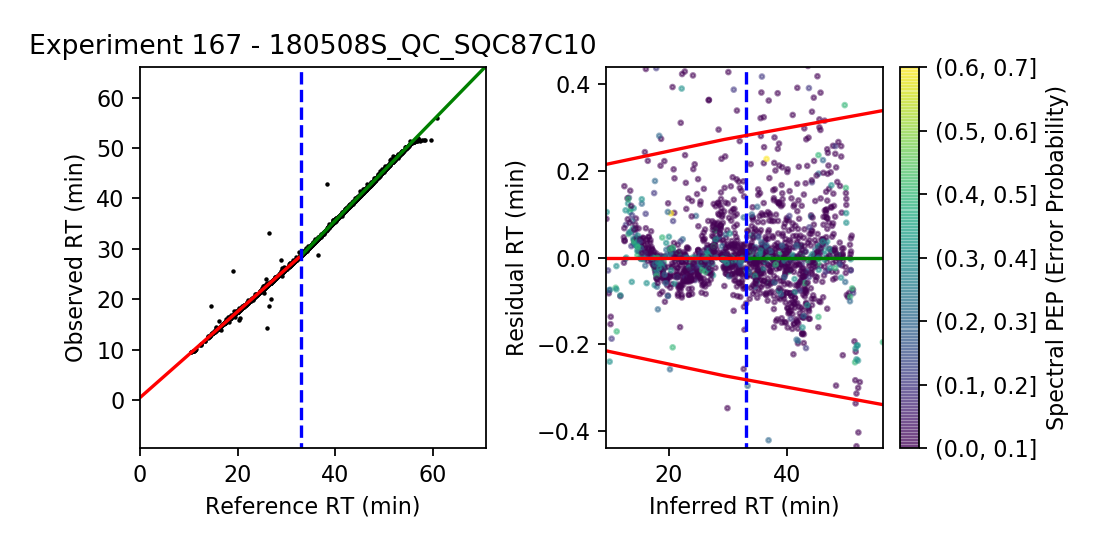

Supplement: S1 File — A optional HTML report generated by the dart_id Python script. The report gives a summary of the alignment for each experiment, as well as a broad overview of the performance of the run as a whole, by showing aggregate increases in PSMs at a chosen confidence threshold. (ZIP) [file pcbi.1007082.s001.zip › DART-ID_SCoPE-MS_Report/figures/alignment_167_180508S_QC_SQC87C10.png]

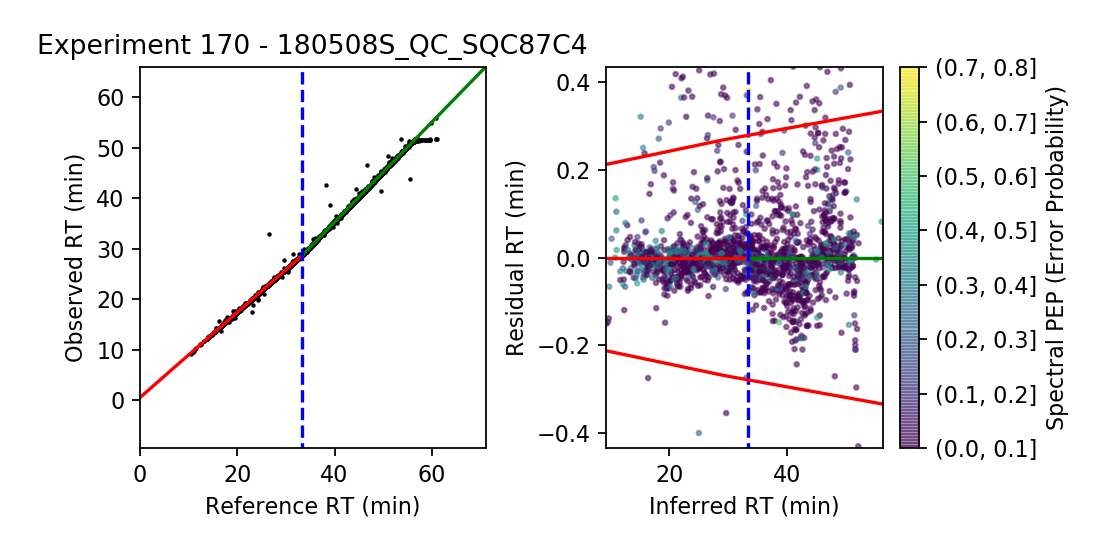

Supplement: S1 File — A optional HTML report generated by the dart_id Python script. The report gives a summary of the alignment for each experiment, as well as a broad overview of the performance of the run as a whole, by showing aggregate increases in PSMs at a chosen confidence threshold. (ZIP) [file pcbi.1007082.s001.zip › DART-ID_SCoPE-MS_Report/figures/alignment_170_180508S_QC_SQC87C4.png]

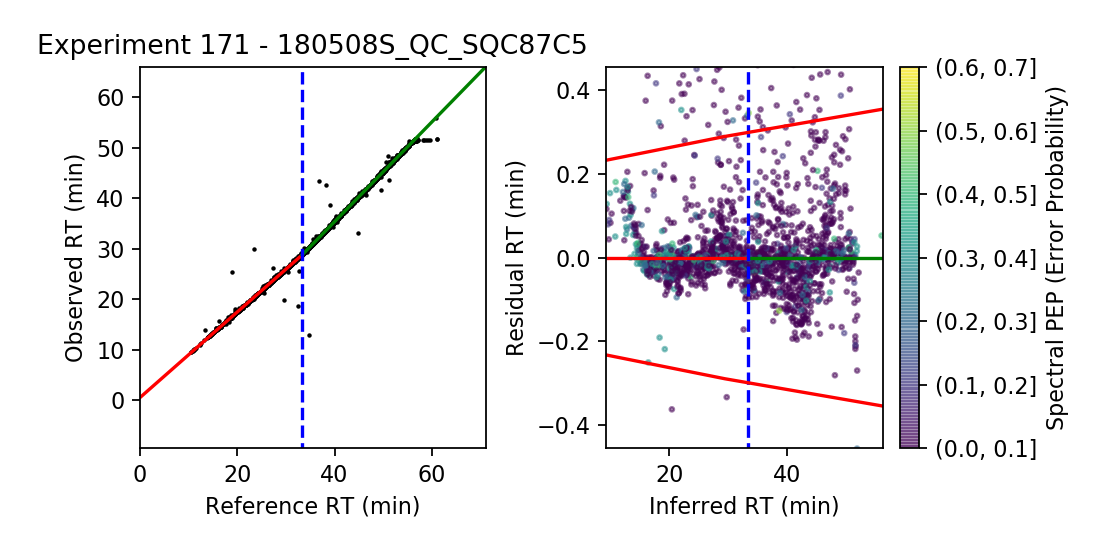

Supplement: S1 File — A optional HTML report generated by the dart_id Python script. The report gives a summary of the alignment for each experiment, as well as a broad overview of the performance of the run as a whole, by showing aggregate increases in PSMs at a chosen confidence threshold. (ZIP) [file pcbi.1007082.s001.zip › DART-ID_SCoPE-MS_Report/figures/alignment_171_180508S_QC_SQC87C5.png]

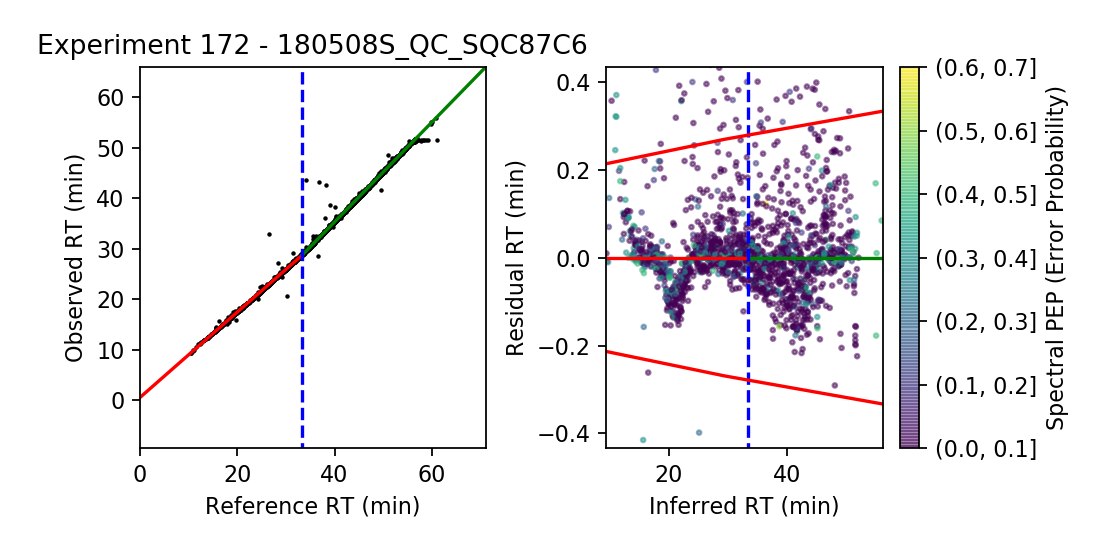

Supplement: S1 File — A optional HTML report generated by the dart_id Python script. The report gives a summary of the alignment for each experiment, as well as a broad overview of the performance of the run as a whole, by showing aggregate increases in PSMs at a chosen confidence threshold. (ZIP) [file pcbi.1007082.s001.zip › DART-ID_SCoPE-MS_Report/figures/alignment_172_180508S_QC_SQC87C6.png]

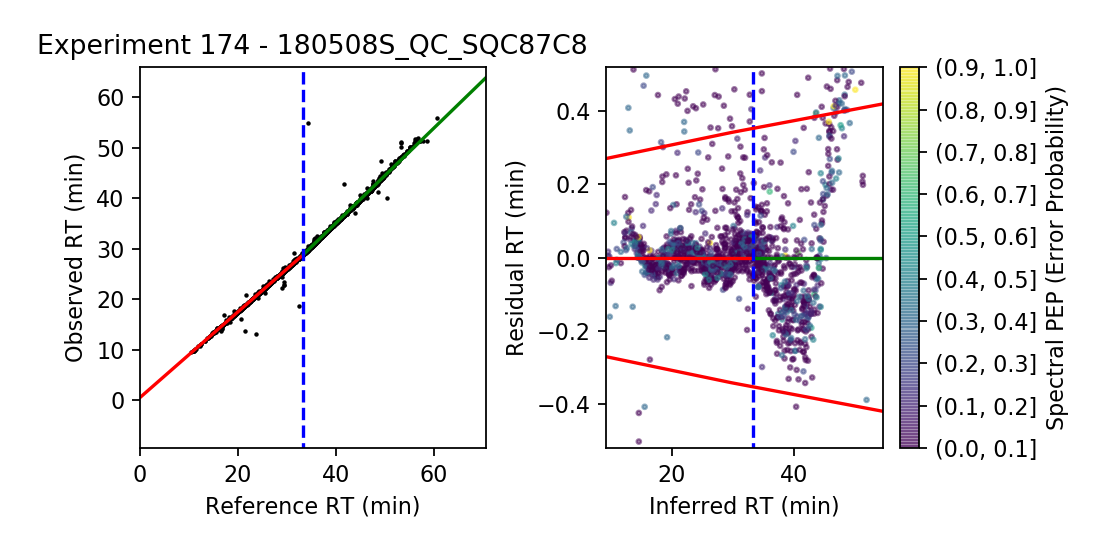

Supplement: S1 File — A optional HTML report generated by the dart_id Python script. The report gives a summary of the alignment for each experiment, as well as a broad overview of the performance of the run as a whole, by showing aggregate increases in PSMs at a chosen confidence threshold. (ZIP) [file pcbi.1007082.s001.zip › DART-ID_SCoPE-MS_Report/figures/alignment_174_180508S_QC_SQC87C8.png]

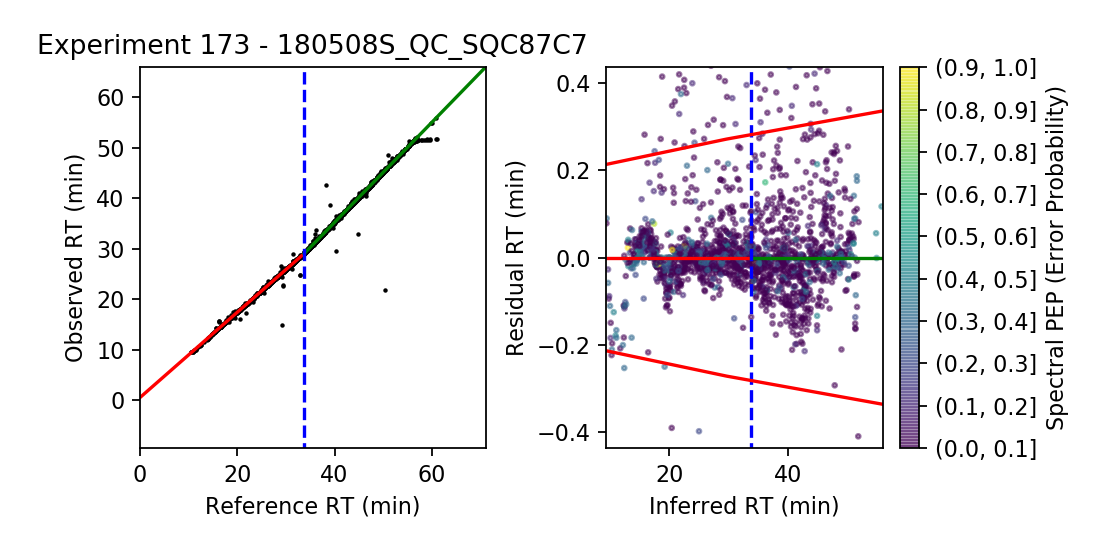

Supplement: S1 File — A optional HTML report generated by the dart_id Python script. The report gives a summary of the alignment for each experiment, as well as a broad overview of the performance of the run as a whole, by showing aggregate increases in PSMs at a chosen confidence threshold. (ZIP) [file pcbi.1007082.s001.zip › DART-ID_SCoPE-MS_Report/figures/alignment_173_180508S_QC_SQC87C7.png]

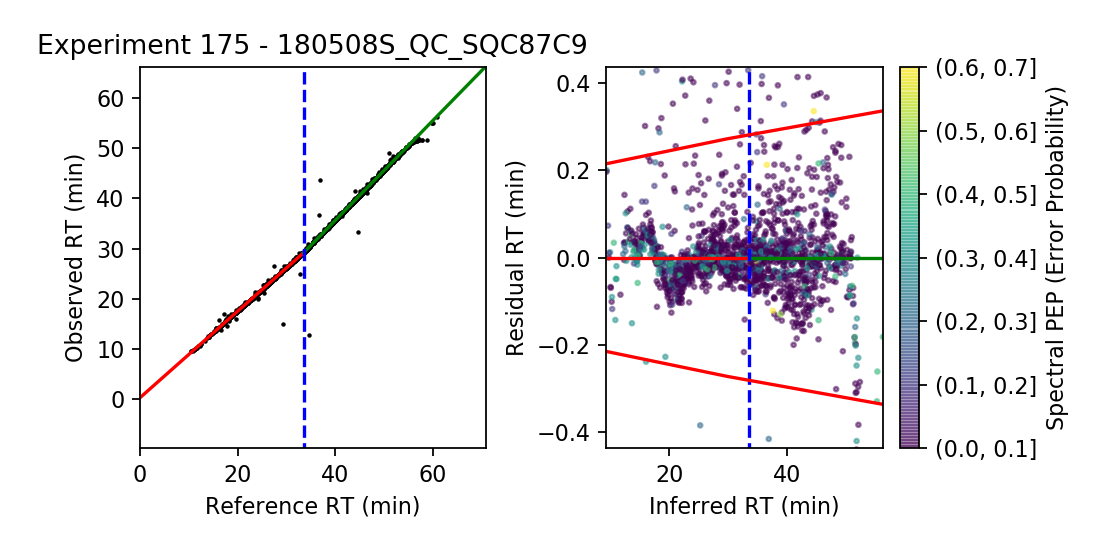

Supplement: S1 File — A optional HTML report generated by the dart_id Python script. The report gives a summary of the alignment for each experiment, as well as a broad overview of the performance of the run as a whole, by showing aggregate increases in PSMs at a chosen confidence threshold. (ZIP) [file pcbi.1007082.s001.zip › DART-ID_SCoPE-MS_Report/figures/alignment_175_180508S_QC_SQC87C9.png]

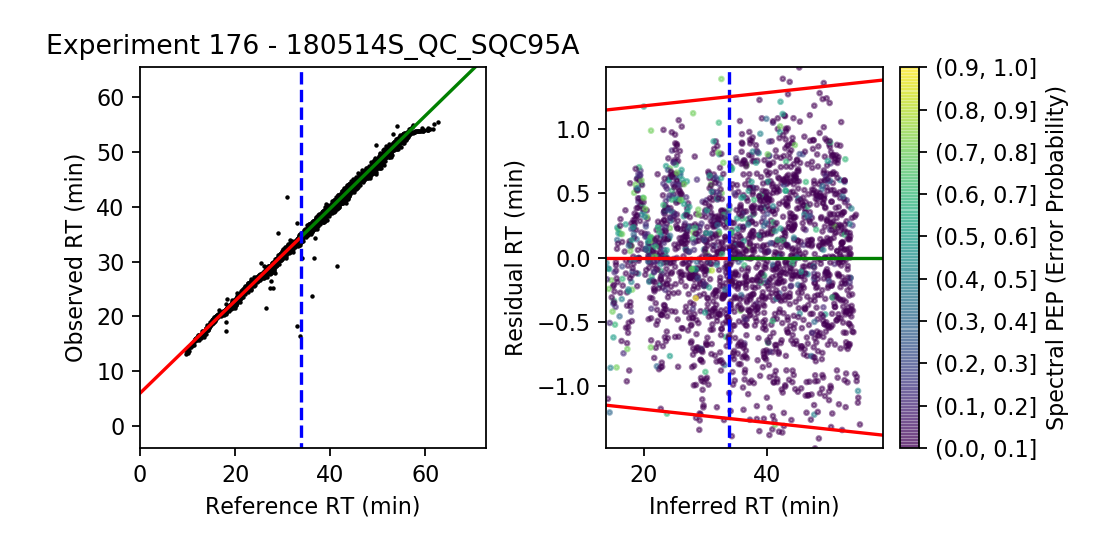

Supplement: S1 File — A optional HTML report generated by the dart_id Python script. The report gives a summary of the alignment for each experiment, as well as a broad overview of the performance of the run as a whole, by showing aggregate increases in PSMs at a chosen confidence threshold. (ZIP) [file pcbi.1007082.s001.zip › DART-ID_SCoPE-MS_Report/figures/alignment_176_180514S_QC_SQC95A.png]

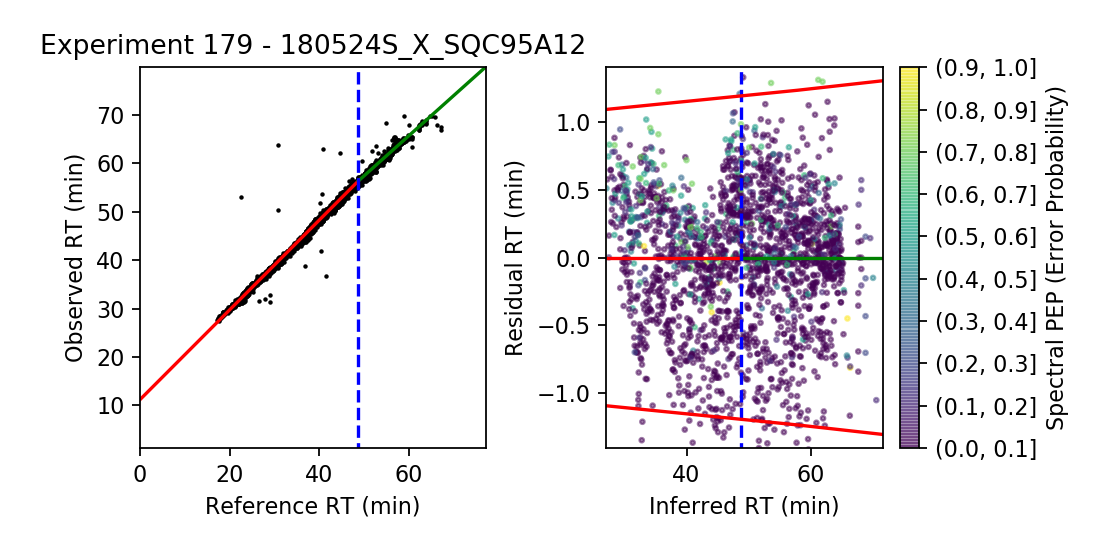

Supplement: S1 File — A optional HTML report generated by the dart_id Python script. The report gives a summary of the alignment for each experiment, as well as a broad overview of the performance of the run as a whole, by showing aggregate increases in PSMs at a chosen confidence threshold. (ZIP) [file pcbi.1007082.s001.zip › DART-ID_SCoPE-MS_Report/figures/alignment_179_180524S_X_SQC95A12.png]

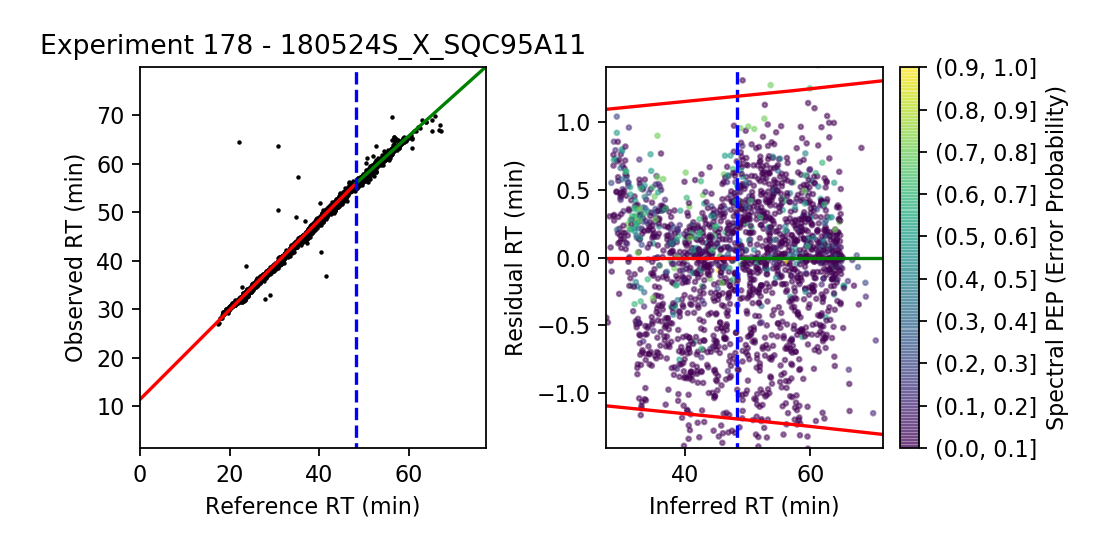

Supplement: S1 File — A optional HTML report generated by the dart_id Python script. The report gives a summary of the alignment for each experiment, as well as a broad overview of the performance of the run as a whole, by showing aggregate increases in PSMs at a chosen confidence threshold. (ZIP) [file pcbi.1007082.s001.zip › DART-ID_SCoPE-MS_Report/figures/alignment_178_180524S_X_SQC95A11.png]

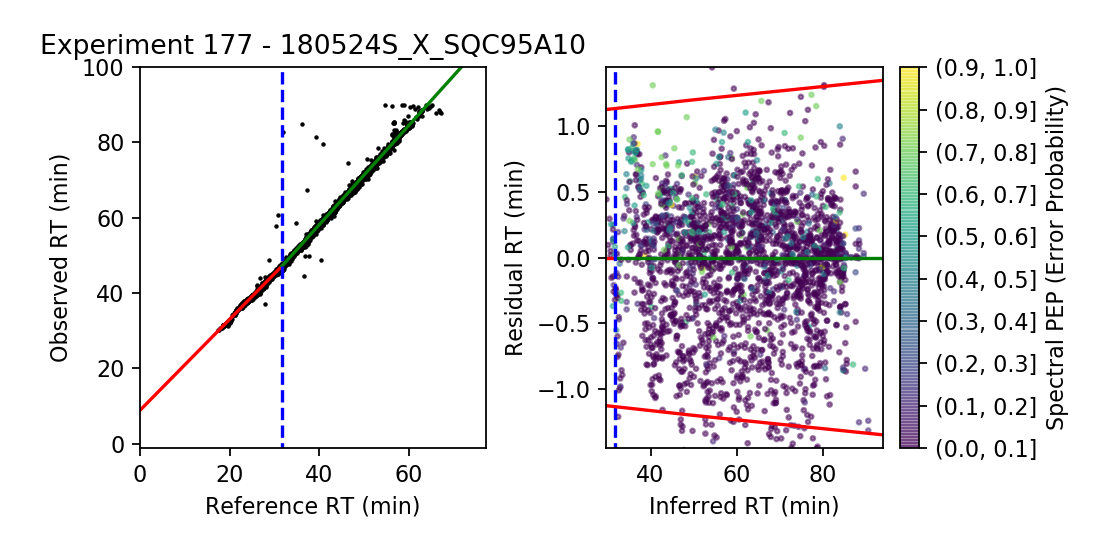

Supplement: S1 File — A optional HTML report generated by the dart_id Python script. The report gives a summary of the alignment for each experiment, as well as a broad overview of the performance of the run as a whole, by showing aggregate increases in PSMs at a chosen confidence threshold. (ZIP) [file pcbi.1007082.s001.zip › DART-ID_SCoPE-MS_Report/figures/alignment_177_180524S_X_SQC95A10.png]

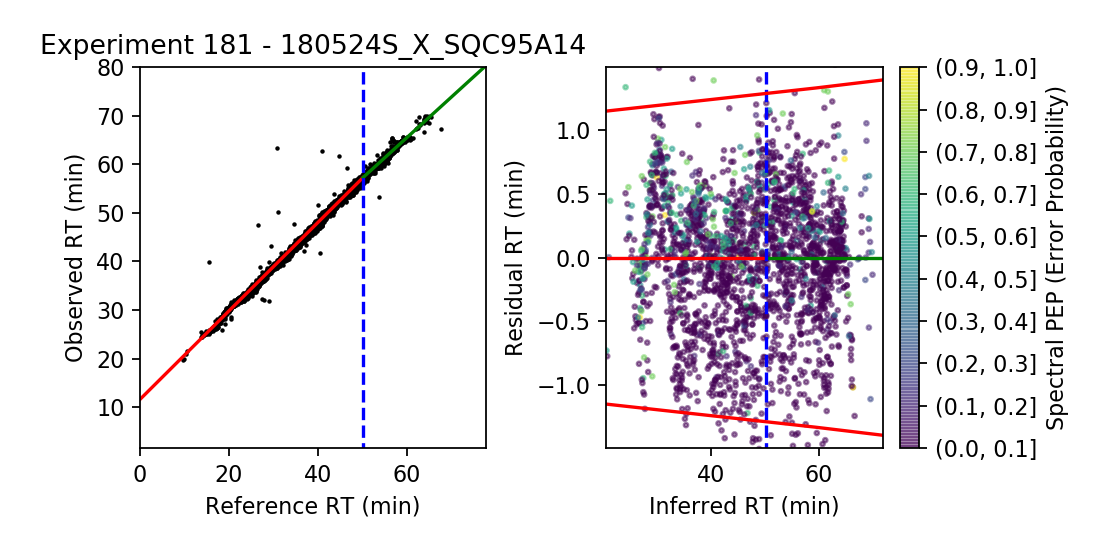

Supplement: S1 File — A optional HTML report generated by the dart_id Python script. The report gives a summary of the alignment for each experiment, as well as a broad overview of the performance of the run as a whole, by showing aggregate increases in PSMs at a chosen confidence threshold. (ZIP) [file pcbi.1007082.s001.zip › DART-ID_SCoPE-MS_Report/figures/alignment_181_180524S_X_SQC95A14.png]

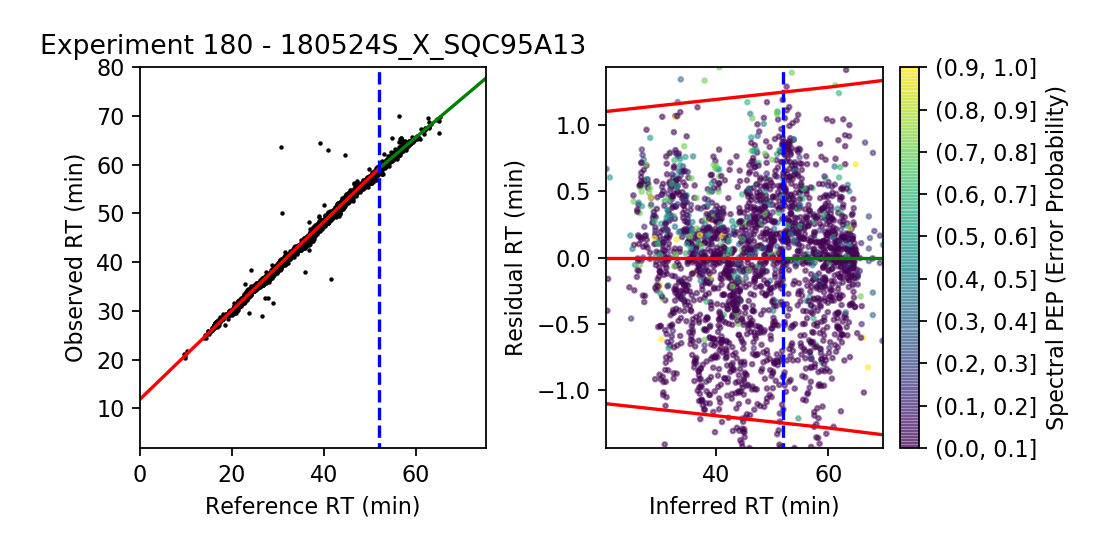

Supplement: S1 File — A optional HTML report generated by the dart_id Python script. The report gives a summary of the alignment for each experiment, as well as a broad overview of the performance of the run as a whole, by showing aggregate increases in PSMs at a chosen confidence threshold. (ZIP) [file pcbi.1007082.s001.zip › DART-ID_SCoPE-MS_Report/figures/alignment_180_180524S_X_SQC95A13.png]

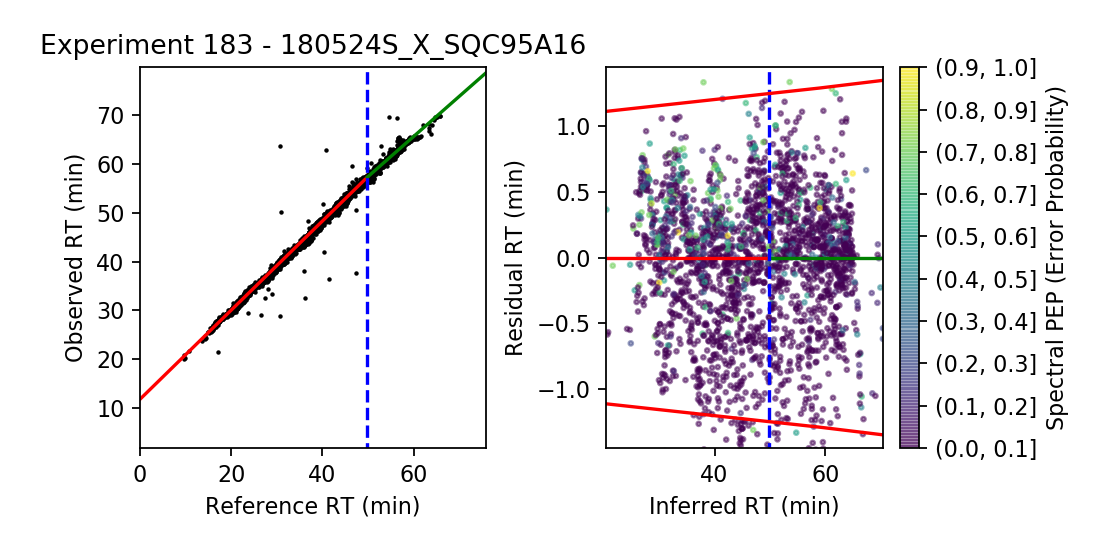

Supplement: S1 File — A optional HTML report generated by the dart_id Python script. The report gives a summary of the alignment for each experiment, as well as a broad overview of the performance of the run as a whole, by showing aggregate increases in PSMs at a chosen confidence threshold. (ZIP) [file pcbi.1007082.s001.zip › DART-ID_SCoPE-MS_Report/figures/alignment_183_180524S_X_SQC95A16.png]

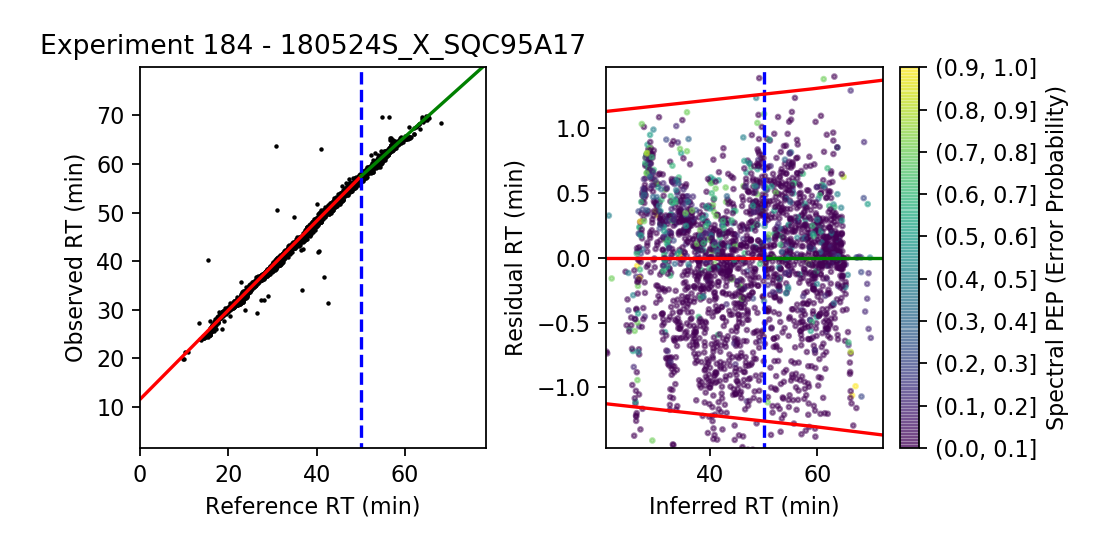

Supplement: S1 File — A optional HTML report generated by the dart_id Python script. The report gives a summary of the alignment for each experiment, as well as a broad overview of the performance of the run as a whole, by showing aggregate increases in PSMs at a chosen confidence threshold. (ZIP) [file pcbi.1007082.s001.zip › DART-ID_SCoPE-MS_Report/figures/alignment_184_180524S_X_SQC95A17.png]

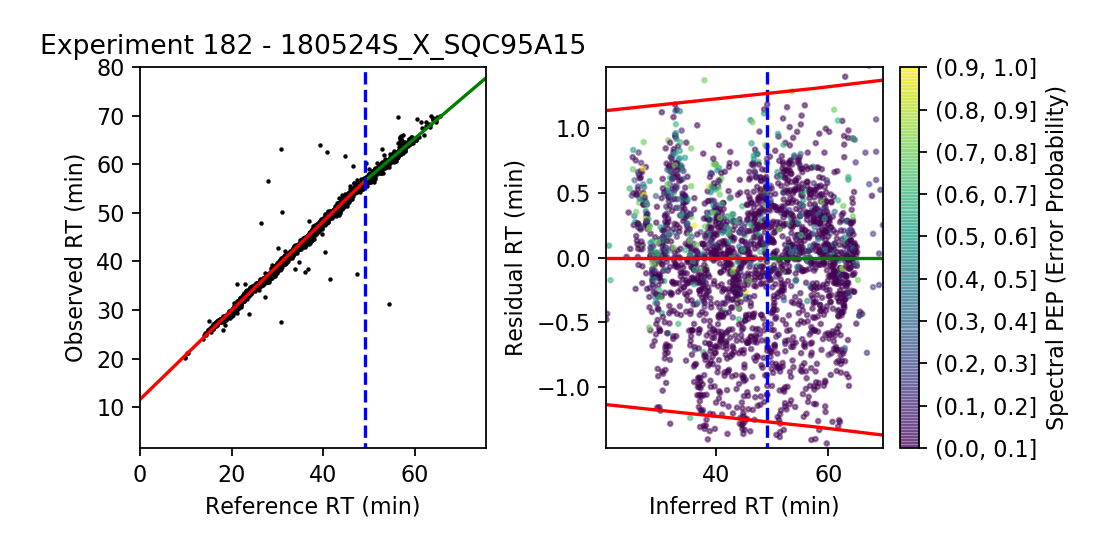

Supplement: S1 File — A optional HTML report generated by the dart_id Python script. The report gives a summary of the alignment for each experiment, as well as a broad overview of the performance of the run as a whole, by showing aggregate increases in PSMs at a chosen confidence threshold. (ZIP) [file pcbi.1007082.s001.zip › DART-ID_SCoPE-MS_Report/figures/alignment_182_180524S_X_SQC95A15.png]

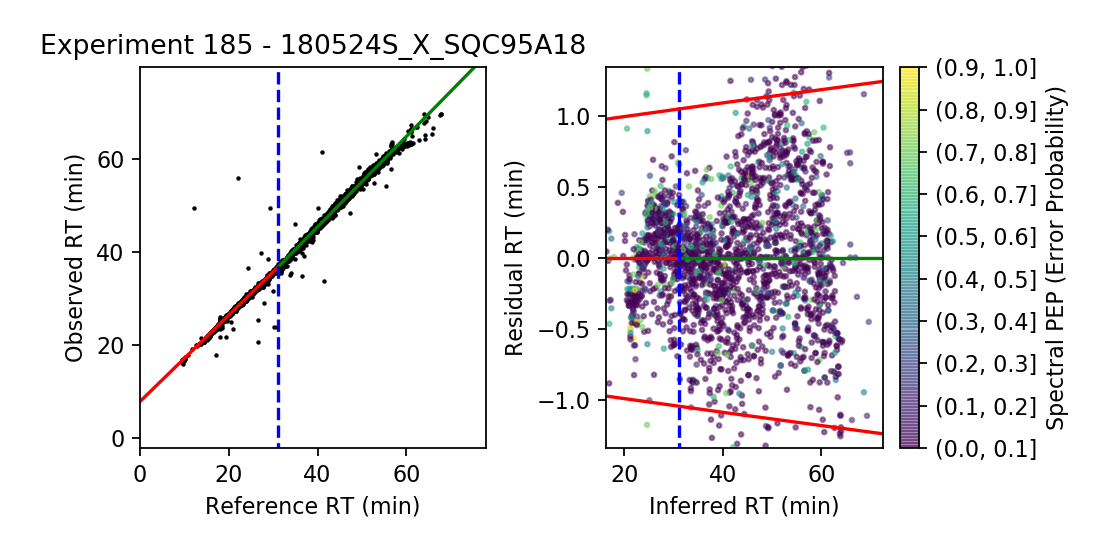

Supplement: S1 File — A optional HTML report generated by the dart_id Python script. The report gives a summary of the alignment for each experiment, as well as a broad overview of the performance of the run as a whole, by showing aggregate increases in PSMs at a chosen confidence threshold. (ZIP) [file pcbi.1007082.s001.zip › DART-ID_SCoPE-MS_Report/figures/alignment_185_180524S_X_SQC95A18.png]

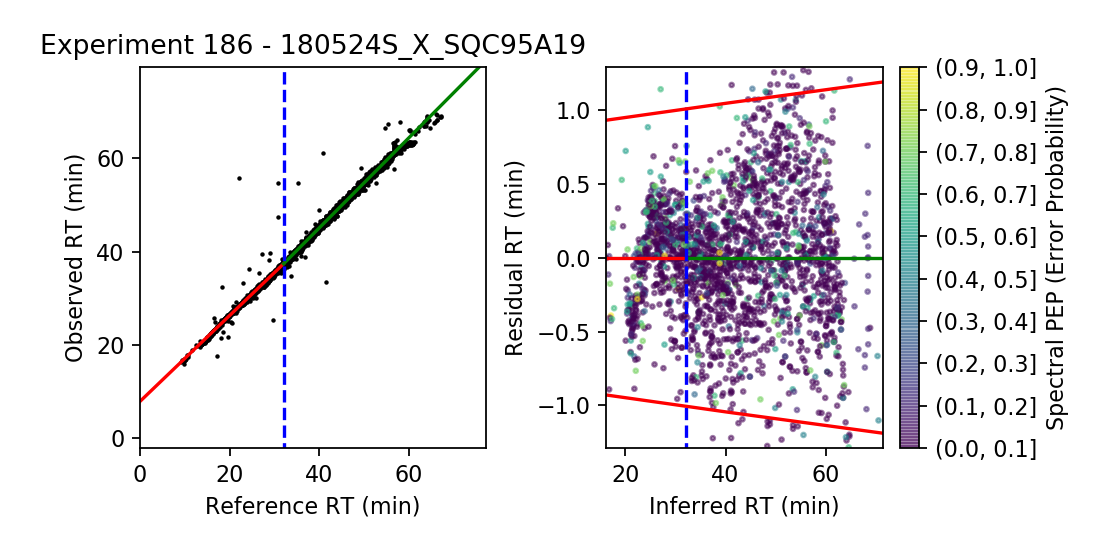

Supplement: S1 File — A optional HTML report generated by the dart_id Python script. The report gives a summary of the alignment for each experiment, as well as a broad overview of the performance of the run as a whole, by showing aggregate increases in PSMs at a chosen confidence threshold. (ZIP) [file pcbi.1007082.s001.zip › DART-ID_SCoPE-MS_Report/figures/alignment_186_180524S_X_SQC95A19.png]

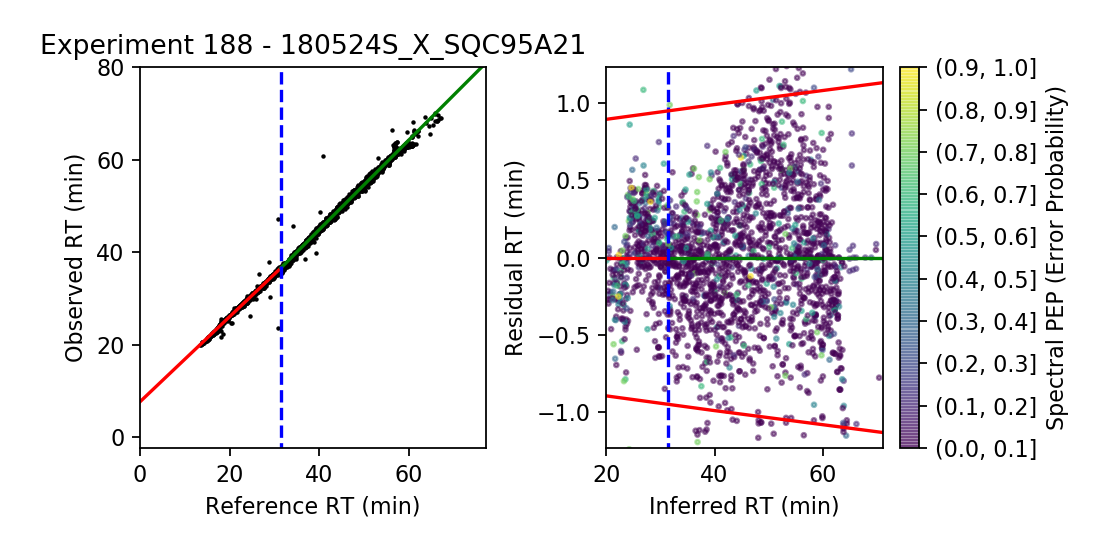

Supplement: S1 File — A optional HTML report generated by the dart_id Python script. The report gives a summary of the alignment for each experiment, as well as a broad overview of the performance of the run as a whole, by showing aggregate increases in PSMs at a chosen confidence threshold. (ZIP) [file pcbi.1007082.s001.zip › DART-ID_SCoPE-MS_Report/figures/alignment_188_180524S_X_SQC95A21.png]

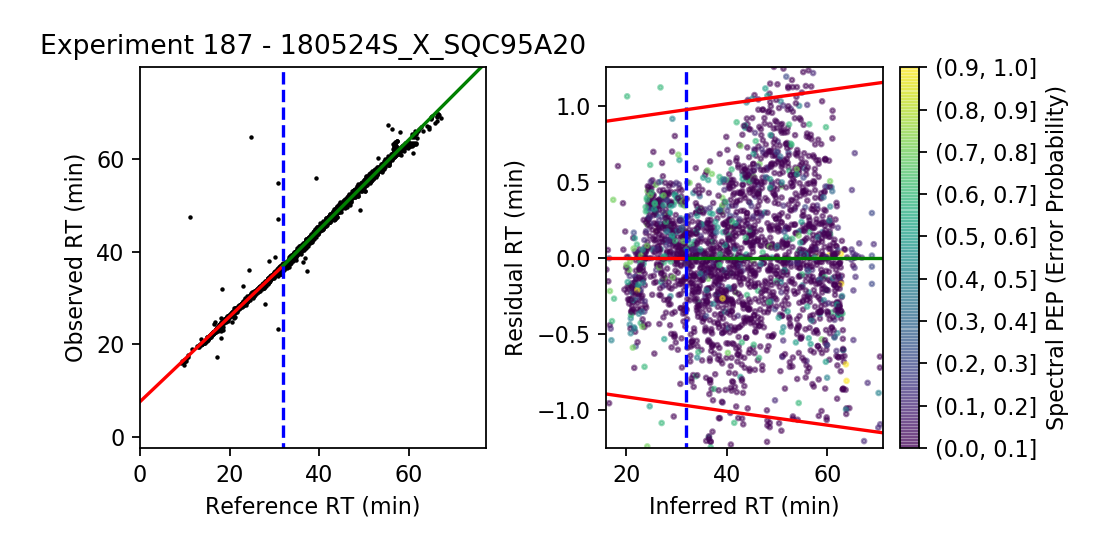

Supplement: S1 File — A optional HTML report generated by the dart_id Python script. The report gives a summary of the alignment for each experiment, as well as a broad overview of the performance of the run as a whole, by showing aggregate increases in PSMs at a chosen confidence threshold. (ZIP) [file pcbi.1007082.s001.zip › DART-ID_SCoPE-MS_Report/figures/alignment_187_180524S_X_SQC95A20.png]

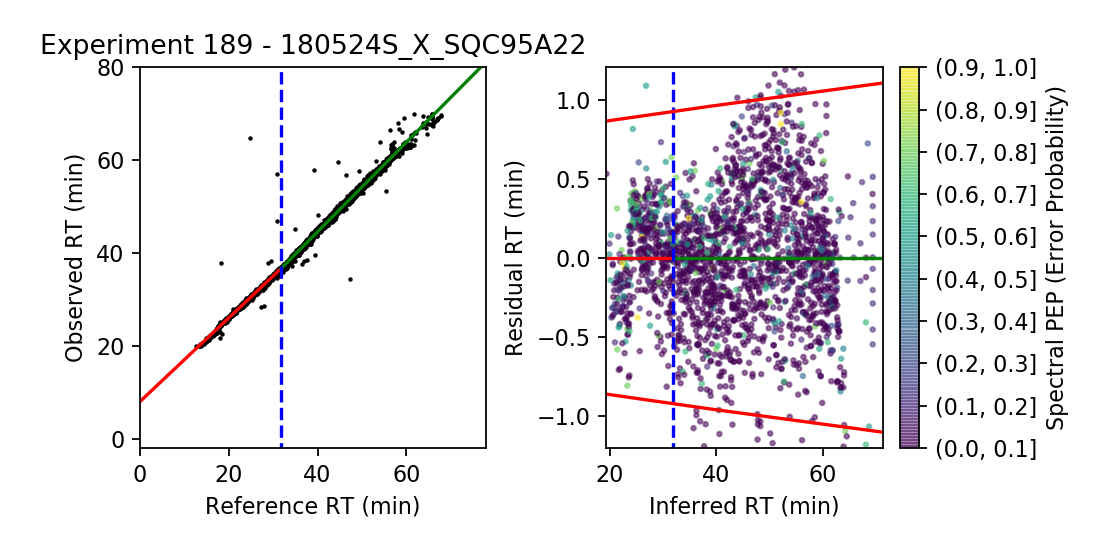

Supplement: S1 File — A optional HTML report generated by the dart_id Python script. The report gives a summary of the alignment for each experiment, as well as a broad overview of the performance of the run as a whole, by showing aggregate increases in PSMs at a chosen confidence threshold. (ZIP) [file pcbi.1007082.s001.zip › DART-ID_SCoPE-MS_Report/figures/alignment_189_180524S_X_SQC95A22.png]

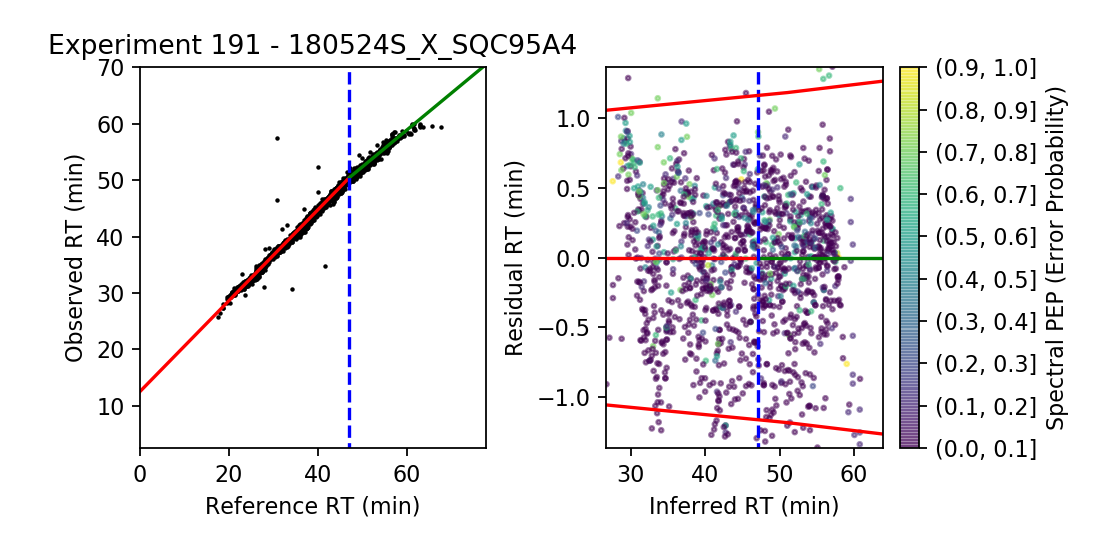

Supplement: S1 File — A optional HTML report generated by the dart_id Python script. The report gives a summary of the alignment for each experiment, as well as a broad overview of the performance of the run as a whole, by showing aggregate increases in PSMs at a chosen confidence threshold. (ZIP) [file pcbi.1007082.s001.zip › DART-ID_SCoPE-MS_Report/figures/alignment_191_180524S_X_SQC95A4.png]

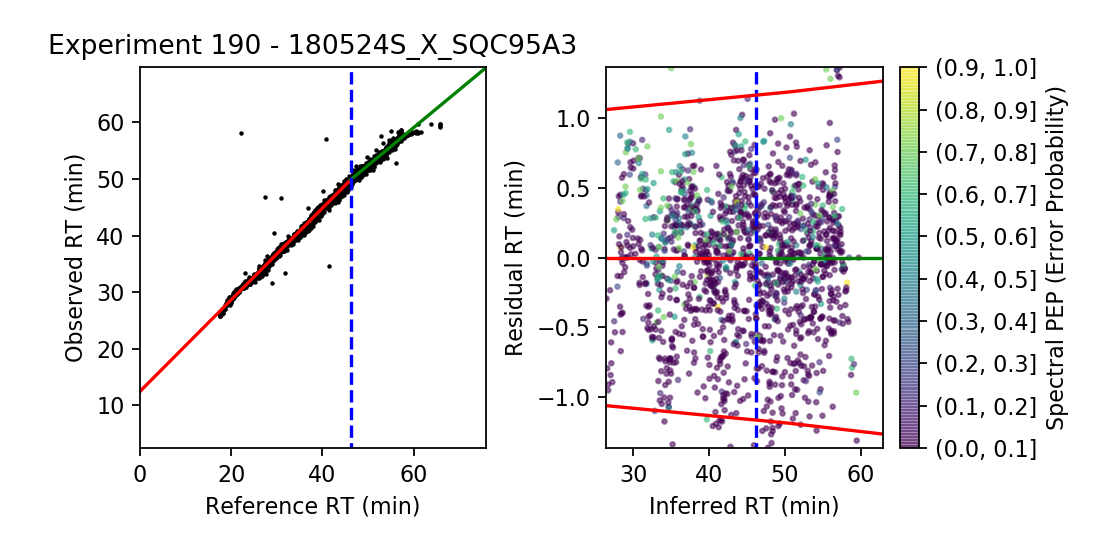

Supplement: S1 File — A optional HTML report generated by the dart_id Python script. The report gives a summary of the alignment for each experiment, as well as a broad overview of the performance of the run as a whole, by showing aggregate increases in PSMs at a chosen confidence threshold. (ZIP) [file pcbi.1007082.s001.zip › DART-ID_SCoPE-MS_Report/figures/alignment_190_180524S_X_SQC95A3.png]

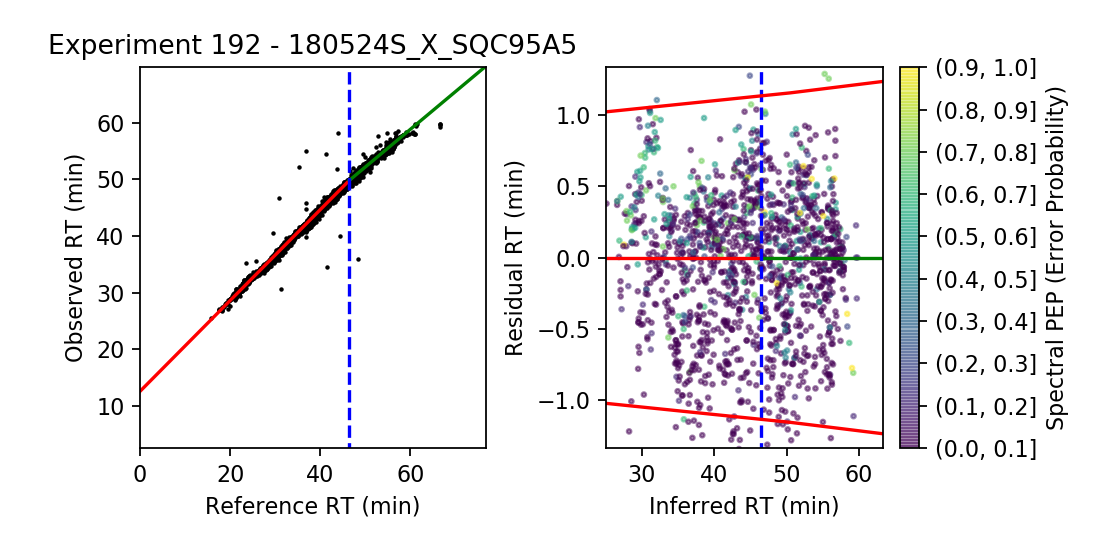

Supplement: S1 File — A optional HTML report generated by the dart_id Python script. The report gives a summary of the alignment for each experiment, as well as a broad overview of the performance of the run as a whole, by showing aggregate increases in PSMs at a chosen confidence threshold. (ZIP) [file pcbi.1007082.s001.zip › DART-ID_SCoPE-MS_Report/figures/alignment_192_180524S_X_SQC95A5.png]

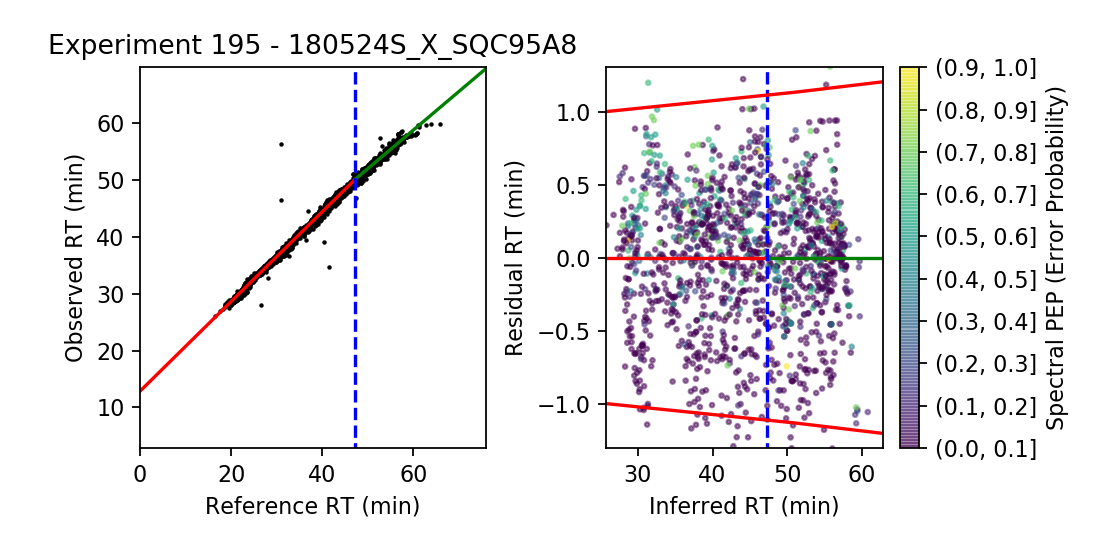

Supplement: S1 File — A optional HTML report generated by the dart_id Python script. The report gives a summary of the alignment for each experiment, as well as a broad overview of the performance of the run as a whole, by showing aggregate increases in PSMs at a chosen confidence threshold. (ZIP) [file pcbi.1007082.s001.zip › DART-ID_SCoPE-MS_Report/figures/alignment_195_180524S_X_SQC95A8.png]

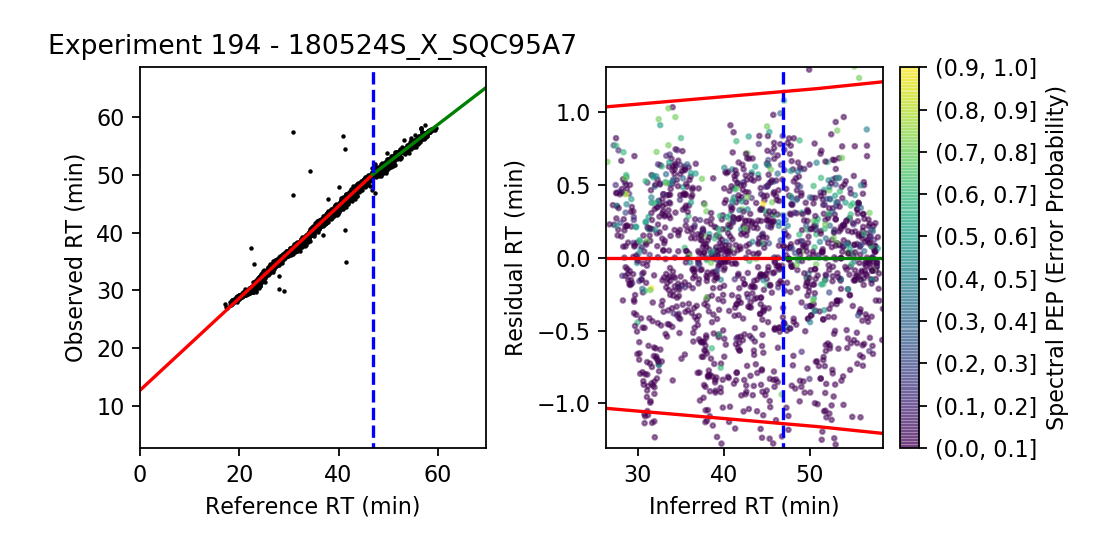

Supplement: S1 File — A optional HTML report generated by the dart_id Python script. The report gives a summary of the alignment for each experiment, as well as a broad overview of the performance of the run as a whole, by showing aggregate increases in PSMs at a chosen confidence threshold. (ZIP) [file pcbi.1007082.s001.zip › DART-ID_SCoPE-MS_Report/figures/alignment_194_180524S_X_SQC95A7.png]

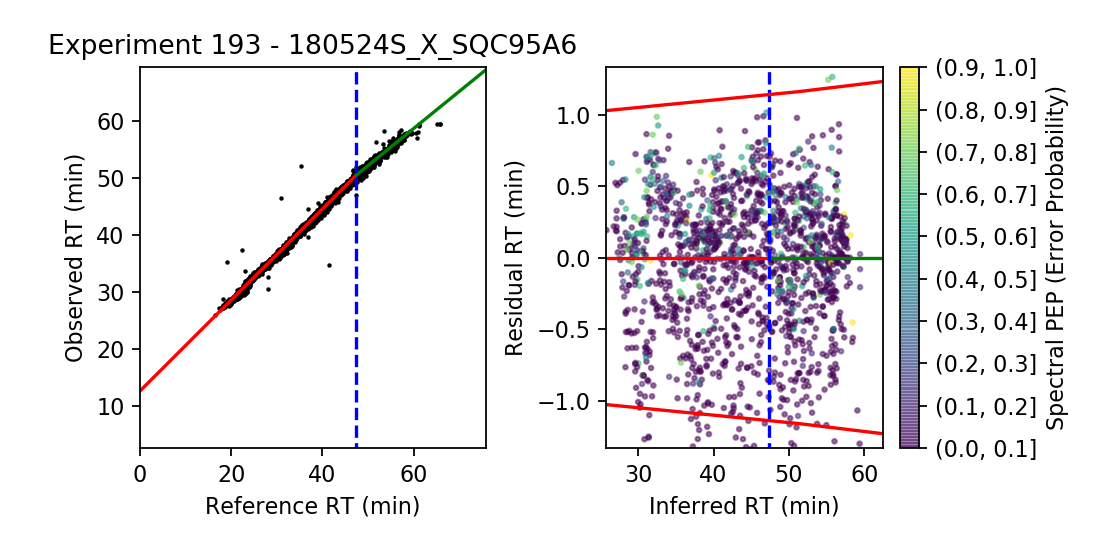

Supplement: S1 File — A optional HTML report generated by the dart_id Python script. The report gives a summary of the alignment for each experiment, as well as a broad overview of the performance of the run as a whole, by showing aggregate increases in PSMs at a chosen confidence threshold. (ZIP) [file pcbi.1007082.s001.zip › DART-ID_SCoPE-MS_Report/figures/alignment_193_180524S_X_SQC95A6.png]

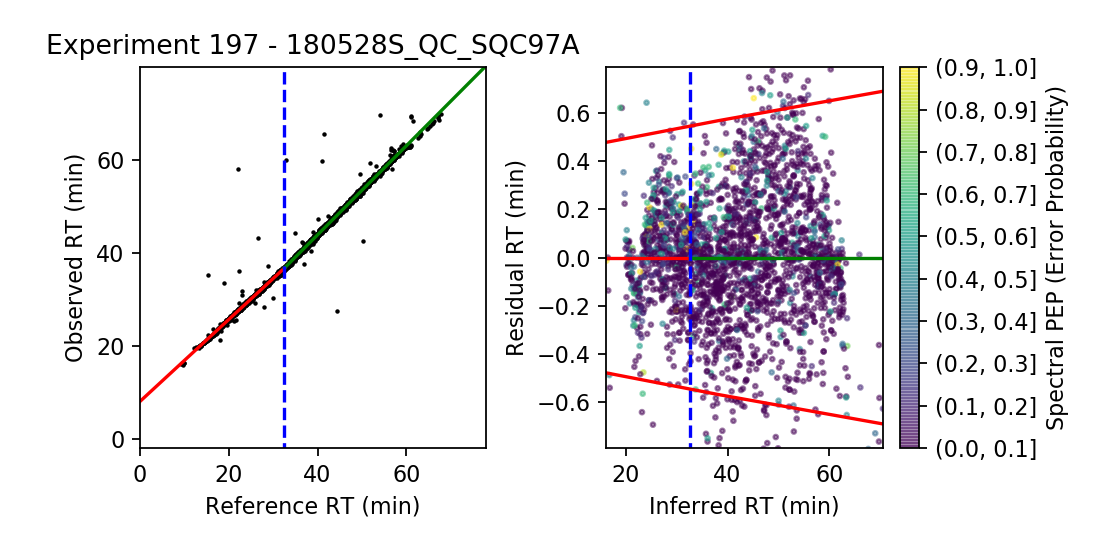

Supplement: S1 File — A optional HTML report generated by the dart_id Python script. The report gives a summary of the alignment for each experiment, as well as a broad overview of the performance of the run as a whole, by showing aggregate increases in PSMs at a chosen confidence threshold. (ZIP) [file pcbi.1007082.s001.zip › DART-ID_SCoPE-MS_Report/figures/alignment_197_180528S_QC_SQC97A.png]

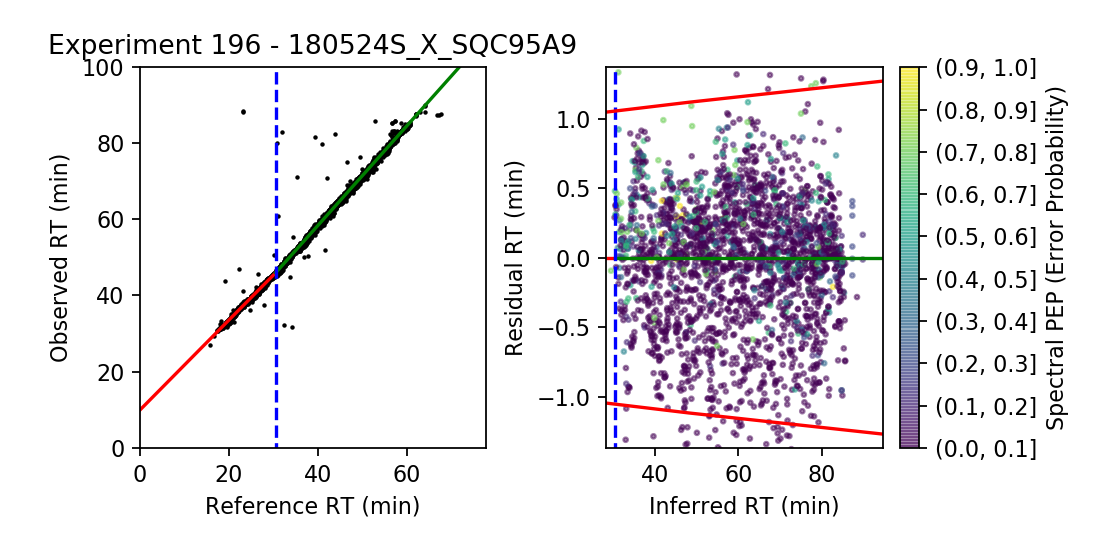

Supplement: S1 File — A optional HTML report generated by the dart_id Python script. The report gives a summary of the alignment for each experiment, as well as a broad overview of the performance of the run as a whole, by showing aggregate increases in PSMs at a chosen confidence threshold. (ZIP) [file pcbi.1007082.s001.zip › DART-ID_SCoPE-MS_Report/figures/alignment_196_180524S_X_SQC95A9.png]

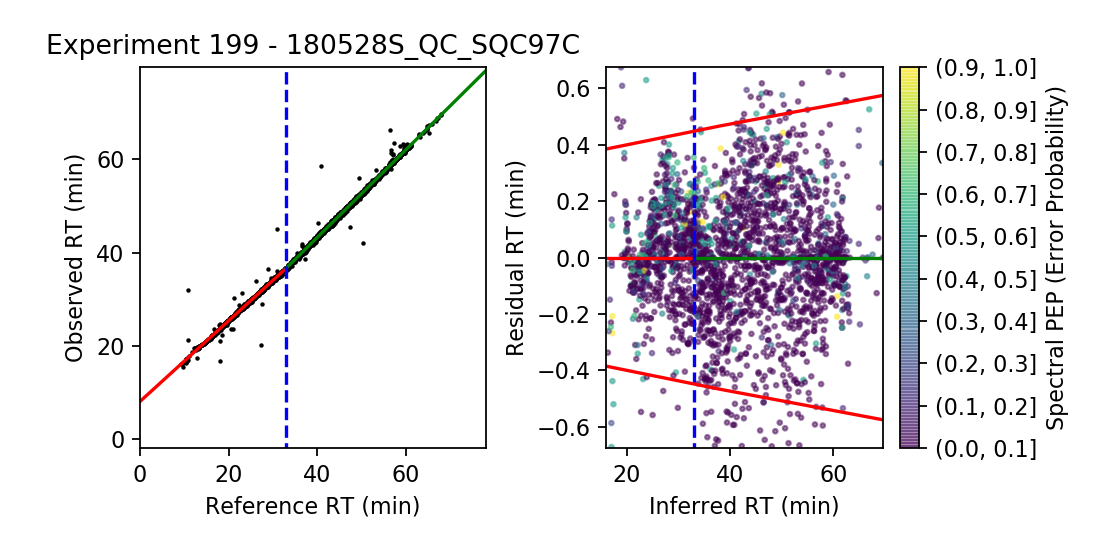

Supplement: S1 File — A optional HTML report generated by the dart_id Python script. The report gives a summary of the alignment for each experiment, as well as a broad overview of the performance of the run as a whole, by showing aggregate increases in PSMs at a chosen confidence threshold. (ZIP) [file pcbi.1007082.s001.zip › DART-ID_SCoPE-MS_Report/figures/alignment_199_180528S_QC_SQC97C.png]

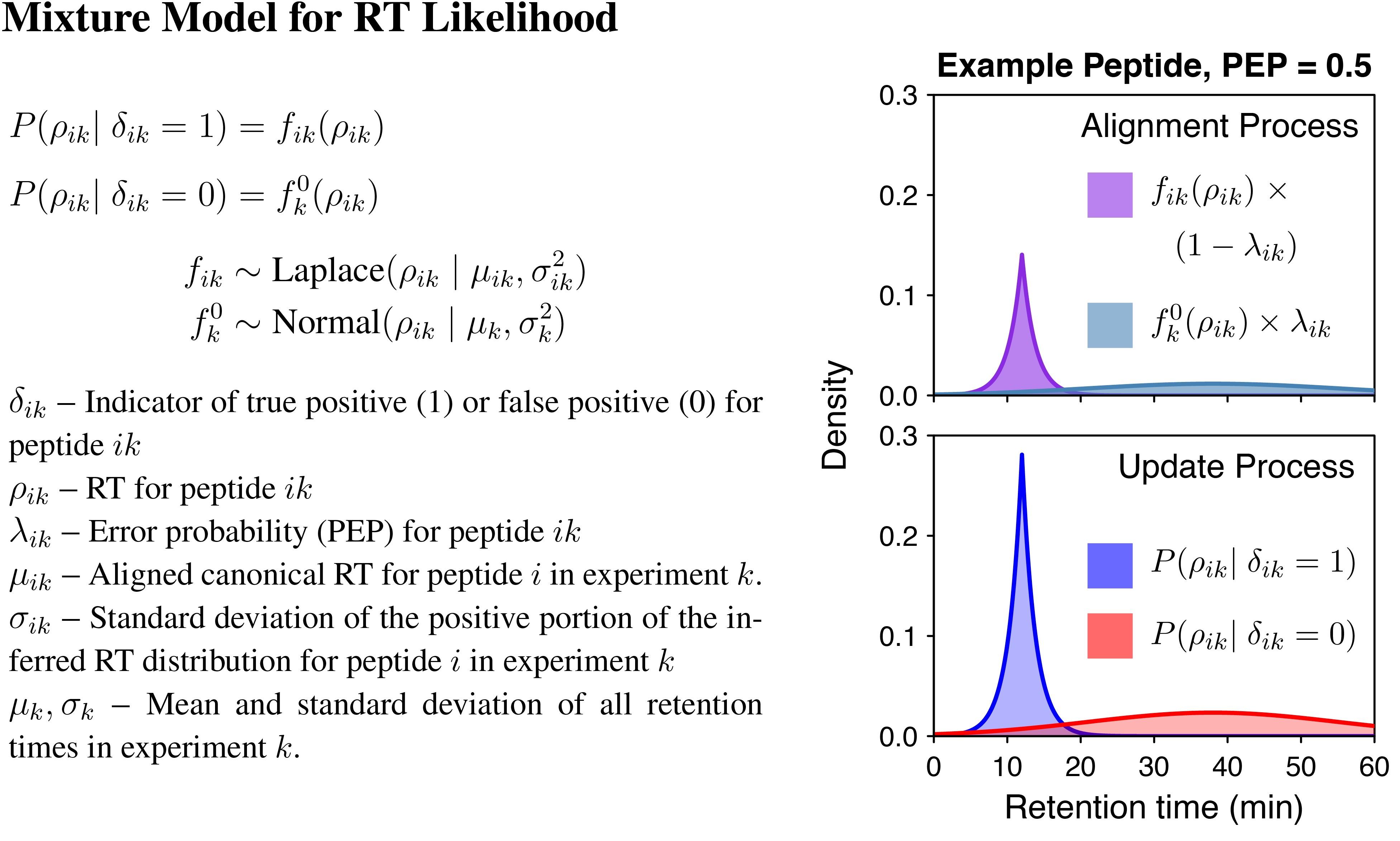

Supplement: S1 Fig — In the global alignment process, the likelihood of the alignment function and the reference RT is estimated from a mixture model, which combines the two possibilities of whether the peptide is assigned the correct or incorrect peptide sequence. These two distributions are then weighted by the error probability (PEP). This is similar to the update process, which updates the error probability and incorporates the previous error probability, as well as the two conditional probability distributions. (TIF) [file pcbi.1007082.s004.tif]
