# Supplementary figures and images for: DART-ID increases single-cell proteome coverage (part 1 of 3)
Source: PLoS Comput Biol. 2019 Jul 1;15(7):e1007082. doi: 10.1371/journal.pcbi.1007082 (PMC6625733; doi:10.1371/journal.pcbi.1007082)

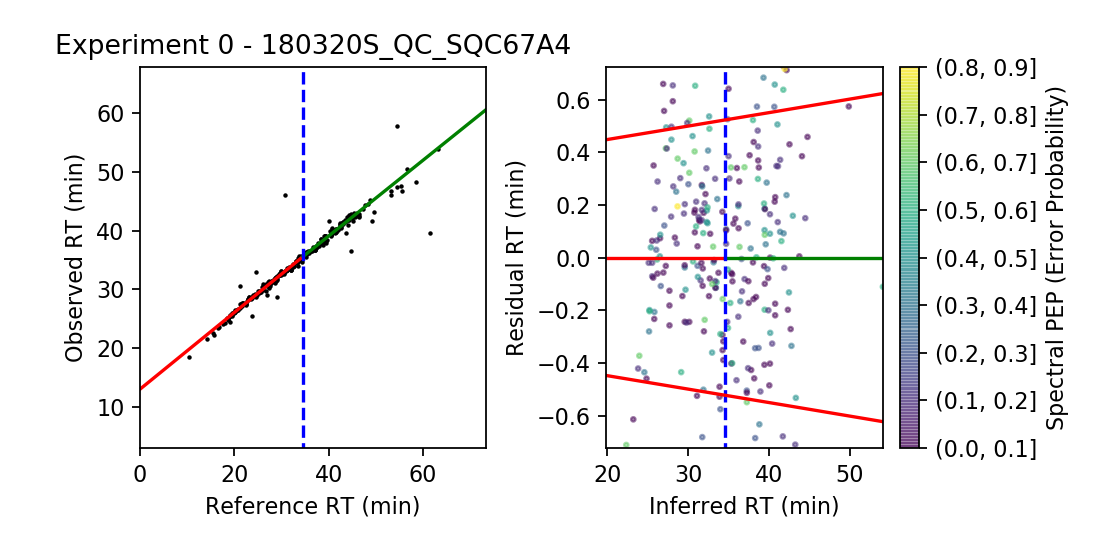

Supplement: S1 File — A optional HTML report generated by the dart_id Python script. The report gives a summary of the alignment for each experiment, as well as a broad overview of the performance of the run as a whole, by showing aggregate increases in PSMs at a chosen confidence threshold. (ZIP) [file pcbi.1007082.s001.zip › DART-ID_SCoPE-MS_Report/figures/alignment_0_180320S_QC_SQC67A4.png]

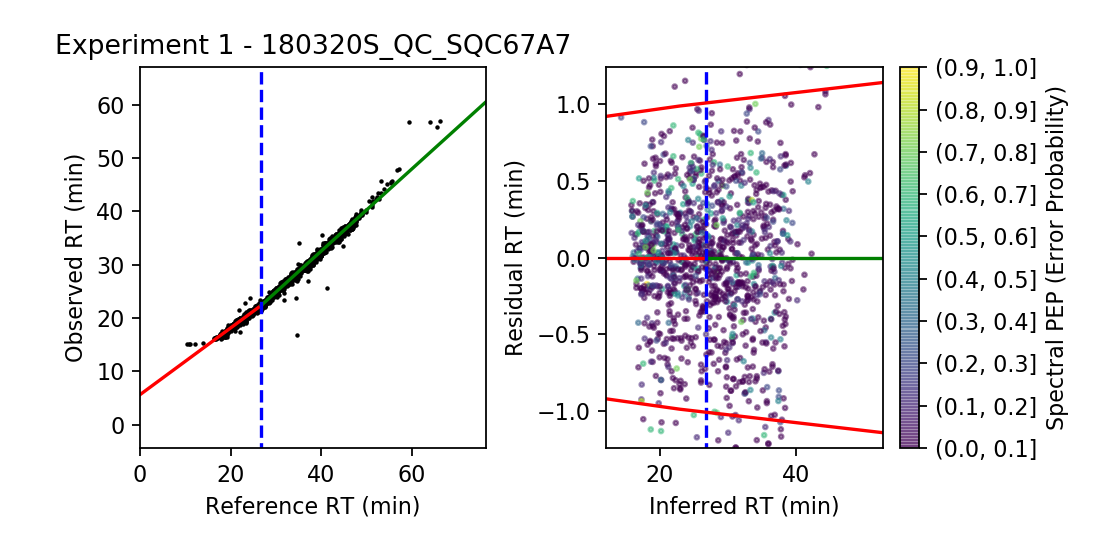

Supplement: S1 File — A optional HTML report generated by the dart_id Python script. The report gives a summary of the alignment for each experiment, as well as a broad overview of the performance of the run as a whole, by showing aggregate increases in PSMs at a chosen confidence threshold. (ZIP) [file pcbi.1007082.s001.zip › DART-ID_SCoPE-MS_Report/figures/alignment_1_180320S_QC_SQC67A7.png]

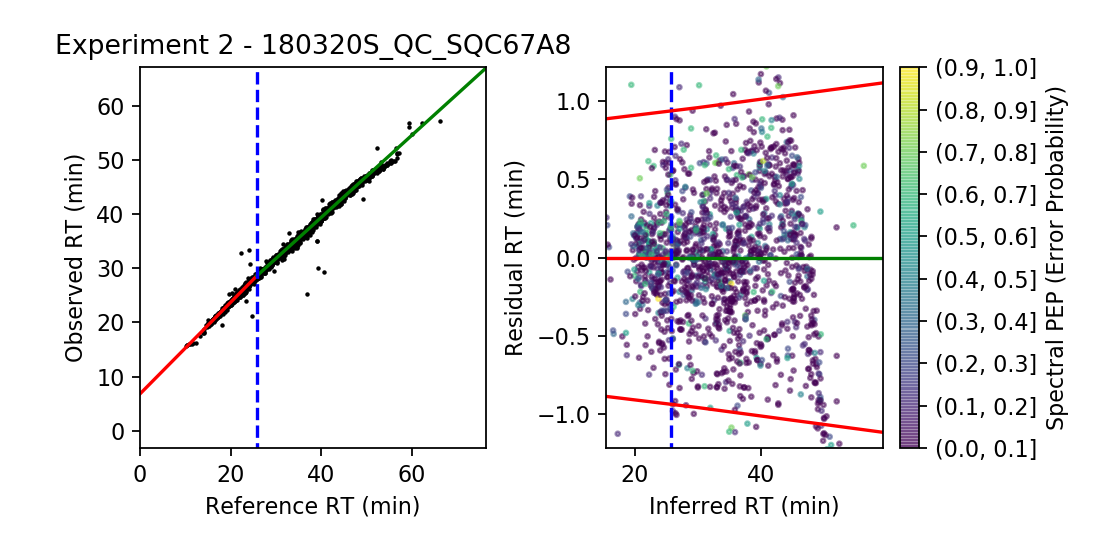

Supplement: S1 File — A optional HTML report generated by the dart_id Python script. The report gives a summary of the alignment for each experiment, as well as a broad overview of the performance of the run as a whole, by showing aggregate increases in PSMs at a chosen confidence threshold. (ZIP) [file pcbi.1007082.s001.zip › DART-ID_SCoPE-MS_Report/figures/alignment_2_180320S_QC_SQC67A8.png]

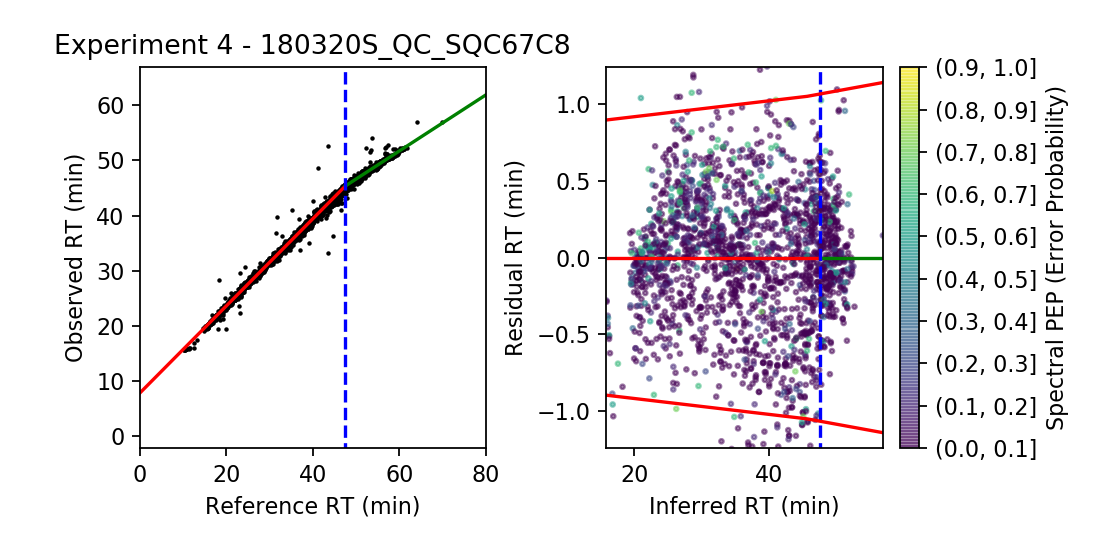

Supplement: S1 File — A optional HTML report generated by the dart_id Python script. The report gives a summary of the alignment for each experiment, as well as a broad overview of the performance of the run as a whole, by showing aggregate increases in PSMs at a chosen confidence threshold. (ZIP) [file pcbi.1007082.s001.zip › DART-ID_SCoPE-MS_Report/figures/alignment_4_180320S_QC_SQC67C8.png]

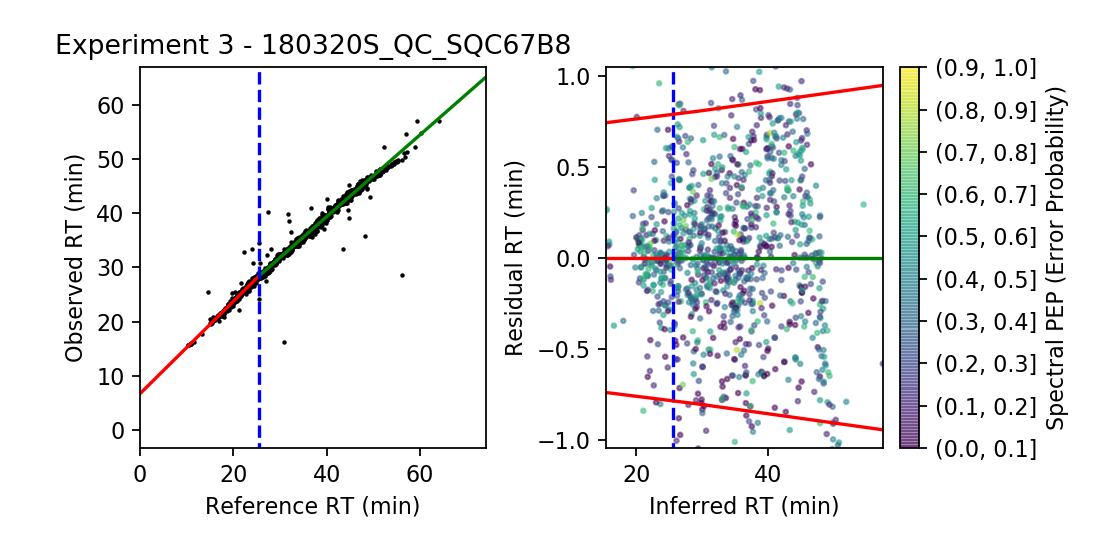

Supplement: S1 File — A optional HTML report generated by the dart_id Python script. The report gives a summary of the alignment for each experiment, as well as a broad overview of the performance of the run as a whole, by showing aggregate increases in PSMs at a chosen confidence threshold. (ZIP) [file pcbi.1007082.s001.zip › DART-ID_SCoPE-MS_Report/figures/alignment_3_180320S_QC_SQC67B8.png]

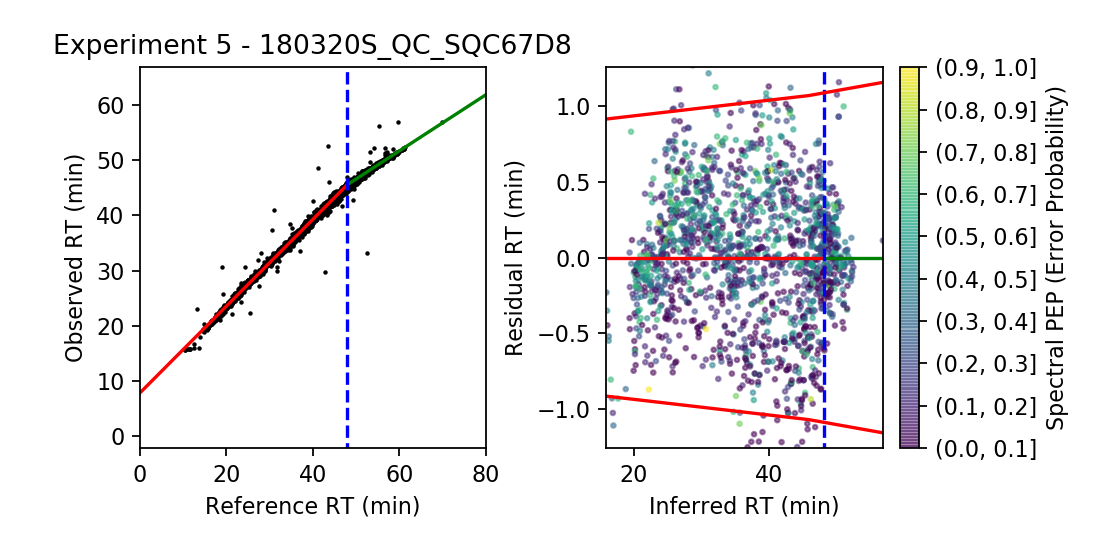

Supplement: S1 File — A optional HTML report generated by the dart_id Python script. The report gives a summary of the alignment for each experiment, as well as a broad overview of the performance of the run as a whole, by showing aggregate increases in PSMs at a chosen confidence threshold. (ZIP) [file pcbi.1007082.s001.zip › DART-ID_SCoPE-MS_Report/figures/alignment_5_180320S_QC_SQC67D8.png]

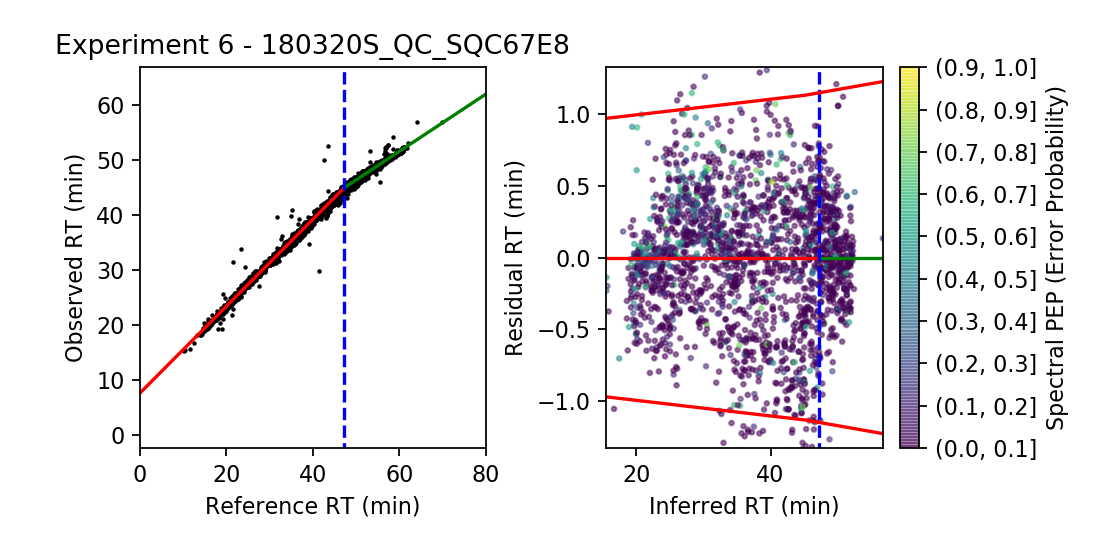

Supplement: S1 File — A optional HTML report generated by the dart_id Python script. The report gives a summary of the alignment for each experiment, as well as a broad overview of the performance of the run as a whole, by showing aggregate increases in PSMs at a chosen confidence threshold. (ZIP) [file pcbi.1007082.s001.zip › DART-ID_SCoPE-MS_Report/figures/alignment_6_180320S_QC_SQC67E8.png]

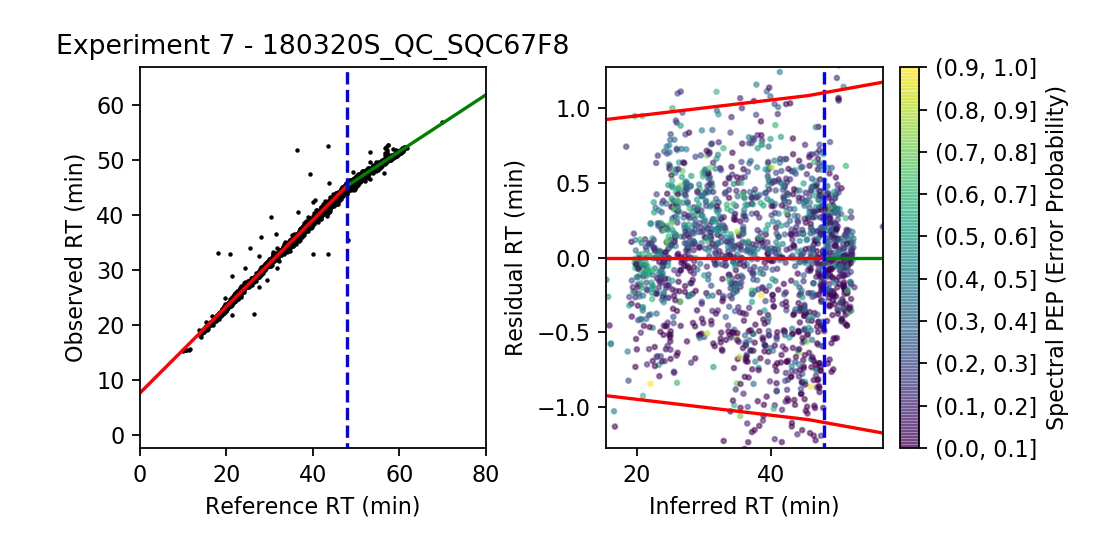

Supplement: S1 File — A optional HTML report generated by the dart_id Python script. The report gives a summary of the alignment for each experiment, as well as a broad overview of the performance of the run as a whole, by showing aggregate increases in PSMs at a chosen confidence threshold. (ZIP) [file pcbi.1007082.s001.zip › DART-ID_SCoPE-MS_Report/figures/alignment_7_180320S_QC_SQC67F8.png]

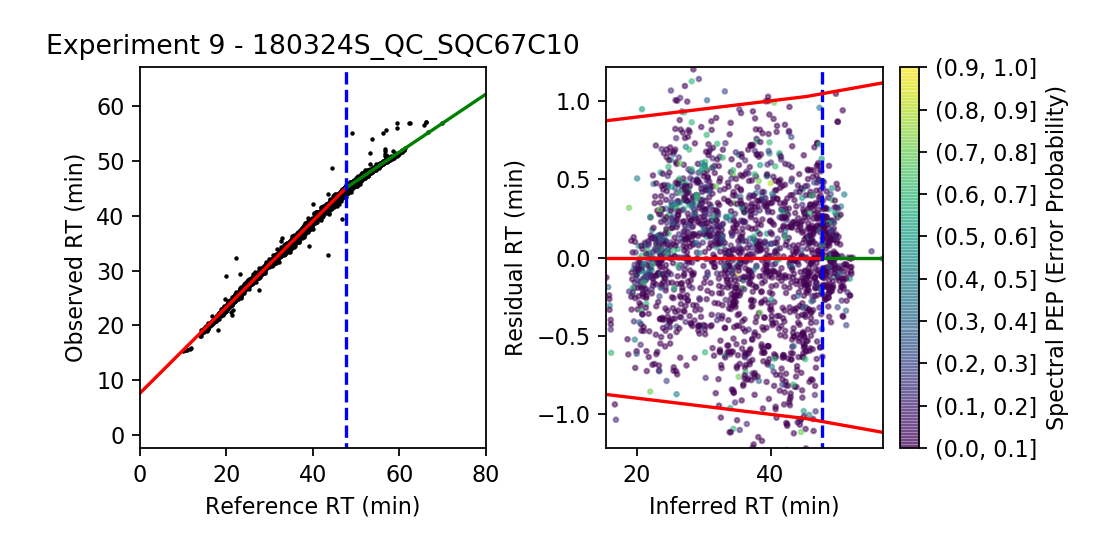

Supplement: S1 File — A optional HTML report generated by the dart_id Python script. The report gives a summary of the alignment for each experiment, as well as a broad overview of the performance of the run as a whole, by showing aggregate increases in PSMs at a chosen confidence threshold. (ZIP) [file pcbi.1007082.s001.zip › DART-ID_SCoPE-MS_Report/figures/alignment_9_180324S_QC_SQC67C10.png]

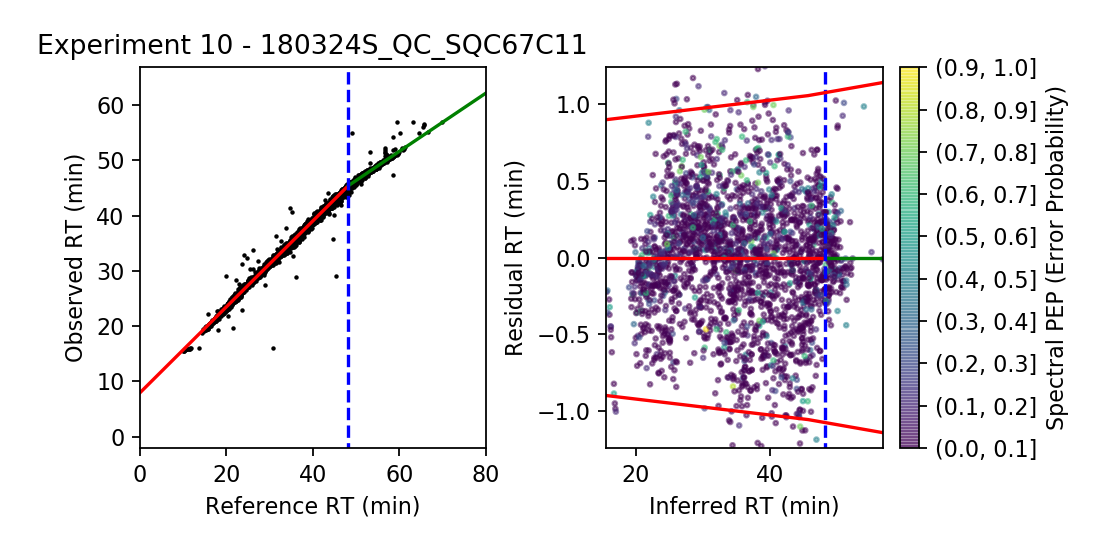

Supplement: S1 File — A optional HTML report generated by the dart_id Python script. The report gives a summary of the alignment for each experiment, as well as a broad overview of the performance of the run as a whole, by showing aggregate increases in PSMs at a chosen confidence threshold. (ZIP) [file pcbi.1007082.s001.zip › DART-ID_SCoPE-MS_Report/figures/alignment_10_180324S_QC_SQC67C11.png]

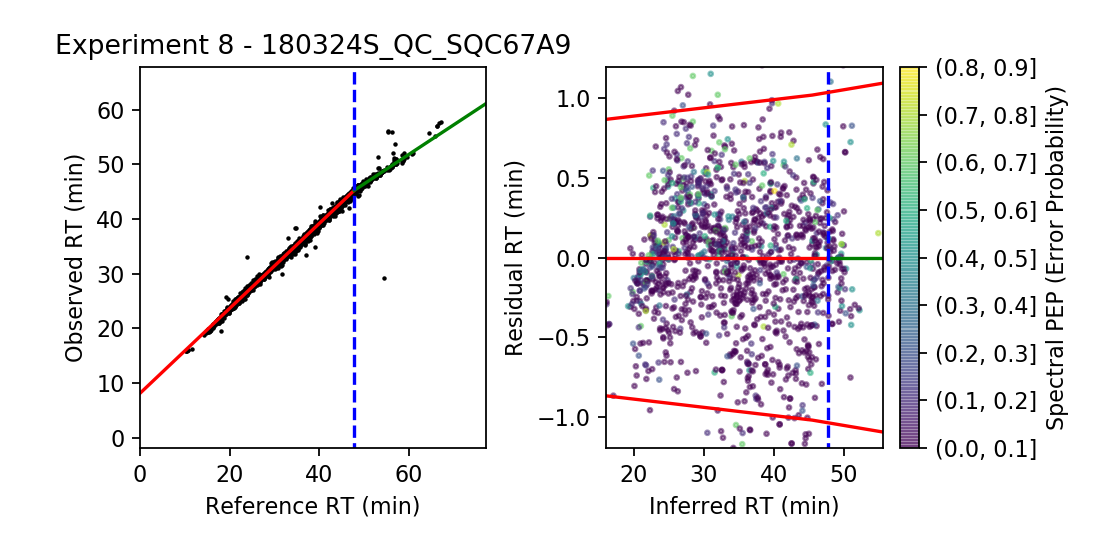

Supplement: S1 File — A optional HTML report generated by the dart_id Python script. The report gives a summary of the alignment for each experiment, as well as a broad overview of the performance of the run as a whole, by showing aggregate increases in PSMs at a chosen confidence threshold. (ZIP) [file pcbi.1007082.s001.zip › DART-ID_SCoPE-MS_Report/figures/alignment_8_180324S_QC_SQC67A9.png]

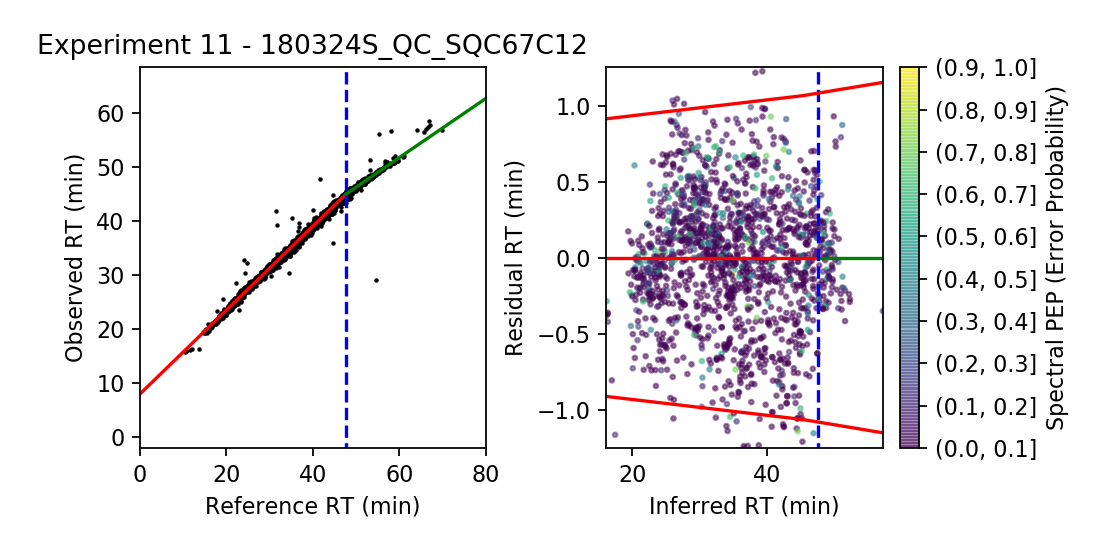

Supplement: S1 File — A optional HTML report generated by the dart_id Python script. The report gives a summary of the alignment for each experiment, as well as a broad overview of the performance of the run as a whole, by showing aggregate increases in PSMs at a chosen confidence threshold. (ZIP) [file pcbi.1007082.s001.zip › DART-ID_SCoPE-MS_Report/figures/alignment_11_180324S_QC_SQC67C12.png]

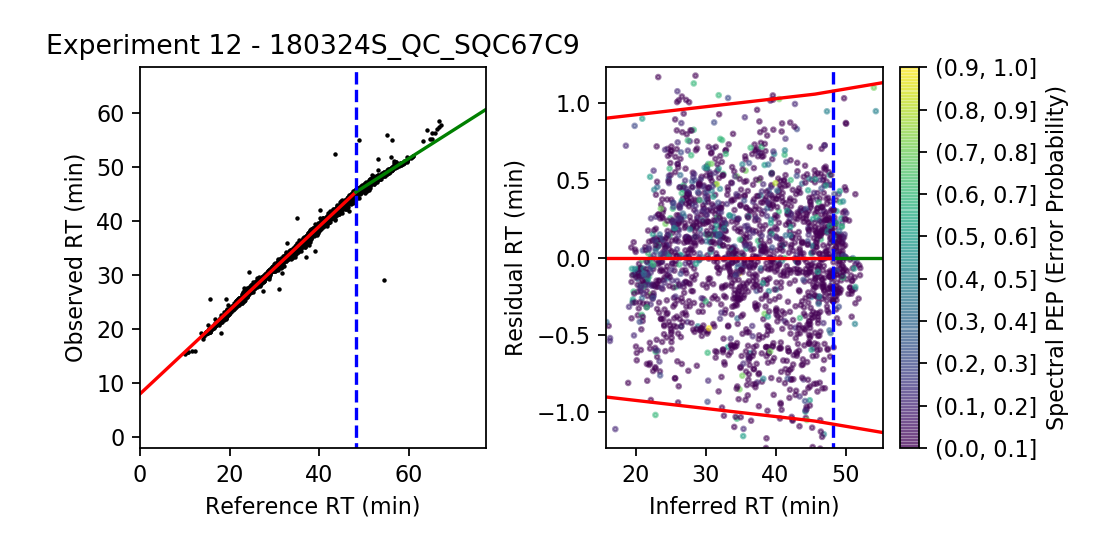

Supplement: S1 File — A optional HTML report generated by the dart_id Python script. The report gives a summary of the alignment for each experiment, as well as a broad overview of the performance of the run as a whole, by showing aggregate increases in PSMs at a chosen confidence threshold. (ZIP) [file pcbi.1007082.s001.zip › DART-ID_SCoPE-MS_Report/figures/alignment_12_180324S_QC_SQC67C9.png]

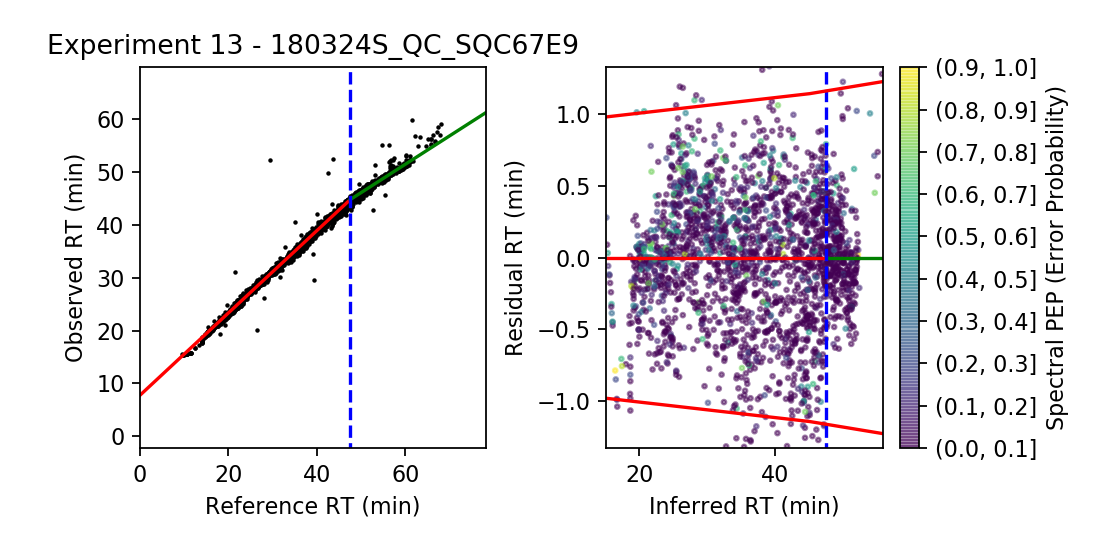

Supplement: S1 File — A optional HTML report generated by the dart_id Python script. The report gives a summary of the alignment for each experiment, as well as a broad overview of the performance of the run as a whole, by showing aggregate increases in PSMs at a chosen confidence threshold. (ZIP) [file pcbi.1007082.s001.zip › DART-ID_SCoPE-MS_Report/figures/alignment_13_180324S_QC_SQC67E9.png]

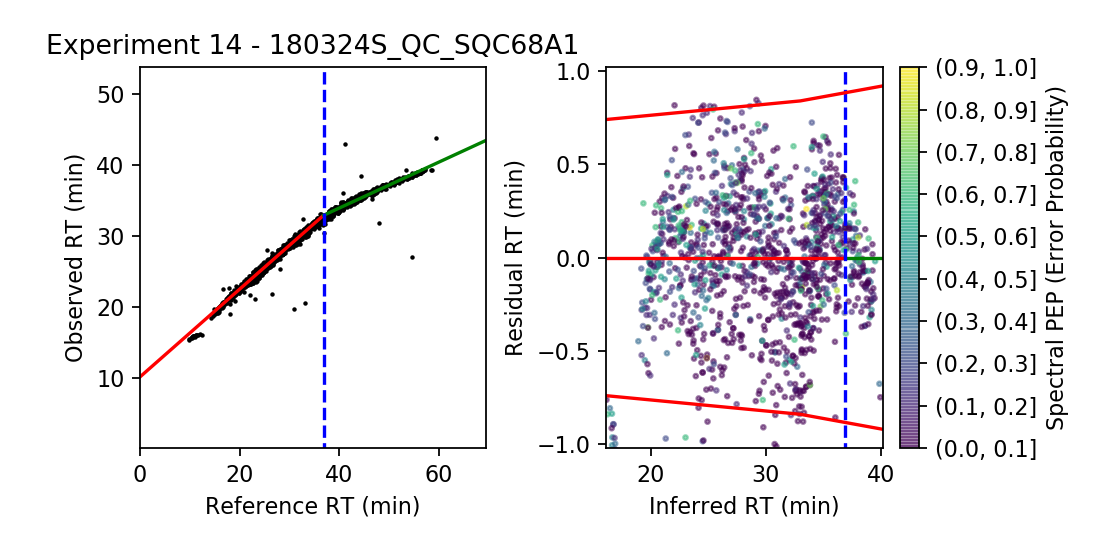

Supplement: S1 File — A optional HTML report generated by the dart_id Python script. The report gives a summary of the alignment for each experiment, as well as a broad overview of the performance of the run as a whole, by showing aggregate increases in PSMs at a chosen confidence threshold. (ZIP) [file pcbi.1007082.s001.zip › DART-ID_SCoPE-MS_Report/figures/alignment_14_180324S_QC_SQC68A1.png]

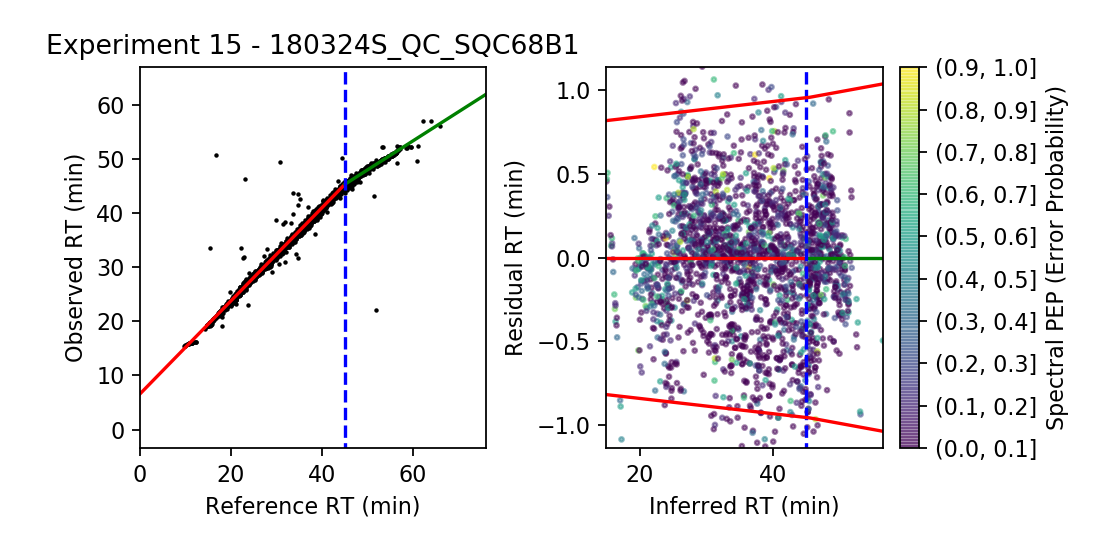

Supplement: S1 File — A optional HTML report generated by the dart_id Python script. The report gives a summary of the alignment for each experiment, as well as a broad overview of the performance of the run as a whole, by showing aggregate increases in PSMs at a chosen confidence threshold. (ZIP) [file pcbi.1007082.s001.zip › DART-ID_SCoPE-MS_Report/figures/alignment_15_180324S_QC_SQC68B1.png]

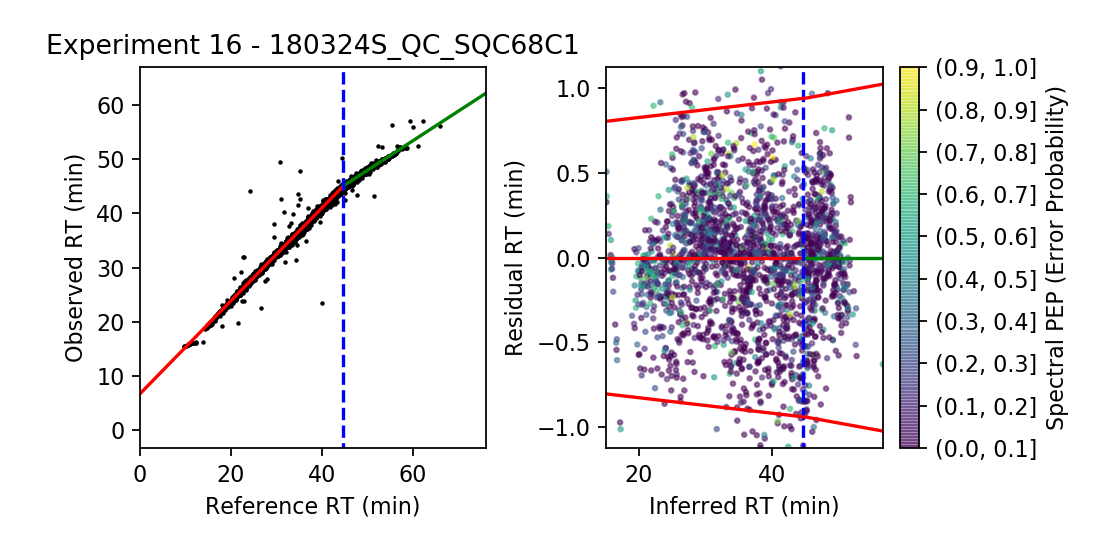

Supplement: S1 File — A optional HTML report generated by the dart_id Python script. The report gives a summary of the alignment for each experiment, as well as a broad overview of the performance of the run as a whole, by showing aggregate increases in PSMs at a chosen confidence threshold. (ZIP) [file pcbi.1007082.s001.zip › DART-ID_SCoPE-MS_Report/figures/alignment_16_180324S_QC_SQC68C1.png]

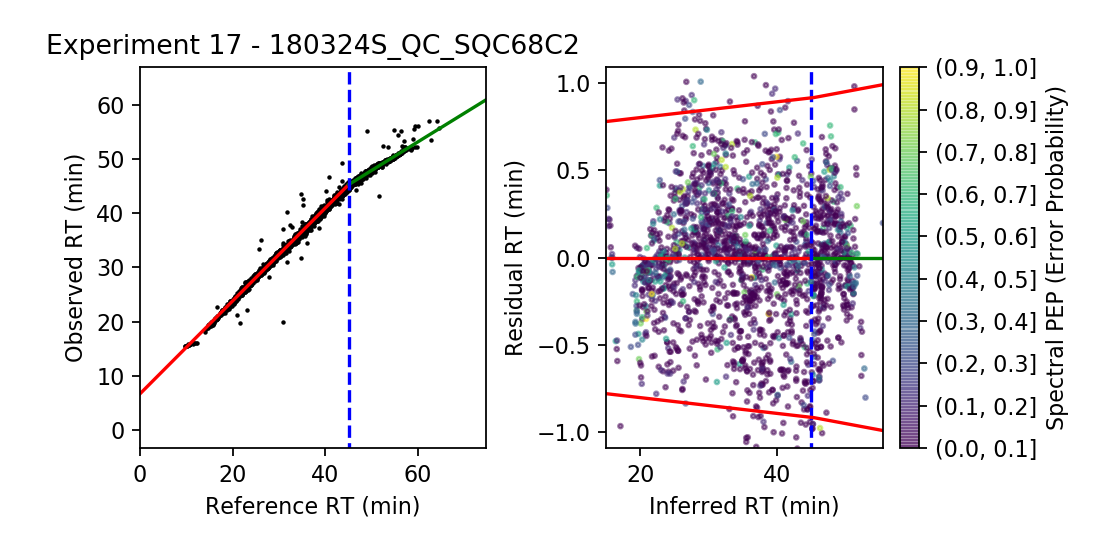

Supplement: S1 File — A optional HTML report generated by the dart_id Python script. The report gives a summary of the alignment for each experiment, as well as a broad overview of the performance of the run as a whole, by showing aggregate increases in PSMs at a chosen confidence threshold. (ZIP) [file pcbi.1007082.s001.zip › DART-ID_SCoPE-MS_Report/figures/alignment_17_180324S_QC_SQC68C2.png]

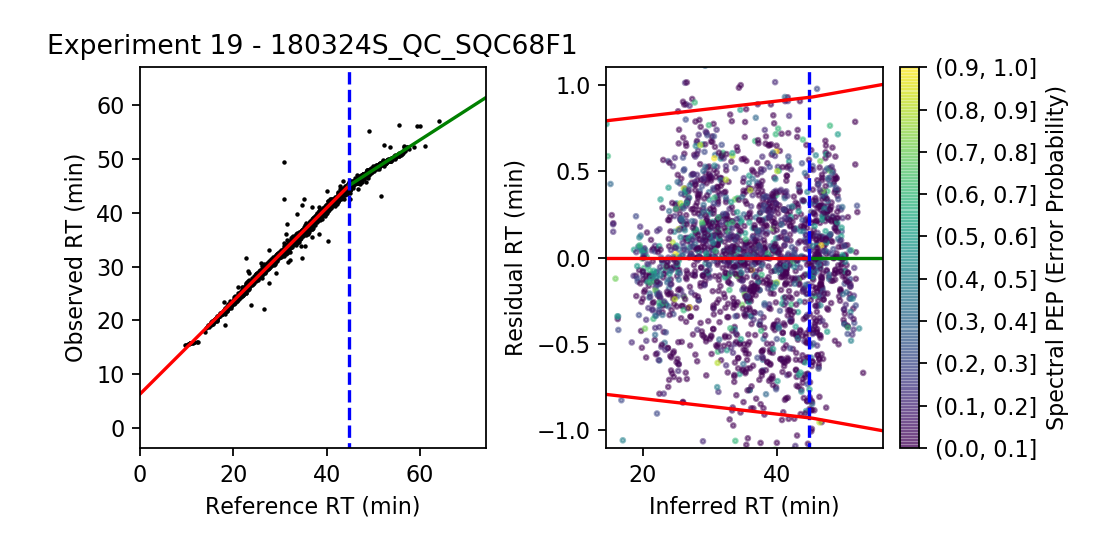

Supplement: S1 File — A optional HTML report generated by the dart_id Python script. The report gives a summary of the alignment for each experiment, as well as a broad overview of the performance of the run as a whole, by showing aggregate increases in PSMs at a chosen confidence threshold. (ZIP) [file pcbi.1007082.s001.zip › DART-ID_SCoPE-MS_Report/figures/alignment_19_180324S_QC_SQC68F1.png]

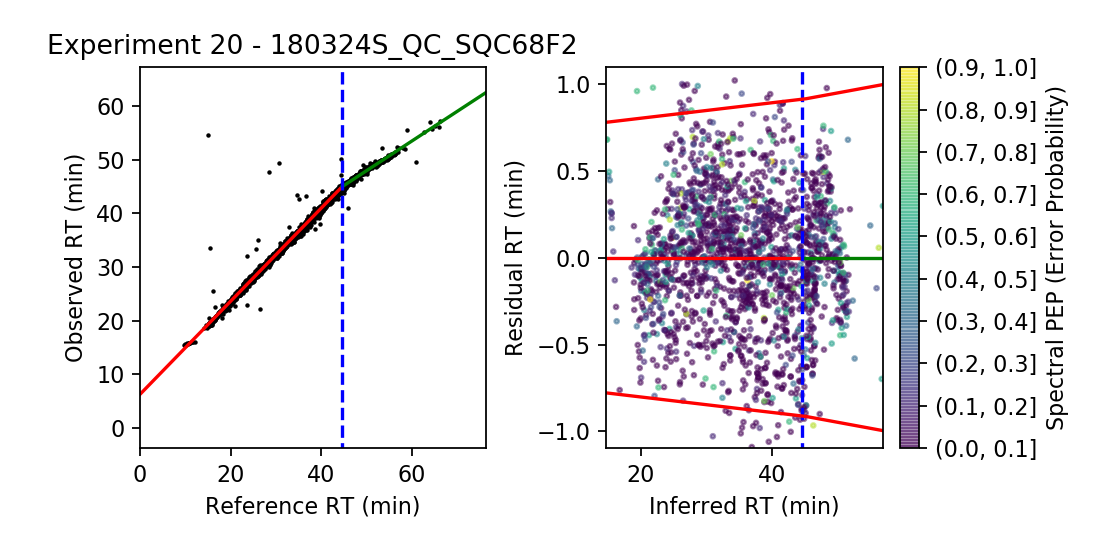

Supplement: S1 File — A optional HTML report generated by the dart_id Python script. The report gives a summary of the alignment for each experiment, as well as a broad overview of the performance of the run as a whole, by showing aggregate increases in PSMs at a chosen confidence threshold. (ZIP) [file pcbi.1007082.s001.zip › DART-ID_SCoPE-MS_Report/figures/alignment_20_180324S_QC_SQC68F2.png]

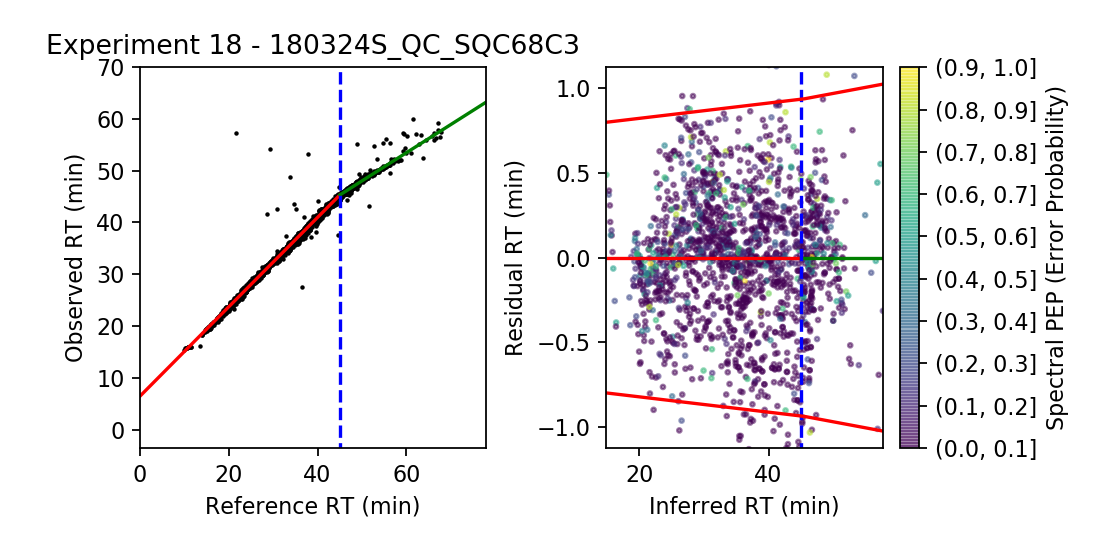

Supplement: S1 File — A optional HTML report generated by the dart_id Python script. The report gives a summary of the alignment for each experiment, as well as a broad overview of the performance of the run as a whole, by showing aggregate increases in PSMs at a chosen confidence threshold. (ZIP) [file pcbi.1007082.s001.zip › DART-ID_SCoPE-MS_Report/figures/alignment_18_180324S_QC_SQC68C3.png]

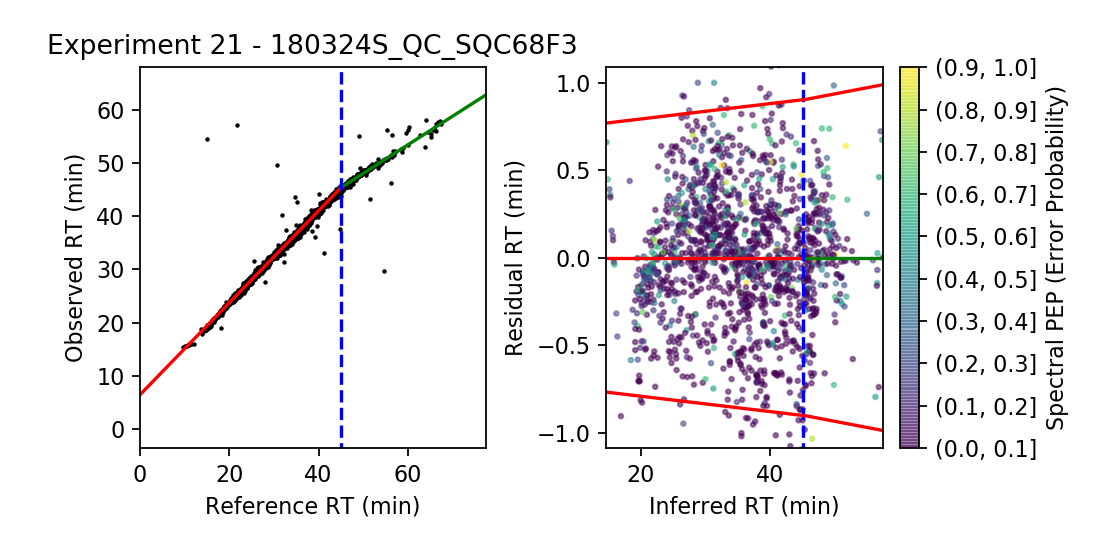

Supplement: S1 File — A optional HTML report generated by the dart_id Python script. The report gives a summary of the alignment for each experiment, as well as a broad overview of the performance of the run as a whole, by showing aggregate increases in PSMs at a chosen confidence threshold. (ZIP) [file pcbi.1007082.s001.zip › DART-ID_SCoPE-MS_Report/figures/alignment_21_180324S_QC_SQC68F3.png]

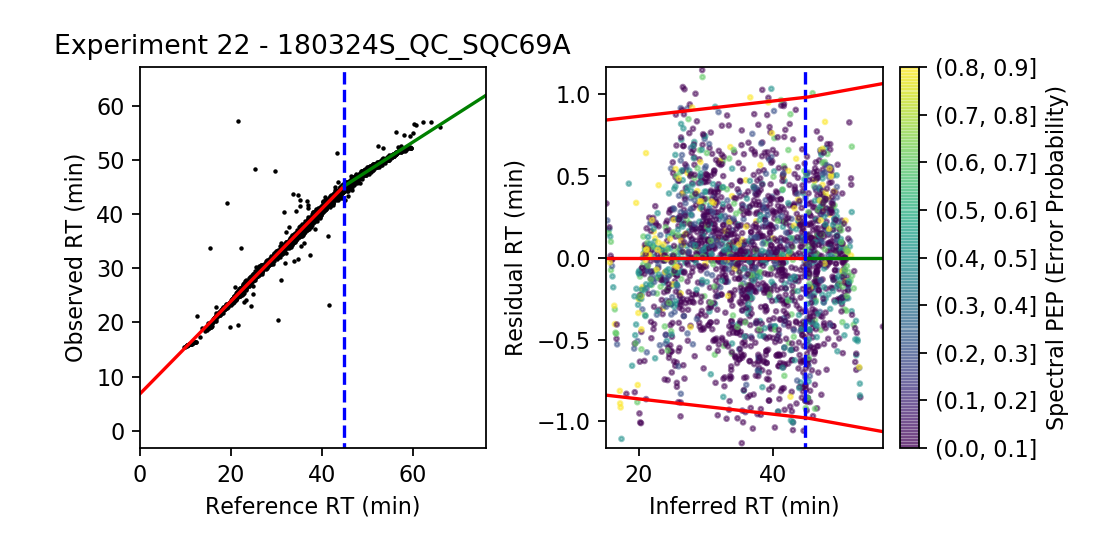

Supplement: S1 File — A optional HTML report generated by the dart_id Python script. The report gives a summary of the alignment for each experiment, as well as a broad overview of the performance of the run as a whole, by showing aggregate increases in PSMs at a chosen confidence threshold. (ZIP) [file pcbi.1007082.s001.zip › DART-ID_SCoPE-MS_Report/figures/alignment_22_180324S_QC_SQC69A.png]

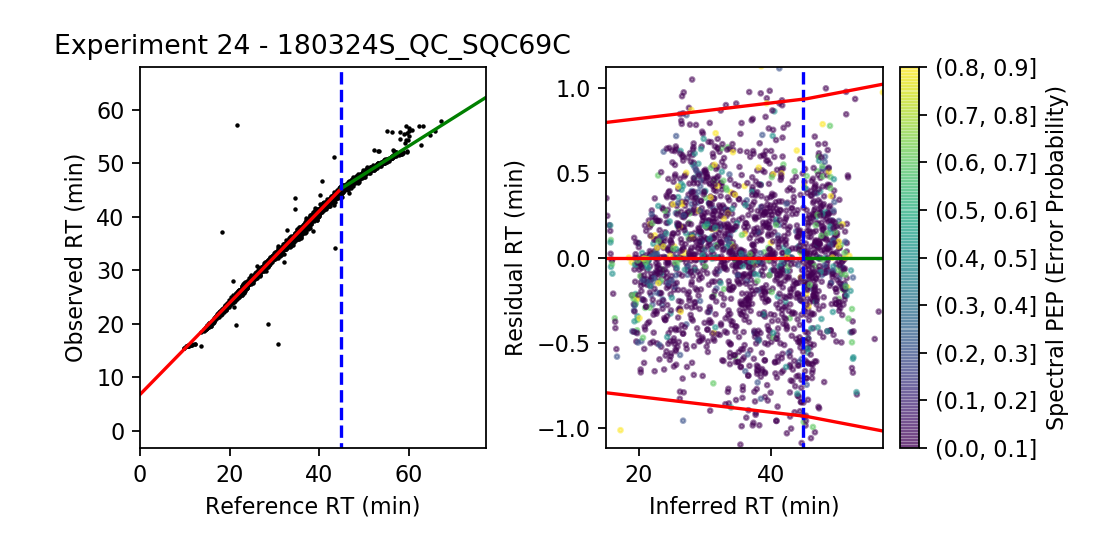

Supplement: S1 File — A optional HTML report generated by the dart_id Python script. The report gives a summary of the alignment for each experiment, as well as a broad overview of the performance of the run as a whole, by showing aggregate increases in PSMs at a chosen confidence threshold. (ZIP) [file pcbi.1007082.s001.zip › DART-ID_SCoPE-MS_Report/figures/alignment_24_180324S_QC_SQC69C.png]

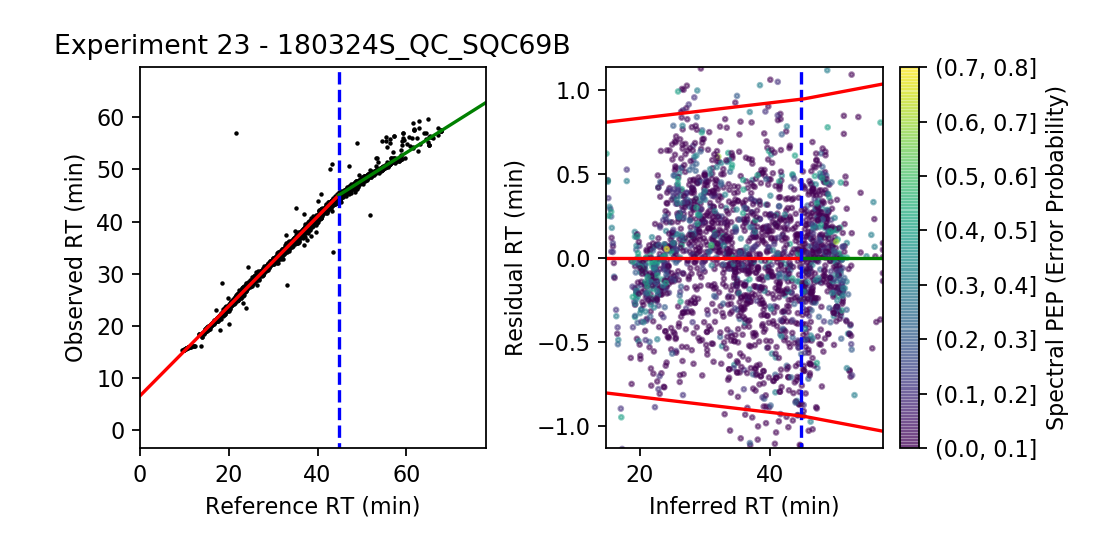

Supplement: S1 File — A optional HTML report generated by the dart_id Python script. The report gives a summary of the alignment for each experiment, as well as a broad overview of the performance of the run as a whole, by showing aggregate increases in PSMs at a chosen confidence threshold. (ZIP) [file pcbi.1007082.s001.zip › DART-ID_SCoPE-MS_Report/figures/alignment_23_180324S_QC_SQC69B.png]

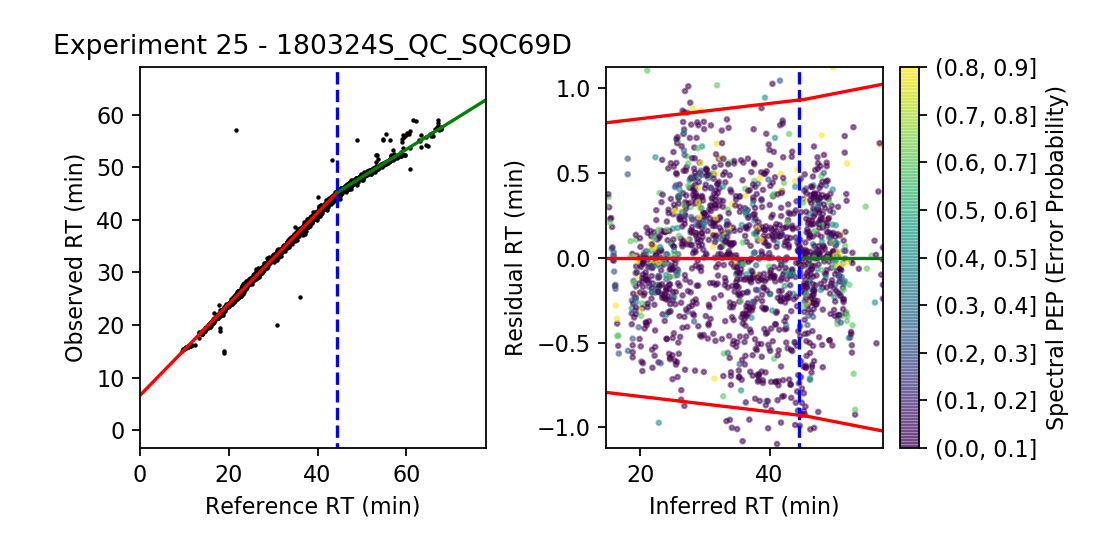

Supplement: S1 File — A optional HTML report generated by the dart_id Python script. The report gives a summary of the alignment for each experiment, as well as a broad overview of the performance of the run as a whole, by showing aggregate increases in PSMs at a chosen confidence threshold. (ZIP) [file pcbi.1007082.s001.zip › DART-ID_SCoPE-MS_Report/figures/alignment_25_180324S_QC_SQC69D.png]

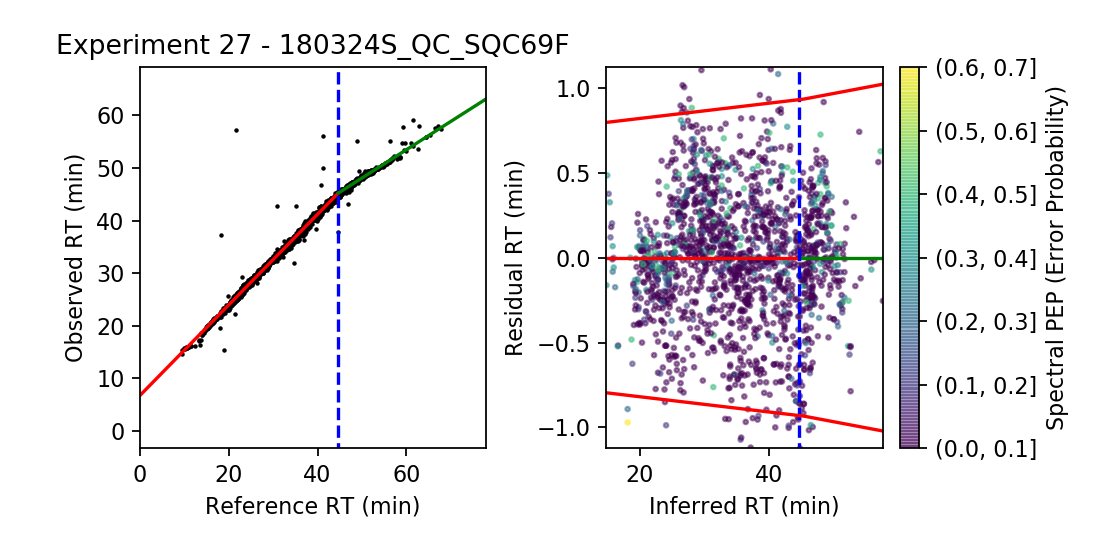

Supplement: S1 File — A optional HTML report generated by the dart_id Python script. The report gives a summary of the alignment for each experiment, as well as a broad overview of the performance of the run as a whole, by showing aggregate increases in PSMs at a chosen confidence threshold. (ZIP) [file pcbi.1007082.s001.zip › DART-ID_SCoPE-MS_Report/figures/alignment_27_180324S_QC_SQC69F.png]

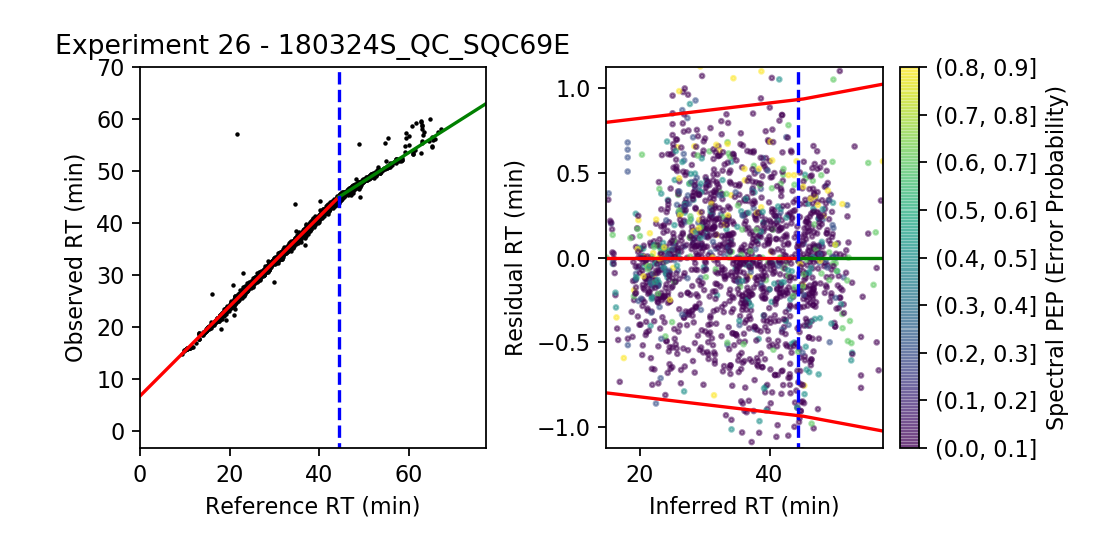

Supplement: S1 File — A optional HTML report generated by the dart_id Python script. The report gives a summary of the alignment for each experiment, as well as a broad overview of the performance of the run as a whole, by showing aggregate increases in PSMs at a chosen confidence threshold. (ZIP) [file pcbi.1007082.s001.zip › DART-ID_SCoPE-MS_Report/figures/alignment_26_180324S_QC_SQC69E.png]

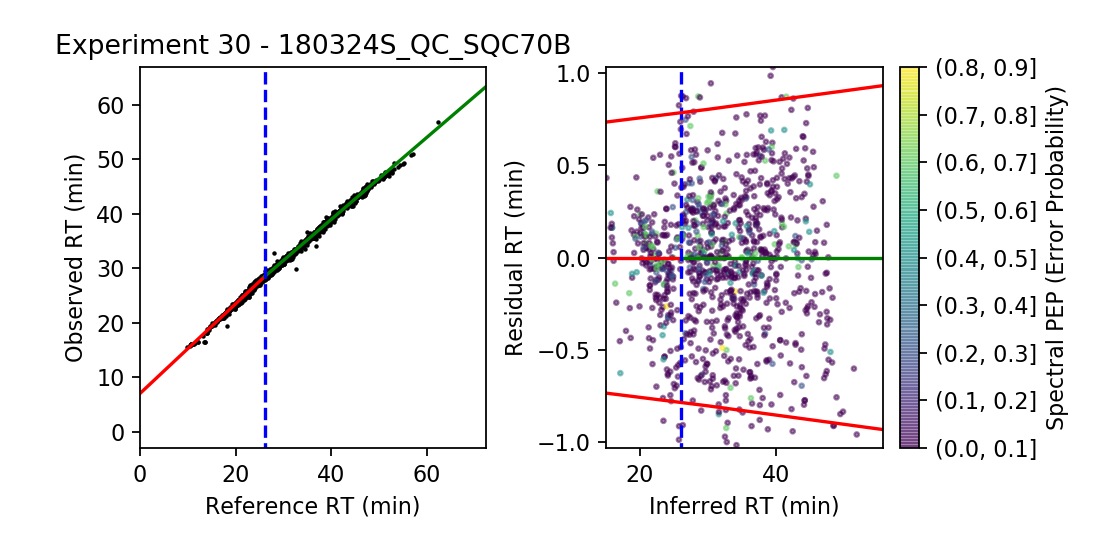

Supplement: S1 File — A optional HTML report generated by the dart_id Python script. The report gives a summary of the alignment for each experiment, as well as a broad overview of the performance of the run as a whole, by showing aggregate increases in PSMs at a chosen confidence threshold. (ZIP) [file pcbi.1007082.s001.zip › DART-ID_SCoPE-MS_Report/figures/alignment_30_180324S_QC_SQC70B.png]

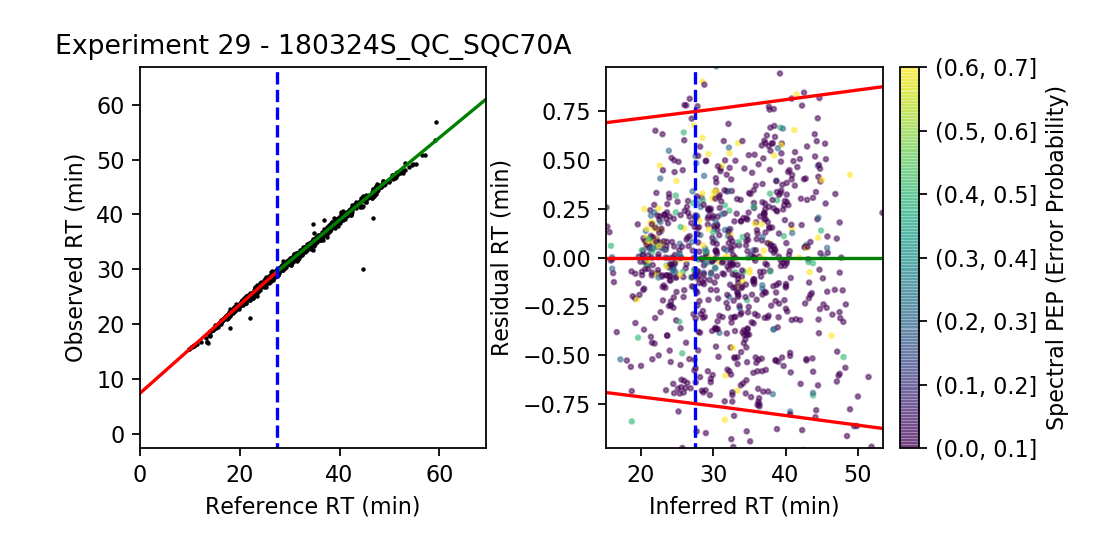

Supplement: S1 File — A optional HTML report generated by the dart_id Python script. The report gives a summary of the alignment for each experiment, as well as a broad overview of the performance of the run as a whole, by showing aggregate increases in PSMs at a chosen confidence threshold. (ZIP) [file pcbi.1007082.s001.zip › DART-ID_SCoPE-MS_Report/figures/alignment_29_180324S_QC_SQC70A.png]

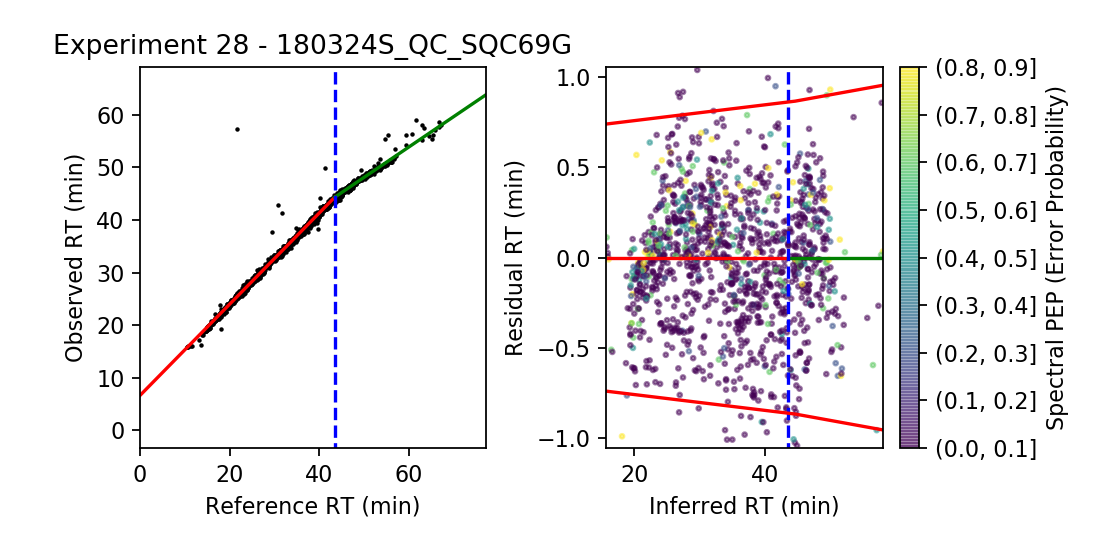

Supplement: S1 File — A optional HTML report generated by the dart_id Python script. The report gives a summary of the alignment for each experiment, as well as a broad overview of the performance of the run as a whole, by showing aggregate increases in PSMs at a chosen confidence threshold. (ZIP) [file pcbi.1007082.s001.zip › DART-ID_SCoPE-MS_Report/figures/alignment_28_180324S_QC_SQC69G.png]

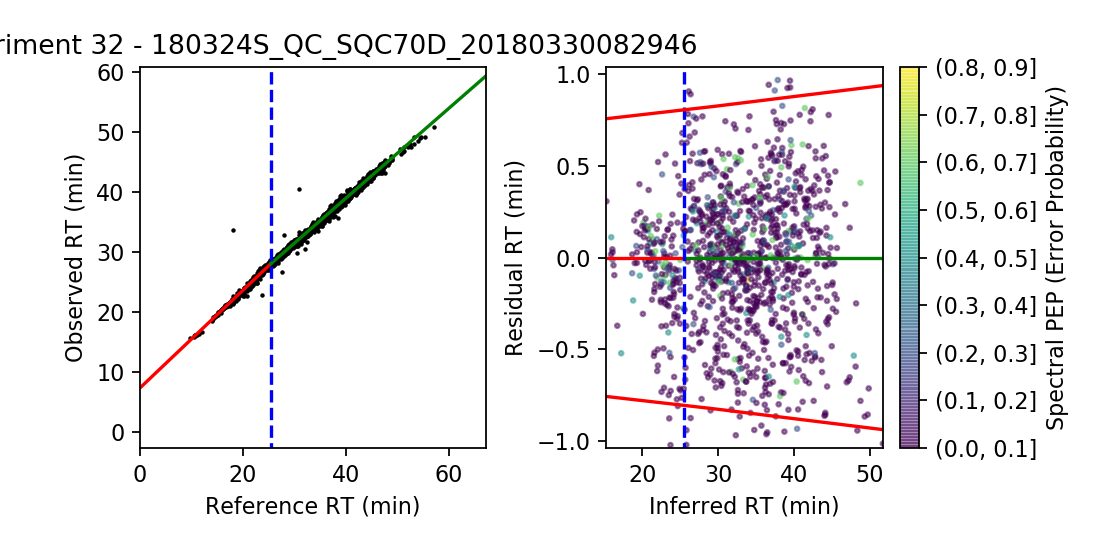

Supplement: S1 File — A optional HTML report generated by the dart_id Python script. The report gives a summary of the alignment for each experiment, as well as a broad overview of the performance of the run as a whole, by showing aggregate increases in PSMs at a chosen confidence threshold. (ZIP) [file pcbi.1007082.s001.zip › DART-ID_SCoPE-MS_Report/figures/alignment_32_180324S_QC_SQC70D_20180330082946.png]

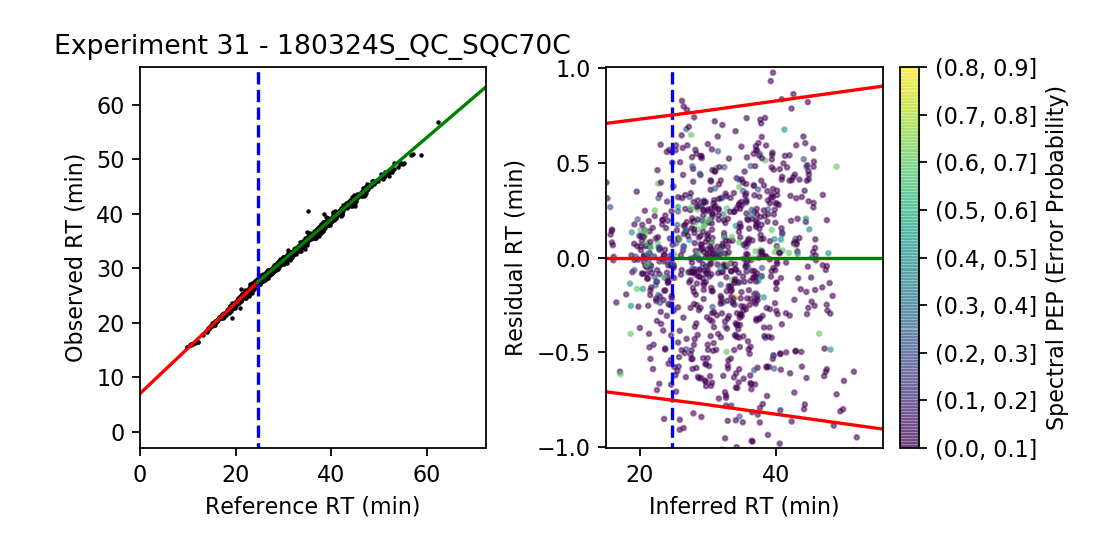

Supplement: S1 File — A optional HTML report generated by the dart_id Python script. The report gives a summary of the alignment for each experiment, as well as a broad overview of the performance of the run as a whole, by showing aggregate increases in PSMs at a chosen confidence threshold. (ZIP) [file pcbi.1007082.s001.zip › DART-ID_SCoPE-MS_Report/figures/alignment_31_180324S_QC_SQC70C.png]

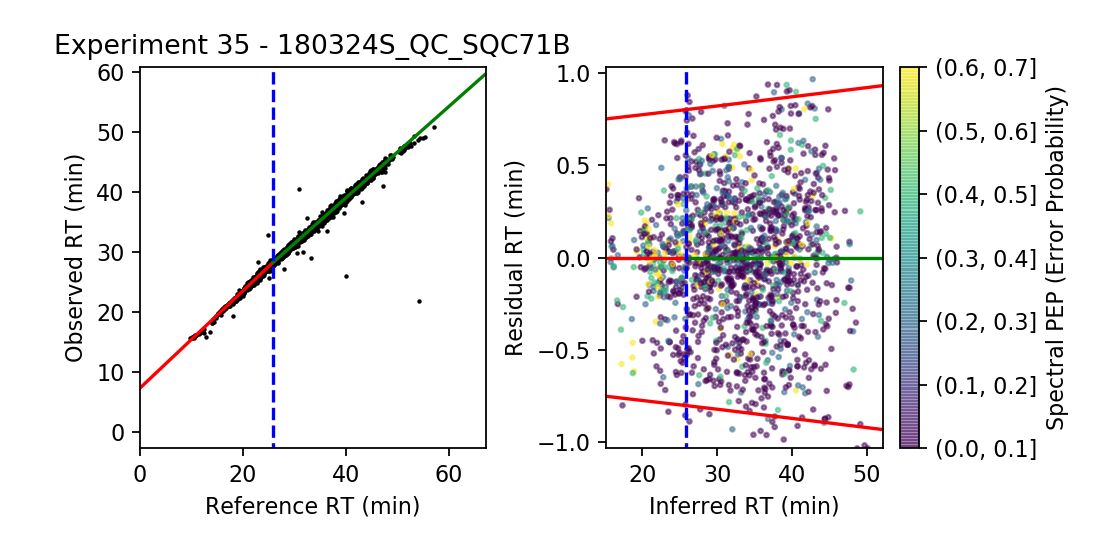

Supplement: S1 File — A optional HTML report generated by the dart_id Python script. The report gives a summary of the alignment for each experiment, as well as a broad overview of the performance of the run as a whole, by showing aggregate increases in PSMs at a chosen confidence threshold. (ZIP) [file pcbi.1007082.s001.zip › DART-ID_SCoPE-MS_Report/figures/alignment_35_180324S_QC_SQC71B.png]

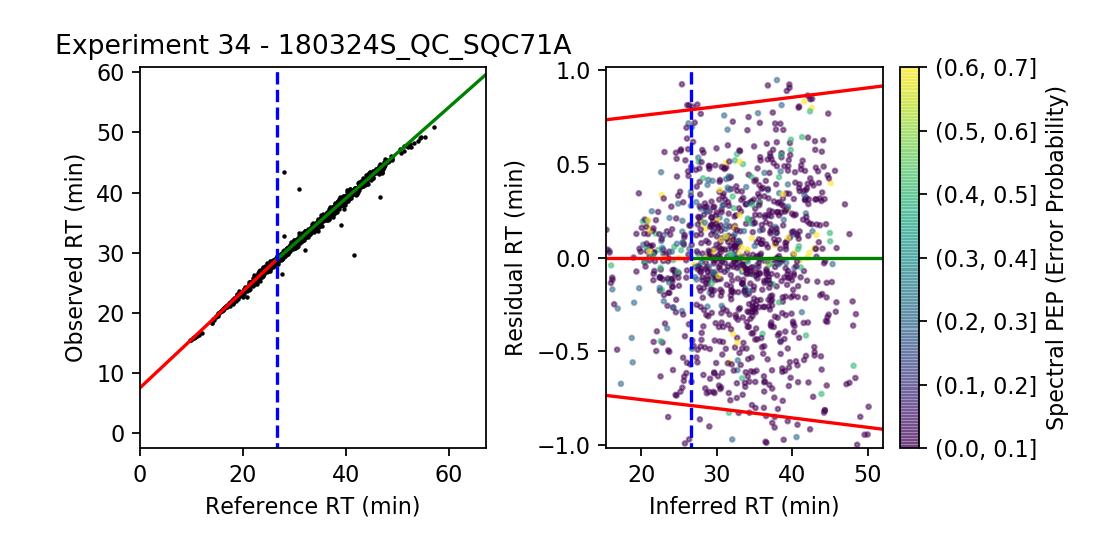

Supplement: S1 File — A optional HTML report generated by the dart_id Python script. The report gives a summary of the alignment for each experiment, as well as a broad overview of the performance of the run as a whole, by showing aggregate increases in PSMs at a chosen confidence threshold. (ZIP) [file pcbi.1007082.s001.zip › DART-ID_SCoPE-MS_Report/figures/alignment_34_180324S_QC_SQC71A.png]

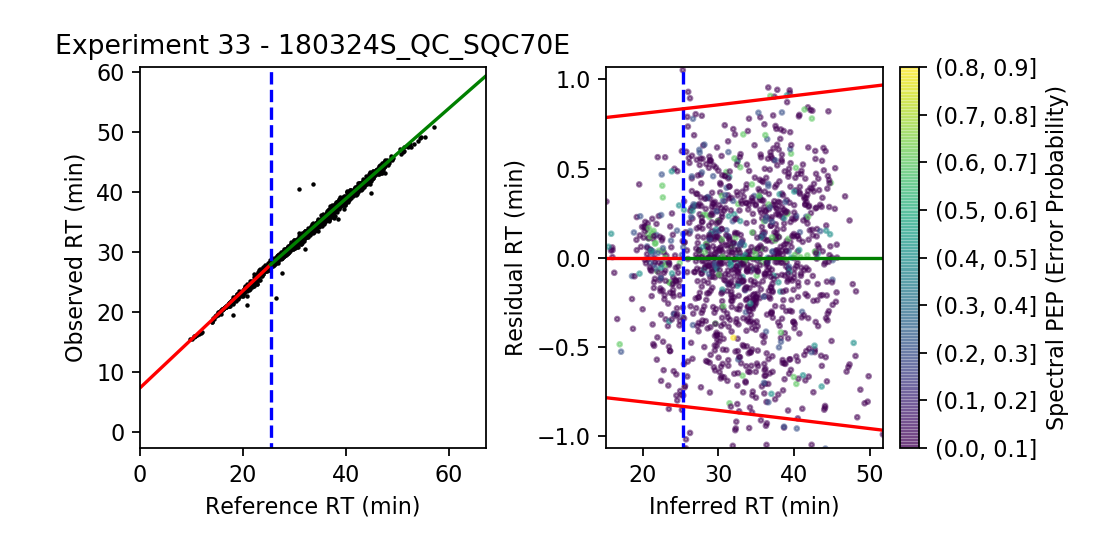

Supplement: S1 File — A optional HTML report generated by the dart_id Python script. The report gives a summary of the alignment for each experiment, as well as a broad overview of the performance of the run as a whole, by showing aggregate increases in PSMs at a chosen confidence threshold. (ZIP) [file pcbi.1007082.s001.zip › DART-ID_SCoPE-MS_Report/figures/alignment_33_180324S_QC_SQC70E.png]

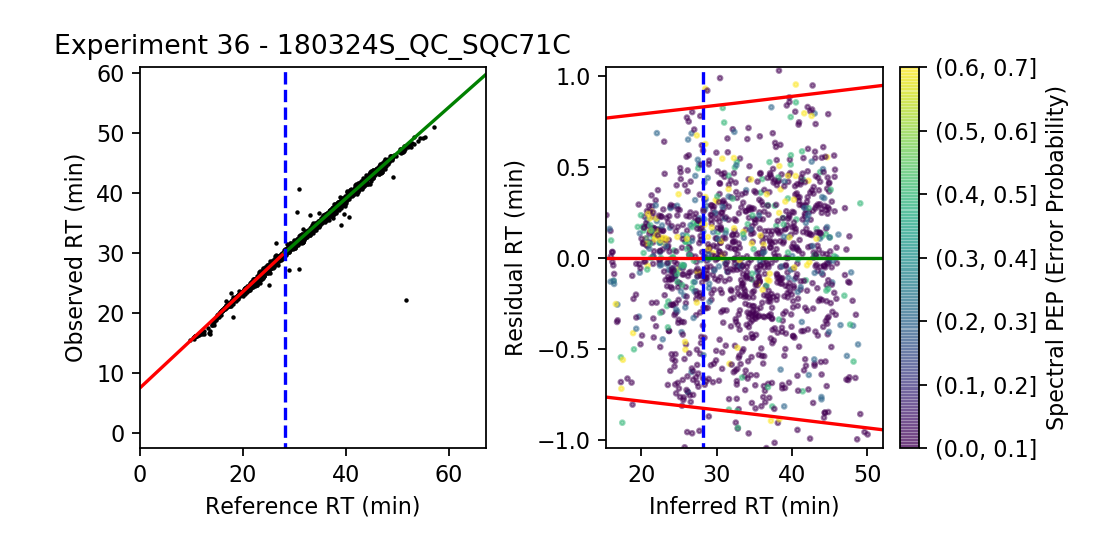

Supplement: S1 File — A optional HTML report generated by the dart_id Python script. The report gives a summary of the alignment for each experiment, as well as a broad overview of the performance of the run as a whole, by showing aggregate increases in PSMs at a chosen confidence threshold. (ZIP) [file pcbi.1007082.s001.zip › DART-ID_SCoPE-MS_Report/figures/alignment_36_180324S_QC_SQC71C.png]

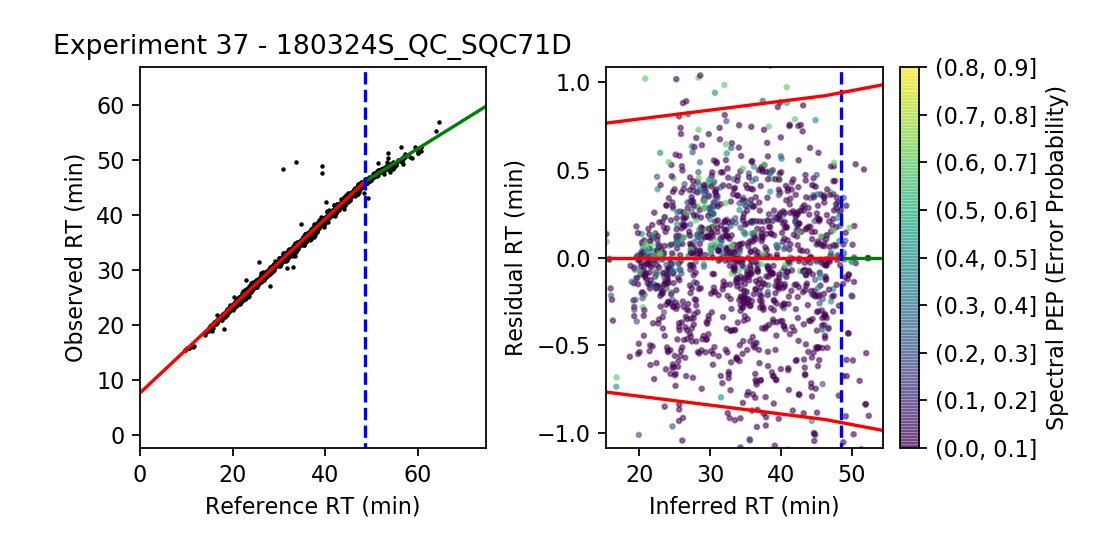

Supplement: S1 File — A optional HTML report generated by the dart_id Python script. The report gives a summary of the alignment for each experiment, as well as a broad overview of the performance of the run as a whole, by showing aggregate increases in PSMs at a chosen confidence threshold. (ZIP) [file pcbi.1007082.s001.zip › DART-ID_SCoPE-MS_Report/figures/alignment_37_180324S_QC_SQC71D.png]

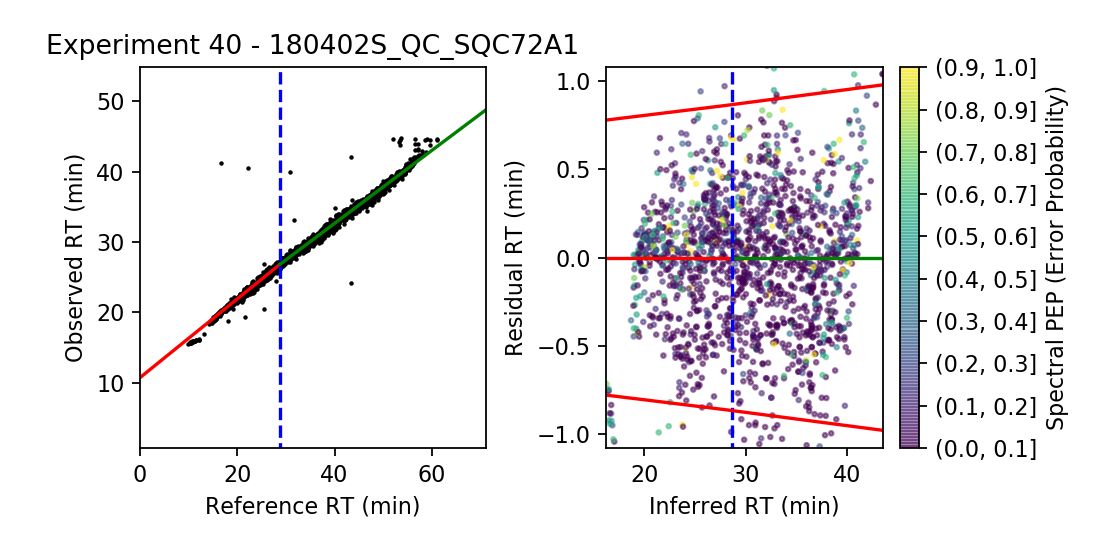

Supplement: S1 File — A optional HTML report generated by the dart_id Python script. The report gives a summary of the alignment for each experiment, as well as a broad overview of the performance of the run as a whole, by showing aggregate increases in PSMs at a chosen confidence threshold. (ZIP) [file pcbi.1007082.s001.zip › DART-ID_SCoPE-MS_Report/figures/alignment_40_180402S_QC_SQC72A1.png]

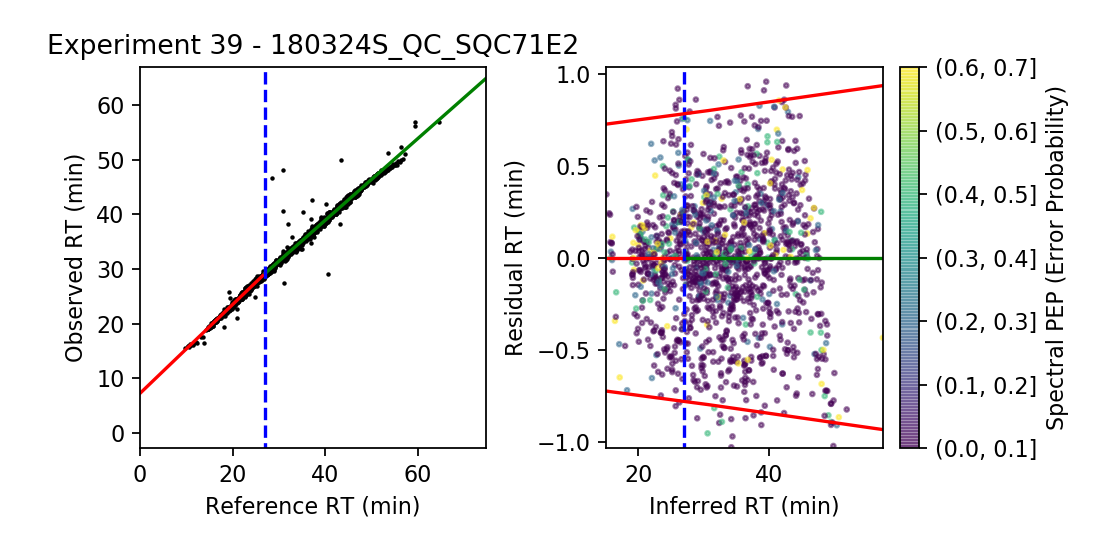

Supplement: S1 File — A optional HTML report generated by the dart_id Python script. The report gives a summary of the alignment for each experiment, as well as a broad overview of the performance of the run as a whole, by showing aggregate increases in PSMs at a chosen confidence threshold. (ZIP) [file pcbi.1007082.s001.zip › DART-ID_SCoPE-MS_Report/figures/alignment_39_180324S_QC_SQC71E2.png]

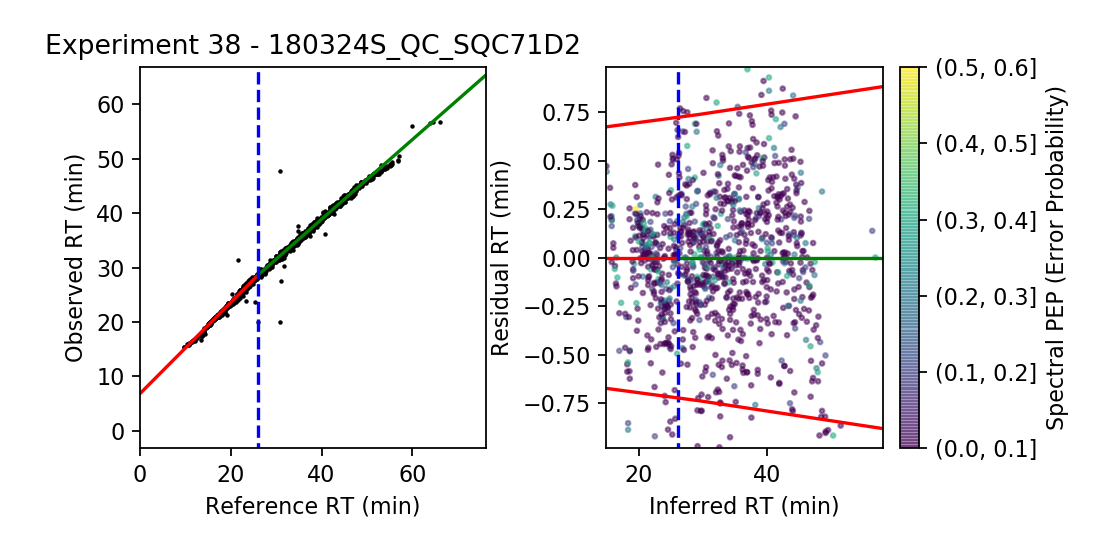

Supplement: S1 File — A optional HTML report generated by the dart_id Python script. The report gives a summary of the alignment for each experiment, as well as a broad overview of the performance of the run as a whole, by showing aggregate increases in PSMs at a chosen confidence threshold. (ZIP) [file pcbi.1007082.s001.zip › DART-ID_SCoPE-MS_Report/figures/alignment_38_180324S_QC_SQC71D2.png]

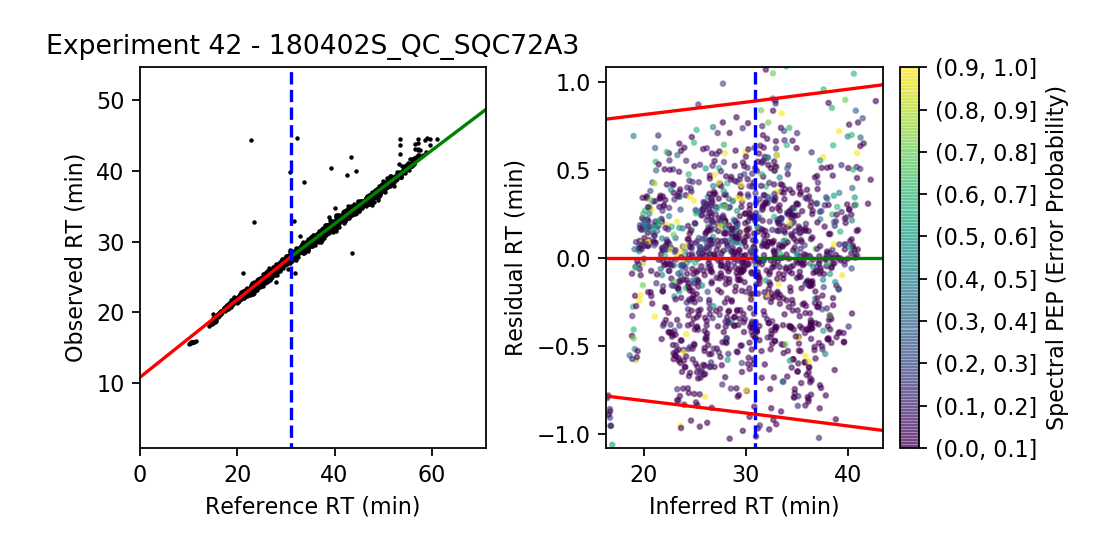

Supplement: S1 File — A optional HTML report generated by the dart_id Python script. The report gives a summary of the alignment for each experiment, as well as a broad overview of the performance of the run as a whole, by showing aggregate increases in PSMs at a chosen confidence threshold. (ZIP) [file pcbi.1007082.s001.zip › DART-ID_SCoPE-MS_Report/figures/alignment_42_180402S_QC_SQC72A3.png]

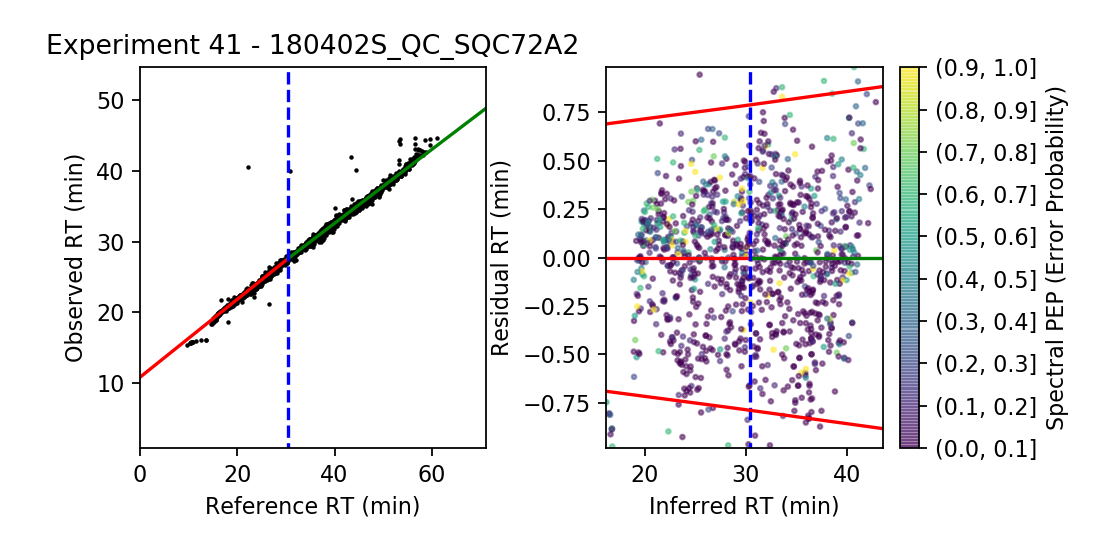

Supplement: S1 File — A optional HTML report generated by the dart_id Python script. The report gives a summary of the alignment for each experiment, as well as a broad overview of the performance of the run as a whole, by showing aggregate increases in PSMs at a chosen confidence threshold. (ZIP) [file pcbi.1007082.s001.zip › DART-ID_SCoPE-MS_Report/figures/alignment_41_180402S_QC_SQC72A2.png]

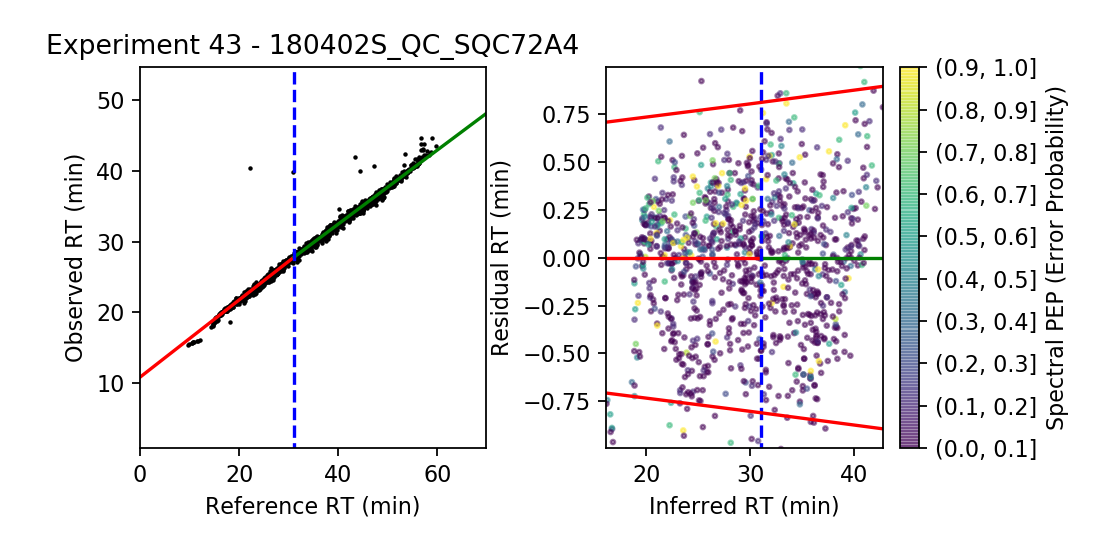

Supplement: S1 File — A optional HTML report generated by the dart_id Python script. The report gives a summary of the alignment for each experiment, as well as a broad overview of the performance of the run as a whole, by showing aggregate increases in PSMs at a chosen confidence threshold. (ZIP) [file pcbi.1007082.s001.zip › DART-ID_SCoPE-MS_Report/figures/alignment_43_180402S_QC_SQC72A4.png]

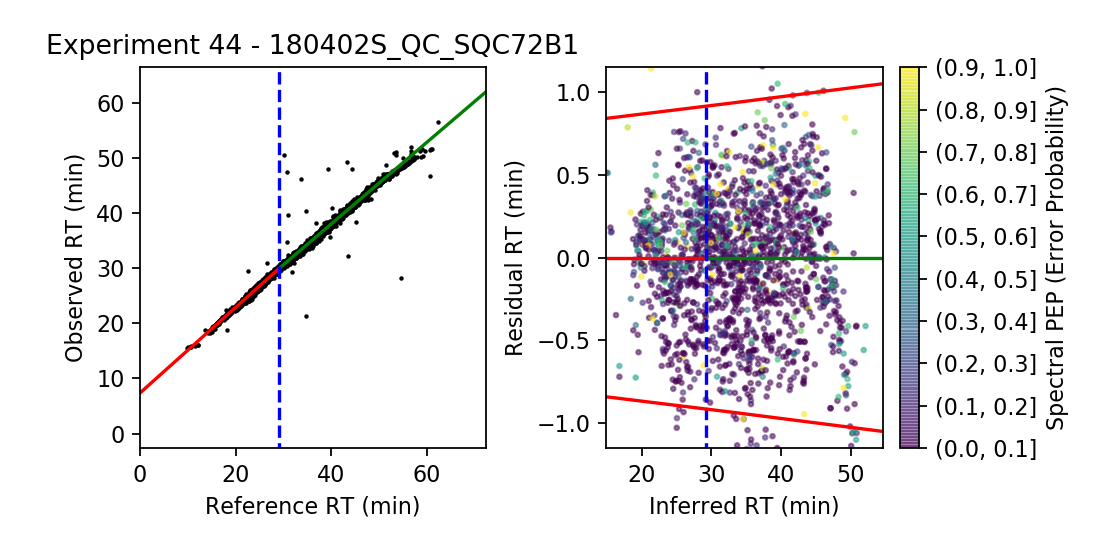

Supplement: S1 File — A optional HTML report generated by the dart_id Python script. The report gives a summary of the alignment for each experiment, as well as a broad overview of the performance of the run as a whole, by showing aggregate increases in PSMs at a chosen confidence threshold. (ZIP) [file pcbi.1007082.s001.zip › DART-ID_SCoPE-MS_Report/figures/alignment_44_180402S_QC_SQC72B1.png]

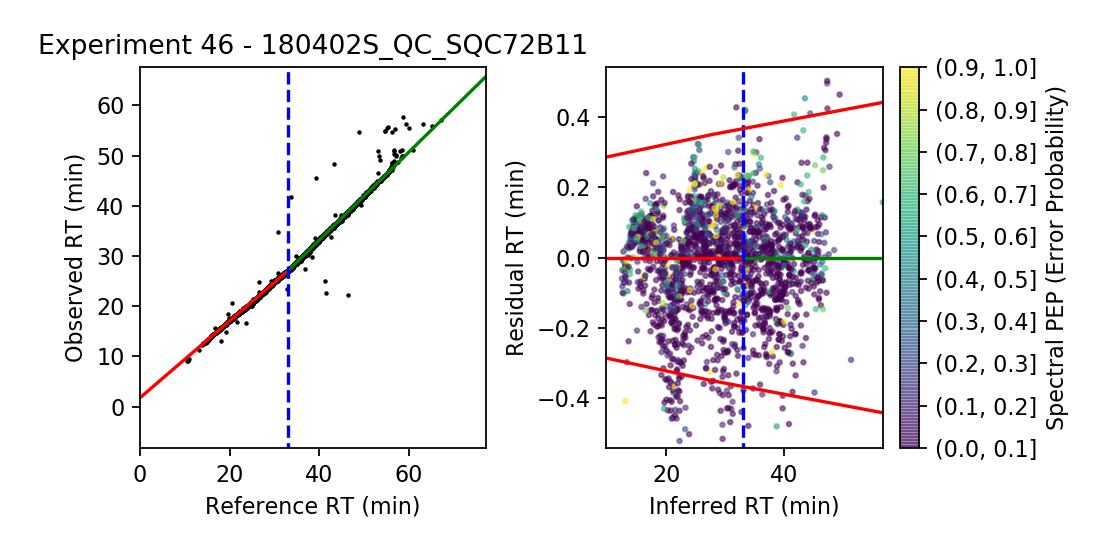

Supplement: S1 File — A optional HTML report generated by the dart_id Python script. The report gives a summary of the alignment for each experiment, as well as a broad overview of the performance of the run as a whole, by showing aggregate increases in PSMs at a chosen confidence threshold. (ZIP) [file pcbi.1007082.s001.zip › DART-ID_SCoPE-MS_Report/figures/alignment_46_180402S_QC_SQC72B11.png]

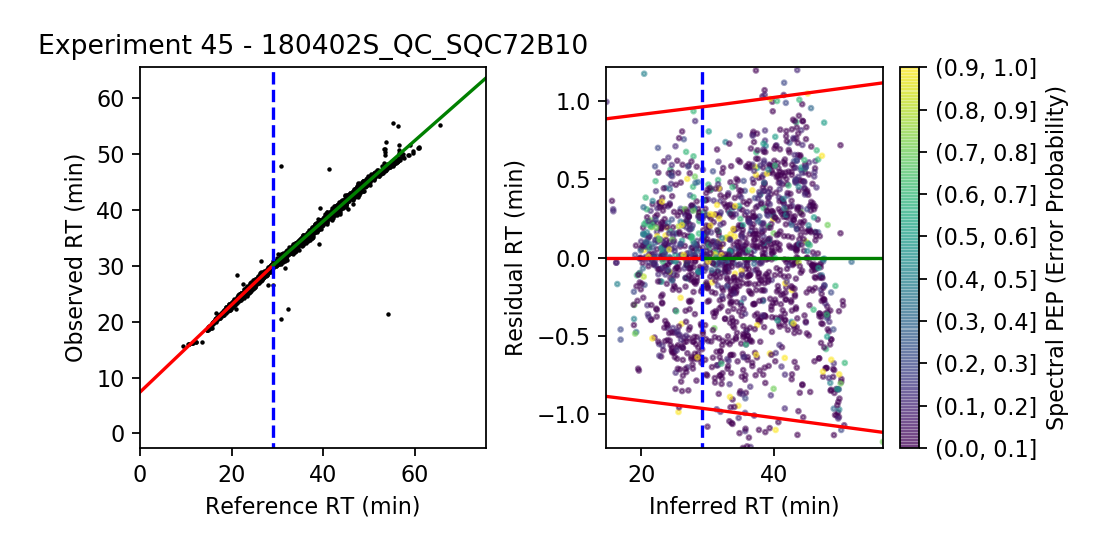

Supplement: S1 File — A optional HTML report generated by the dart_id Python script. The report gives a summary of the alignment for each experiment, as well as a broad overview of the performance of the run as a whole, by showing aggregate increases in PSMs at a chosen confidence threshold. (ZIP) [file pcbi.1007082.s001.zip › DART-ID_SCoPE-MS_Report/figures/alignment_45_180402S_QC_SQC72B10.png]

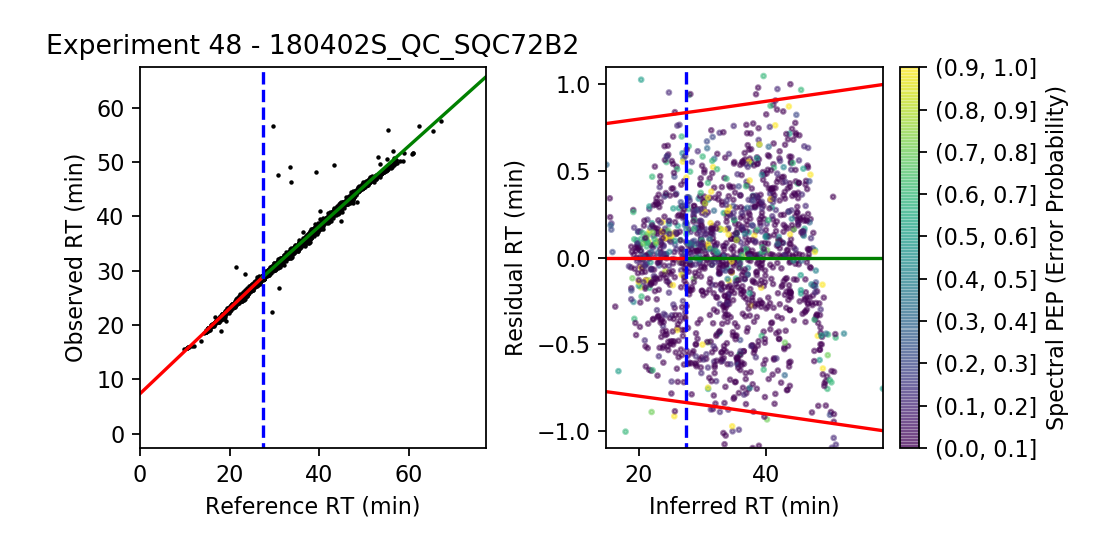

Supplement: S1 File — A optional HTML report generated by the dart_id Python script. The report gives a summary of the alignment for each experiment, as well as a broad overview of the performance of the run as a whole, by showing aggregate increases in PSMs at a chosen confidence threshold. (ZIP) [file pcbi.1007082.s001.zip › DART-ID_SCoPE-MS_Report/figures/alignment_48_180402S_QC_SQC72B2.png]

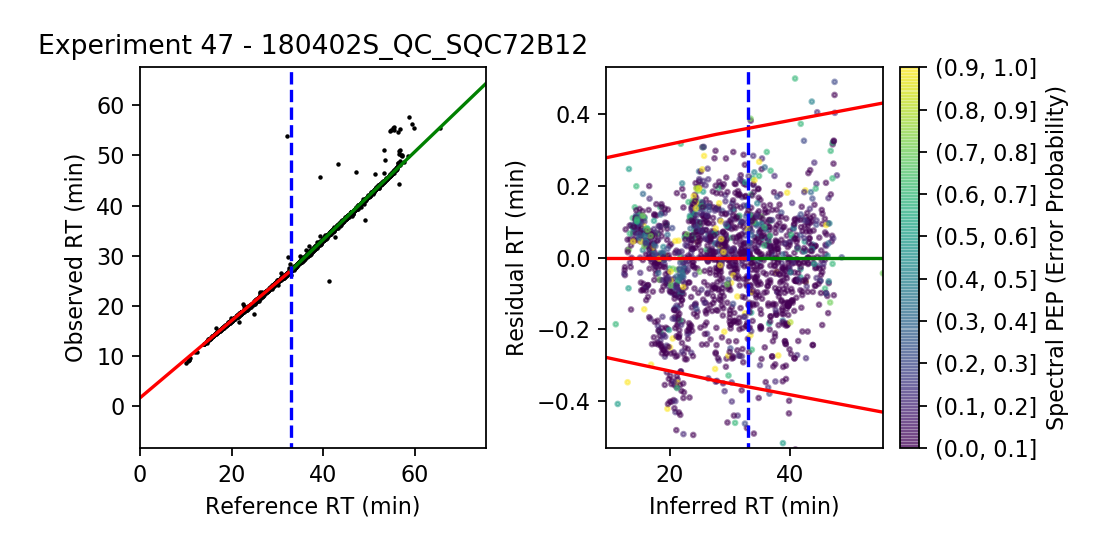

Supplement: S1 File — A optional HTML report generated by the dart_id Python script. The report gives a summary of the alignment for each experiment, as well as a broad overview of the performance of the run as a whole, by showing aggregate increases in PSMs at a chosen confidence threshold. (ZIP) [file pcbi.1007082.s001.zip › DART-ID_SCoPE-MS_Report/figures/alignment_47_180402S_QC_SQC72B12.png]

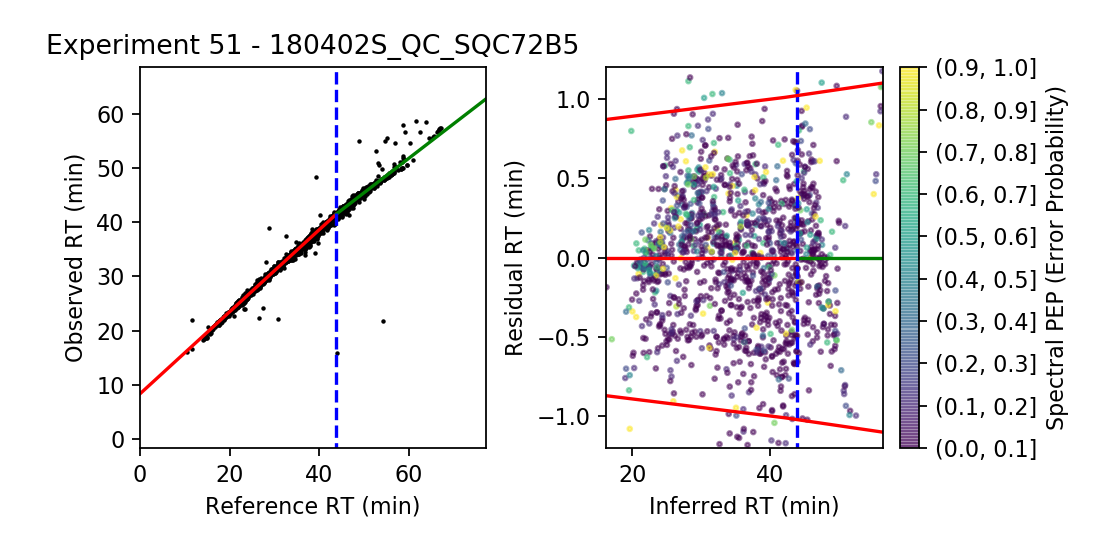

Supplement: S1 File — A optional HTML report generated by the dart_id Python script. The report gives a summary of the alignment for each experiment, as well as a broad overview of the performance of the run as a whole, by showing aggregate increases in PSMs at a chosen confidence threshold. (ZIP) [file pcbi.1007082.s001.zip › DART-ID_SCoPE-MS_Report/figures/alignment_51_180402S_QC_SQC72B5.png]

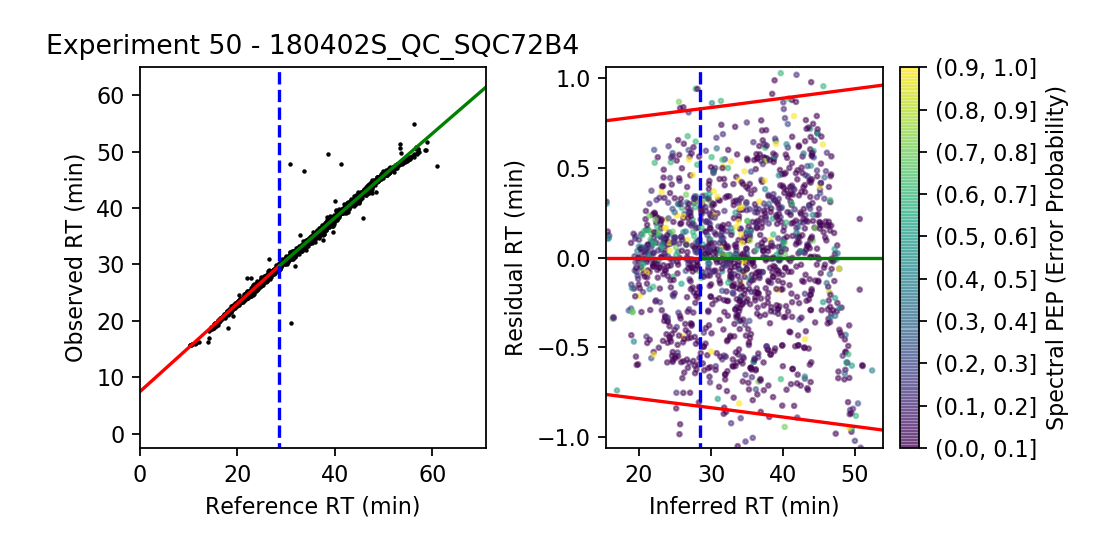

Supplement: S1 File — A optional HTML report generated by the dart_id Python script. The report gives a summary of the alignment for each experiment, as well as a broad overview of the performance of the run as a whole, by showing aggregate increases in PSMs at a chosen confidence threshold. (ZIP) [file pcbi.1007082.s001.zip › DART-ID_SCoPE-MS_Report/figures/alignment_50_180402S_QC_SQC72B4.png]

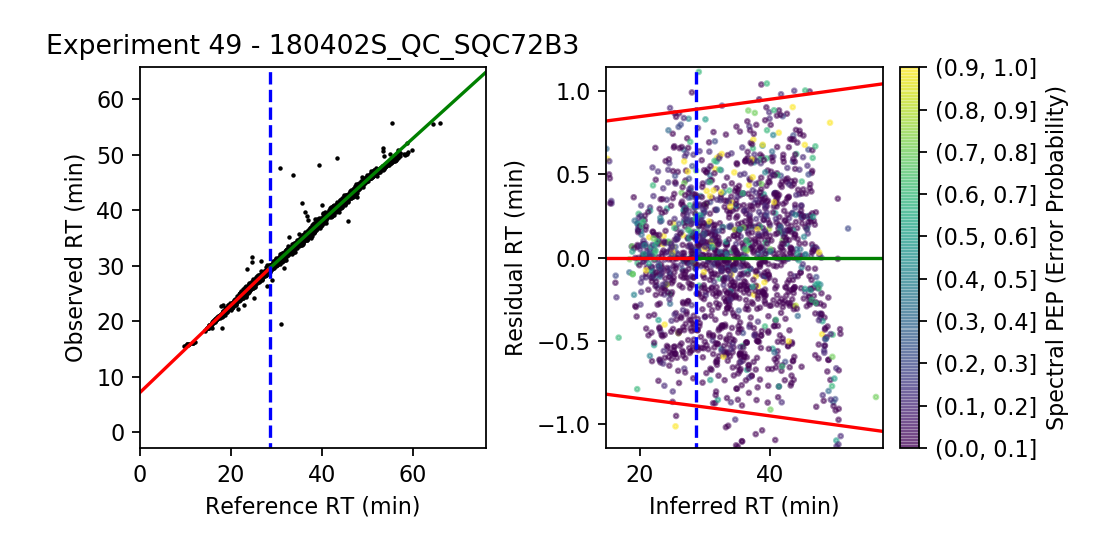

Supplement: S1 File — A optional HTML report generated by the dart_id Python script. The report gives a summary of the alignment for each experiment, as well as a broad overview of the performance of the run as a whole, by showing aggregate increases in PSMs at a chosen confidence threshold. (ZIP) [file pcbi.1007082.s001.zip › DART-ID_SCoPE-MS_Report/figures/alignment_49_180402S_QC_SQC72B3.png]

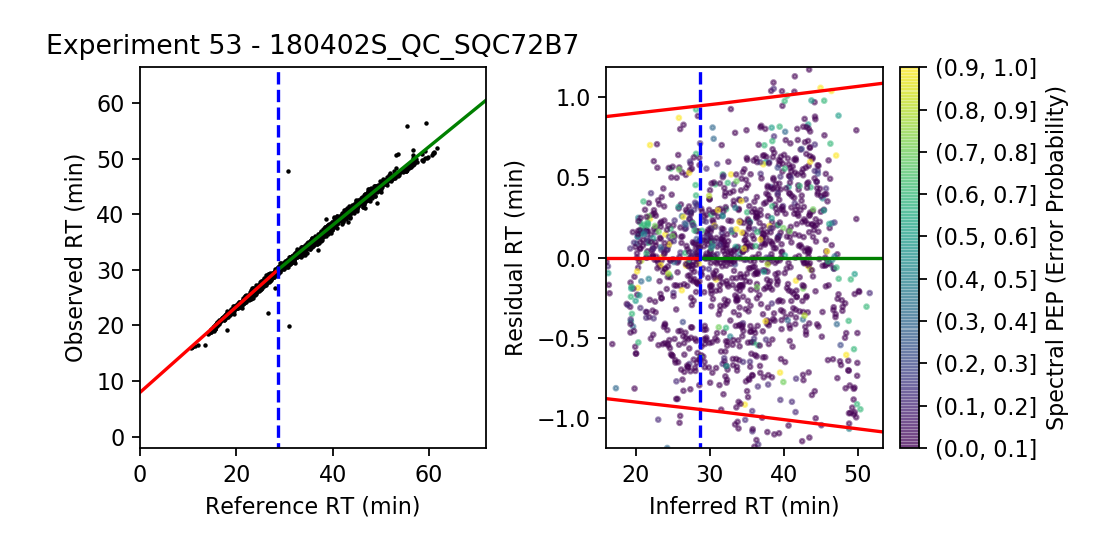

Supplement: S1 File — A optional HTML report generated by the dart_id Python script. The report gives a summary of the alignment for each experiment, as well as a broad overview of the performance of the run as a whole, by showing aggregate increases in PSMs at a chosen confidence threshold. (ZIP) [file pcbi.1007082.s001.zip › DART-ID_SCoPE-MS_Report/figures/alignment_53_180402S_QC_SQC72B7.png]

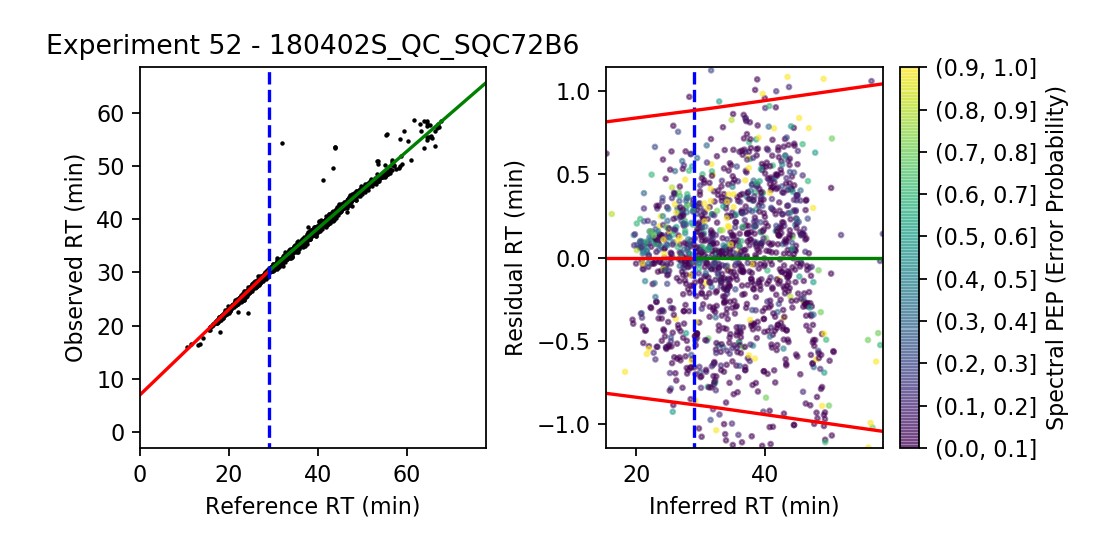

Supplement: S1 File — A optional HTML report generated by the dart_id Python script. The report gives a summary of the alignment for each experiment, as well as a broad overview of the performance of the run as a whole, by showing aggregate increases in PSMs at a chosen confidence threshold. (ZIP) [file pcbi.1007082.s001.zip › DART-ID_SCoPE-MS_Report/figures/alignment_52_180402S_QC_SQC72B6.png]

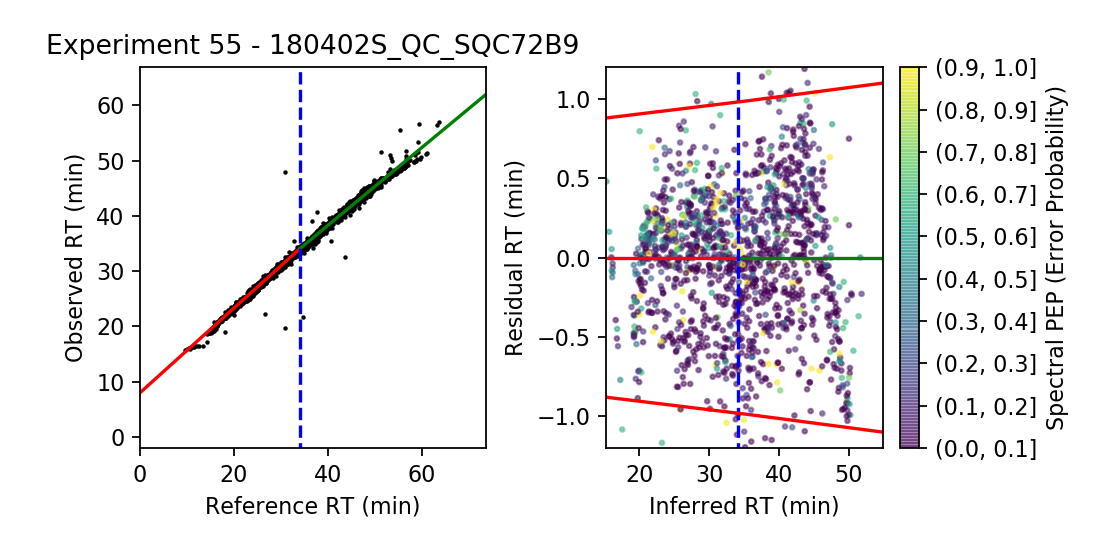

Supplement: S1 File — A optional HTML report generated by the dart_id Python script. The report gives a summary of the alignment for each experiment, as well as a broad overview of the performance of the run as a whole, by showing aggregate increases in PSMs at a chosen confidence threshold. (ZIP) [file pcbi.1007082.s001.zip › DART-ID_SCoPE-MS_Report/figures/alignment_55_180402S_QC_SQC72B9.png]

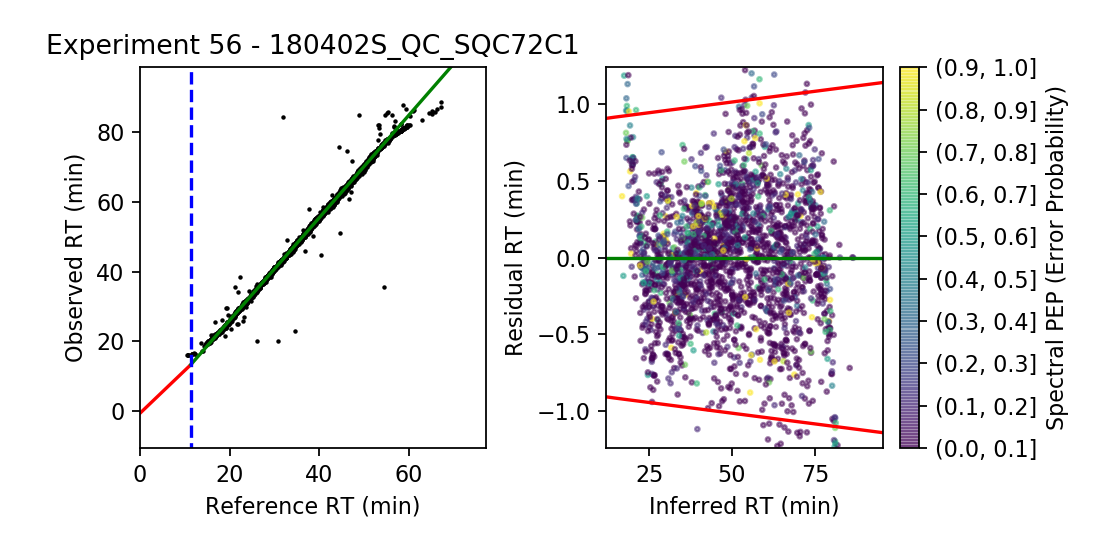

Supplement: S1 File — A optional HTML report generated by the dart_id Python script. The report gives a summary of the alignment for each experiment, as well as a broad overview of the performance of the run as a whole, by showing aggregate increases in PSMs at a chosen confidence threshold. (ZIP) [file pcbi.1007082.s001.zip › DART-ID_SCoPE-MS_Report/figures/alignment_56_180402S_QC_SQC72C1.png]

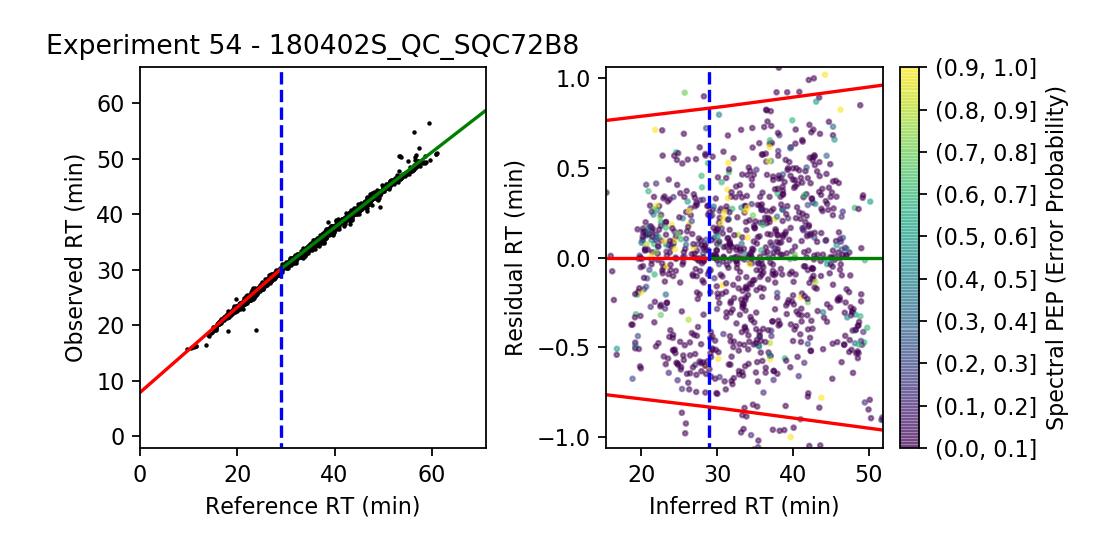

Supplement: S1 File — A optional HTML report generated by the dart_id Python script. The report gives a summary of the alignment for each experiment, as well as a broad overview of the performance of the run as a whole, by showing aggregate increases in PSMs at a chosen confidence threshold. (ZIP) [file pcbi.1007082.s001.zip › DART-ID_SCoPE-MS_Report/figures/alignment_54_180402S_QC_SQC72B8.png]

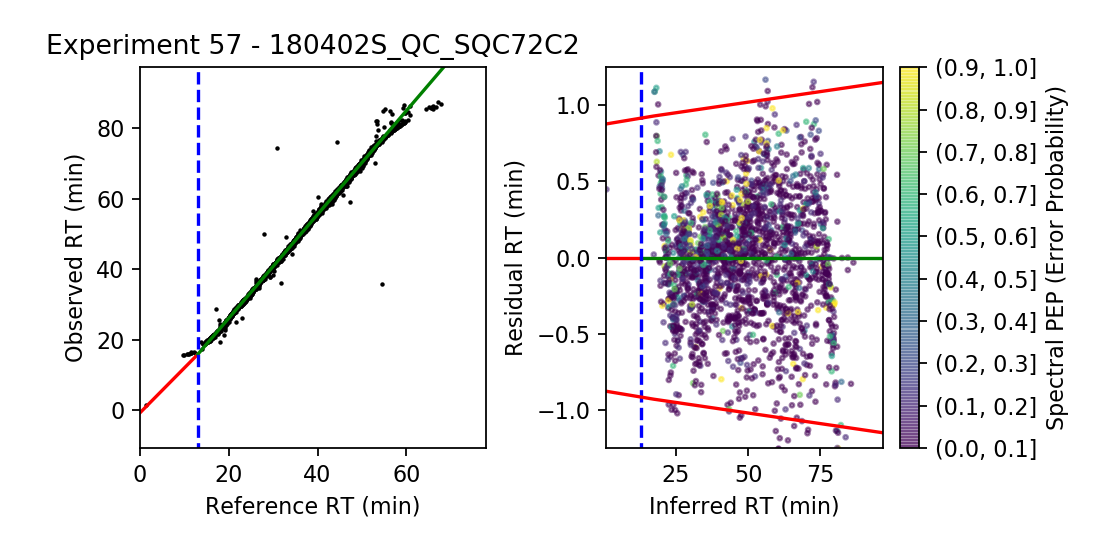

Supplement: S1 File — A optional HTML report generated by the dart_id Python script. The report gives a summary of the alignment for each experiment, as well as a broad overview of the performance of the run as a whole, by showing aggregate increases in PSMs at a chosen confidence threshold. (ZIP) [file pcbi.1007082.s001.zip › DART-ID_SCoPE-MS_Report/figures/alignment_57_180402S_QC_SQC72C2.png]

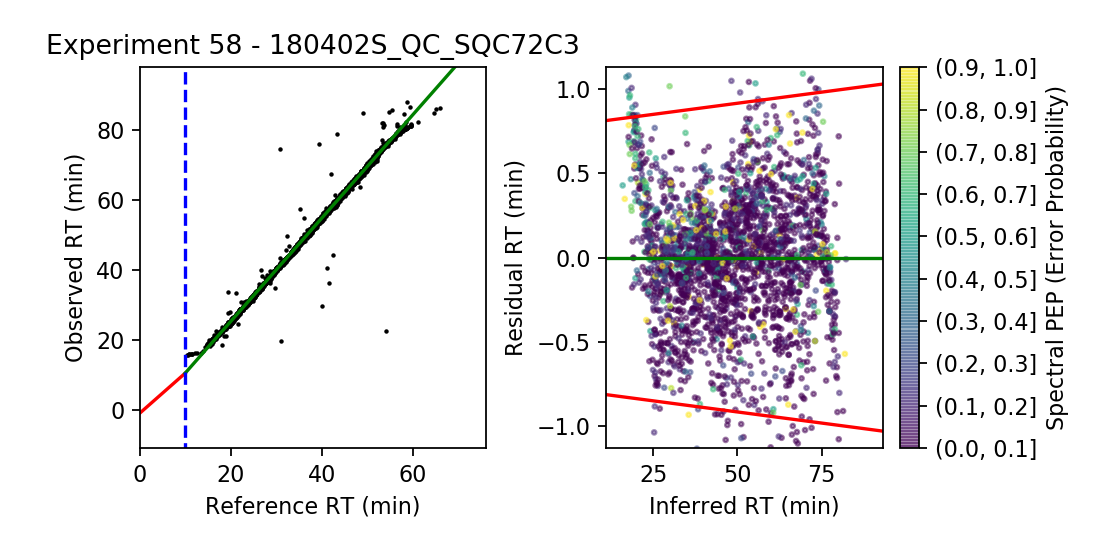

Supplement: S1 File — A optional HTML report generated by the dart_id Python script. The report gives a summary of the alignment for each experiment, as well as a broad overview of the performance of the run as a whole, by showing aggregate increases in PSMs at a chosen confidence threshold. (ZIP) [file pcbi.1007082.s001.zip › DART-ID_SCoPE-MS_Report/figures/alignment_58_180402S_QC_SQC72C3.png]

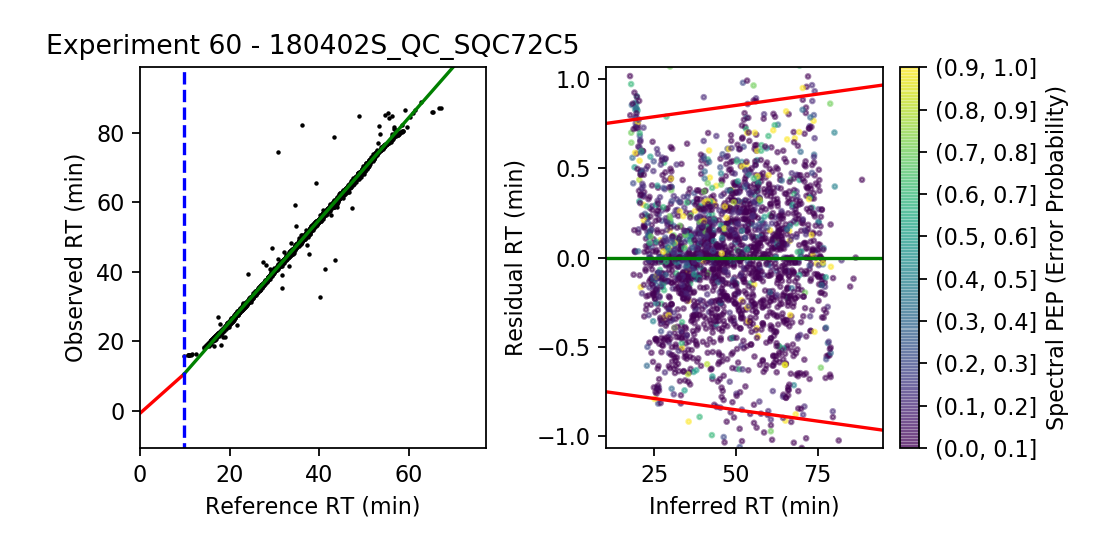

Supplement: S1 File — A optional HTML report generated by the dart_id Python script. The report gives a summary of the alignment for each experiment, as well as a broad overview of the performance of the run as a whole, by showing aggregate increases in PSMs at a chosen confidence threshold. (ZIP) [file pcbi.1007082.s001.zip › DART-ID_SCoPE-MS_Report/figures/alignment_60_180402S_QC_SQC72C5.png]

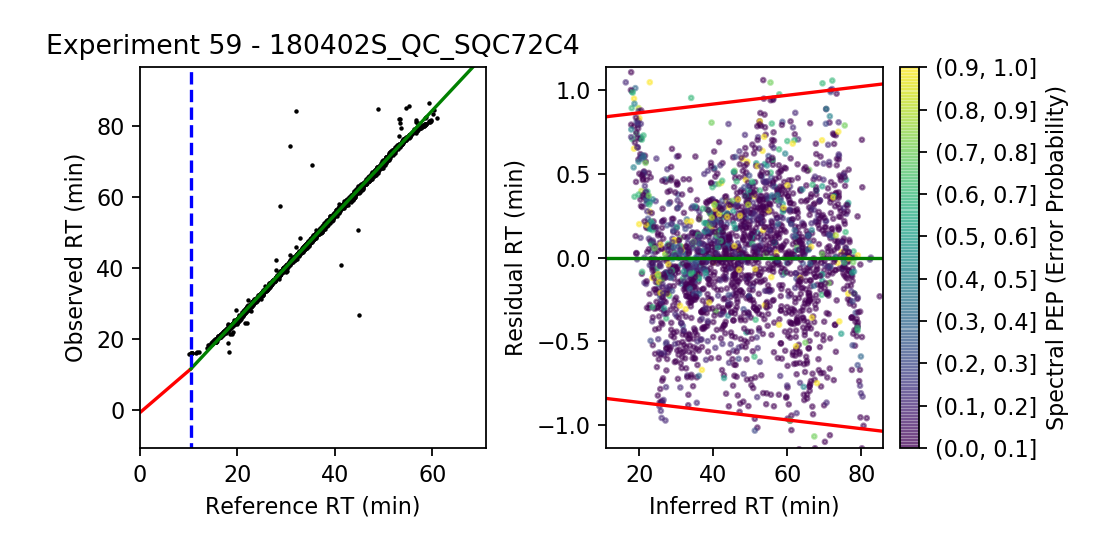

Supplement: S1 File — A optional HTML report generated by the dart_id Python script. The report gives a summary of the alignment for each experiment, as well as a broad overview of the performance of the run as a whole, by showing aggregate increases in PSMs at a chosen confidence threshold. (ZIP) [file pcbi.1007082.s001.zip › DART-ID_SCoPE-MS_Report/figures/alignment_59_180402S_QC_SQC72C4.png]

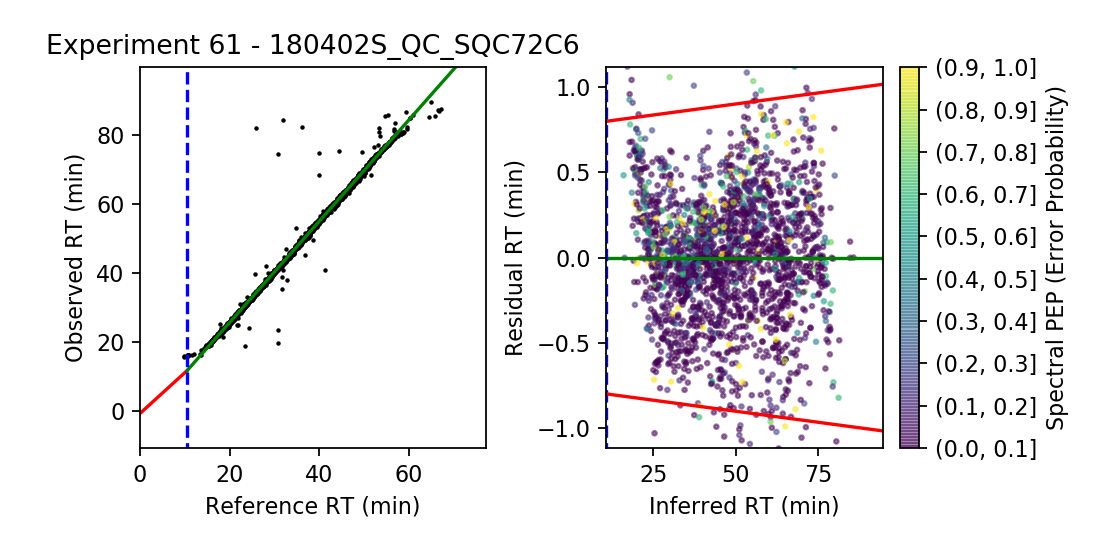

Supplement: S1 File — A optional HTML report generated by the dart_id Python script. The report gives a summary of the alignment for each experiment, as well as a broad overview of the performance of the run as a whole, by showing aggregate increases in PSMs at a chosen confidence threshold. (ZIP) [file pcbi.1007082.s001.zip › DART-ID_SCoPE-MS_Report/figures/alignment_61_180402S_QC_SQC72C6.png]

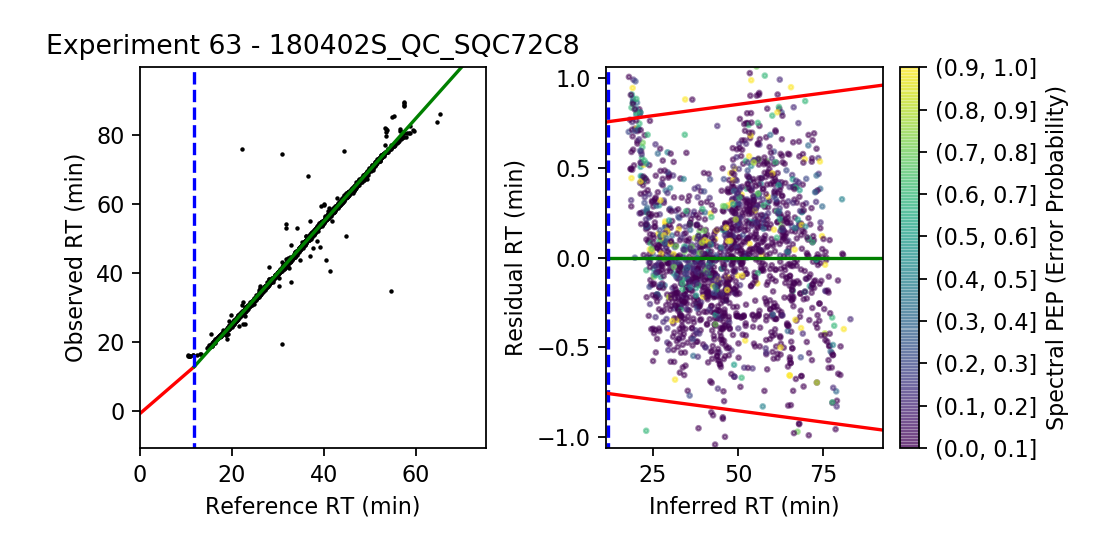

Supplement: S1 File — A optional HTML report generated by the dart_id Python script. The report gives a summary of the alignment for each experiment, as well as a broad overview of the performance of the run as a whole, by showing aggregate increases in PSMs at a chosen confidence threshold. (ZIP) [file pcbi.1007082.s001.zip › DART-ID_SCoPE-MS_Report/figures/alignment_63_180402S_QC_SQC72C8.png]

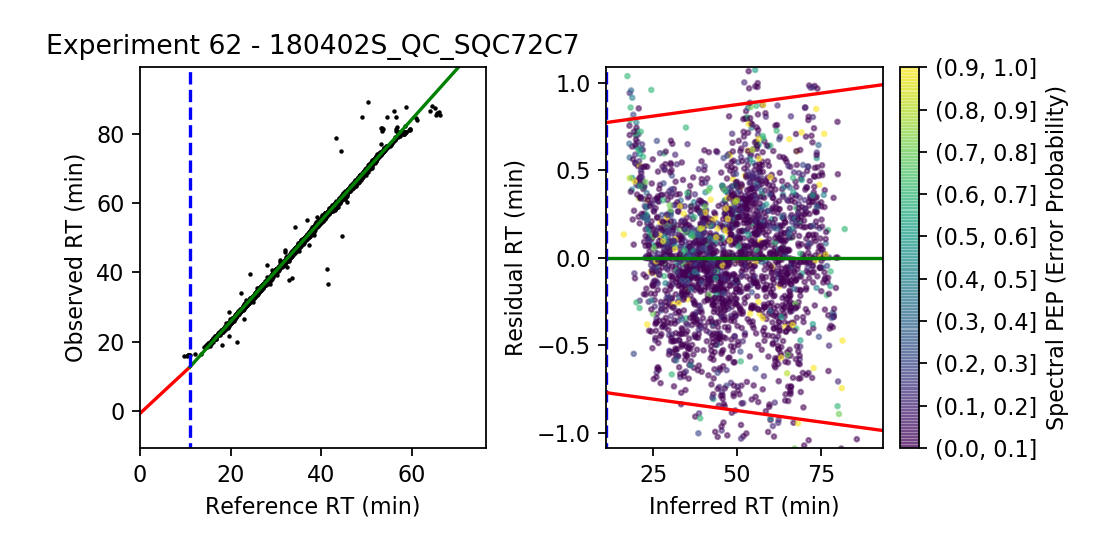

Supplement: S1 File — A optional HTML report generated by the dart_id Python script. The report gives a summary of the alignment for each experiment, as well as a broad overview of the performance of the run as a whole, by showing aggregate increases in PSMs at a chosen confidence threshold. (ZIP) [file pcbi.1007082.s001.zip › DART-ID_SCoPE-MS_Report/figures/alignment_62_180402S_QC_SQC72C7.png]

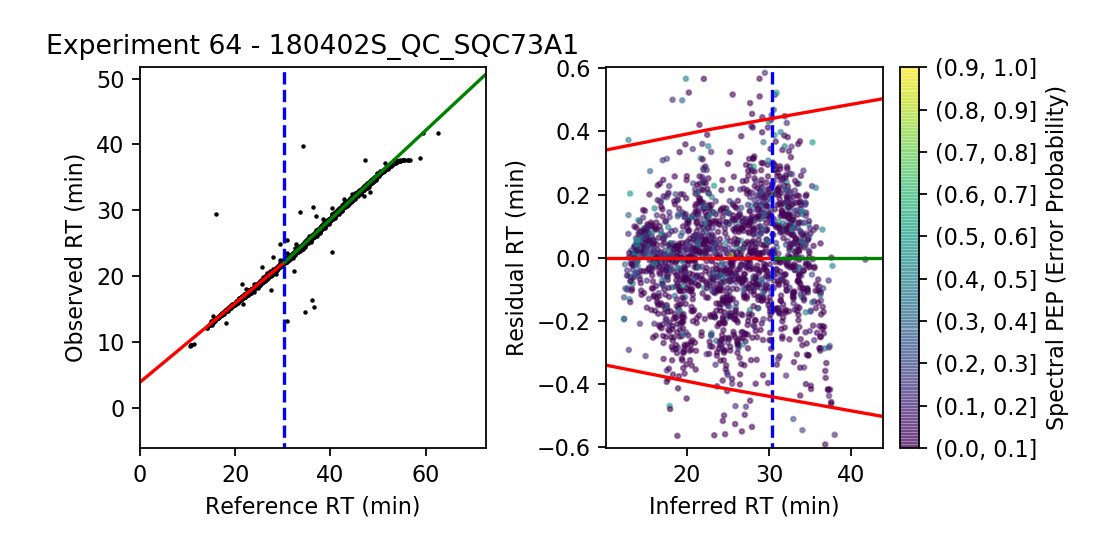

Supplement: S1 File — A optional HTML report generated by the dart_id Python script. The report gives a summary of the alignment for each experiment, as well as a broad overview of the performance of the run as a whole, by showing aggregate increases in PSMs at a chosen confidence threshold. (ZIP) [file pcbi.1007082.s001.zip › DART-ID_SCoPE-MS_Report/figures/alignment_64_180402S_QC_SQC73A1.png]

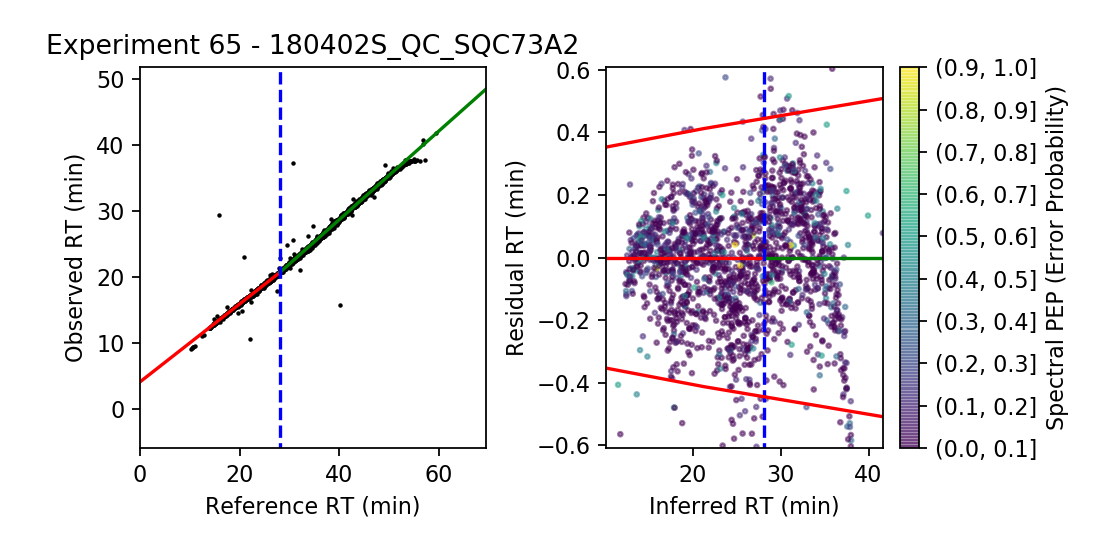

Supplement: S1 File — A optional HTML report generated by the dart_id Python script. The report gives a summary of the alignment for each experiment, as well as a broad overview of the performance of the run as a whole, by showing aggregate increases in PSMs at a chosen confidence threshold. (ZIP) [file pcbi.1007082.s001.zip › DART-ID_SCoPE-MS_Report/figures/alignment_65_180402S_QC_SQC73A2.png]

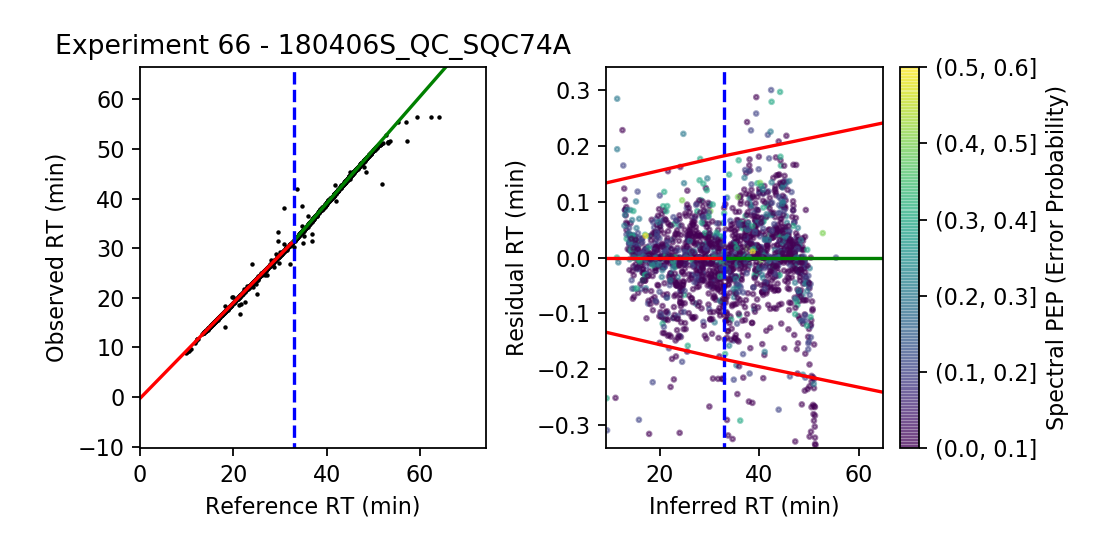

Supplement: S1 File — A optional HTML report generated by the dart_id Python script. The report gives a summary of the alignment for each experiment, as well as a broad overview of the performance of the run as a whole, by showing aggregate increases in PSMs at a chosen confidence threshold. (ZIP) [file pcbi.1007082.s001.zip › DART-ID_SCoPE-MS_Report/figures/alignment_66_180406S_QC_SQC74A.png]

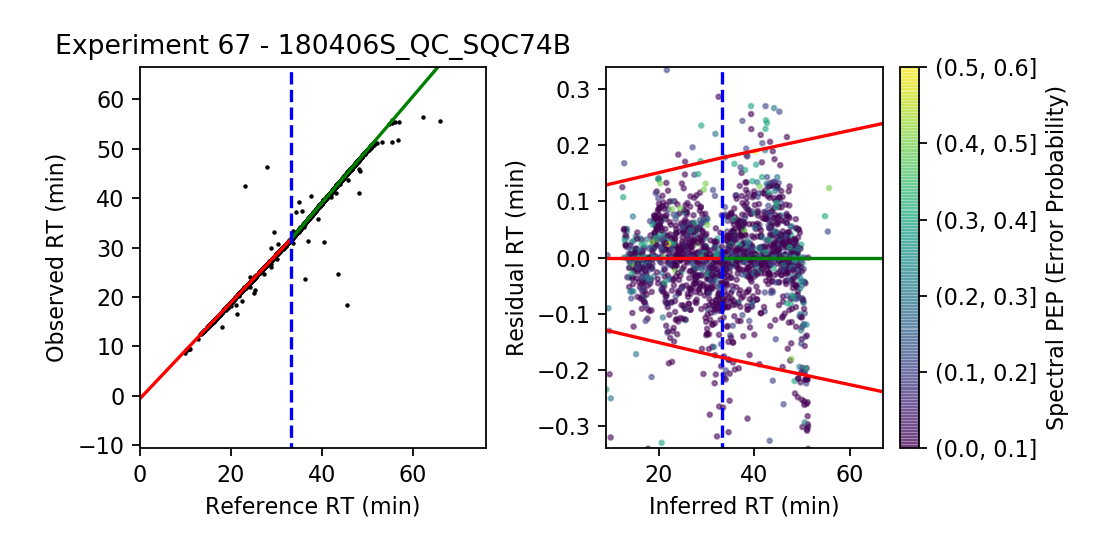

Supplement: S1 File — A optional HTML report generated by the dart_id Python script. The report gives a summary of the alignment for each experiment, as well as a broad overview of the performance of the run as a whole, by showing aggregate increases in PSMs at a chosen confidence threshold. (ZIP) [file pcbi.1007082.s001.zip › DART-ID_SCoPE-MS_Report/figures/alignment_67_180406S_QC_SQC74B.png]

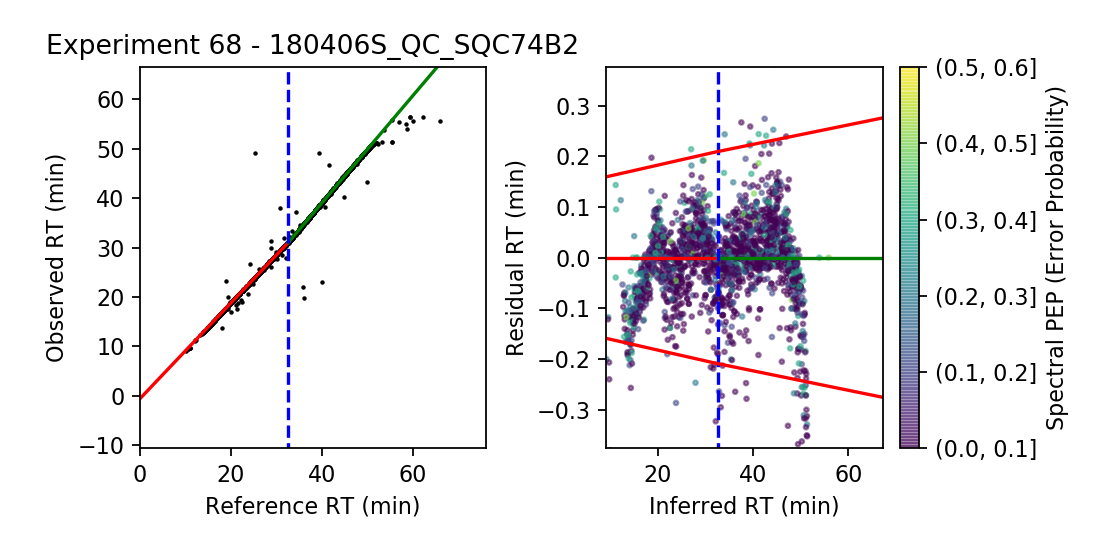

Supplement: S1 File — A optional HTML report generated by the dart_id Python script. The report gives a summary of the alignment for each experiment, as well as a broad overview of the performance of the run as a whole, by showing aggregate increases in PSMs at a chosen confidence threshold. (ZIP) [file pcbi.1007082.s001.zip › DART-ID_SCoPE-MS_Report/figures/alignment_68_180406S_QC_SQC74B2.png]

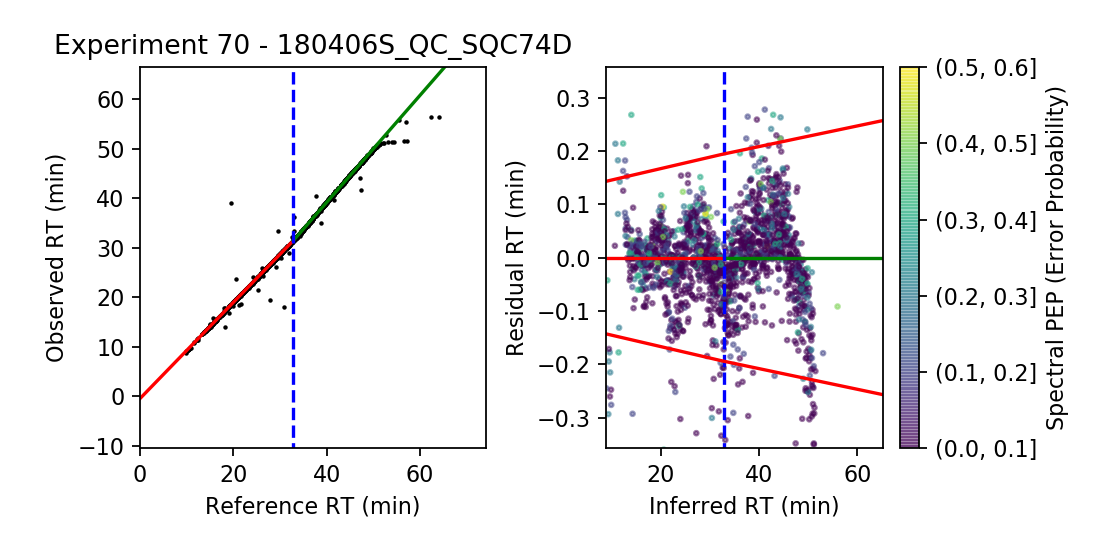

Supplement: S1 File — A optional HTML report generated by the dart_id Python script. The report gives a summary of the alignment for each experiment, as well as a broad overview of the performance of the run as a whole, by showing aggregate increases in PSMs at a chosen confidence threshold. (ZIP) [file pcbi.1007082.s001.zip › DART-ID_SCoPE-MS_Report/figures/alignment_70_180406S_QC_SQC74D.png]

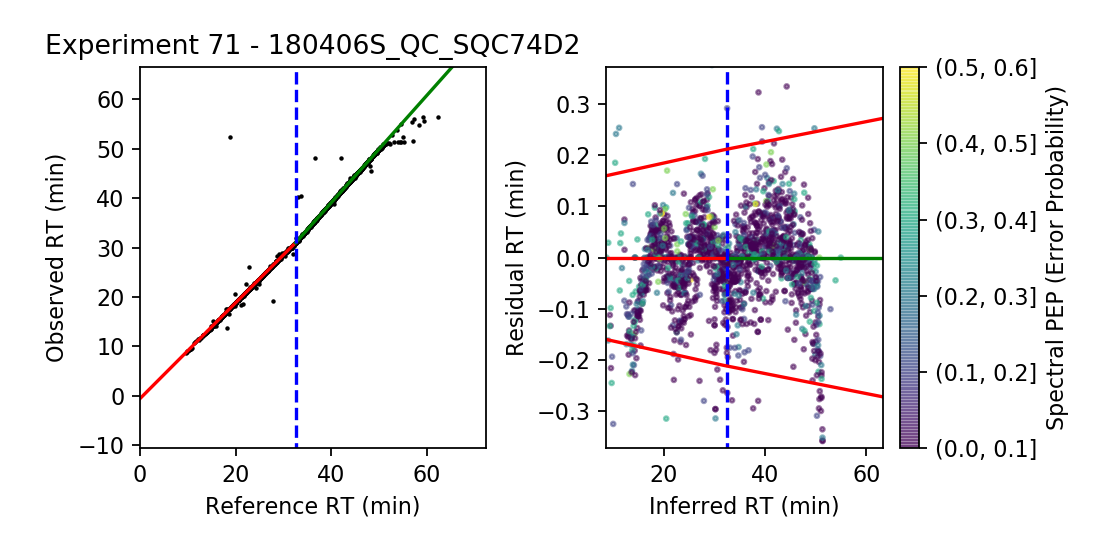

Supplement: S1 File — A optional HTML report generated by the dart_id Python script. The report gives a summary of the alignment for each experiment, as well as a broad overview of the performance of the run as a whole, by showing aggregate increases in PSMs at a chosen confidence threshold. (ZIP) [file pcbi.1007082.s001.zip › DART-ID_SCoPE-MS_Report/figures/alignment_71_180406S_QC_SQC74D2.png]

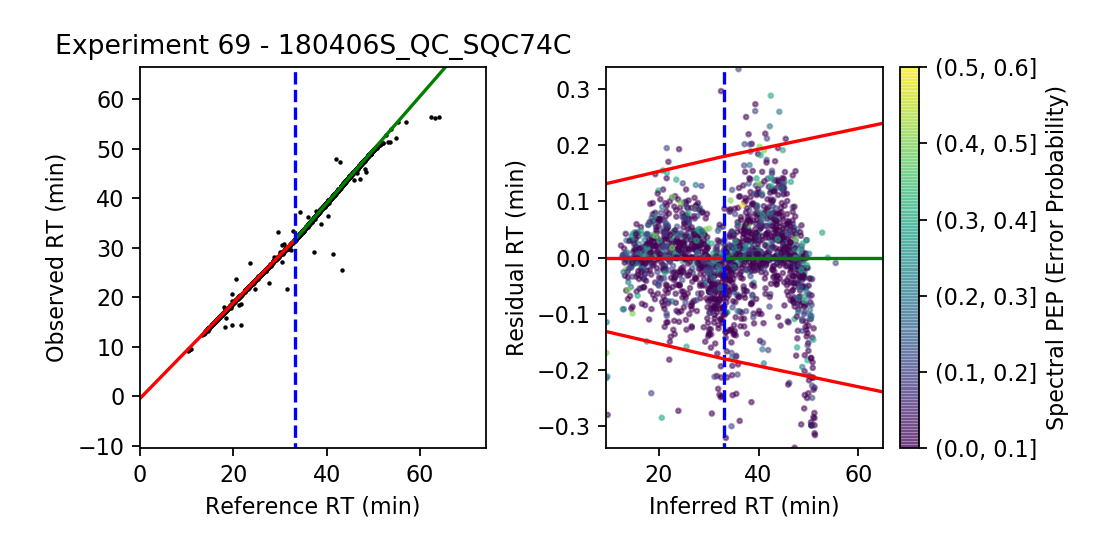

Supplement: S1 File — A optional HTML report generated by the dart_id Python script. The report gives a summary of the alignment for each experiment, as well as a broad overview of the performance of the run as a whole, by showing aggregate increases in PSMs at a chosen confidence threshold. (ZIP) [file pcbi.1007082.s001.zip › DART-ID_SCoPE-MS_Report/figures/alignment_69_180406S_QC_SQC74C.png]

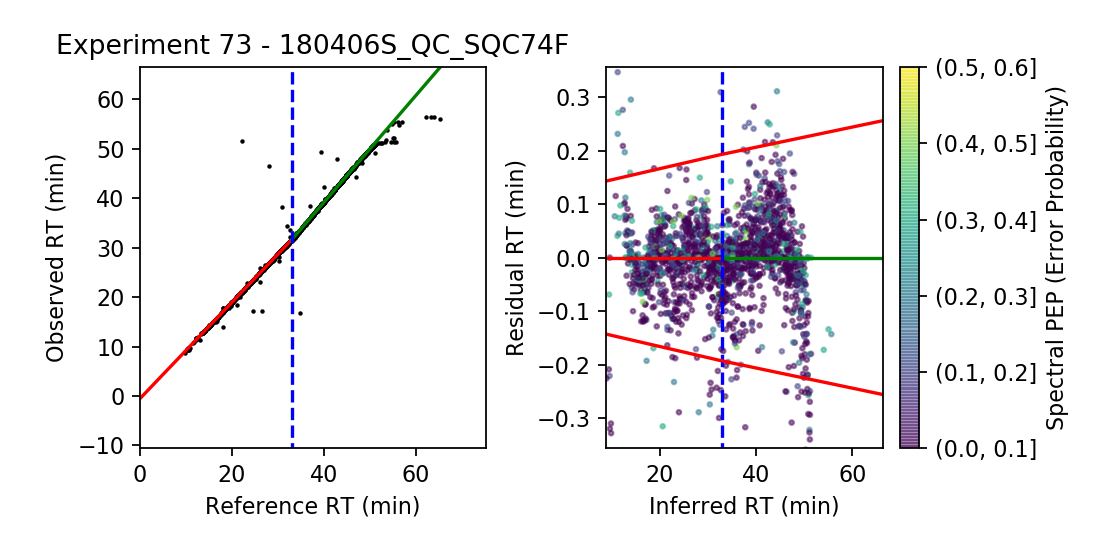

Supplement: S1 File — A optional HTML report generated by the dart_id Python script. The report gives a summary of the alignment for each experiment, as well as a broad overview of the performance of the run as a whole, by showing aggregate increases in PSMs at a chosen confidence threshold. (ZIP) [file pcbi.1007082.s001.zip › DART-ID_SCoPE-MS_Report/figures/alignment_73_180406S_QC_SQC74F.png]

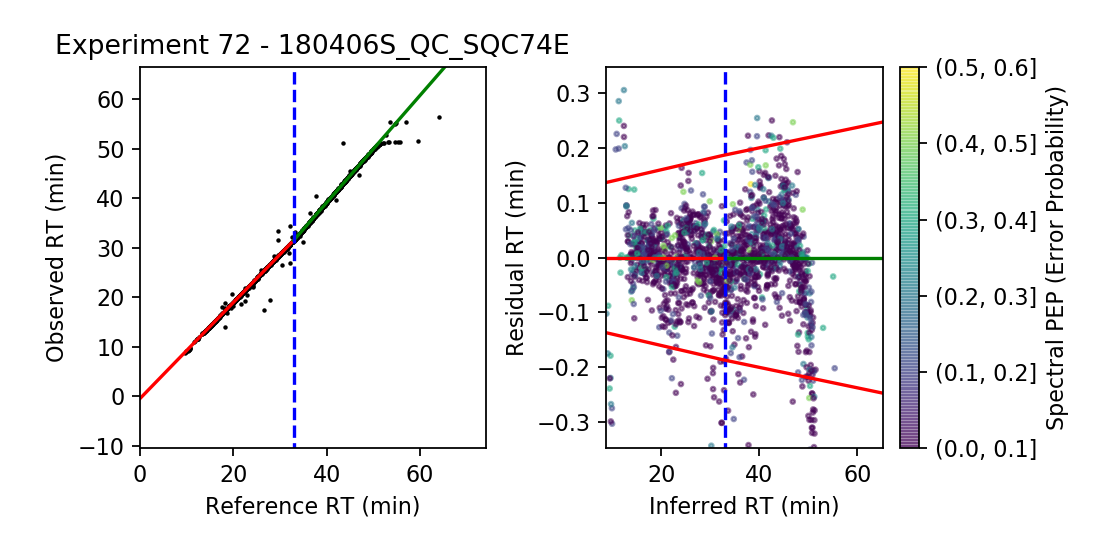

Supplement: S1 File — A optional HTML report generated by the dart_id Python script. The report gives a summary of the alignment for each experiment, as well as a broad overview of the performance of the run as a whole, by showing aggregate increases in PSMs at a chosen confidence threshold. (ZIP) [file pcbi.1007082.s001.zip › DART-ID_SCoPE-MS_Report/figures/alignment_72_180406S_QC_SQC74E.png]

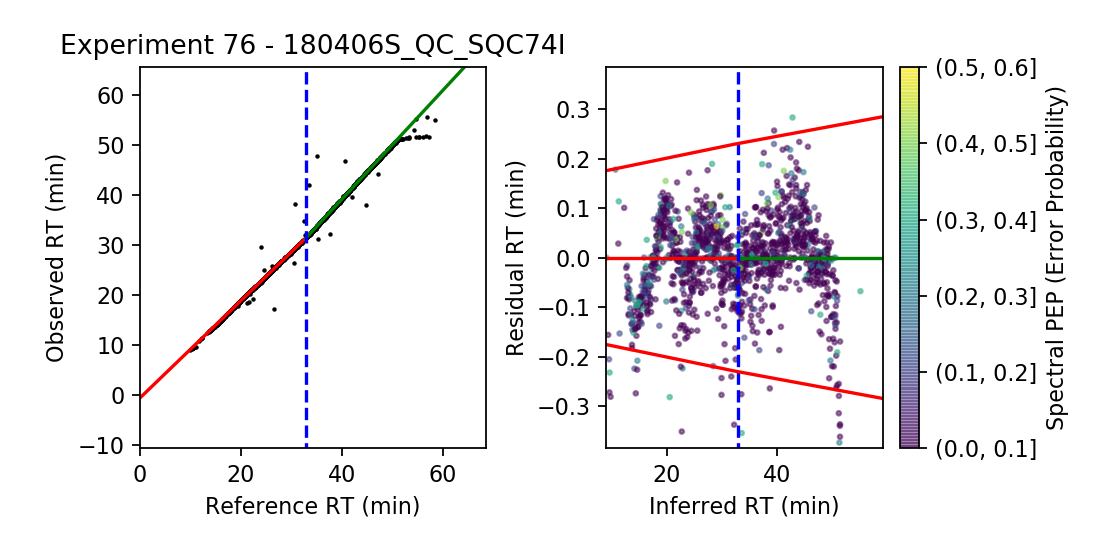

Supplement: S1 File — A optional HTML report generated by the dart_id Python script. The report gives a summary of the alignment for each experiment, as well as a broad overview of the performance of the run as a whole, by showing aggregate increases in PSMs at a chosen confidence threshold. (ZIP) [file pcbi.1007082.s001.zip › DART-ID_SCoPE-MS_Report/figures/alignment_76_180406S_QC_SQC74I.png]

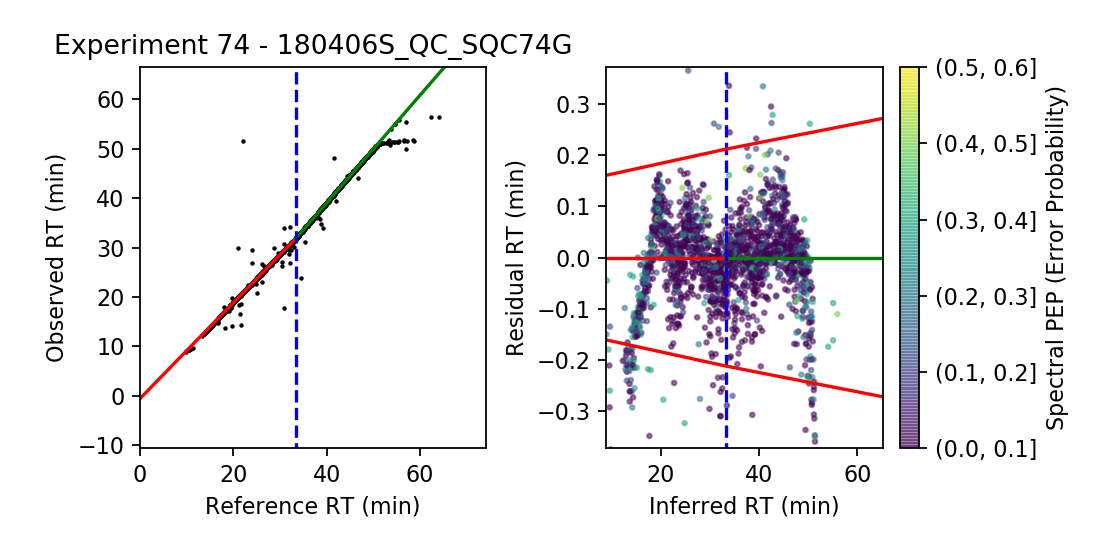

Supplement: S1 File — A optional HTML report generated by the dart_id Python script. The report gives a summary of the alignment for each experiment, as well as a broad overview of the performance of the run as a whole, by showing aggregate increases in PSMs at a chosen confidence threshold. (ZIP) [file pcbi.1007082.s001.zip › DART-ID_SCoPE-MS_Report/figures/alignment_74_180406S_QC_SQC74G.png]

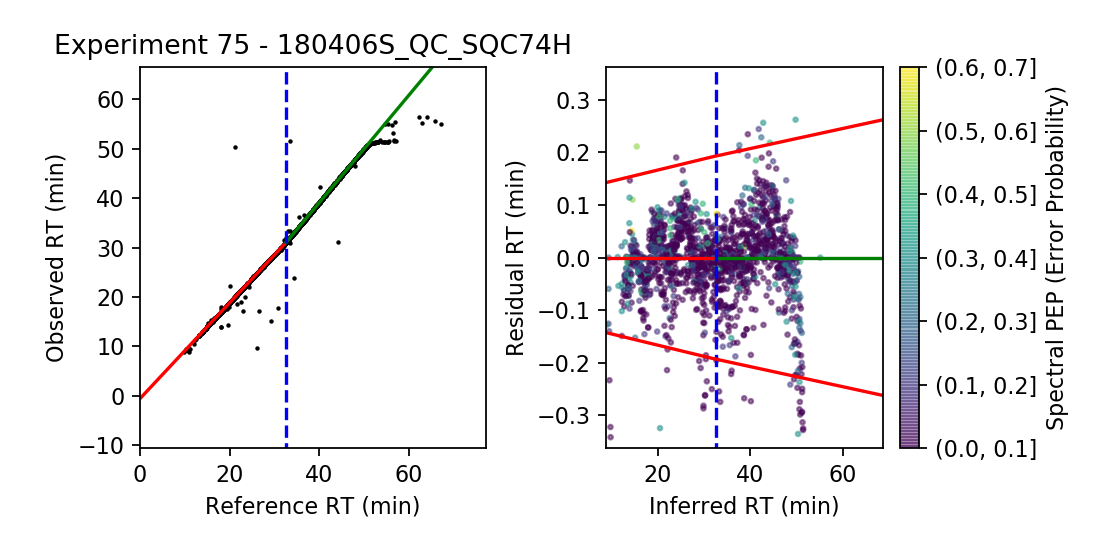

Supplement: S1 File — A optional HTML report generated by the dart_id Python script. The report gives a summary of the alignment for each experiment, as well as a broad overview of the performance of the run as a whole, by showing aggregate increases in PSMs at a chosen confidence threshold. (ZIP) [file pcbi.1007082.s001.zip › DART-ID_SCoPE-MS_Report/figures/alignment_75_180406S_QC_SQC74H.png]

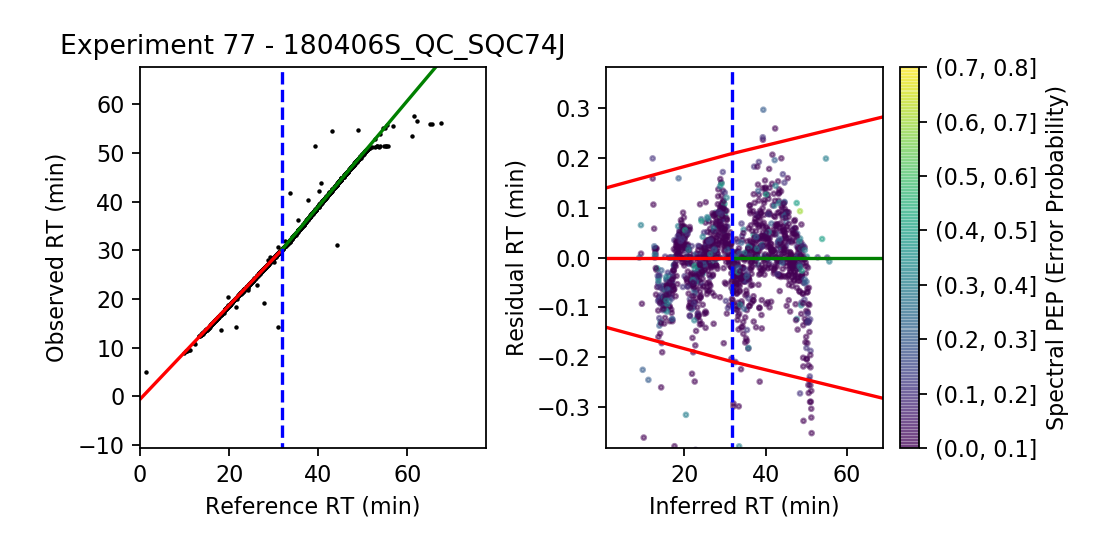

Supplement: S1 File — A optional HTML report generated by the dart_id Python script. The report gives a summary of the alignment for each experiment, as well as a broad overview of the performance of the run as a whole, by showing aggregate increases in PSMs at a chosen confidence threshold. (ZIP) [file pcbi.1007082.s001.zip › DART-ID_SCoPE-MS_Report/figures/alignment_77_180406S_QC_SQC74J.png]

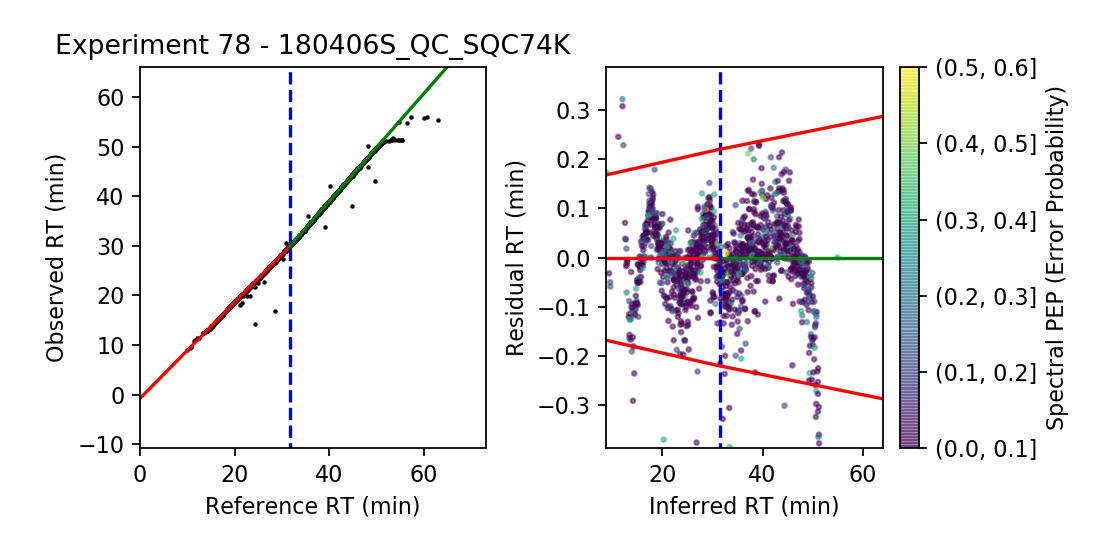

Supplement: S1 File — A optional HTML report generated by the dart_id Python script. The report gives a summary of the alignment for each experiment, as well as a broad overview of the performance of the run as a whole, by showing aggregate increases in PSMs at a chosen confidence threshold. (ZIP) [file pcbi.1007082.s001.zip › DART-ID_SCoPE-MS_Report/figures/alignment_78_180406S_QC_SQC74K.png]

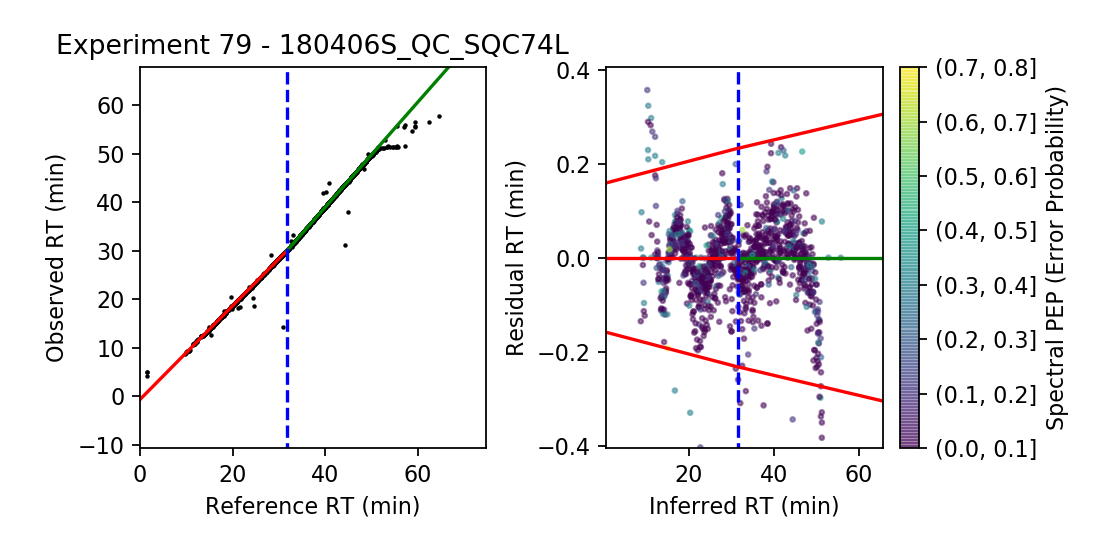

Supplement: S1 File — A optional HTML report generated by the dart_id Python script. The report gives a summary of the alignment for each experiment, as well as a broad overview of the performance of the run as a whole, by showing aggregate increases in PSMs at a chosen confidence threshold. (ZIP) [file pcbi.1007082.s001.zip › DART-ID_SCoPE-MS_Report/figures/alignment_79_180406S_QC_SQC74L.png]

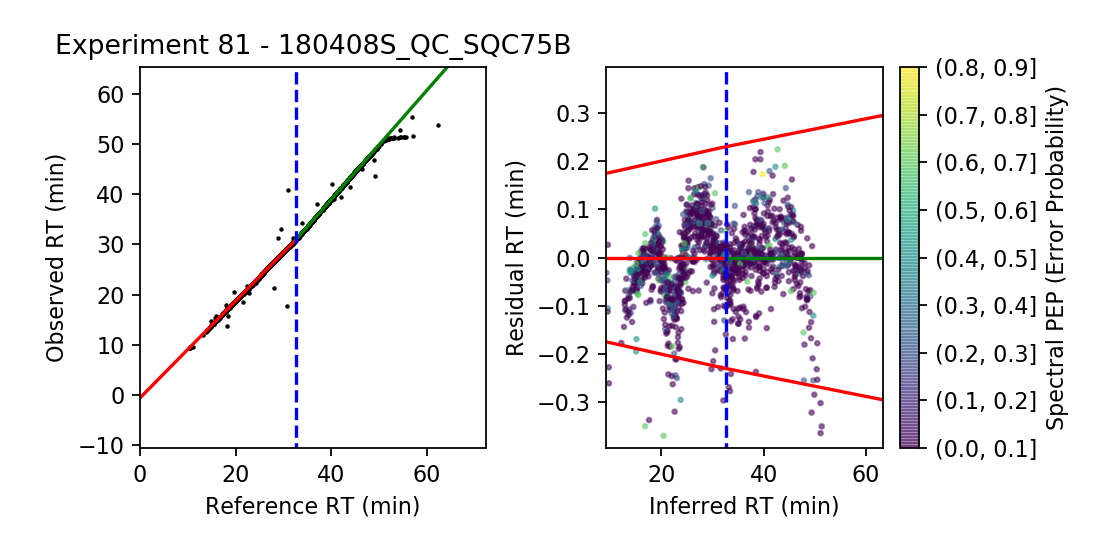

Supplement: S1 File — A optional HTML report generated by the dart_id Python script. The report gives a summary of the alignment for each experiment, as well as a broad overview of the performance of the run as a whole, by showing aggregate increases in PSMs at a chosen confidence threshold. (ZIP) [file pcbi.1007082.s001.zip › DART-ID_SCoPE-MS_Report/figures/alignment_81_180408S_QC_SQC75B.png]

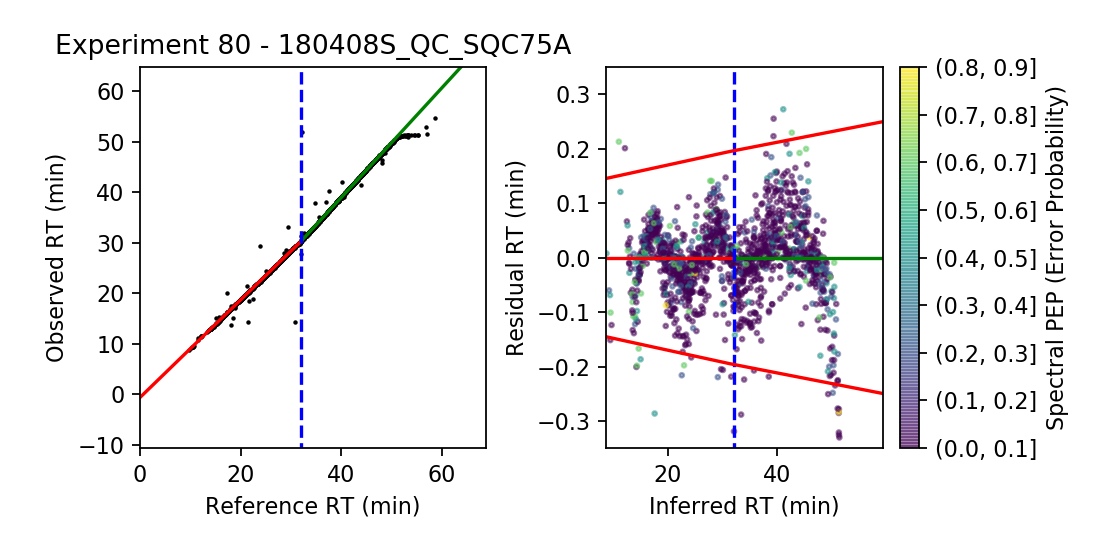

Supplement: S1 File — A optional HTML report generated by the dart_id Python script. The report gives a summary of the alignment for each experiment, as well as a broad overview of the performance of the run as a whole, by showing aggregate increases in PSMs at a chosen confidence threshold. (ZIP) [file pcbi.1007082.s001.zip › DART-ID_SCoPE-MS_Report/figures/alignment_80_180408S_QC_SQC75A.png]

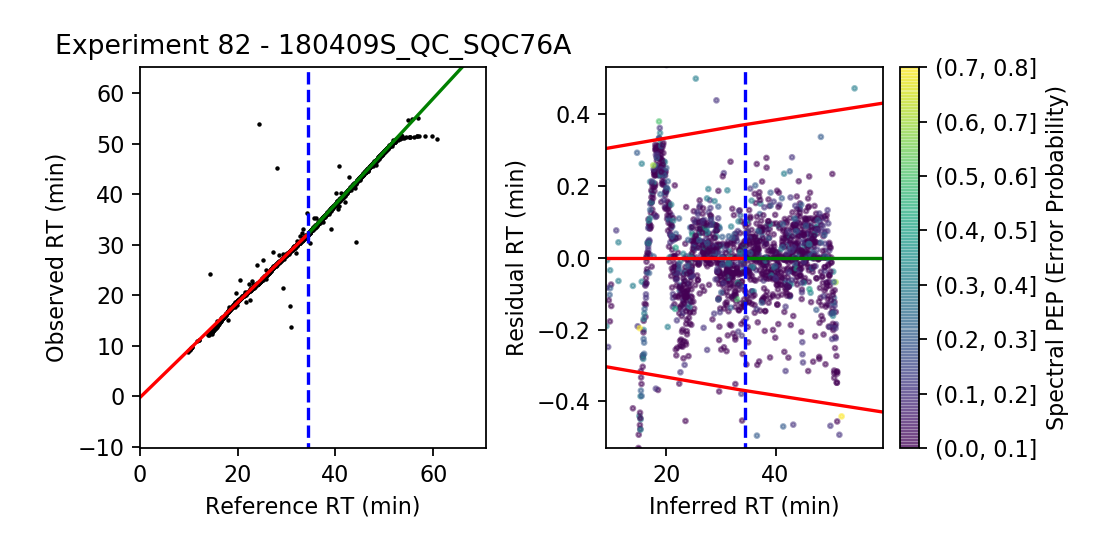

Supplement: S1 File — A optional HTML report generated by the dart_id Python script. The report gives a summary of the alignment for each experiment, as well as a broad overview of the performance of the run as a whole, by showing aggregate increases in PSMs at a chosen confidence threshold. (ZIP) [file pcbi.1007082.s001.zip › DART-ID_SCoPE-MS_Report/figures/alignment_82_180409S_QC_SQC76A.png]

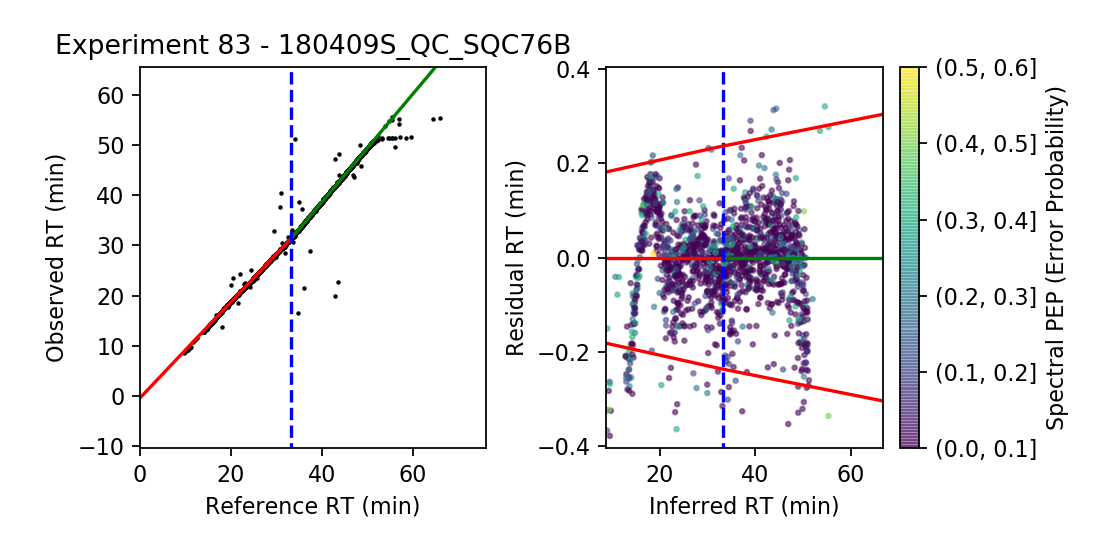

Supplement: S1 File — A optional HTML report generated by the dart_id Python script. The report gives a summary of the alignment for each experiment, as well as a broad overview of the performance of the run as a whole, by showing aggregate increases in PSMs at a chosen confidence threshold. (ZIP) [file pcbi.1007082.s001.zip › DART-ID_SCoPE-MS_Report/figures/alignment_83_180409S_QC_SQC76B.png]

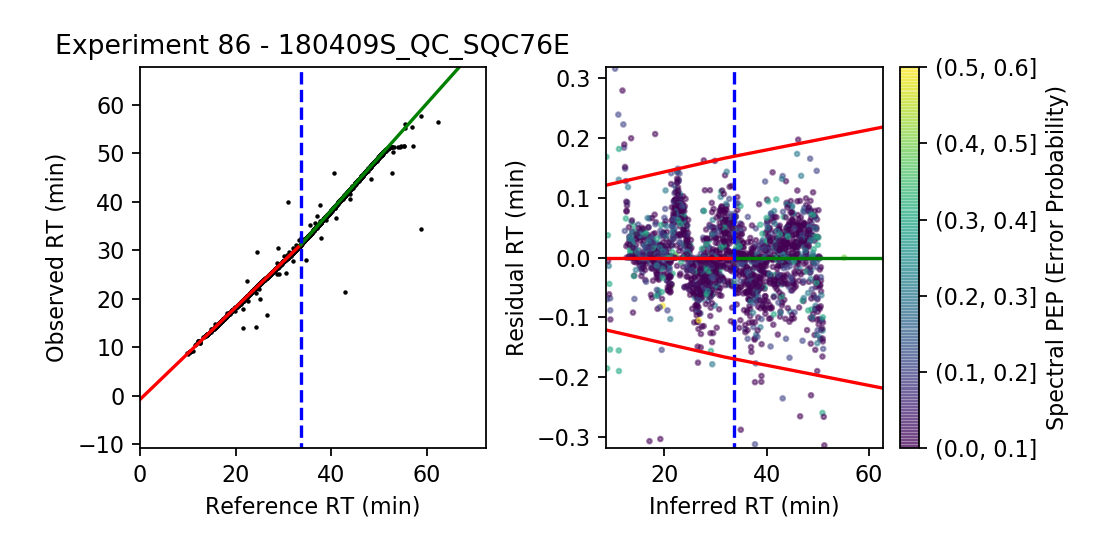

Supplement: S1 File — A optional HTML report generated by the dart_id Python script. The report gives a summary of the alignment for each experiment, as well as a broad overview of the performance of the run as a whole, by showing aggregate increases in PSMs at a chosen confidence threshold. (ZIP) [file pcbi.1007082.s001.zip › DART-ID_SCoPE-MS_Report/figures/alignment_86_180409S_QC_SQC76E.png]

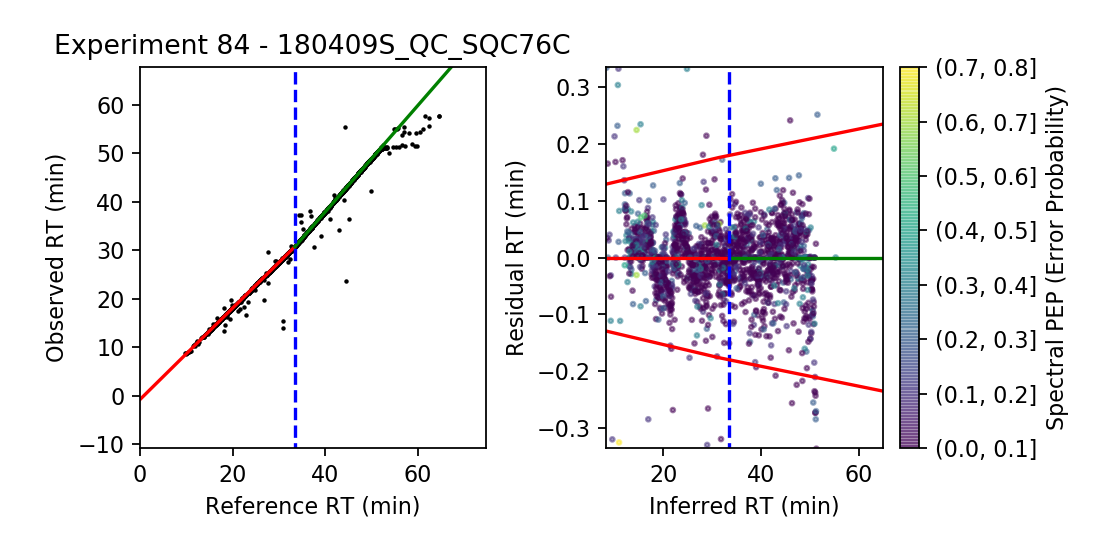

Supplement: S1 File — A optional HTML report generated by the dart_id Python script. The report gives a summary of the alignment for each experiment, as well as a broad overview of the performance of the run as a whole, by showing aggregate increases in PSMs at a chosen confidence threshold. (ZIP) [file pcbi.1007082.s001.zip › DART-ID_SCoPE-MS_Report/figures/alignment_84_180409S_QC_SQC76C.png]

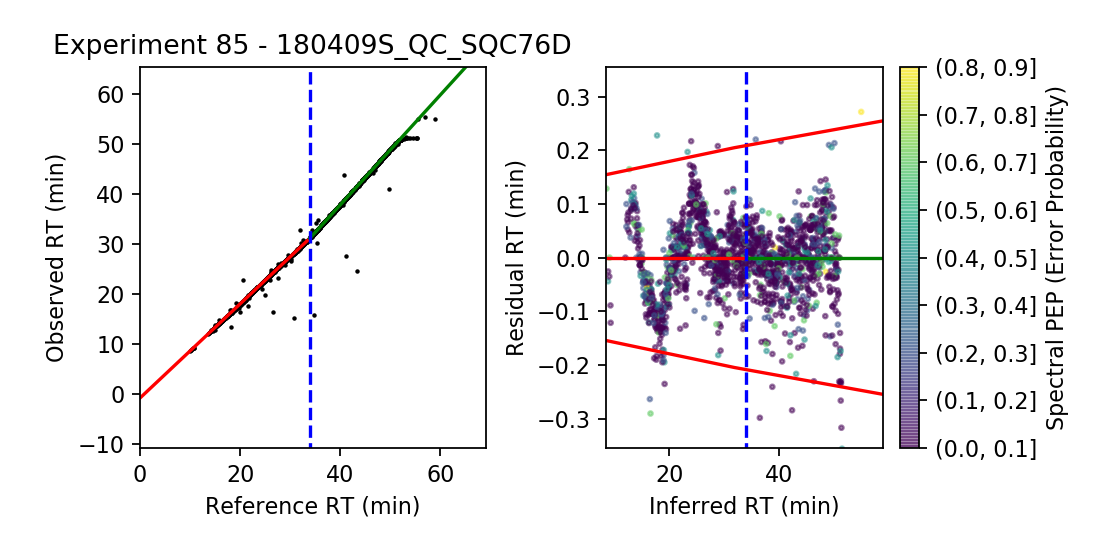

Supplement: S1 File — A optional HTML report generated by the dart_id Python script. The report gives a summary of the alignment for each experiment, as well as a broad overview of the performance of the run as a whole, by showing aggregate increases in PSMs at a chosen confidence threshold. (ZIP) [file pcbi.1007082.s001.zip › DART-ID_SCoPE-MS_Report/figures/alignment_85_180409S_QC_SQC76D.png]

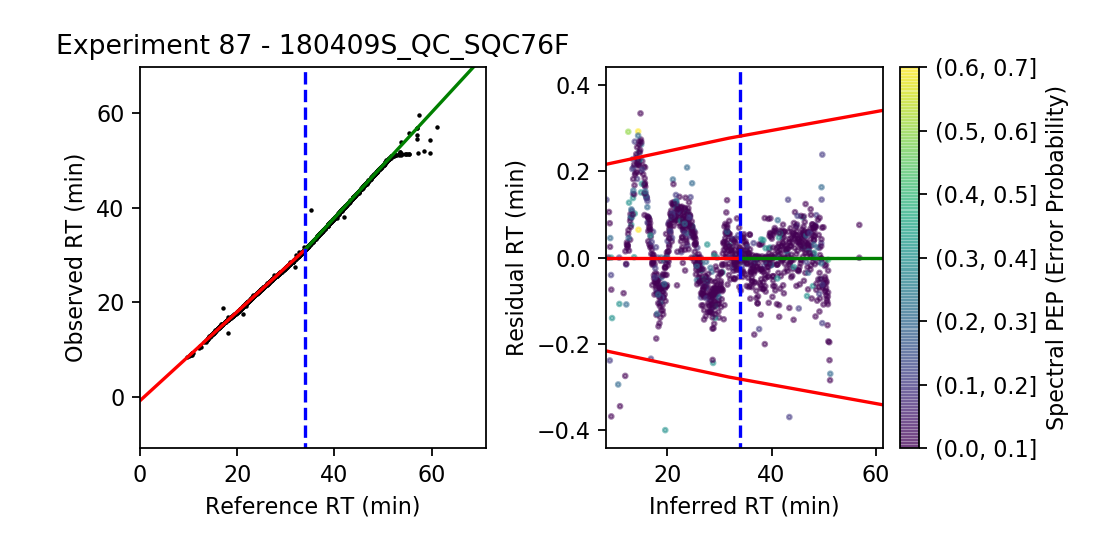

Supplement: S1 File — A optional HTML report generated by the dart_id Python script. The report gives a summary of the alignment for each experiment, as well as a broad overview of the performance of the run as a whole, by showing aggregate increases in PSMs at a chosen confidence threshold. (ZIP) [file pcbi.1007082.s001.zip › DART-ID_SCoPE-MS_Report/figures/alignment_87_180409S_QC_SQC76F.png]

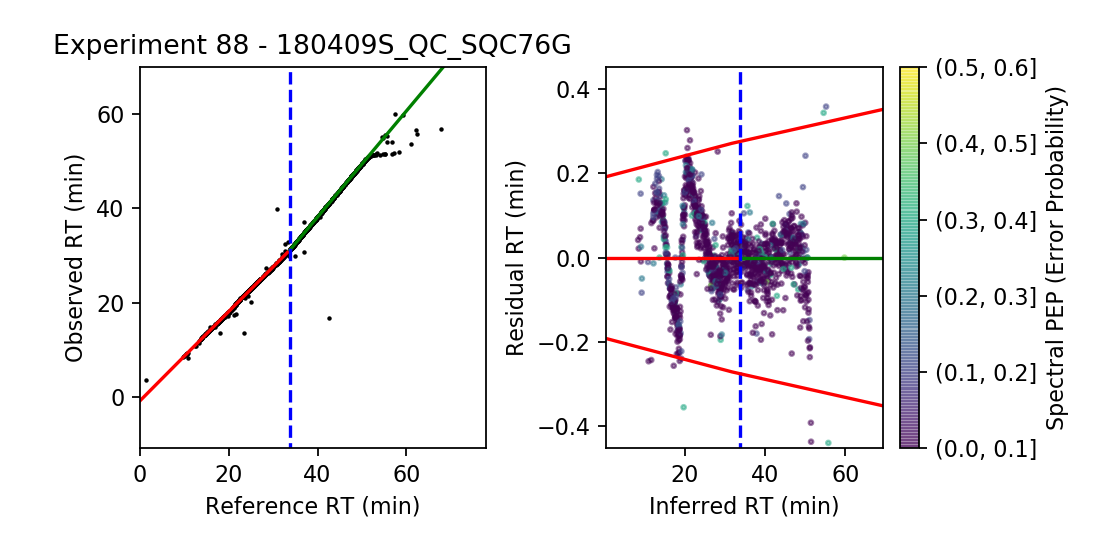

Supplement: S1 File — A optional HTML report generated by the dart_id Python script. The report gives a summary of the alignment for each experiment, as well as a broad overview of the performance of the run as a whole, by showing aggregate increases in PSMs at a chosen confidence threshold. (ZIP) [file pcbi.1007082.s001.zip › DART-ID_SCoPE-MS_Report/figures/alignment_88_180409S_QC_SQC76G.png]

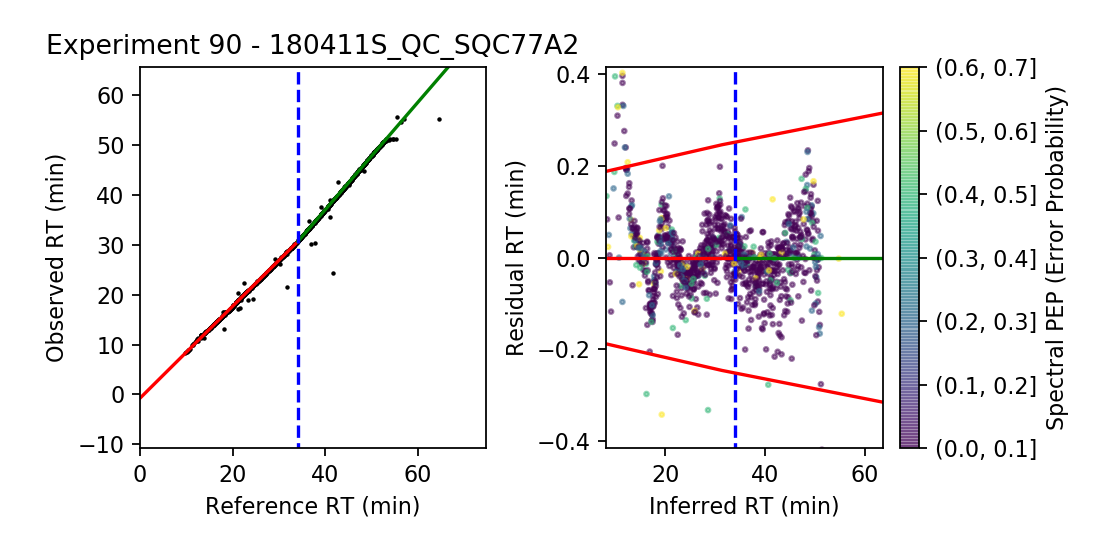

Supplement: S1 File — A optional HTML report generated by the dart_id Python script. The report gives a summary of the alignment for each experiment, as well as a broad overview of the performance of the run as a whole, by showing aggregate increases in PSMs at a chosen confidence threshold. (ZIP) [file pcbi.1007082.s001.zip › DART-ID_SCoPE-MS_Report/figures/alignment_90_180411S_QC_SQC77A2.png]

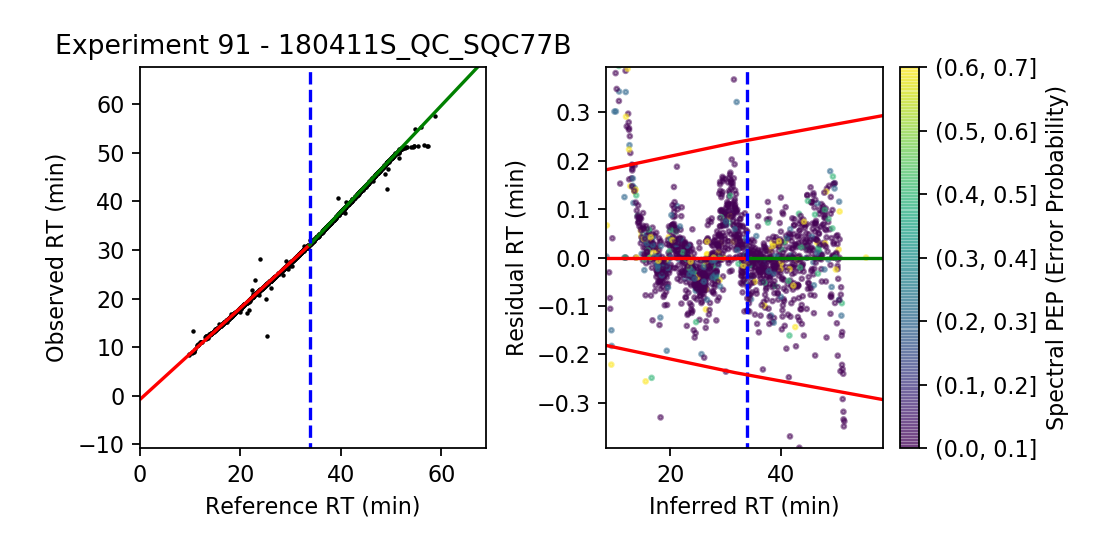

Supplement: S1 File — A optional HTML report generated by the dart_id Python script. The report gives a summary of the alignment for each experiment, as well as a broad overview of the performance of the run as a whole, by showing aggregate increases in PSMs at a chosen confidence threshold. (ZIP) [file pcbi.1007082.s001.zip › DART-ID_SCoPE-MS_Report/figures/alignment_91_180411S_QC_SQC77B.png]

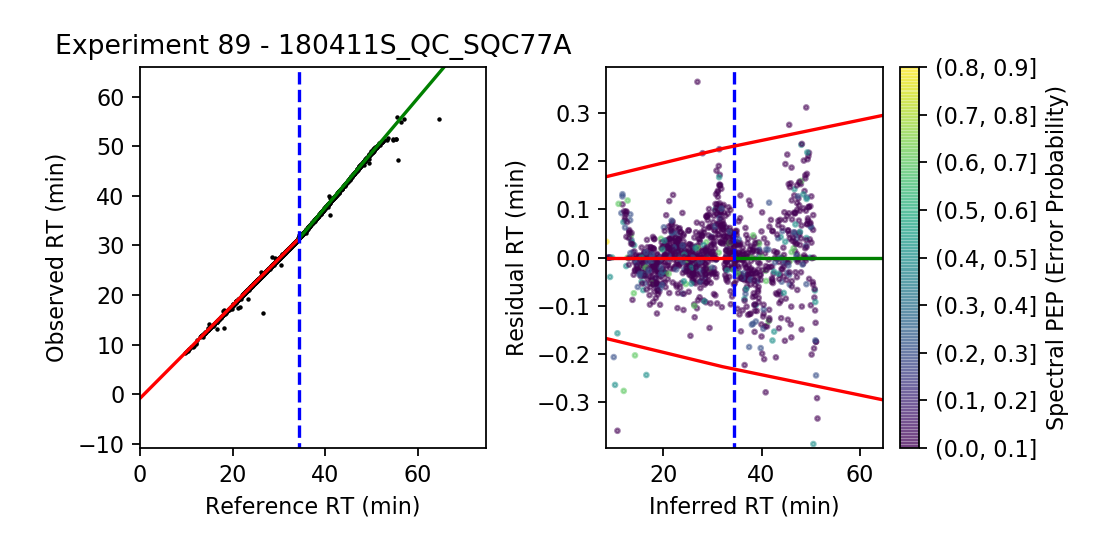

Supplement: S1 File — A optional HTML report generated by the dart_id Python script. The report gives a summary of the alignment for each experiment, as well as a broad overview of the performance of the run as a whole, by showing aggregate increases in PSMs at a chosen confidence threshold. (ZIP) [file pcbi.1007082.s001.zip › DART-ID_SCoPE-MS_Report/figures/alignment_89_180411S_QC_SQC77A.png]

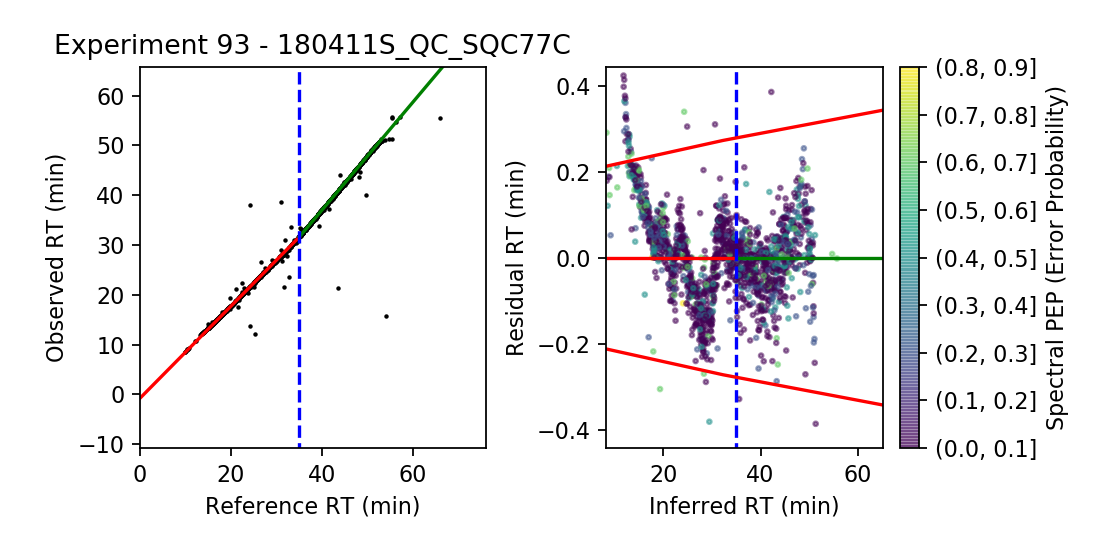

Supplement: S1 File — A optional HTML report generated by the dart_id Python script. The report gives a summary of the alignment for each experiment, as well as a broad overview of the performance of the run as a whole, by showing aggregate increases in PSMs at a chosen confidence threshold. (ZIP) [file pcbi.1007082.s001.zip › DART-ID_SCoPE-MS_Report/figures/alignment_93_180411S_QC_SQC77C.png]

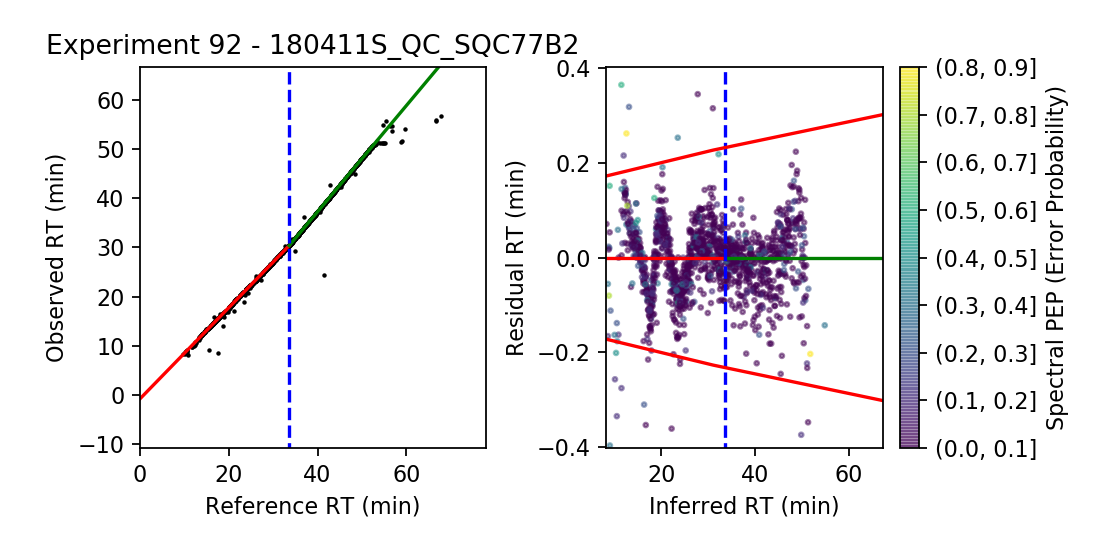

Supplement: S1 File — A optional HTML report generated by the dart_id Python script. The report gives a summary of the alignment for each experiment, as well as a broad overview of the performance of the run as a whole, by showing aggregate increases in PSMs at a chosen confidence threshold. (ZIP) [file pcbi.1007082.s001.zip › DART-ID_SCoPE-MS_Report/figures/alignment_92_180411S_QC_SQC77B2.png]

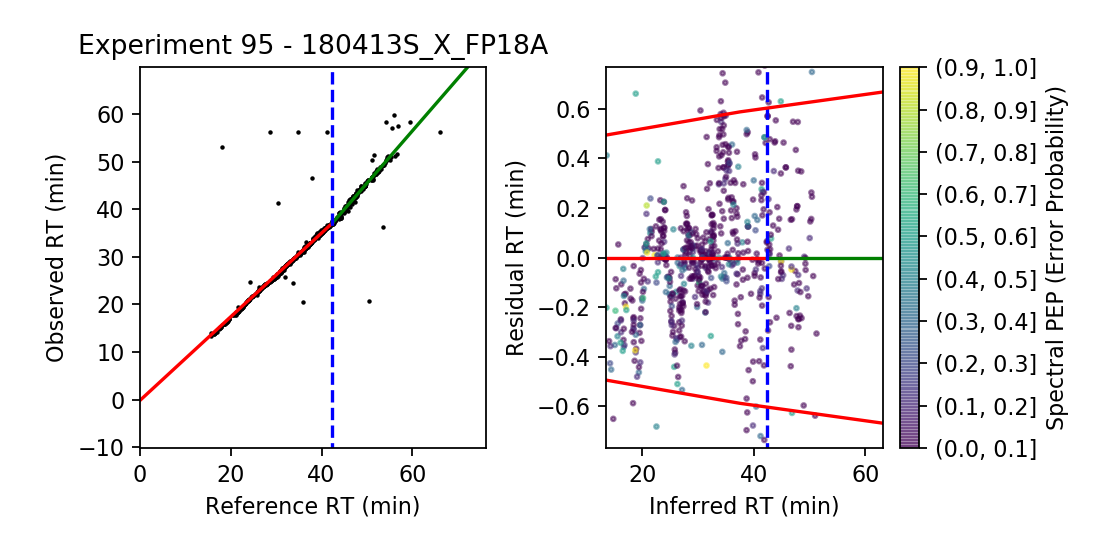

Supplement: S1 File — A optional HTML report generated by the dart_id Python script. The report gives a summary of the alignment for each experiment, as well as a broad overview of the performance of the run as a whole, by showing aggregate increases in PSMs at a chosen confidence threshold. (ZIP) [file pcbi.1007082.s001.zip › DART-ID_SCoPE-MS_Report/figures/alignment_95_180413S_X_FP18A.png]

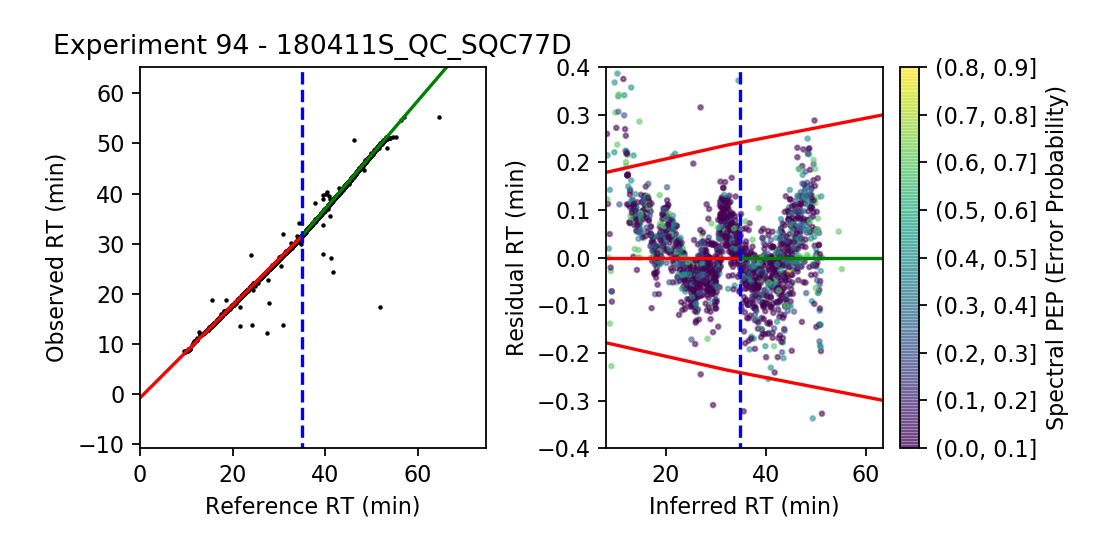

Supplement: S1 File — A optional HTML report generated by the dart_id Python script. The report gives a summary of the alignment for each experiment, as well as a broad overview of the performance of the run as a whole, by showing aggregate increases in PSMs at a chosen confidence threshold. (ZIP) [file pcbi.1007082.s001.zip › DART-ID_SCoPE-MS_Report/figures/alignment_94_180411S_QC_SQC77D.png]

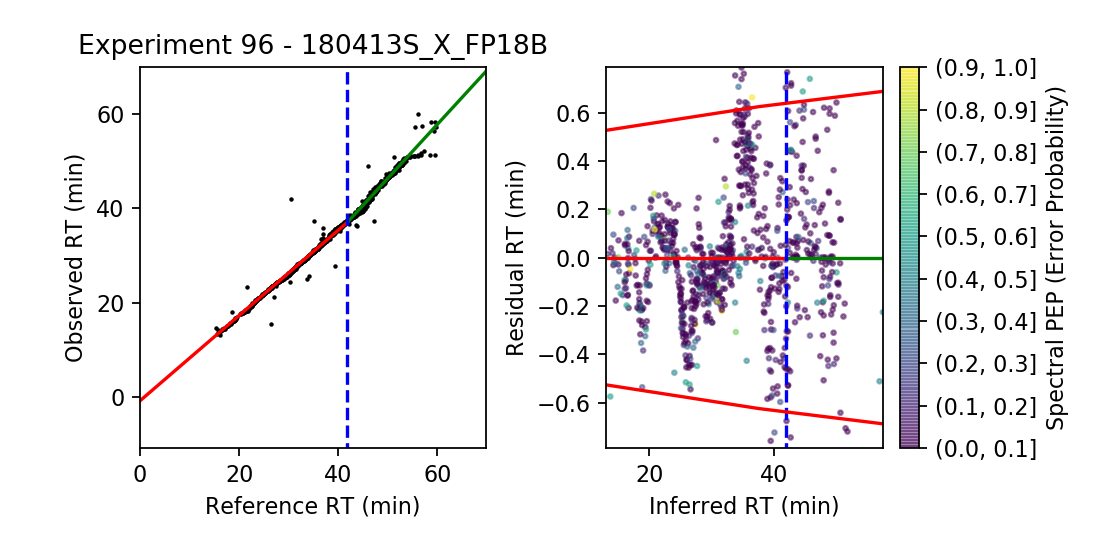

Supplement: S1 File — A optional HTML report generated by the dart_id Python script. The report gives a summary of the alignment for each experiment, as well as a broad overview of the performance of the run as a whole, by showing aggregate increases in PSMs at a chosen confidence threshold. (ZIP) [file pcbi.1007082.s001.zip › DART-ID_SCoPE-MS_Report/figures/alignment_96_180413S_X_FP18B.png]

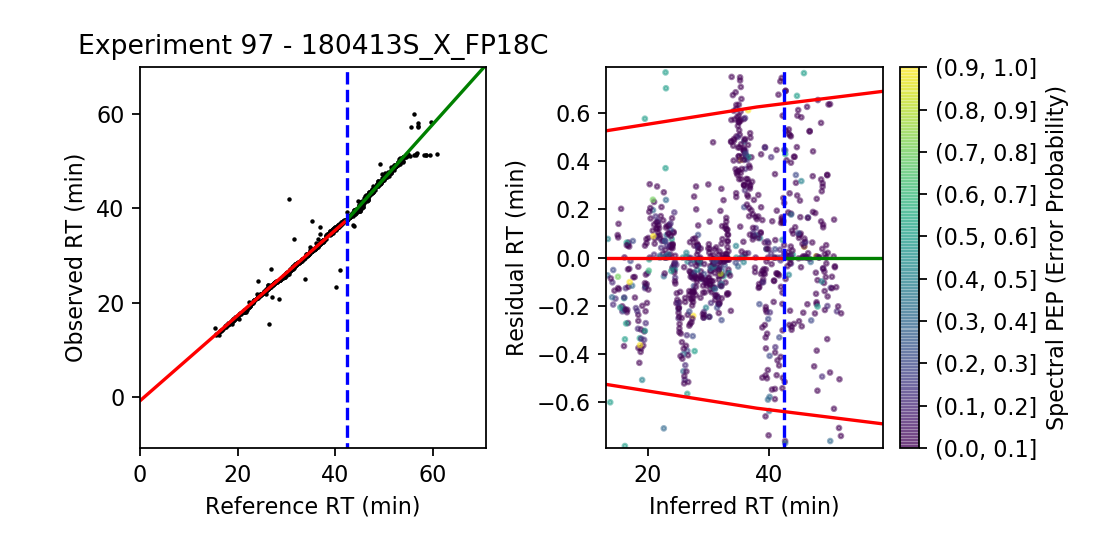

Supplement: S1 File — A optional HTML report generated by the dart_id Python script. The report gives a summary of the alignment for each experiment, as well as a broad overview of the performance of the run as a whole, by showing aggregate increases in PSMs at a chosen confidence threshold. (ZIP) [file pcbi.1007082.s001.zip › DART-ID_SCoPE-MS_Report/figures/alignment_97_180413S_X_FP18C.png]

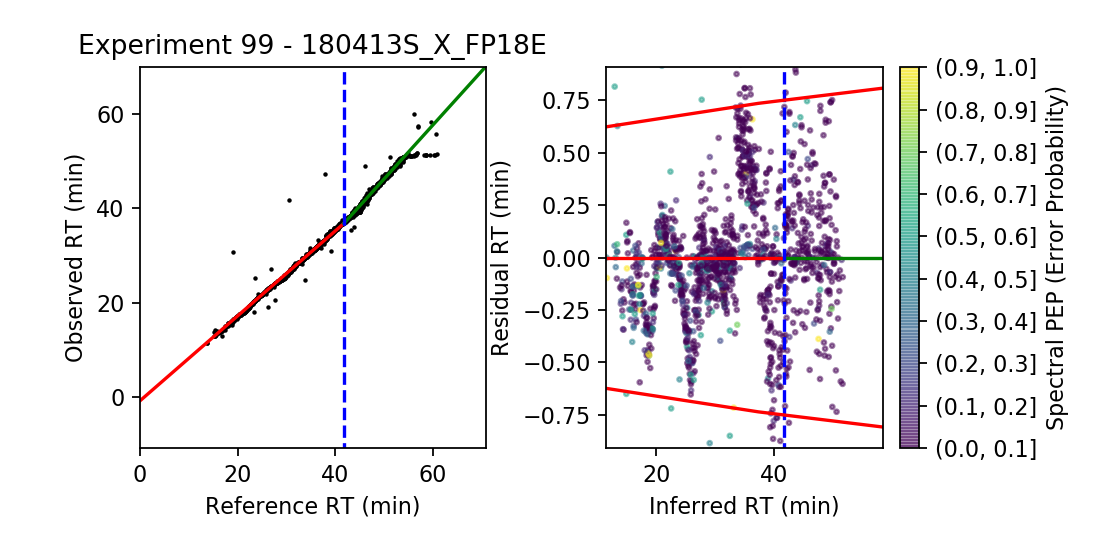

Supplement: S1 File — A optional HTML report generated by the dart_id Python script. The report gives a summary of the alignment for each experiment, as well as a broad overview of the performance of the run as a whole, by showing aggregate increases in PSMs at a chosen confidence threshold. (ZIP) [file pcbi.1007082.s001.zip › DART-ID_SCoPE-MS_Report/figures/alignment_99_180413S_X_FP18E.png]

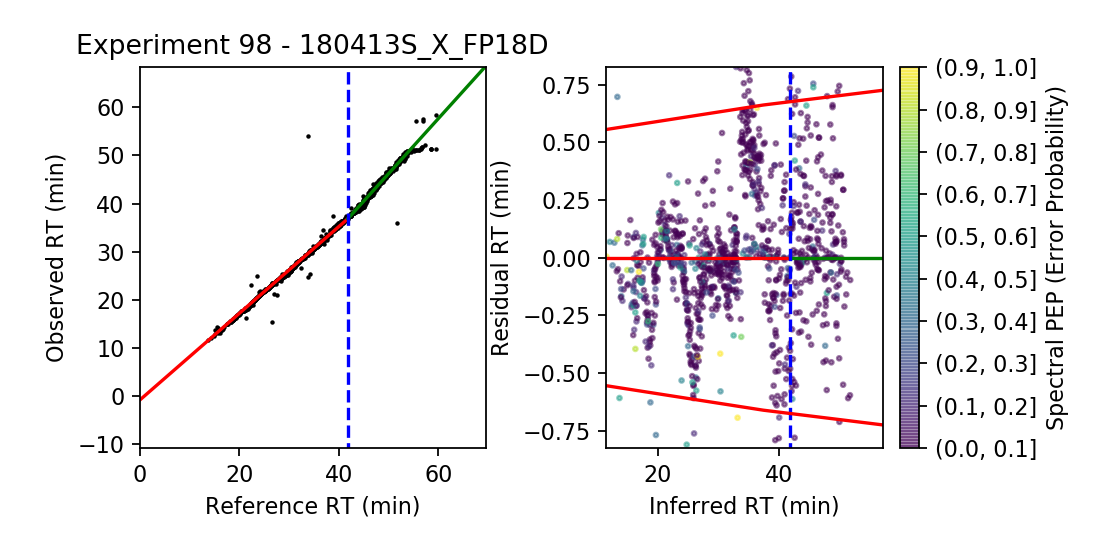

Supplement: S1 File — A optional HTML report generated by the dart_id Python script. The report gives a summary of the alignment for each experiment, as well as a broad overview of the performance of the run as a whole, by showing aggregate increases in PSMs at a chosen confidence threshold. (ZIP) [file pcbi.1007082.s001.zip › DART-ID_SCoPE-MS_Report/figures/alignment_98_180413S_X_FP18D.png]
