# Supplementary figures and images for: DART-ID increases single-cell proteome coverage (part 3 of 3)
Source: PLoS Comput Biol. 2019 Jul 1;15(7):e1007082. doi: 10.1371/journal.pcbi.1007082 (PMC6625733; doi:10.1371/journal.pcbi.1007082)

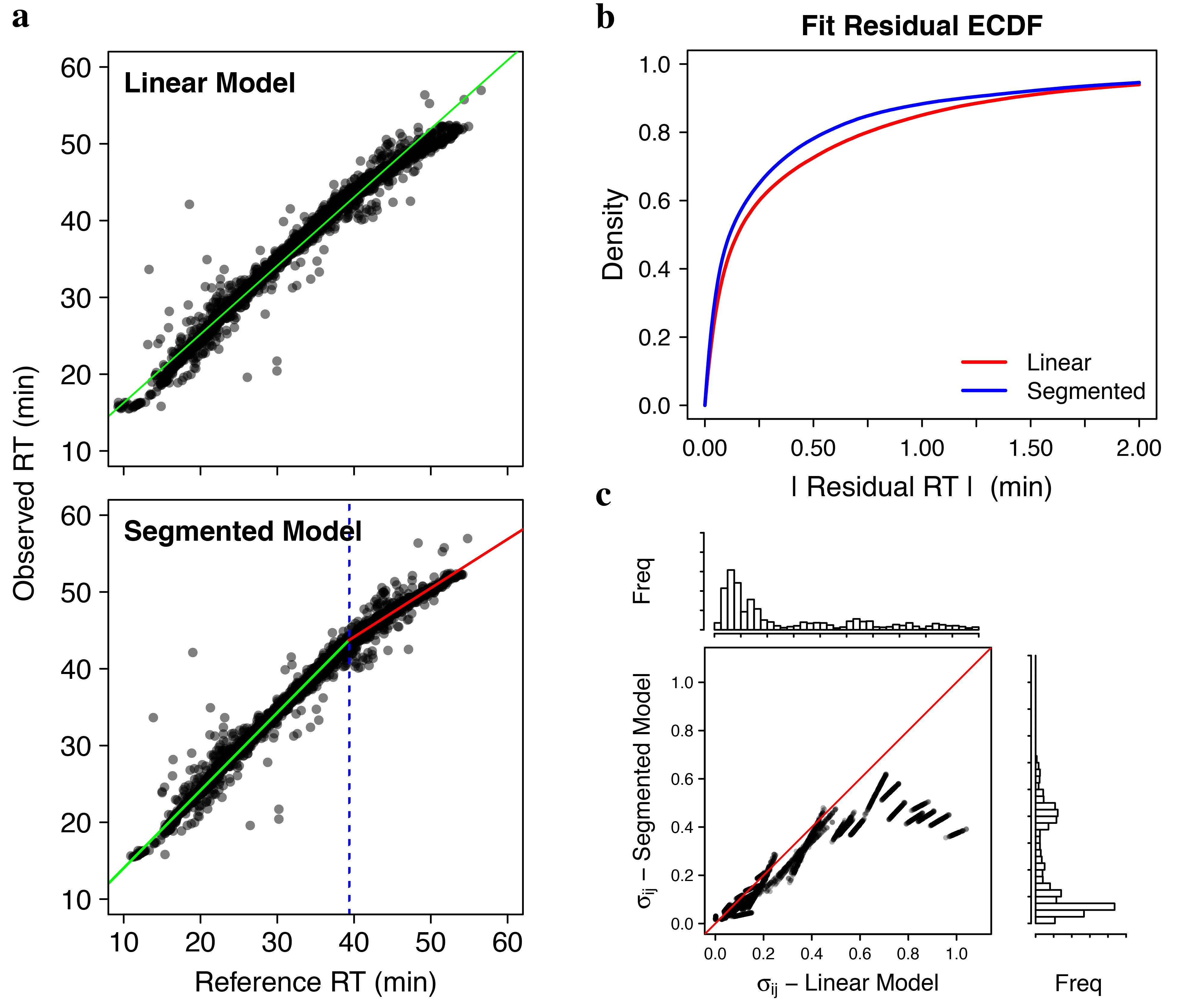

Supplement: S2 Fig — (a) The reference RT of PSMs compared with their observed RTs, and the model plotted in green line (linear fit), or green and red lines (segmented fit, representing the two segments). For the segmented fit, the inflection point is marked with the dotted blue line. Both fits were specified separately and run separately with the same input data. (b) Empirical cumulative density function (ECDF) of the residual RTs for both fits. The residual RT is defined as the Observed RT − Inferred RT, where the inferred RT is the reference RT aligned to that particular experiment via. the model function—linear or segmented. (c) Model-fitted standard deviations, σik, for each PSM as estimated by both linear and segmented fits. Points below the 45° line indicate a lower modeled RT standard deviation for the segmented fit, and vice versa. Clusters of points correspond to PSMs belonging to a particular experiment, as the PSM-specific variance of σik is mostly reliant on the experiment in which the PSM is observed. (TIF) [file pcbi.1007082.s005.tif]

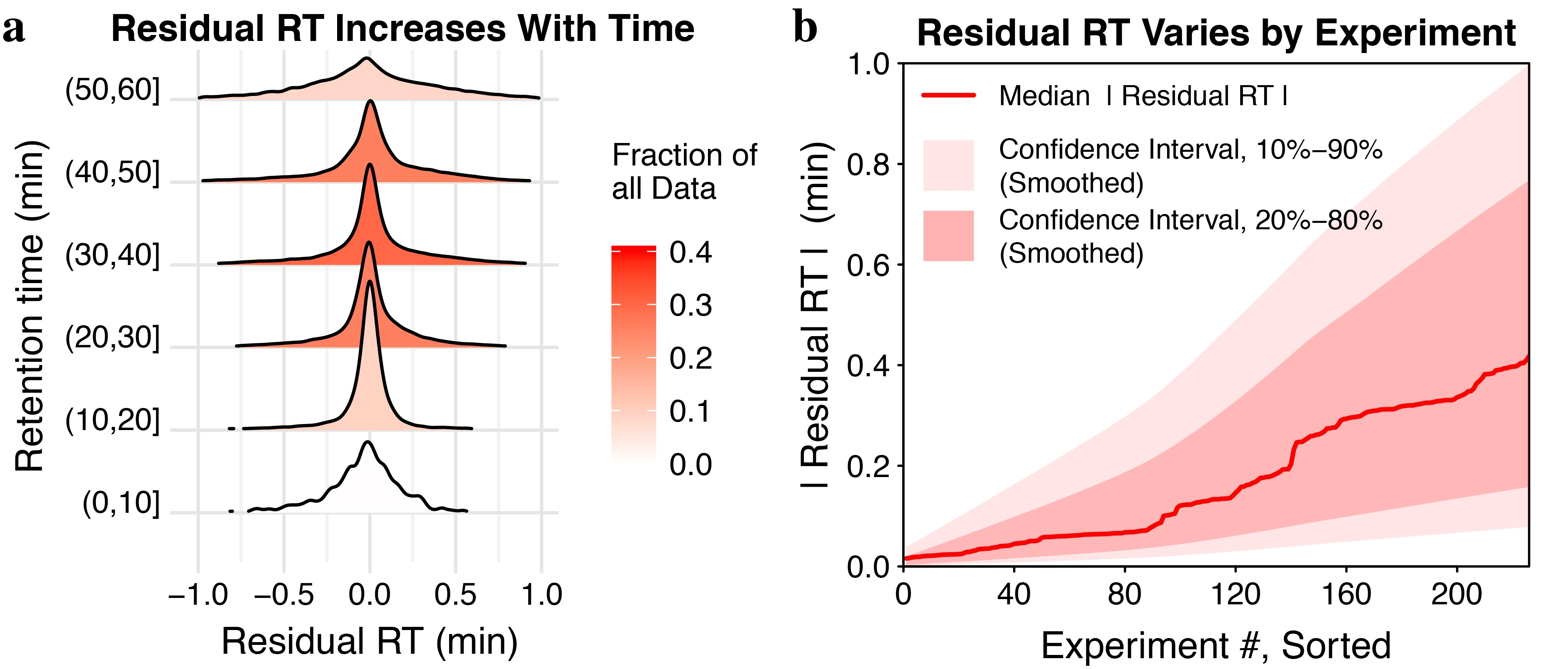

Supplement: S3 Fig — (a) Residual RT (observed RT—aligned RT) binned by RT for 60 min LC-MS runs. The gradient run is 5–35%B from 0–48 min, with a wash step of 35–100%B from 48–60 min. (b) Residual RT varying between different experiments, all 60 min LC-MS/MS runs. (TIF) [file pcbi.1007082.s006.tif]

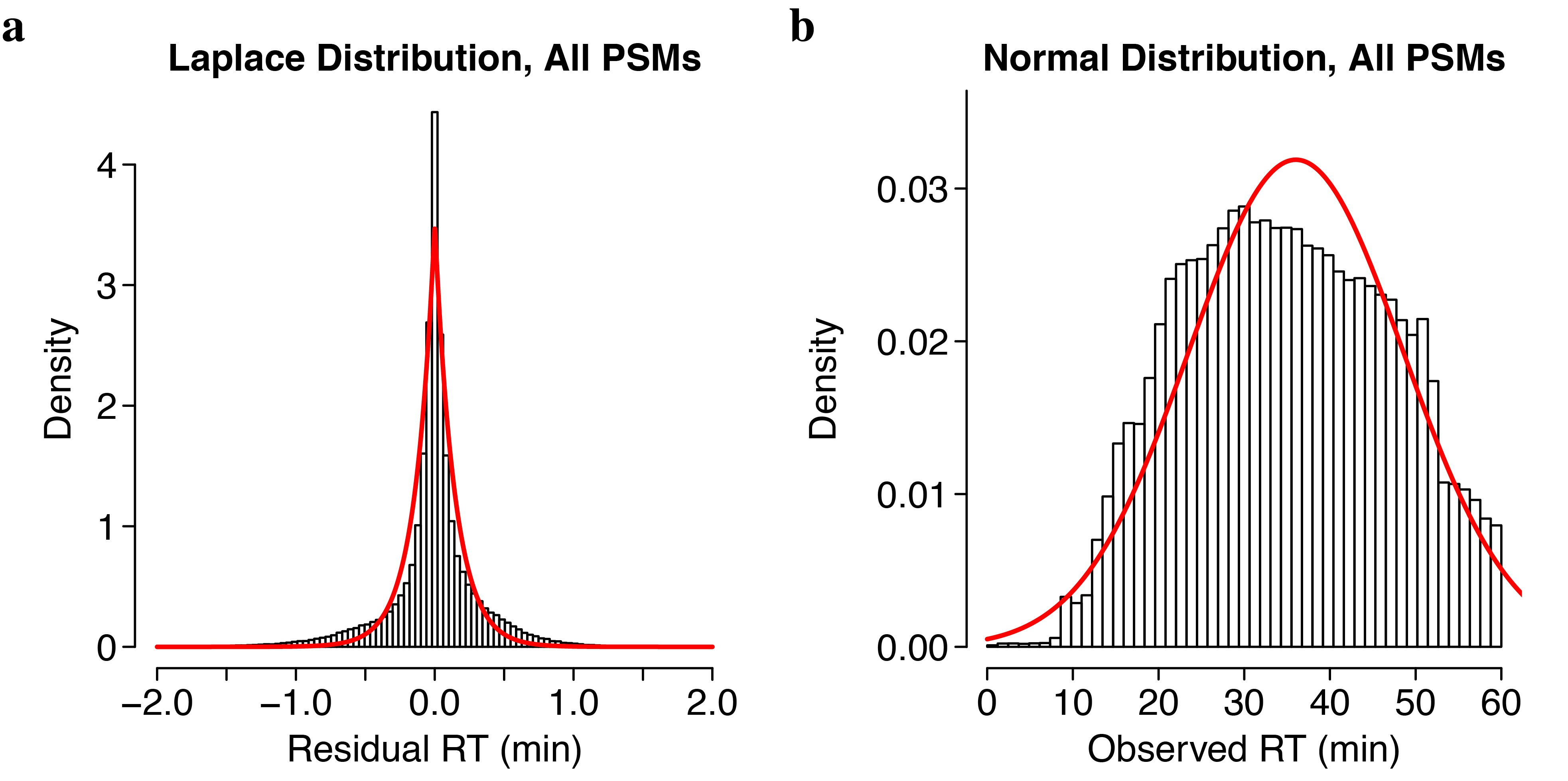

Supplement: S4 Fig — (a) Empirical distribution of all residual RTs, i.e., Observed RT − Predicted RT, and (b) all RTs. Red lines denote the distributions parametrized from the data. (TIF) [file pcbi.1007082.s007.tif]

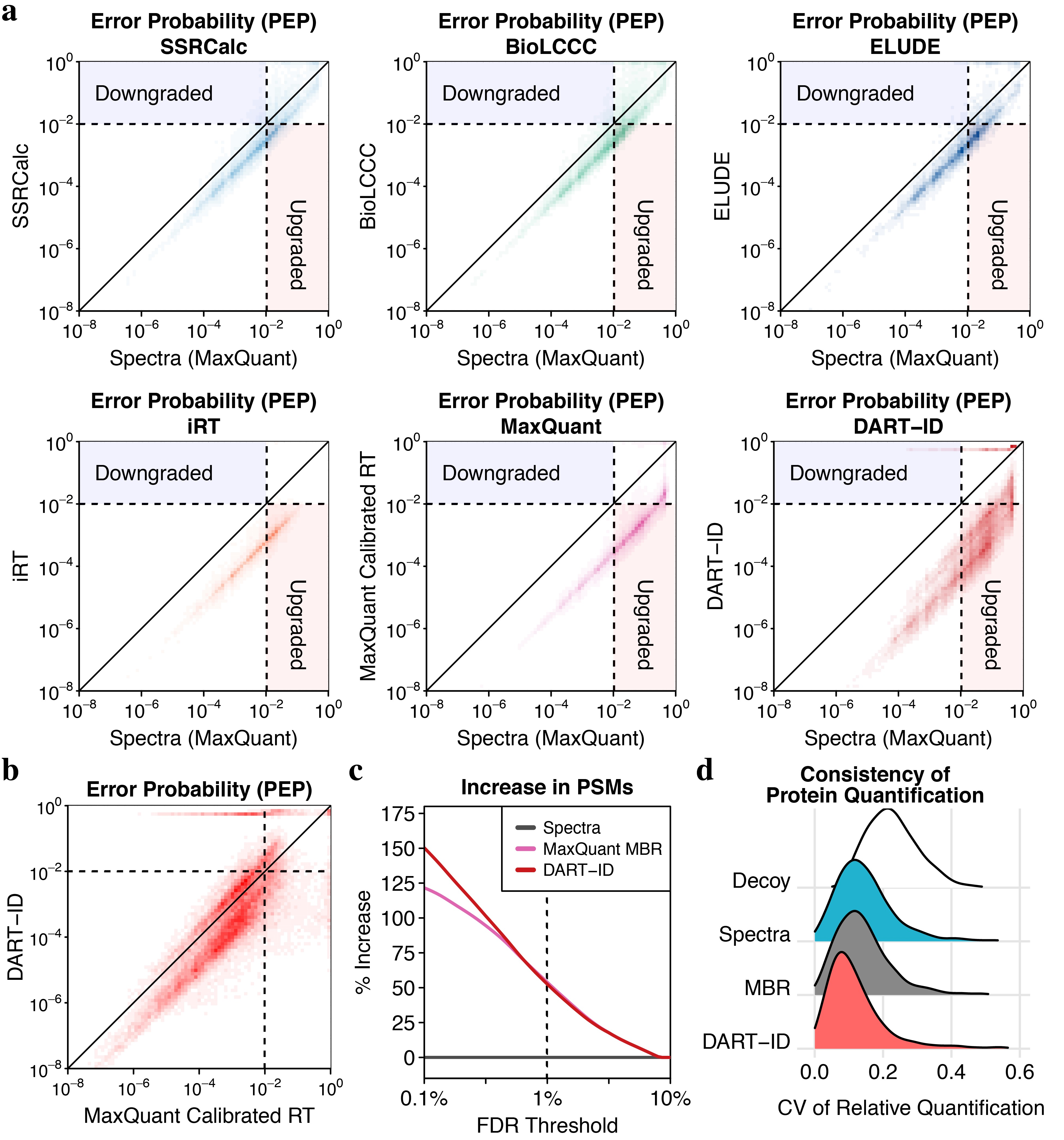

Supplement: S5 Fig — (a) 2D density distributions of posterior error probabilities (PEP) derived from spectra alone (Spectral PEP) compared to the PEP after incorporating RT evidence. The RT estimates are the same as the ones shown in Fig 2. (b) Comparison of updated PEP derived from DART-ID and MaxQuant RT estimates. (c) Increase in confident PSMs at set confidence threshold using updated PEPs. (d) Validation of upgraded PSMs with quantification variance within proteins. (TIF) [file pcbi.1007082.s008.tif]

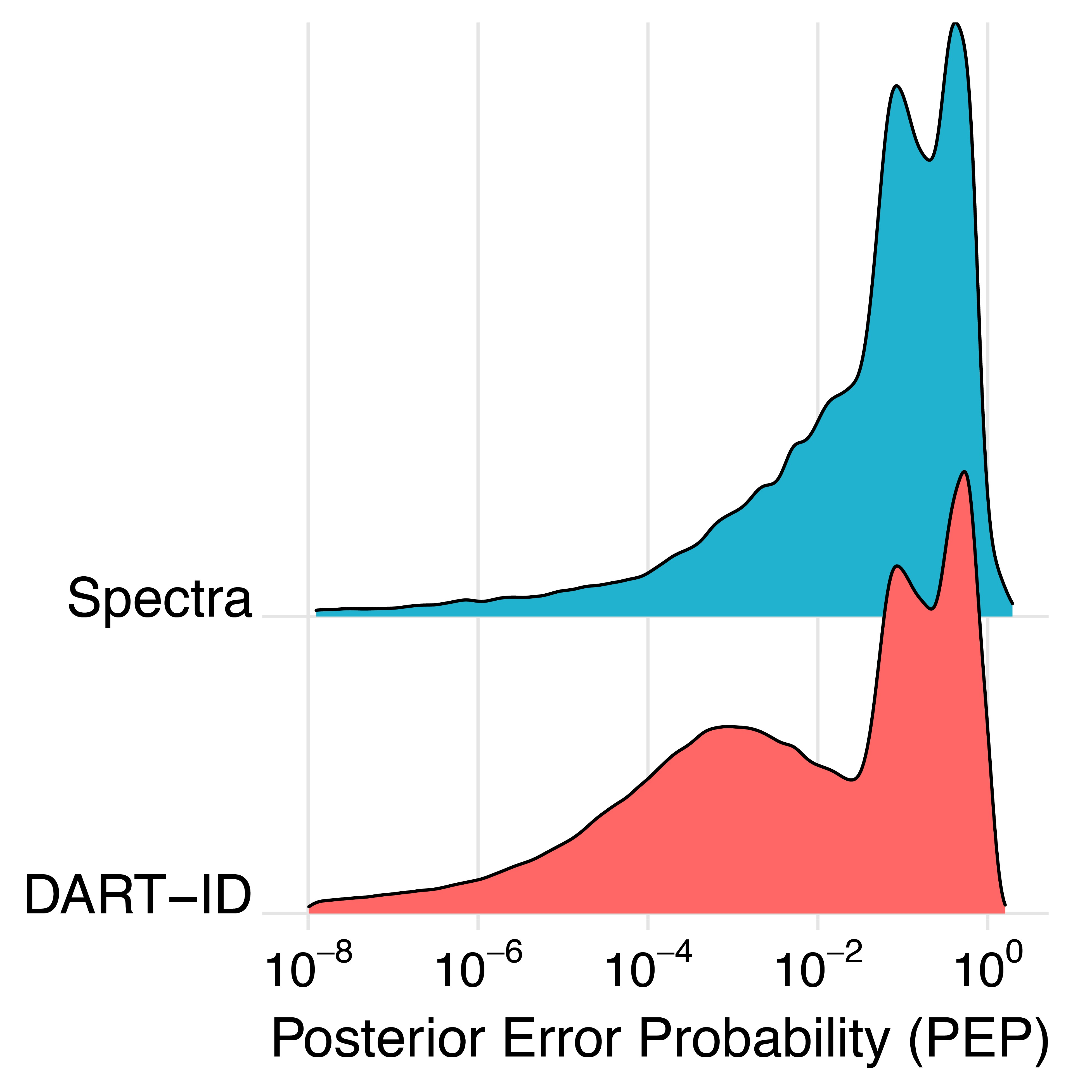

Supplement: S6 Fig — The bimodality of the DART-ID distribution suggests that DART-ID’s use of RTs helps cleanly separate correct from incorrect PSMs. (TIF) [file pcbi.1007082.s009.tif]

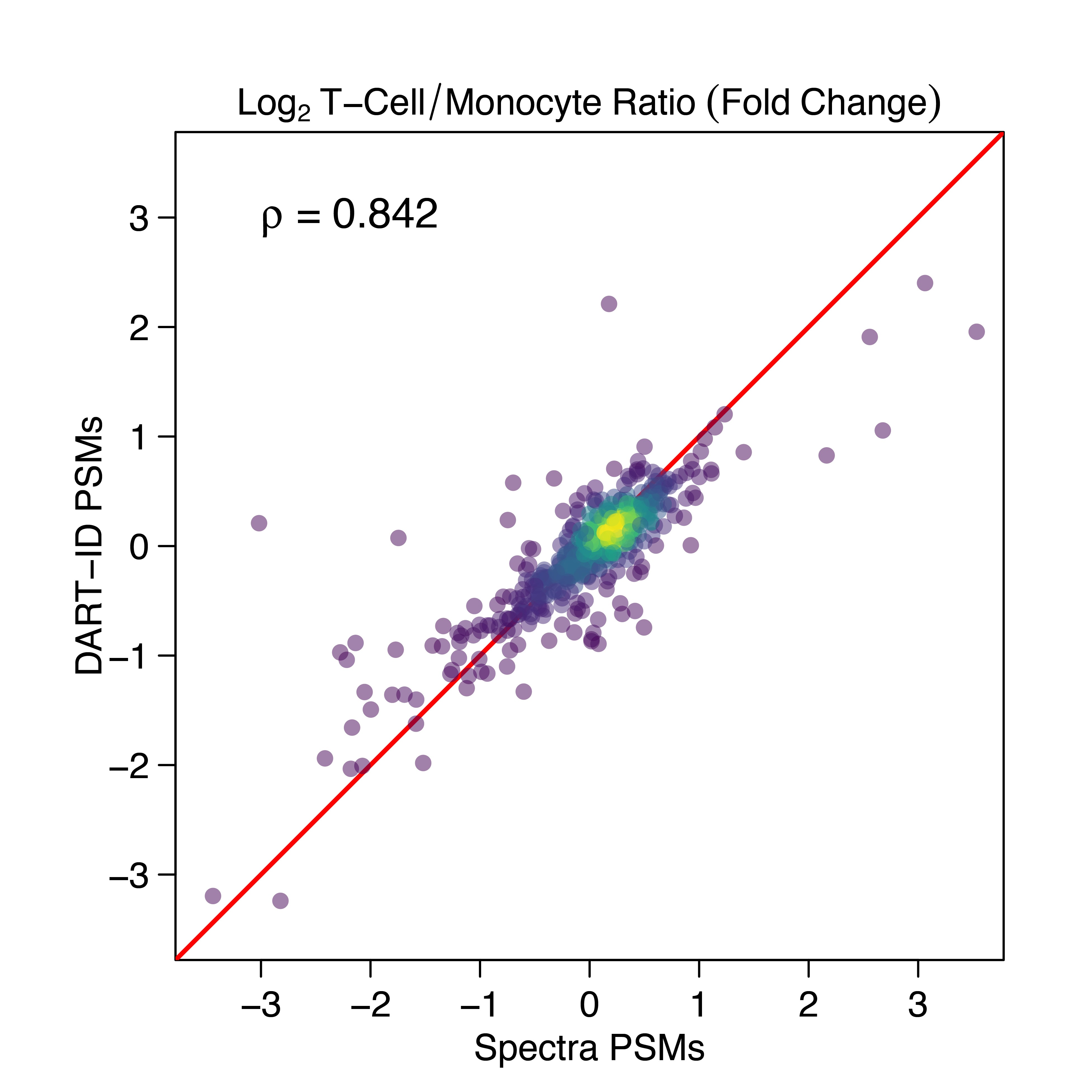

Supplement: S7 Fig — The fold change in normalized RI intensity (T-cell/monocyte), from common proteins between the Spectra and DART-ID PSM sets. We included all proteins—not just those that are significantly (< 1% FDR) differentially abundant. (TIF) [file pcbi.1007082.s010.tif]

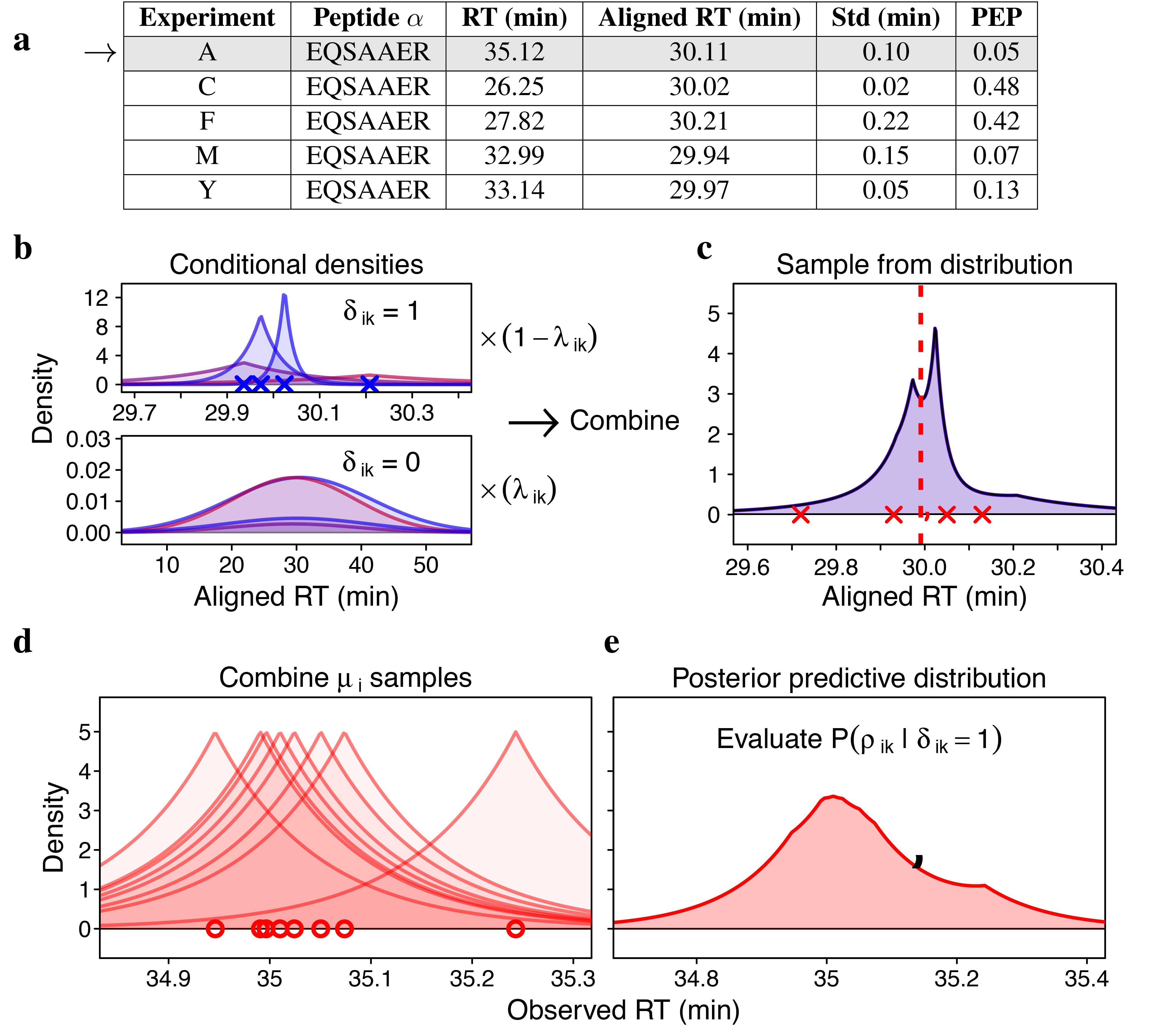

Supplement: S8 Fig — (a) The conditional probability distribution of RT given a correct peptide sequence assignment incorporates evidence about that peptide sequence across many different experiments. “Aligned RT” is the RT after applying the alignment function, and “Std” is inferred RT standard deviation for the peptide in the given experiment. (b) For each RT observation for a sequence in an experiment, we infer two distributions: one corresponding to RT density given a correct PSM and the other to an incorrect PSM match. These densities are weighted by the 1-PEP and the PEP respectively and summed to produce the marginal RT distribution. (c) The marginal RT distribution is then used to sample B bootstrap replicates of of the observed RTs. Each bootstrapped RT is then used to construct a bootstrapped reference RT for a given sequence. The reference RT is the median of the resampled RTs (in the aligned space). (d) The B bootstrap samples of μi are used to build distributions where the variance is determined by the model-derived variance of the peptide in an experiment. (e) The combination of the distributions in panel (d) forms a posterior predictive distribution for the observed RT, given that the peptide sequence assignment is correct. (TIF) [file pcbi.1007082.s011.tif]

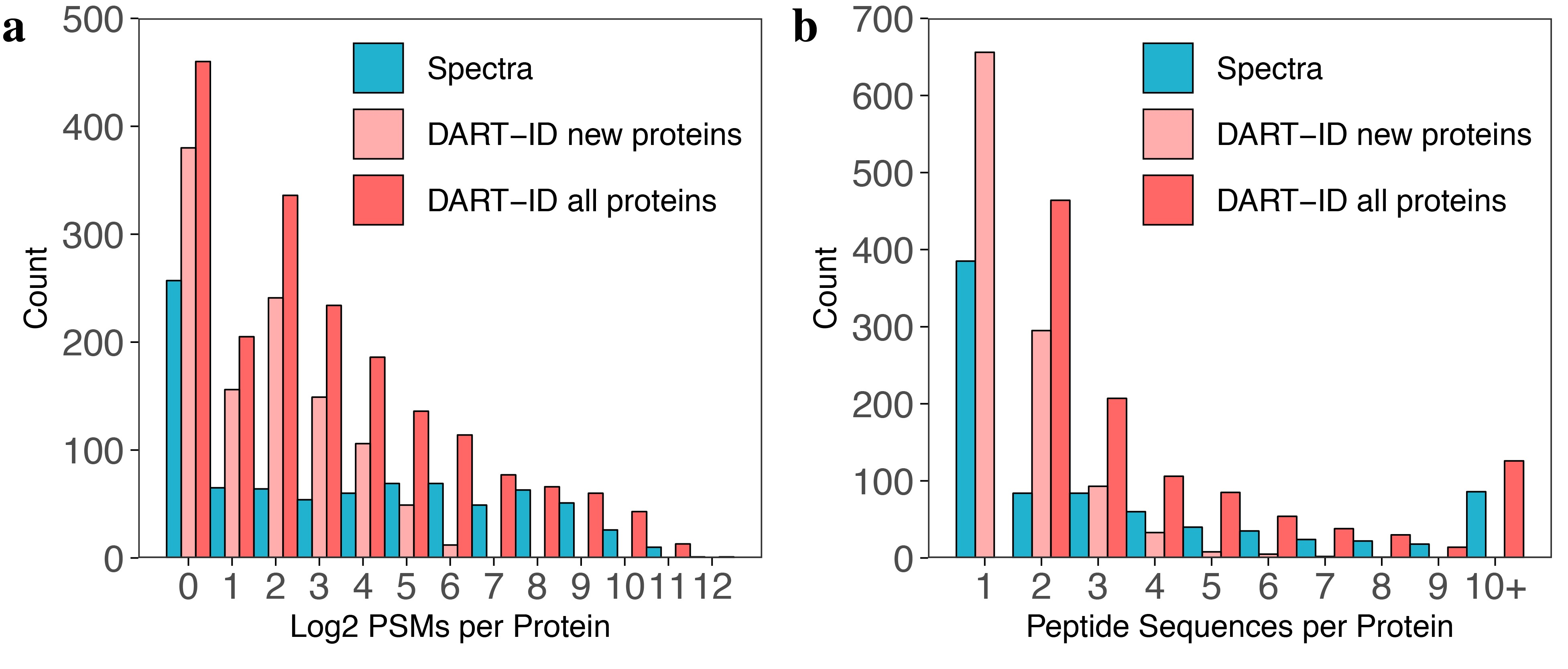

Supplement: S9 Fig — (a) Quantified PSMs per protein, including peptide sequences quantified across multiple experiments, and (b) peptide sequences quantified per protein. “Spectra” indicates proteins from PSMs identified below 1% FDR. “DART-ID new proteins” indicates PSMs boosted to below 1% FDR, that have different protein assignments from “Spectra”, i.e., this set of proteins and the “Spectra” set of proteins is disjoint. “DART-ID all proteins” contains all PSMs with updated DART-ID FDR < 1% FDR regardless of protein assignment. All PSMs are filtered at < 1% FDR at the protein level. (TIF) [file pcbi.1007082.s012.tif]
